# Synergistic anti-tumor activity and inhibition of angiogenesis by cotargeting of oncogenic and death receptor pathways in human melanoma

**DOI:** 10.1038/cddis.2014.410

**Published:** 2014-10-02

**Authors:** G Grazia, C Vegetti, F Benigni, I Penna, V Perotti, E Tassi, I Bersani, G Nicolini, S Canevari, C Carlo-Stella, A M Gianni, R Mortarini, A Anichini

**Affiliations:** 1 Human Tumors Immunobiology Unit, Department of Experimental Oncology and Molecular Medicine, Fondazione IRCCS Istituto Nazionale dei Tumori, Milan, and Medical Oncology, Università degli Studi di Milano, Milan, Italy; 2 San Raffaele Scientific Institute, URI, Milan, Italy; 3 Functional Genomics Unit, Department of Experimental Oncology and Molecular Medicine, Fondazione IRCCS Istituto Nazionale dei Tumori, Milan, and Medical Oncology, Università degli Studi di Milano, Milan, Italy; 4 Department of Oncology and Hematology, Humanitas Cancer Center, Humanitas Clinical and Research Center, Rozzano, Italy; 5 Department of Medical Biotechnology and Translational Medicine, University of Milan, Milan, Italy; 6 Medical Oncology Unit 2, Department of Medical Oncology, Fondazione IRCCS Istituto Nazionale dei Tumori, Milan, and Medical Oncology, Università degli Studi di Milano, Milan, Italy

## Abstract

Improving treatment of advanced melanoma may require the development of effective strategies to overcome resistance to different anti-tumor agents and to counteract relevant pro-tumoral mechanisms in the microenvironment. Here we provide preclinical evidence that these goals can be achieved in most melanomas, by co-targeting of oncogenic and death receptor pathways, and independently of their BRAF, NRAS, p53 and PTEN status. In 49 melanoma cell lines, we found independent susceptibility profiles for response to the MEK1/2 inhibitor AZD6244, the PI3K/mTOR inhibitor BEZ235 and the death receptor ligand TRAIL, supporting the rationale for their association. Drug interaction analysis indicated that a strong synergistic anti-tumor activity could be achieved by the three agents and the AZD6244–TRAIL association on 20/21 melanomas, including cell lines resistant to the inhibitors or to TRAIL. Mechanistically, synergy was explained by enhanced induction of caspase-dependent apoptosis, mitochondrial depolarization and modulation of key regulators of extrinsic and intrinsic cell death pathways, including c-FLIP, BIM, BAX, clusterin, Mcl-1 and several IAP family members. Moreover, silencing experiments confirmed the central role of Apollon downmodulation in promoting the apoptotic response of melanoma cells to the combinatorial treatments. In SCID mice, the AZD6244–TRAIL association induced significant growth inhibition of a tumor resistant to TRAIL and poorly responsive to AZD6244, with no detectable adverse events on body weight and tissue histology. Reduction in tumor volume was associated not only with promotion of tumor apoptosis but also with suppression of the pro-angiogenic molecules HIF1*α*, VEGF*α*, IL-8 and TGF*β*1 and with inhibition of tumor angiogenesis. These results suggest that synergistic co-targeting of oncogenic and death receptor pathways can not only overcome melanoma resistance to different anti-tumor agents *in vitro* but can also promote pro-apoptotic effects and inhibition of tumor angiogenesis *in vivo*.

The development of mutant BRAF (v-raf murine sarcoma viral oncogene homolog B)- and mitogen/extracellular signal-regulated kinase (MEK)-specific inhibitors, such as Vemurafenib, Dabrafenib and Trametinib, as well as of monoclonal antibodies targeting immune checkpoints, has markedly improved the treatment of advanced melanoma, as shown by highly significant effects, achieved in several trials, on progression-free and/or overall survival.^1, 2, 3, 4, 5^ However, a fraction of patients does not benefit from target-specific therapy or immunotherapy, and duration of clinical responses may be limited.^1, 2, 3, 4, 5^ Mechanisms of resistance to specific inhibitors^6^ and of tumor escape from immune recognition^7^ contribute to prevent induction of melanoma cell death by the new therapies and explain the urgent need for the identification of more effective approaches. Different strategies are being investigated to overcome melanoma resistance to single anti-tumor agents and to rescue tumor susceptibility to cell death, including co-targeting of constitutively active intracellular signaling pathways,^8, 9, 10^ association of target-specific drugs with inhibitors of autophagy or with endoplasmic reticulum-stress inducers^11,12^ and association of anti-tumor agents that trigger the extrinsic and the intrinsic pathway of apoptosis.^13, 14, 15^

The latter approach is based on the combination of specific inhibitors of main oncogenic pathways, which in different tumor types can modulate relevant pro- and anti-apoptotic molecules in the intrinsic pathway of cell death,^16, 17, 18^ with targeting of the extrinsic, death receptor-dependent pathway, by usage of tumor necrosis factor-related apoptosis-inducing ligand (TRAIL) or of agonistic death receptor 5 (DR5)-specific mAbs.^19^ Indeed, this approach has shown that association of MEK, pan-RAF or phosphoinositide 3-kinase (PI3K) inhibitors with TRAIL can overcome resistance to TRAIL^13, 14, 15^ and can lead to enhanced melanoma apoptosis *in vitro* through different mechanisms, including upregulation of bcl-2-like protein 11 isoform 1 (Bim) and activation of BCL2-associated X protein (Bax).^13, 14, 15^ Moreover, as hypothesized recently by Geserick *et al.*,^20^ the association of MEK or pan-RAF inhibitors with TRAIL could even be exploited as a potential approach to promote rapid elimination of most tumor cells, thus preventing the emergence of secondary resistance to BRAF inhibitors. Furthermore, the interest in the death receptor pathway, as a therapeutic target, has been recently strengthened by the evidence that TRAIL mediates disruption of the tumor-associated vasculature^21^ and by the discovery of TIC10, a drug that stimulates production of TRAIL and that exerts significant anti-tumor activities in preclinical *in vivo* models, including aggressive intracranial xenografts of human glioblastoma cells.^22^

Nevertheless, it is currently not known whether co-targeting of MEK and/or PI3K/mammalian target of rapamycin (mTOR) and of the death receptor pathway in melanoma can overcome intrinsic resistance to each of the anti-tumor agents in most instances, irrespective of the different genetic make-up of the tumors, and whether this approach can exert synergistic, rather than additive, anti-melanoma effects. Furthermore, it remains to be verified whether the combination of MEK or PI3K/mTOR inhibitors with death receptor agonists (such as TRAIL itself or DR5-specific mAbs) may also exert significant pro-apoptotic effects *in vivo* on melanoma xenografts and whether this is associated with inhibition of relevant pro-tumoral processes in the tumor microenvironment.

To address these issues, in this study we evaluated the anti-melanoma activity *in vitro* and *in vivo* of two- or three-drug associations using TRAIL, the MEK 1/2 inhibitor AZD6244/Selumetinib, which has significant clinical activity in melanoma,^23^ and the PI3K/mTOR inhibitor BEZ235, currently in clinical trials in different solid tumors, including melanoma (source www.clinicaltrials.gov). The results indicated that the three-agent (AZD6244/BEZ235/TRAIL) and two-agent (AZD6244/TRAIL) combinations exerted synergistic pro-apoptotic effects on most melanomas in a large panel. These results were observed even on melanoma cell lines resistant to TRAIL or to the inhibitors and independently of their BRAF, neuroblastoma RAS viral (v-ras) oncogene homolog (NRAS), p53 and phosphatase and tensin homolog (PTEN) status. Moreover, an *in vivo* model showed that the AZD6244/TRAIL association promoted melanoma apoptosis associated with marked inhibition of angiogenesis.

## Results

### Independent susceptibility profiles to target-specific inhibitors and TRAIL in human melanomas

**Figure 1:**
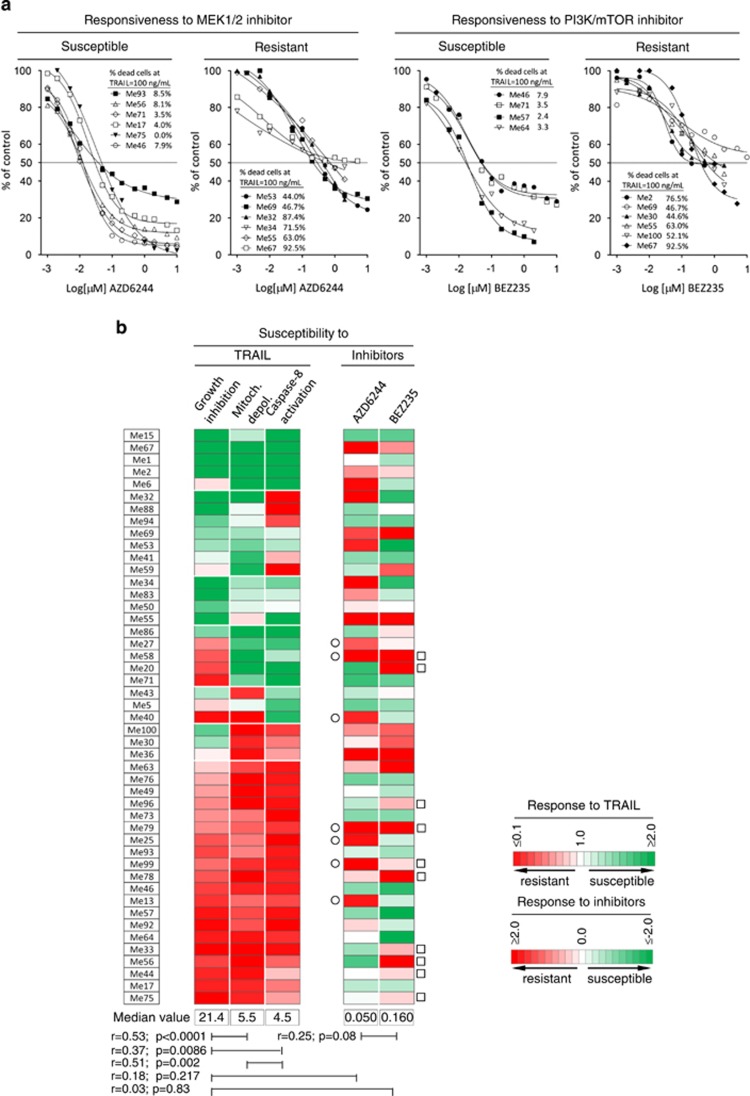
Independent susceptibility profiles of melanoma cells to MEK, PI3K/mTOR inhibitors and TRAIL. (**a**) Responsiveness of selected melanoma cell lines to AZD6244, BEZ235 and TRAIL, by MTT assay. (**b**) Cell viability (by MTT assay), mitochondrial depolarization (by TMRE assay) and caspase-8 cleavage (by flow cytometry) in response to TRAIL (100 ng/ml, 48 h) shown by a color code indicating the ratio of the values for each tumor to the median value of each parameter in the whole panel of cell lines. Responsiveness to AZD6244 and BEZ235 (by MTT assay, 48 h) shown by a color code indicating the log-transformed ratio of the IC_50_ of each tumor to the median IC_50_ of each inhibitor in the whole panel. Data clustered by the three parameters in response to TRAIL. Statistical analysis by Spearman correlation analysis. TRAIL-resistant melanoma cell lines with IC_50_ to AZD6244 (○) or to BEZ235 (□) >0.2 *μ*M

We asked whether concomitant resistance to MEK, PI3K/mTOR inhibitors and to the death receptor ligand TRAIL is frequent in human melanoma. To this end, a panel of 49 melanoma cell lines (Supplementary Table S1), with known BRAF, NRAS, PTEN and p53 status, was characterized for susceptibility to AZD6244, BEZ235 and TRAIL. Several lines responsive (IC_50_<0.05 *μ*M) to AZD6244 or BEZ235 were TRAIL resistant (<10% dead cells at TRAIL 100 ng/ml), whereas a number of cell lines resistant to these inhibitors (IC_50_≥0.2 *μ*M) showed good susceptibility to TRAIL (representative data in Figure 1a). Spearman analysis of raw data shown in Supplementary Table S1 indicated no significant correlation between susceptibility to the inhibitors and to TRAIL (Figure 1b). Indeed, concomitant resistance to AZD6244 and TRAIL or to BEZ235 and TRAIL was found in only 7 and 10 cell lines, respectively (marked by open circles and squares, Figure 1b), supporting the rationale for association of these anti-tumor agents. We then found that response to TRAIL correlated significantly not only with caspase-8 cleavage (*P*=0.0086) but even with mitochondrial depolarization (*P*<0.0001, Figure 1b and Supplementary Table S1), in agreement with the notion that melanoma cells behave as type II cells in response to TRAIL, requiring activation of the intrinsic pathway of cell death.^24^ This latter finding provided also a mechanistic rationale for the co-targeting of oncogenic and death receptor pathways. In fact, TRAIL triggers the extrinsic pathway of apoptosis, while inhibitors, such as AZD6244 or BEZ235, can promote cell death by modulating pro- and anti-apoptotic molecules in the intrinsic pathway of cell death.^16, 17, 18^

In addition, TRAIL-R1 (DR4) and TRAIL-R2 (DR5) were always expressed *in vitro*, although with different staining intensity (Supplementary Figure S1a), and the levels of expression of DR5 correlated significantly with responsiveness to TRAIL, indicating the relevance of such receptor (Supplementary Figure S1a). *In vivo* TRAIL-R2/DR5 expression was confirmed in neoplastic cells from melanoma metastases (Supplementary Figure S1b), supporting the choice of targeting this pathway in melanoma.

### Co-targeting of oncogenic and death receptor pathways exerts synergistic anti-tumor effects in most melanomas, irrespective of genetic background, and overcomes resistance to each agent

Melanoma cell lines susceptible to AZD6244, BEZ235 and TRAIL (Me1 and Me83) or resistant to TRAIL and poorly responsive to AZD6244 (Me13 and Me6) were selected for drug interaction analysis. All possible two- and three-drug combinations were evaluated by the Chou and Talalay method.^25^ Outcome of drug interaction, in terms of synergism/antagonism and of fraction affected (FA) values, was markedly dependent on the specificity of the combination and on dosing of each agent, as indicated by FA *versus* Combination Index (CI) plots (Supplementary Figure S2a). However, the AZD6244–BEZ235–TRAIL and the AZD6244–TRAIL combinations could achieve strong synergism (CI<0.3), with high FA values, in all four cell lines, and the lowest CI values were observed when AZD6244 was used at 0.05 *μ*M (asterisks, Supplementary Figure S2a). Western blotting analysis confirmed effective inhibition of *P*-ERK, by AZD6244, and of *P*-AKT by BEZ235 in different melanoma cell lines (Supplementary Figure S3).

**Figure 2:**
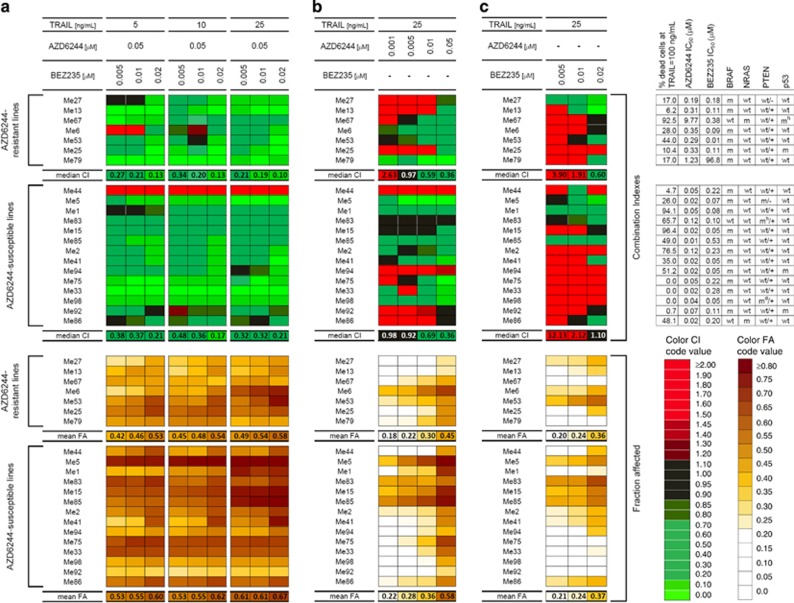
Synergistic anti-tumor interaction of AZD6244-BEZ235-TRAIL and AZD6244-TRAIL combinations but not of BEZ-TRAIL association in melanoma cells. Drug interaction analysis by Chou and Talalay method in two groups of melanoma cell lines with different responsiveness to AZD6244 (resistant lines: IC_50_≥0.2 *μ*M, *n*=7; susceptible lines: IC_50_≤0.2 *μ*M, *n*=14) treated with the association of (**a**) AZD6244, BEZ235 and TRAIL or (**b**) AZD6244 and TRAIL or (**c**) BEZ235 and TRAIL. Combination indexes (CI, upper panel), and fraction affected (FA, lower panel) by a color code shown in the lower right hand side of the figure. Red indicates antagonism while green indicates synergy, as shown in Supplementary Figure S2b. Susceptibility to TRAIL, AZD6244 and BEZ235 and main molecular features of all cell lines summarized in the right hand side panel (m: mutant; wt: wild type; +, −: expression/lack of expression of PTEN by western blotting). Numbers at the bottom of the upper and lower panels: median CI values and mean FA values, respectively, for each combination of drugs and TRAIL doses

Drug interaction analysis was then extended to a panel of 21 melanoma cell lines with distinct susceptibility profiles to the inhibitors and TRAIL. Analysis of the AZD6244–BEZ235–TRAIL combinatorial treatment confirmed a strong synergistic interaction with CI<0.3 and high levels of FA in all AZD6244-resistant lines (*n*=7) and in 13/14 AZD6244-susceptible lines, when AZD6244 was used at 0.05 *μ*M, irrespective of TRAIL and BEZ235 concentrations (Figure 2a). In contrast, strong antagonism emerged frequently in most lines at [AZD6244]<0.05 *μ*M and at [BEZ235]<0.02 *μ*M (Supplementary Figure S4a). At the most effective AZD6244 dosing, the synergism between target-specific inhibitors and TRAIL was observed not only in cell lines resistant to AZD6244 but even in those with high IC_50_ values to BEZ235, or completely resistant to TRAIL, and irrespective of BRAF, NRAS, p53 and PTEN status (Figure 2, right hand table). Moreover, increasing doses of BEZ235 and of TRAIL were associated with further improvement in either CI (Figure 2a) or FA values (Supplementary Figure S5 for box and whiskers plots of FA data). Detailed statistical analysis of FA data (Supplementary Table S2) indicated that significant increase of FA values could be observed when: (a) TRAIL was used at the highest dose (25 ng/ml) in the AZD6244–BEZ235–TRAIL combination compared with 5 and 10 ng/ml; (b) adding BEZ235 to the AZD–TRAIL association; (c) adding TRAIL to the AZD6244–BEZ235 association. In the same panel of cell lines, strong synergism could be observed even by the AZD6244–TRAIL combination, again when AZD6244 was used at 0.05 *μ*M (Figure 2b, for [TRAIL]=25 ng/ml, and Supplementary Figure S4b, for [TRAIL]=5 or 10 ng/ml and Supplementary Figure S6a for box and whiskers plot of FA data). In contrast, the BEZ235-TRAIL combinatorial treatment showed marked antagonism and poor fraction affected at in most instances (Figure 2c for [TRAIL]=25 ng/ml, Supplementary Figure S4c, for [TRAIL]=5 or 10 ng/ml and Supplementary Figure S6b for box and whiskers plot of FA data). Synergistic interaction of AZD6244–BEZ235–TRAIL and AZD6244–TRAIL associations was confirmed also in melanoma cells freshly isolated from surgical samples (data not shown). Taken together, these results indicated that association of MEK and PI3K/mTOR inhibitors with TRAIL, or MEK blockade with TRAIL, leads to synergistic anti-tumor effects on most melanoma cell lines, even on inhibitor- or TRAIL-resistant ones.

### Combinatorial treatments rescue susceptibility of melanoma cells to caspase-dependent apoptosis

**Figure 3:**
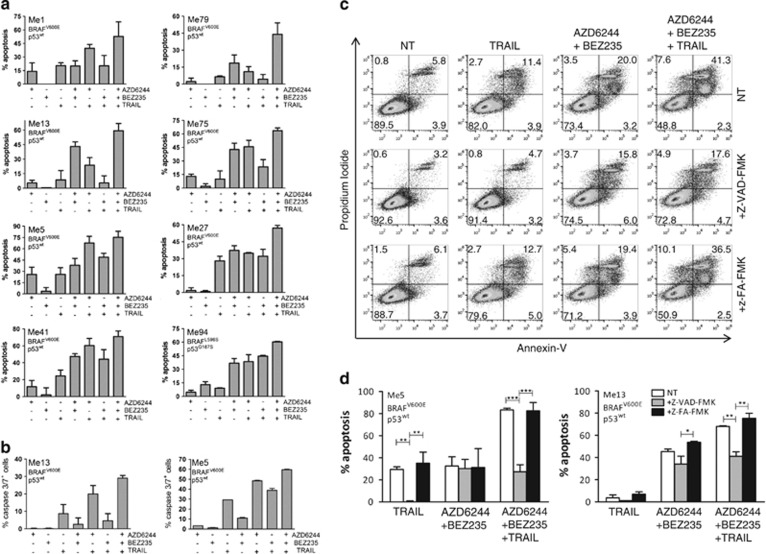
Combinatorial treatments promote caspase-dependent melanoma apoptosis. (**a**) Melanoma cells were treated with AZD6244 (0.05 *μ*M), BEZ235 (0.05 *μ*M) and TRAIL (25 ng/ml) and their combinations for 72 h, and apoptosis was assessed by Annexin-V/PI assay. Results shown as sum of early (annexin-V^+^ PI^−^) and late (annexin-V^+^ PI^+^) apoptosis values. (**b**) caspase 3/7 activation in two melanoma cell lines treated as in panel (**a**) but assessed at 24 h. (**c** and **d**) Annexin-V/PI assays ((**c**), single experiment; (**d**), average of three experiments) in melanoma cells (Me 5, Me13) treated as in panel (**a**), in the presence of the pancaspase inhibitor z-VAD-fmk or of the negative control peptide z-FA-fmk. (**a**, **b**, **d**), Mean values (±S.D.) for three independent experiments; (**d**), statistical analysis by ANOVA followed by SNK test. ***P<0.001, **P<0.01; *P<0.05

To uncover the main biological processes behind the synergistic interactions, we carried out whole genome gene expression analysis of Me13 cells, resistant to TRAIL and poorly responsive to AZD6244, upon treatment with inhibitors and TRAIL. Genes significantly modulated by the AZD6244–BEZ235–TRAIL association, identified by class comparison and visualized by Edwards-VENN diagrams (light blue circles in Supplementary Figure S7a),^26^ were subjected to downstream effects analysis. Significant upregulation of the functions ‘cell death' and ‘apoptosis' and downregulation of the functions ‘tumorigenesis', ‘cell migration' and ‘proliferation' (Supplementary Figure S7b and Supplementary Table S3) were identified. Analysis of genes modulated by the AZD6244–TRAIL association confirmed enhancement of the functions ‘cell death' and ‘apoptosis' (Supplementary Figures S8a and b and Supplementary Table S4). By annexin-V/PI flow cytometry assays (Figure 3a), significantly higher levels of apoptosis, compared with single agents, were observed in 5/8 cell lines by AZD6244–TRAIL or by BEZ235–TRAIL treatments (*P*<0.01 by analysis of variance (ANOVA) followed by Student–Newman–Keul (SNK) test) and in 8/8 cell lines by the AZD6244–BEZ235–TRAIL combination (*P*<0.01 in 7/8 lines and *P*<0.05 in 1/8 lines). Enhanced melanoma apoptosis (Supplementary Figure S9) was also observed by the association of inhibitors with membrane-bound TRAIL,^27^ as well as when TRAIL was associated with different MEK (PD0325901) and mTOR (rapamycin) inhibitors (Supplementary Figure S10).

Caspase activation assays indicated significant activation of caspase 3/7, by AZD6244–BEZ235–TRAIL and AZD6244–TRAIL combinations, compared with single agents (*P*<0.01) and to AZD6244–BEZ235 (*P*<0.01, Figure 3b), as well as of caspase-8 and 9 (data not shown). A pan caspase inhibitor (z-vad-fmk) completely abolished the increase in apoptosis induced by the addition of TRAIL to the AZD6244–BEZ235 combination, even in TRAIL-resistant (Me13) or in weakly susceptible (Me5) melanomas, but did not impact on cell death promoted by the AZD6244–BEZ235 combination (Figures 3c and d). Similar results were observed by the AZD6244–TRAIL combinatorial treatment (Supplementary Figure S11). Taken together, these results indicated that co-targeting of MEK and PI3K/mTOR pathways and of the death receptor pathway has synergistic anti-melanoma activity likely mediated by enhanced induction of caspase-dependent apoptosis.

### Pro- and anti-apoptotic molecules in the extrinsic and intrinsic apoptosis pathways are modulated by combinatorial treatments

**Figure 4:**
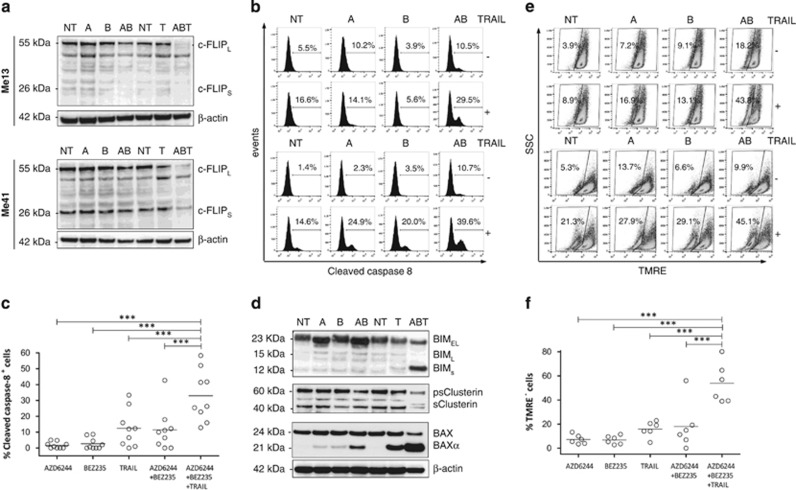
Modulation of pro- and anti-apoptotic molecules by melanoma treatment with target-specific inhibitors, TRAIL and their combination. (**a**) Western blotting analysis for c-FLIP expression and (**b**) flow cytometry analysis for cleaved caspase-8 in two melanoma cell lines (Me13 and Me41) treated with AZD6244 (A), BEZ235 (B), TRAIL (T) or the indicated combinations. (**c**) Cleaved caspase-8 analysis in a panel of nine cell lines. (**d**) Western blotting analysis for expression of BIM, clusterin and BAX in Me13 cells treated as in panel (**a**). (**e**) TMRE analysis for mitochondrial depolarization in two melanoma cell lines (Me 13 and Me 41) treated as in panel (**a**). (**f**) TMRE assay in a panel of six melanoma cell lines. The numbers in flow cytometry panels in (**b**) and (**e**) indicate the percentage of caspase-8^+^ cells and the percentage of TMRE^−^ cells, respectively. Statistical analysis in panels (**c** and **f)** by ANOVA followed by SNK test; ***P<0.001

To gain insight into the mechanisms leading to enhanced activation of caspase-dependent apoptosis, we tested whether combinatorial treatments affected expression of pro- and anti-apoptotic molecules in the extrinsic and intrinsic pathways of cell death. Western blotting analysis indicated that the AZD6244–BEZ235–TRAIL combination induced the most marked downregulation of the caspase-8 inhibitor c-FLICE-like inhibitory protein (c-FLIP), with effects seen on both c-FLIP_L_ and/or c-FLIP_S_ (Figure 4a). This occurred not only in Me41, partially responsive to TRAIL and susceptible to the inhibitors but also in Me13 cells, poorly responsive to AZD6244 and resistant to TRAIL. The same association induced significant caspase-8 cleavage (Figures 4b and c). The efficacy of this combinatorial treatment was documented also by analysis of Bcl-2 family members: upregulation of the pro-apoptotic isoforms BIM_S_ and BAX*α*,^28,29^ downregulation of two isoforms (ps and s) of the Bax inhibitor clusterin^30^ (Figure 4d), and downmodulation of myeloid cell leukemia 1 (Mcl-1) (Supplementary Figure S12) and BH3 interacting domain death agonist (BID) (data not shown) were found. In agreement with the known role of these Bcl-2 family members in the mitochondrial pathway of cell death,^31^ the AZD6244–BEZ235–TRAIL combination also promoted the strongest increase in mitochondrial depolarization compared with single agents and to the AZD6244–BEZ235 combination (Figures 4e and f). Enhanced modulation of c-FLIP, and upregulation of BIM_S_ and BAX*α*, but not of clusterin, compared with single agents (Supplementary Figures S13a and b), as well as caspase-8 cleavage and mitochondrial depolarization (Supplementary Figures S13c and d) were confirmed also for the AZD6244–TRAIL association.

**Figure 5:**
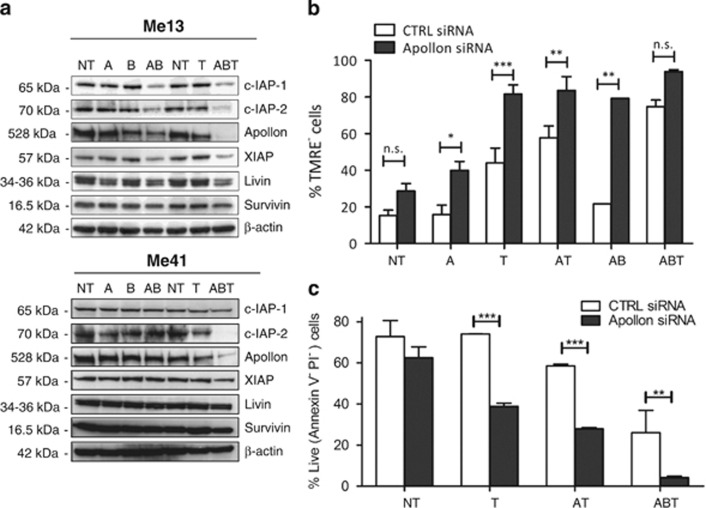
Modulation of IAP proteins and role of Apollon in the response of melanoma to the combination of target-specific inhibitors and TRAIL. (**a**) Modulation of IAP proteins by treatment of two melanoma cells lines (Me13 and Me41) with AZD6244 (A), BEZ235 (B) TRAIL (T) and their combinations (AB, ABT); NT: untreated. (**b** and **c**) Effect of Apollon silencing on mitochondrial depolarization (**b**) and cell death (**c**) in Me41 cells treated with the indicated agents and their combinations. (**b** and **c**) Mean values (±S.D.) for three independent experiments; statistical analysis in panels (**b** and **c**) by ANOVA followed by SNK test; ***P<0.001. **P<0.01. *P<0.05. NS, not significant

We then assessed the potential role of several X-linked inhibitor of apoptosis (IAP) family members. Treatment of two melanoma cell lines (Me13 and Me41) with the AZD6244–BEZ235–TRAIL combination induced strong downmodulation of c-IAP1, c-IAP2, XIAP and Apollon compared with the effects of single agents and to AZD6244–BEZ235 treatment (Figure 5a). Similarly, the AZD6244–TRAIL association was more effective than single agents in downmodulating these IAPs (Supplementary Figure S14). The giant IAP Apollon was recently shown by us to have a relevant role in suppressing melanoma response to MEK inhibitors and to TRAIL.^27^ Silencing experiments, by previously validated siRNA,^27^ confirmed the central role of Apollon downmodulation in promoting the apoptotic response of melanoma cells even to these combinatorial treatments. In cells treated with TRAIL only, or with AZD6244-BEZ235, Apollon silencing led to mitochondrial depolarization (Figure 5b) and apoptosis levels (Figure 5c) similar to those found in cells treated with the AZD6244–TRAIL or the AZD6244–BEZ235–TRAIL combination.

Taken together, these results suggest that association of TRAIL with co-targeting of MEK and PI3K/mTOR, or with MEK blockade only, promotes effective melanoma cell death by modulating key molecules involved in regulation of both the extrinsic and intrinsic apoptosis pathways.

### Co-targeting of MEK and death receptor pathways has anti-tumor activity *in vivo* by promotion of melanoma cell death and inhibition of angiogenesis

Our goal was then to verify whether co-targeting of oncogenic and death receptor pathways could exert significant anti-tumor effects *in vivo* and whether such activity was associated with effects on the tumor microenvironment. To this end, we decided to investigate the effects of treatment with AZD6244 and TRAIL, as both the three-drug (AZD/BEZ/TRAIL) and two-drug (AZD/TRAIL) associations shared synergistic anti-tumor activity *in vitro* and similar mechanisms of promotion of apoptosis.

Downstream effect analysis on the set of genes modulated by the AZD6244–TRAIL association (Supplementary Figure S8a) showed evidence for inhibition of functions associated with migration and proliferation of endothelial cells (Supplementary Figure S8b and Supplementary Table S4), in addition to upregulation of the functions ‘cell death and apoptosis'. Upstream regulator analysis predicted a highly significant inhibition of several angiogenesis-related master regulators, including transforming growth factor *β*1 (TGF*β*1), hepatocyte growth factor, epidermal growth factor, v-myc avian myelocytomatosis viral oncogene homolog (Myc), hypoxia inducible factor 1 alpha subunit (HIF1*α*) and vascular endothelial growth factor alpha (VEGF*α*) (Supplementary Table S5), in addition to microphthalmia-associated transcription factor activation, an expected effect of extracellular signal-regulated kinase (ERK) pathway inhibition.^32^ Protein array experiments on Me13 cells confirmed strong downmodulation of several angiogenesis-related molecules by the AZD6244-TRAIL association, compared with untreated or to AZD6244-treated cells (Supplementary Figure S15). Western blotting analysis showed a decrease in Myc and HIF1*α* levels upon treatment of Me13 cells with AZD6244–TRAIL or with AZD6244 only (Supplementary Figure S16a). Overnight treatment of melanoma cells with AZD6244–TRAIL led to a more pronounced suppression of VEGF*α* and TGF*β*1 secretion, compared with the effects induced by AZD6244 alone (Supplementary Figure S16b). Control apoptosis assays indicated that the inhibitory effect by AZD6244–TRAIL treatment on VEGF*α* secretion at 18 h was not explained by induction of melanoma cell death (Supplementary Figure S16c). Moreover, significant inhibition of VEGF*α* secretion by overnight AZD6244–TRAIL treatment was confirmed in 4 out of the 5 additional melanoma cell lines (Supplementary Figure S16d).

**Figure 6:**
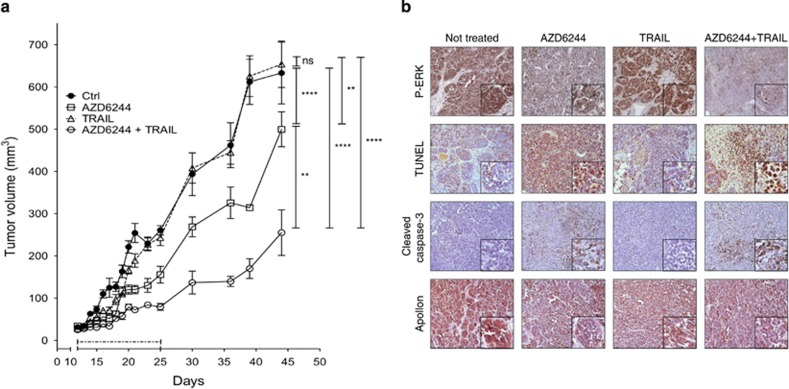
Tumor growth inhibition *in vivo*, by AZD6244 and TRAIL treatment, is associated with promotion of melanoma apoptosis and reduction of Apollon expression. (**a**) Female SCID mice (*n*=7/group) bearing Me13 xenografts were treated between day 11 and day 25 (dotted line) with AZD6244, TRAIL or their combination. Statistical analysis by mixed models ANOVA; ****P<0.0001; **P<0.01. (**b**) Immunohistochemistry analysis of tumor nodules removed from control and treated mice. Insets, higher magnification of a representative area of each panel. Original magnification, × 20. NS, not significant

**Figure 7:**
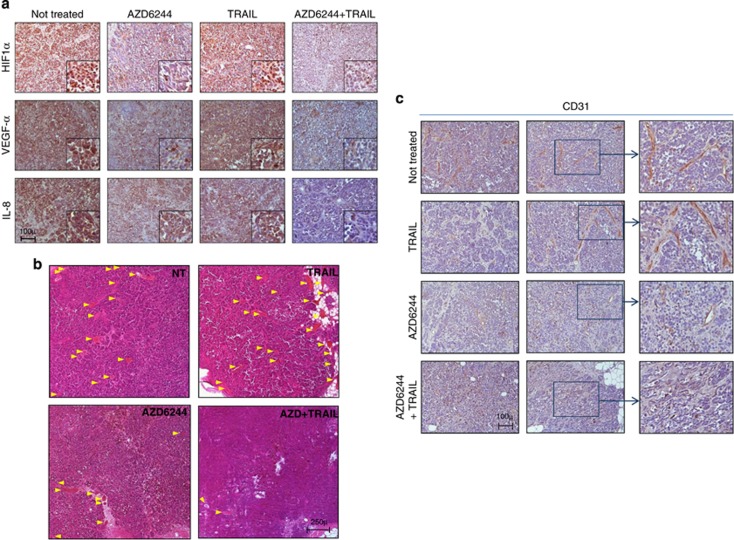
Inhibition of pro-angiogenic molecules and anti-angiogenic effects of AZD6244 plus TRAIL treatment *in vivo*. (**a**) Immunohistochemistry analysis for HIF-1*α*, VEGF*α* and IL-8 of Me13 tumor nodules removed from control and treated mice. Insets, higher magnification of a representative area of each panel. Original magnification, × 20. (**b**) Hematoxylin–eosin staining of neoplastic nodules from mice treated with AZD6244, TRAIL and their combination. Arrows: large blood vessels. (**c**) Staining of Me13 nodules for murine CD31 endothelial cell marker. Two representative fields (left and middle panel) and a higher magnification area (inset, right panel) are shown for each treatment group

Based on this preliminary *in vitro* evidence, we then selected a tumor (Me13) resistant to TRAIL and poorly responsive to AZD6244 for *in vivo* experiments. AZD6244 treatment of severe combined immunodeficiency (SCID) mice, bearing s.c. Me13 xenografts, exerted a moderate but significant tumor inhibition effect, while TRAIL had no impact on tumor growth (Figure 6a). However, the AZD6244–TRAIL combination showed a highly significant anti-tumor activity compared not only with TRAIL treatment but also with AZD6244 (Figure 6a). Histological analysis of main organs (Supplementary Figure S17a) and monitoring of mice weight (Supplementary Figure S17b) did not indicate any significant toxicity associated with these treatments. Melanoma cells from neoplastic nodules removed at the end of treatment showed enhanced positivity for terminal deoxynucleotidyl transferase-mediated dUTP nick end-labeling (TUNEL) and cleaved caspase 3, associated with downmodulation of *P*-ERK and Apollon, in animals treated with the AZD6244–TRAIL association, compared with single treatments (Figure 6b). These results were consistent with effective inhibition of the ERK pathway and with promotion of melanoma cell death. In addition, the AZD6244–TRAIL combination was more effective than AZD6244 treatment in reducing the expression *in vivo*, in neoplastic cells, of HIF1*α*, VEGF*α* and even of interleukin8, a well-known pro-angiogenic target of HIF-1*α* (Figure 7a). Most importantly, neoplastic nodules from animals treated with the AZD6244–TRAIL association showed a markedly reduced density of both large and small blood vessels, as assessed, respectively, by analysis of very wide microscopy fields on hematoxylin and eosin-stained sections (Figure 7b) and by staining for murine CD31 endothelial cell marker (Figure 7c).

Taken together, these results indicate that a combinatorial treatment approach that targets at least one relevant melanoma survival pathway (MEK–ERK) and the TRAIL signaling pathway has significant anti-tumor activity *in vivo* even against a tumor poorly responsive to a MEK inhibitor and completely resistant to TRAIL. Moreover, these results indicate that this strategy acts by both direct anti-tumor effects and by inhibition of angiogenesis.

## Discussion

The results of this study indicate that co-targeting of oncogenic and death receptor pathways in melanoma exerts synergistic anti-tumor effects through a direct induction of cell death, by promotion of caspase-dependent apoptosis and through an indirect activity on tumor vasculature. The two effective combinatorial treatments (AZD6244–BEZ235–TRAIL and AZD–TRAIL) were shown to overcome resistance to each agent, and the synergistic drug interaction effects were observed on melanoma cell lines with different genetic background, including mutations of BRAF or NRAS, as well as mutations of p53 and/or PTEN, two genes whose inactivation contributes to melanoma resistance to target therapy.^33,34^ In contrast, the third combinatorial treatment that we investigated (BEZ235–TRAIL) was characterized by marked antagonism and poor fraction affected in most instances, suggesting that inhibition of the PI3K/mTOR pathway can have synergistic anti-melanoma effects with TRAIL only when associated with targeting of the MEK–ERK pathway.

Gene expression experiments coupled to *in vitro* cell death assays indicated that the anti-tumor effects of the three- and two-drug associations were due to promotion of caspase-dependent melanoma cell death. This observation suggested that the combinatorial treatments could effectively counteract distinct apoptosis resistance mechanisms that hinder melanoma response to monotherapy based on TRAIL or target-specific inhibitors.^9,35, 36, 37^ This hypothesis was confirmed by investigation of several components of the extrinsic and intrinsic pathways of apoptosis. c-FLIP, the main caspase-8 inhibitor, is overexpressed in melanoma lesions^38^ and is a main mechanism of melanoma resistance to TRAIL, as indicated by studies where c-FLIP downregulation was sufficient to render TRAIL-R2^+^ melanoma cells responsive to this death ligand.^39^ We found that both the AZD6244–BEZ235–TRAIL and the AZD6244–TRAIL combinations induced strong downmodulation of c-FLIP_L_ and/or c-FLIP_s_ isoforms, associated with caspase-8 activation, compared with single treatments and to the AZD6244–BEZ235 treatment. Moreover, we hypothesized that rescuing the activation of caspase-8 could not be sufficient to explain the overall pro-apoptotic efficacy of the combinatorial treatments but that additional effects on the mitochondrial pathway were likely to occur. In agreement with this hypothesis, we found that co-targeting of oncogenic and death receptor pathways promoted a strong upregulation of pro-apoptotic BIM_S_ and BAX*α* isoforms, associated with downregulation of clusterin and of Mcl-1. Interestingly, BIM has been recently shown to activate BAX,^40^ while clusterin is a known inhibitor of the pro-apoptotic activity of BAX.^30^ Moreover, BIM mediates the apoptotic response to AZD6244 in diffuse large B cell lymphoma^16^ and to BEZ235 in KRAS-mutant colorectal cancer cells,^41^ while BIM_S_ upregulation has a key role in melanoma apoptosis induced by BRAFV600E inhibitors.^29^ Taken together, our results suggest that association of TRAIL with co-targeting of MEK and PI3K/mTOR, or with MEK blockade, triggers a BIM–BAX axis, thus effectively contributing to promotion of mitochondrial depolarization.

We also found that the AZD6244–BEZ235–TRAIL and AZD6244–TRAIL associations influenced the expression of several IAP family members. Overexpression of these proteins contributes to apoptosis resistance in different tumors, including melanoma,^27,42^ by inhibition of both initiator and effector caspases.^43^ Interestingly, we found that the combinatorial treatments induced a strong downmodulation of c-IAP1, c-IAP2, XIAP and Apollon, and this may contribute to explain the significant activation of caspase-3/7 that we observed. Moreover, the key role of IAP downmodulation, in the pro-apoptotic effects of the combinatorial treatments, was confirmed by silencing of Apollon, a IAP whose role in melanoma resistance to a wide range of pro-apoptotic agents we have recently demonstrated.^27^

The available evidence on the mechanisms of action, *in vivo*, of TRAIL and inhibitors as AZD6244^21,44,45^ led us to hypothesize that their association might have anti-tumor effects not only uniquely exerted through induction of apoptosis but also through inhibition of angiogenesis. Indeed, in SCID mice bearing s.c. melanoma xenografts, we found that the AZD6244–TRAIL association exerted a significant anti-tumor activity, compared with single treatments, in a TRAIL-resistant tumor. The combinatorial treatment suppressed not only several pro-angiogenic molecules and, *in vivo*, promoted melanoma apoptosis, but also significantly affected tumor angiogenesis. The inhibitory effects of AZD6244–TRAIL treatment on master regulators of angiogenesis, such as TGF*β*1, HIF1*α* and Myc, as well as on production of angiogenic molecules as VEGF*α*, were observed at a time (8–18 h of treatment) when no significant melanoma cell death was yet induced, suggesting that the observed anti-angiogenic effect was not simply explained by increased melanoma apoptosis. Moreover, inhibition of the main angiogenesis regulator VEGF*α* by AZD–TRAIL treatment was observed in several melanoma cell lines, suggesting that this is a general effect of this combinatorial treatment.

In conclusion, the results of this study provide a proof of principle for future combinatorial approaches to melanoma therapy that may overcome intrinsic resistance to single agents^46^ and exert relevant effects on the tumor microenvironment.

## Materials and Methods

### Melanoma cell lines

Melanoma cell lines were established as described^47,48^ from surgical specimens of American Joint Committee on Cancer Stage IIIb to IV melanoma patients not previously subjected to chemotherapy and admitted to Fondazione IRCCS Istituto Nazionale dei Tumori, Milan. All lesions were histologically confirmed to be cutaneous malignant melanomas. The study was conducted according to the Declaration of Helsinki Principles and following institutional guidelines. Molecular and biological features of the cell lines, including susceptibility to TRAIL and to target-specific inhibitors, are listed in Supplementary Table S1. Methods for identification of mutations in BRAF, NRAS, PTEN and p53 genes and main molecular features of the cell lines have been previously reported.^27,42,48,49^ All cell lines were maintained as described.^47^

### Treatment of melanoma cells with inhibitors or TRAIL

Cells were treated with different concentrations of AZD6244 (SelleckChem, Houston, TX, USA), BEZ235 (SelleckChem), recombinant human TRAIL (AdipoGen, San Diego, CA, USA) or with combinations of these agents. Treatments were performed in quadruplicates in 96-well flat bottom plates with 200 *μ*l RPMI 1640 (BioWhittaker, VWR International, Radnor, PA, USA) supplemented with 2% fetal calf serum without antibiotics. Cultures were evaluated at 48–72 h as described^27,42^ for cell viability, by the 3-(4,5)dimethylthiazol-2,5-diphenyltetrazolium bromide (MTT) assay. IC_50_ values for melanoma response to AZD6244 and BEZ235 were obtained through nonlinear regression analysis (by the PRISM software, Graphpad, La Jolla, CA, USA) of dose–response curves. Data from MTT assays were analyzed for drug interaction by the Chou and Talalay method.^25^ Combination indexes and FA values were obtained by the CompuSyn software (ComboSyn Inc., Paramus, NJ, USA).

### Antibodies for flow cytometry and western blotting analysis

Flow cytometry and/or western blottig analyses were carried out with antibodies specific for: cleaved caspase 8, livin, clusterin, cIAP-2, BIM, BID, BAX, Mcl-1 and c-Myc (Cell Signaling Technology Inc., Danvers, MA, USA); survivin (Novus Biological, Littleton, CO, USA); c-IAP1 (R&D Systems, Minnneapolis, MN, USA); HIF1*α* and *β*-actin (Abcam, Cambridge, UK); Apollon/BIRC6 and XIAP (BD Biosciences, Franklin Lakes, NJ, USA); cFLIP_L/S_ (Alexis Biochemicals, Enzo Life Sciences Inc., Farmingdale, NY, USA); PE-TRAIL-R1/DR4, PE-TRAIL-R2/DR5 (BioLegend, San Diego, CA, USA); and TRAIL-R3/DCR1 and TRAIL-R4/DCR2 (AdipoGen).

### Flow cytometry assays

Melanoma apoptosis was assessed by the Annexin V/PI flow cytometry assay as described.^27^ Expression of intracellular molecules was evaluated by flow cytometry after cell permeabilization with saponin or methanol, as described.^47^ Mitochondrial membrane depolarization was assessed by the fluorescent probe tetramethyl rhodamine ethyl ester (TMRE; Invitrogen, Life Technologies, Grand Island, NY, USA) as described. ^27^ Flow cytometry experiments were carried out with a Gallios flow cytometer (Beckman Coulter, Inc., Brea, CA, USA). All data were analyzed with the FlowJo software (FlowJo LLC, Ashland, OR, USA).

### Western blots

SDS-PAGE was performed using 30–60 *μ*g of protein samples on 7% NuPAGE Tris-Acetate (for Apollon) or 4–12% NuPAGE Bis-Tris polyacrylamide gels (Invitrogen, Life Technologies), as described.^27,49^ Development was performed by the chemiluminescence method with the ECL Western Blotting Detection System (GE Healthcare, Fairfield, CT, USA).

### Apoptosis and angiogenesis protein arrays

The Human Angiogenesis Array kit (R&D Systems) was used according to the manufacturer's instructions. Signals on membranes were detected by chemiluminescence and quantitated by densitometric analysis with Quantity One software (Bio-Rad Laboratories Inc., Hercules, CA, USA). Protein expression values were expressed as the percentage of the mean of the relative positive controls, after background subtraction.

### Genome-wide expression profiling of melanoma cells treated with target-specific inhibitors and TRAIL

Melanoma cells were treated with AZD6244 (0.1 *μ*M), BEZ235 (0.1 *μ*M) or TRAIL (25 ng/ml) or with their combinations for 8 h. Three biological replicates for each treatment were set up. RNA isolation and processing were performed as described.^27^ Single-color hybridization of RNAs was performed on Illumina Bead Chip HumanHT-12_v4 Microarrays (Illumina, San Diego, CA, USA) containing >48 000 transcript probes. The expression profiles have been deposited in NCBI's Gene Expression Omnibus (GEO) with GSE accession number GSE55050. Background correction, filtering of data and quantile normalization were done using the BeadStudio Illumina software. Identification of significantly modulated genes (by BRB array tools vers. 4.3.0), generation of Edwards-VENN diagrams (by the VENNTURE software^26^) and downstream effects analysis and upstream regulator analysis (by Ingenuity Pathway Analysis, IPA 8.5, www.ingenuity.com) were carried out as described in Supplementary Methods.

### Enzymatic activity of caspases and treatment of melanoma cells with caspase inhibitors

Caspase 3/7 activation was evaluated by the MUSE caspase 3/7 kit, specific for the MUSE cell analyzer (Merck Millipore, Billerica, MA, USA). Where mentioned, melanoma cells were preincubated with general caspase inhibitor z-VAD-fmk or control z-FA-fmk (BD Pharmingen, Franklin Lakes, NJ, USA) at 5 *μ*M for 1 h at 37 °C before treatment with drugs. Apoptosis was then assessed by the annexin-V/PI flow cytometry assay.

### Silencing of Apollon by small interfering RNA (siRNA)

Cells were transfected with one of the two previously validated^27^ Apollon-specific siRNA (Stealth RNAi siRNA, sequence 5′-GGGCAUGCUGGAAUGUUGACGUUAA-3′, Invitrogen) and corresponding negative control siRNAs according to Lipofectamine RNAiMAX guidelines (Invitrogen) to reach a final siRNA concentration of 75 nmol/l.

### ELISA

The Quantikine ELISA kit (R&D Systems) for TGF*β*1 and VEGF*α* were used according to the manufacturer's instructions. The optical density of each plate was determined using a microplate reader (Infinite M1000, Tecan Group Ltd., Männedorf, Switzerland).

### *In vivo* treatments

Animal experiments were performed according to the Italian laws (D.L. 116/92 and after additions) and were approved by the institutional Ethical Committee for Animal Experimentation of our Institute and by the Italian Ministry of Health (Project INT_17/2011). Female SCID mice 8–10-weeks old (Charles River Laboratories, Wilmington, MA, USA) were provided with food and water *ad libitum*. Melanoma cells (Me13) were harvested in exponential growth phase and were injected subcutaneously (5 × 10^6^) in the left flank of each mouse. When tumors became palpable, mice were randomized into four groups (7 animals/group). Animals received either vehicle, 25 mg/kg AZD6244 (oral gavage), 30 mg/kg TRAIL (i.p. injection) or a combination of the two drugs, 7 days per week for 2 consecutive weeks. Mice were monitored daily for signs of toxicity and were weighed twice weekly. Tumor size was regularly evaluated by measuring the orthogonal diameters (*d* and *D)* and calculating the volumes with the following formula:


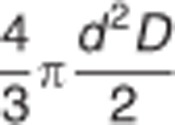


### Immunohistochemistry

Immunohistochemistry was performed on formalin-fixed, paraffin-embedded tissues as described.^27,42,47^ Tissue sections from melanoma metastases were characterized by staining for TRAIL-R2/DR5 (Sigma-Aldrich, St. Louis, MO, USA). Neoplastic nodules removed from SCID mice at the end of treatment were characterized by staining with mAbs to human pERK, cleaved caspase-3 (Cell Signaling Technology Inc.), Apollon, HIF1*α*, IL8 (Abcam), VEGF*α* (Santa Cruz Biotechnology Inc., Dallas, TX, USA), as well as to mouse CD31 (Dianova GmbH, Hamburg, Germany). The extent of apoptosis in neoplastic nodules was evaluated by TUNEL staining (Roche). Images were acquired at × 20 with an Axiovert 100 microscope (Zeiss, Oberkochen, Germany) equipped with a digital camera (AxioCam MrC5, Zeiss).

### Statistical analysis

Data from TRAIL susceptibility, TMRE and caspase-8 cleavage assays, as well as the results of drug interaction analyses (combination index values) were clustered by the Cluster 3.0 software (Tokyo, Japan). Significance of different treatments on melanoma apoptosis, caspase activation, mitochondrial depolarization and modulation of apoptosis- and angiogenesis-related molecules was assessed by ANOVA, followed by the SNK multiple comparison test. Correlation of melanoma responsiveness to TRAIL with susceptibility to target-specific inhibitors or with expression of TRAIL receptors were assessed by Spearman's correlation analysis. In xenograft experiments, comparison of the anti-tumor activity of different treatments was carried out by mixed effects model ANOVA^50^ by the XLSTAT software (Xlstat, Addinsoft's, New York, NY, USA).

## Abbreviations

TRAIL: tumor necrosis factor-related apoptosis-inducing ligand
MEK: mitogen/extracellular signal-regulated kinase
PI3K: phosphoinositide 3-kinase
mTOR: mammalian target of rapamycin
BRAF: v-raf murine sarcoma viral oncogene homolog B
NRAS: neuroblastoma RAS viral (v-ras) oncogene homolog
PTEN: phosphatase and tensin homolog
c-FLIP: c-FLICE-like inhibitory protein
BIM: bcl-2-like protein 11 isoform 1
BAX: BCL2-associated X protein
Mcl-1: myeloid cell leukemia 1
IAP: inhibitor of apoptosis protein
SCID: severe combined immunodeficiency
HIF1*α*: hypoxia inducible factor 1 alpha subunit
VEGF*α*: vascular endothelial growth factor alpha
TGF*β*1: transforming growth factor *β*1
DR5: death receptor 5
BID: BH3 interacting domain death agonist
XIAP: X-linked inhibitor of apoptosis
Myc: v-myc avian myelocytomatosis viral oncogene homolog
ERK: extracellular signal-regulated kinase
TUNEL: terminal deoxynucleotidyl transferase-mediated dUTP nick end labeling
ANOVA: analysis of variance

## References

[bib1] 1Chapman PB, Hauschild A, Robert C, Haanen JB, Ascierto P, Larkin J et al. Improved survival with vemurafenib in melanoma with BRAF V600E mutation. N Engl J Med 2011; 364: 2507–2516. 21639808 10.1056/NEJMoa1103782 PMC3549296

[bib2] 2Flaherty KT, Robert C, Hersey P, Nathan P, Garbe C, Milhem M et al. Improved survival with MEK inhibition in BRAF-mutated melanoma. N Engl J Med 2012; 367: 107–114. 22663011 10.1056/NEJMoa1203421

[bib3] 3Hauschild A, Grob JJ, Demidov LV, Jouary T, Gutzmer R, Millward M et al. Dabrafenib in BRAF-mutated metastatic melanoma: a multicentre, open-label, phase 3 randomised controlled trial. Lancet 2012; 380: 358–365. 22735384 10.1016/S0140-6736(12)60868-X

[bib4] 4Hodi FS, O'Day SJ, McDermott DF, Weber RW, Sosman JA, Haanen JB et al. Improved survival with ipilimumab in patients with metastatic melanoma. N Engl J Med 2010; 363: 711–723. 20525992 10.1056/NEJMoa1003466 PMC3549297

[bib5] 5Topalian SL, Hodi FS, Brahmer JR, Gettinger SN, Smith DC, McDermott DF et al. Safety, activity, and immune correlates of anti-PD-1 antibody in cancer. N Engl J Med 2012; 366: 2443–2454. 22658127 10.1056/NEJMoa1200690 PMC3544539

[bib6] 6Hartsough E, Shao Y, Aplin AE. Resistance to RAF inhibitors revisited. J Invest Dermatol 2014; 134: 319–325. 24108405 10.1038/jid.2013.358 PMC3947111

[bib7] 7Leone P, Shin EC, Perosa F, Vacca A, Dammacco F, Racanelli V. MHC class I antigen processing and presenting machinery: organization, function, and defects in tumor cells. J Natl Cancer Inst 2013; 105: 1172–1187. 23852952 10.1093/jnci/djt184

[bib8] 8Greger JG, Eastman SD, Zhang V, Bleam MR, Hughes AM, Smitheman KN et al. Combinations of BRAF, MEK, and PI3K/mTOR inhibitors overcome acquired resistance to the BRAF inhibitor GSK2118436 dabrafenib, mediated by NRAS or MEK mutations. Mol Cancer Ther 2012; 11: 909–920. 22389471 10.1158/1535-7163.MCT-11-0989

[bib9] 9Kwong LN, Davies MA. Targeted therapy for melanoma: rational combinatorial approaches. Oncogene 2014; 33: 1–9. 23416974 10.1038/onc.2013.34

[bib10] 10Roberts PJ, Usary JE, Darr DB, Dillon PM, Pfefferle AD, Whittle MC et al. Combined PI3K/mTOR and MEK inhibition provides broad antitumor activity in faithful murine cancer models. Clin Cancer Res 2012; 18: 5290–5303. 22872574 10.1158/1078-0432.CCR-12-0563 PMC3715399

[bib11] 11Beck D, Niessner H, Smalley KS, Flaherty K, Paraiso KH, Busch C et al. Vemurafenib potently induces endoplasmic reticulum stress-mediated apoptosis in BRAFV600E melanoma cells. Sci Signal 2013; 6: ra7. 23362240 10.1126/scisignal.2003057 PMC3698985

[bib12] 12Ma XH, Piao SF, Dey S, McAfee Q, Karakousis G, Villanueva J et al. Targeting ER stress-induced autophagy overcomes BRAF inhibitor resistance in melanoma. J Clin Invest 2014; 124: 1406–1417. 24569374 10.1172/JCI70454 PMC3934165

[bib13] 13Berger A, Quast SA, Plotz M, Kuhn NF, Trefzer U, Eberle J. RAF inhibition overcomes resistance to TRAIL-induced apoptosis in melanoma cells. J Invest Dermatol 2014; 134: 430–440. 23955071 10.1038/jid.2013.347

[bib14] 14Quast SA, Berger A, Eberle J. ROS-dependent phosphorylation of Bax by wortmannin sensitizes melanoma cells for TRAIL-induced apoptosis. Cell Death Dis 2013; 4: e839. 24113173 10.1038/cddis.2013.344 PMC3824654

[bib15] 15Zhang XD, Borrow JM, Zhang XY, Nguyen T, Hersey P. Activation of ERK1/2 protects melanoma cells from TRAIL-induced apoptosis by inhibiting Smac/DIABLO release from mitochondria. Oncogene 2003; 22: 2869–2881. 12771938 10.1038/sj.onc.1206427

[bib16] 16Bhalla S, Evens AM, Dai B, Prachand S, Gordon LI, Gartenhaus RB. The novel anti-MEK small molecule AZD6244 induces BIM-dependent and AKT-independent apoptosis in diffuse large B-cell lymphoma. Blood 2011; 118: 1052–1061. 21628402 10.1182/blood-2011-03-340109 PMC3148157

[bib17] 17Rahmani M, Aust MM, Attkisson E, Williams Jr DC, Ferreira-Gonzalez A, Grant S. Dual inhibition of Bcl-2 and Bcl-xL strikingly enhances PI3K inhibition-induced apoptosis in human myeloid leukemia cells through a GSK3- and Bim-dependent mechanism. Cancer Res 2013; 73: 1340–1351. 23243017 10.1158/0008-5472.CAN-12-1365 PMC3578060

[bib18] 18Zang C, Eucker J, Liu H, Muller A, Possinger K, Scholz CW. Concurrent inhibition of PI3-kinase and mTOR induces cell death in diffuse large B cell lymphomas, a mechanism involving down regulation of Mcl-1. Cancer Lett 2013; 339: 288–297. 23200668 10.1016/j.canlet.2012.11.013

[bib19] 19Gunda V, Bucur O, Varnau J, Vanden Borre P, Bernasconi MJ, Khosravi-Far R et al. Blocks to thyroid cancer cell apoptosis can be overcome by inhibition of the MAPK and PI3K/AKT pathways. Cell Death Dis 2014; 5: e1104. 24603332 10.1038/cddis.2014.78 PMC3973207

[bib20] 20Geserick P, Herlyn M, Leverkus M. On the TRAIL to overcome BRAF-inhibitor resistance. J Invest Dermatol 2014; 134: 315–318. 24424456 10.1038/jid.2013.348

[bib21] 21Wilson NS, Yang A, Yang B, Couto S, Stern H, Gogineni A et al. Proapoptotic activation of death receptor 5 on tumor endothelial cells disrupts the vasculature and reduces tumor growth. Cancer Cell 2012; 22: 80–90. 22789540 10.1016/j.ccr.2012.05.014

[bib22] 22Allen JE, Krigsfeld G, Mayes PA, Patel L, Dicker DT, Patel AS et al. Dual inactivation of Akt and ERK by TIC10 signals Foxo3a nuclear translocation, TRAIL gene induction, and potent antitumor effects. Sci Transl Med 2013; 5: 171ra117. 10.1126/scitranslmed.3004828 PMC4535715 23390247

[bib23] 23Robert C, Dummer R, Gutzmer R, Lorigan P, Kim KB, Nyakas M et al. Selumetinib plus dacarbazine versus placebo plus dacarbazine as first-line treatment for BRAF-mutant metastatic melanoma: a phase 2 double-blind randomised study. Lancet Oncol 2013; 14: 733–740. 23735514 10.1016/S1470-2045(13)70237-7

[bib24] 24Thomas WD, Zhang XD, Franco AV, Nguyen T, Hersey P. TNF-related apoptosis-inducing ligand-induced apoptosis of melanoma is associated with changes in mitochondrial membrane potential and perinuclear clustering of mitochondria. J Immunol 2000; 165: 5612–5620. 11067917 10.4049/jimmunol.165.10.5612

[bib25] 25Chou TC. Theoretical basis, experimental design, and computerized simulation of synergism and antagonism in drug combination studies. Pharmacol Rev 2006; 58: 621–681. 16968952 10.1124/pr.58.3.10

[bib26] 26Martin B, Chadwick W, Yi T, Park SS, Lu D, Ni B et al. VENNTURE–a novel Venn diagram investigational tool for multiple pharmacological dataset analysis. PLoS One 2012; 7: e36911. 22606307 10.1371/journal.pone.0036911 PMC3351456

[bib27] 27Tassi E, Zanon M, Vegetti C, Molla A, Bersani I, Perotti V et al. Role of Apollon in human melanoma resistance to antitumor agents that activate the intrinsic or the extrinsic apoptosis pathways. Clin Cancer Res 2012; 18: 3316–3327. 22553342 10.1158/1078-0432.CCR-11-2232 PMC3426233

[bib28] 28Cartron PF, Priault M, Oliver L, Meflah K, Manon S, Vallette FM. The N-terminal end of Bax contains a mitochondrial-targeting signal. J Biol Chem 2003; 278: 11633–11641. 12529375 10.1074/jbc.M208955200

[bib29] 29Jiang CC, Lai F, Tay KH, Croft A, Rizos H, Becker TM et al. Apoptosis of human melanoma cells induced by inhibition of B-RAFV600E involves preferential splicing of bimS. Cell Death Dis 2010; 1: e69. 21364673 10.1038/cddis.2010.48 PMC3032346

[bib30] 30Zhang H, Kim JK, Edwards CA, Xu Z, Taichman R, Wang CY. Clusterin inhibits apoptosis by interacting with activated Bax. Nat Cell Biol 2005; 7: 909–915. 16113678 10.1038/ncb1291

[bib31] 31Lindsay J, Esposti MD, Gilmore AP. Bcl-2 proteins and mitochondria–specificity in membrane targeting for death. Biochim Biophys Acta 2011; 1813: 532–539. 21056595 10.1016/j.bbamcr.2010.10.017

[bib32] 32Haq R, Shoag J, Andreu-Perez P, Yokoyama S, Edelman H, Rowe GC et al. Oncogenic BRAF regulates oxidative metabolism via PGC1alpha and MITF. Cancer Cell 2013; 23: 302–315. 23477830 10.1016/j.ccr.2013.02.003 PMC3635826

[bib33] 33Ji Z, Kumar R, Taylor M, Rajadurai A, Marzuka-Alcala A, Chen YE et al. Vemurafenib synergizes with nutlin-3 to deplete survivin and suppresses melanoma viability and tumor growth. Clin Cancer Res 2013; 19: 4383–4391. 23812671 10.1158/1078-0432.CCR-13-0074 PMC3777641

[bib34] 34Paraiso KH, Xiang Y, Rebecca VW, Abel EV, Chen YA, Munko AC et al. PTEN loss confers BRAF inhibitor resistance to melanoma cells through the suppression of BIM expression. Cancer Res 71: 2750–2760. 10.1158/0008-5472.CAN-10-2954 PMC3070772 21317224

[bib35] 35Chawla-Sarkar M, Bae SI, Reu FJ, Jacobs BS, Lindner DJ, Borden EC. Downregulation of Bcl-2, FLIP or IAPs (XIAP and survivin) by siRNAs sensitizes resistant melanoma cells to Apo2L/TRAIL-induced apoptosis. Cell Death Differ 2004; 11: 915–923. 15118763 10.1038/sj.cdd.4401416

[bib36] 36Gonzalvez F, Ashkenazi A. New insights into apoptosis signaling by Apo2L/TRAIL. Oncogene 2010; 29: 4752–4765. 20531300 10.1038/onc.2010.221

[bib37] 37Werzowa J, Koehrer S, Strommer S, Cejka D, Fuereder T, Zebedin E et al. Vertical inhibition of the mTORC1/mTORC2/PI3K pathway shows synergistic effects against melanoma *in vitro* and *in vivo*. J Invest Dermatol 2011; 131: 495–503. 21048785 10.1038/jid.2010.327

[bib38] 38Bullani RR, Huard B, Viard-Leveugle I, Byers HR, Irmler M, Saurat JH et al. Selective expression of FLIP in malignant melanocytic skin lesions. J Invest Dermatol 2001; 117: 360–364. 11511316 10.1046/j.0022-202x.2001.01418.x

[bib39] 39Geserick P, Drewniok C, Hupe M, Haas TL, Diessenbacher P, Sprick MR et al. Suppression of cFLIP is sufficient to sensitize human melanoma cells to TRAIL- and CD95L-mediated apoptosis. Oncogene 2008; 27: 3211–3220. 18084329 10.1038/sj.onc.1210985

[bib40] 40Sarosiek KA, Chi X, Bachman JA, Sims JJ, Montero J, Patel L et al. BID preferentially activates BAK while BIM preferentially activates BAX, affecting chemotherapy response. Mol Cell 2013; 51: 751–765. 24074954 10.1016/j.molcel.2013.08.048 PMC4164233

[bib41] 41Kim A, Lee JE, Lee SS, Kim C, Lee SJ, Jang WS et al. Coexistent mutations of KRAS and PIK3CA affect the efficacy of NVP-BEZ235, a dual PI3K/MTOR inhibitor, in regulating the PI3K/MTOR pathway in colorectal cancer. Int J Cancer 2013; 133: 984–996. 23475782 10.1002/ijc.28073

[bib42] 42Perotti V, Baldassari P, Bersani I, Molla A, Vegetti C, Tassi E et al. NFATc2 is a potential therapeutic target in human melanoma. J Invest Dermatol 2012; 132: 2652–2660. 22718120 10.1038/jid.2012.179

[bib43] 43Fulda S, Vucic D. Targeting IAP proteins for therapeutic intervention in cancer. Nat Rev Drug Discov 2012; 11: 109–124. 22293567 10.1038/nrd3627

[bib44] 44Na HJ, Hwang JY, Lee KS, Choi YK, Choe J, Kim JY et al. TRAIL negatively regulates VEGF-induced angiogenesis via caspase-8-mediated enzymatic and non-enzymatic functions. Angiogenesis 2014; 17: 179–194. 24097299 10.1007/s10456-013-9387-0

[bib45] 45Takahashi O, Komaki R, Smith PD, Jurgensmeier JM, Ryan A, Bekele BN et al. Combined MEK and VEGFR inhibition in orthotopic human lung cancer models results in enhanced inhibition of tumor angiogenesis, growth, and metastasis. Clin Cancer Res 2012; 18: 1641–1654. 22275507 10.1158/1078-0432.CCR-11-2324 PMC3306446

[bib46] 46Lito P, Rosen N, Solit DB. Tumor adaptation and resistance to RAF inhibitors. Nat Med 2013; 19: 1401–1409. 24202393 10.1038/nm.3392

[bib47] 47Anichini A, Mortarini R, Nonaka D, Molla A, Vegetti C, Montaldi E et al. Association of antigen-processing machinery and HLA antigen phenotype of melanoma cells with survival in American Joint Committee on Cancer stage III and IV melanoma patients. Cancer Res 2006; 66: 6405–6411. 16778219 10.1158/0008-5472.CAN-06-0854

[bib48] 48Daniotti M, Oggionni M, Ranzani T, Vallacchi V, Campi V, Di Stasi D et al. BRAF alterations are associated with complex mutational profiles in malignant melanoma. Oncogene 2004; 23: 5968–5977. 15195137 10.1038/sj.onc.1207780

[bib49] 49Sensi M, Nicolini G, Petti C, Bersani I, Lozupone F, Molla A et al. Mutually exclusive NRASQ61R and BRAFV600E mutations at the single-cell level in the same human melanoma. Oncogene 2006; 25: 3357–3364. 16462768 10.1038/sj.onc.1209379

[bib50] 50Liu C, Cripe TP, Kim MO. Statistical issues in longitudinal data analysis for treatment efficacy studies in the biomedical sciences. Mol Ther 2010; 18: 1724–1730. 20588256 10.1038/mt.2010.127 PMC2956920

