# Supplementary material for: Synergistic anti-tumor activity and inhibition of angiogenesis by cotargeting of oncogenic and death receptor pathways in human melanoma
Source: Cell Death Dis. 2014 Oct 2;5(10):e1434–. doi: 10.1038/cddis.2014.410 (PMC4649516; doi:10.1038/cddis.2014.410)
Supplement: Supplementary Figure S1 and S2 [file cddis2014410x1.pdf]

Figure S1

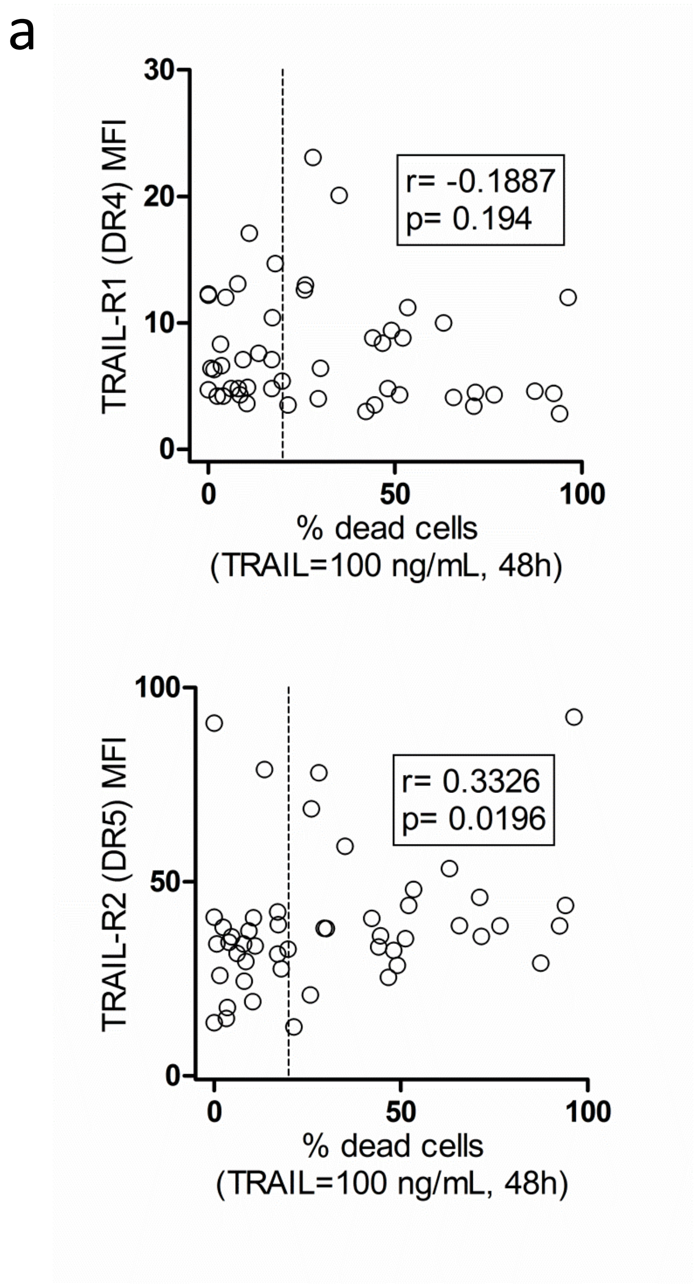
a
TRAIL-R1 (DR4) MFI
30
20
10
0
r= -0.1887
p= 0.194
0
50
100
% dead cells
(TRAIL=100 ng/mL, 48h)
TRAIL-R2 (DR5) MFI
100
50
0
r= 0.3326
p= 0.0196
0
50
100
% dead cells
(TRAIL=100 ng/mL, 48h)

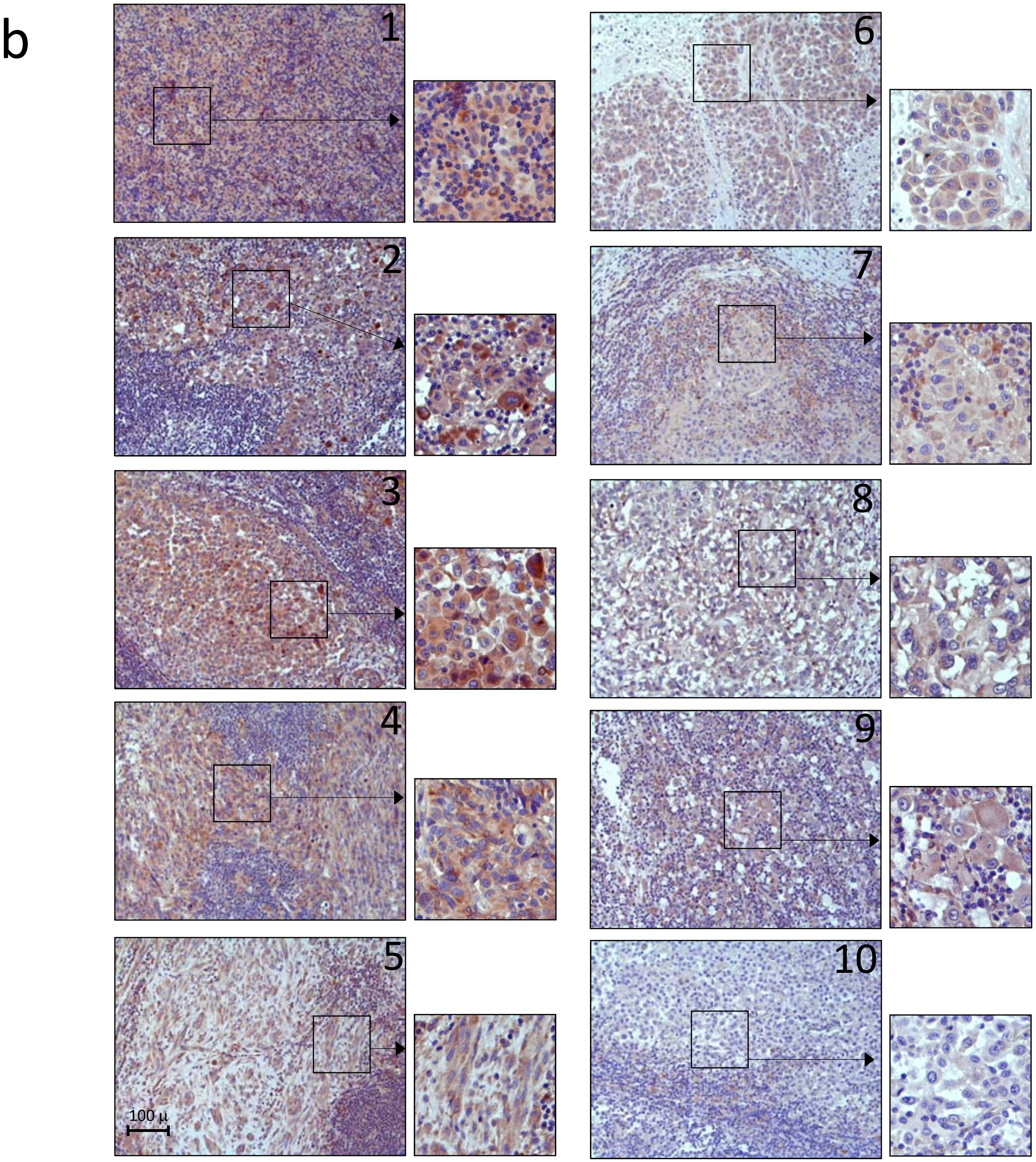
b
1
2
3
4
5
100 µ
6
7
8
9
10

Figure S1. Correlation of TRAIL susceptibility with TRAIL-R2 (DR5) expression and TRAIL-R2 expression in vivo in metastatic melanoma lesions. (a), susceptibility to TRAIL in a panel of 47 melanoma cell lines was tested by a 48 h MTT assay at TRAIL=100 ng/mL; expression of TRAIL-R1 and -R2 was assessed by flow cytometry. TRAIL-R expression indicated as mean fluorescence intensity (MFI) after subtraction of MFI of cells stained with secondary ab only. Statistical analysis by Spearman correlation. Dotted vertical lines at x=20% dead cells distinguish TRAIL- susceptible and TRAIL-resistant melanoma cell lines. (**b),** Tissue sections from lymph node (lesions #1-3 and #5-10) or subcutaneous (lesion #4) melanoma metastases from 10 patients were stained with anti-DR5 mAb. Lesion #5 is a lymphnode metastasis from a desmoplastic melanoma. Lesions are ranked from #1 to #10 according to their staining intensity for TRAIL-R2/DR5.

Figure S2

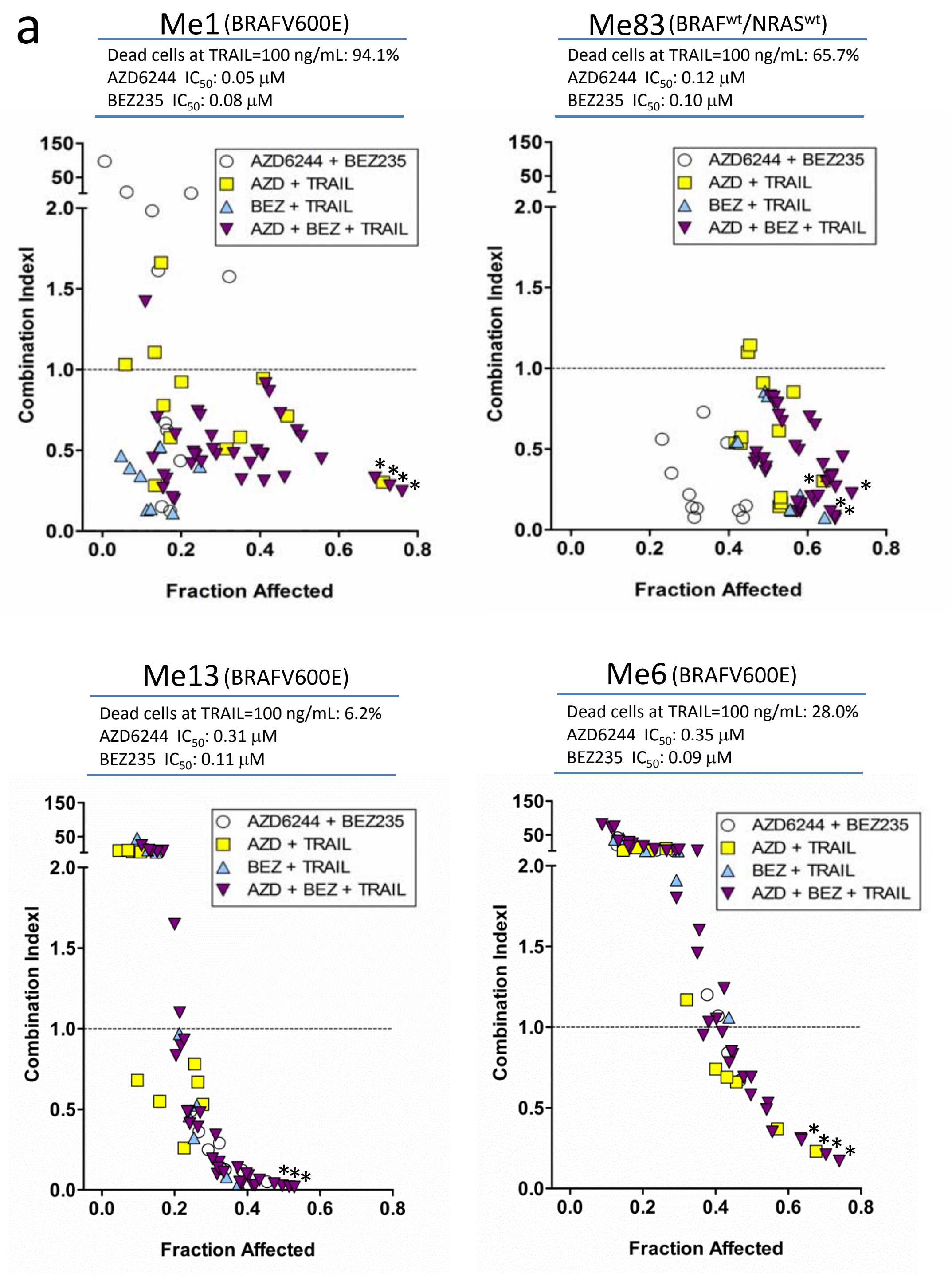

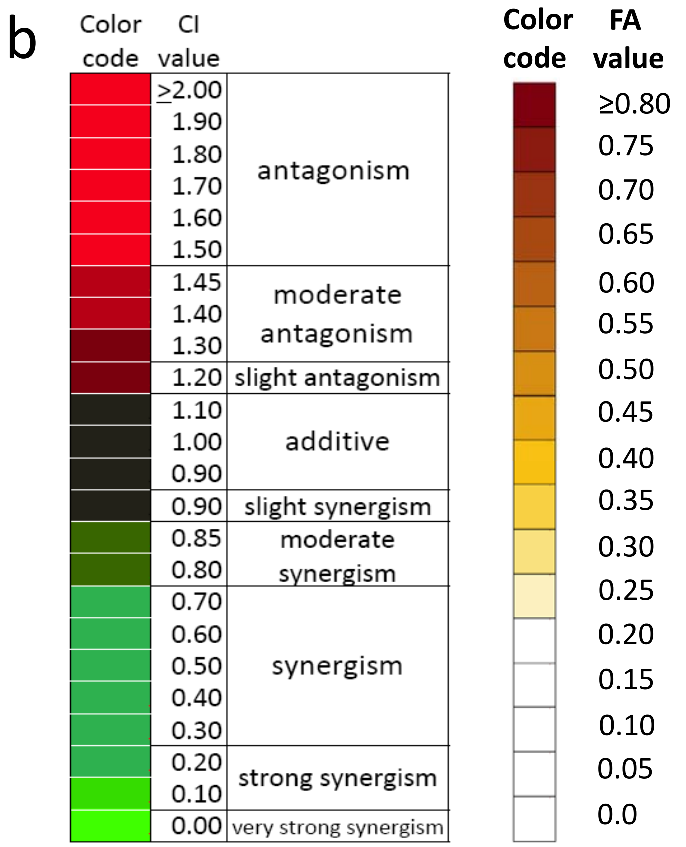

Figure S2. Drug interaction analysis by Chou-Talaly method in melanoma cell lines treated with four different combinations of AZD6244, BEZ235 and TRAIL. (a), fraction affected (Fa) vs combination index (CI) plots in four melanoma cell lines (Me1, Me83, Me13, Me6) as assessed by a 48 h MTT assay followed by data analysis by Compusyn software. Cells were treated with combinations of AZD6244 (at 0.001, 0.005, 0.01, and 0.05 μM), BEZ235 (at 0.005, 0.01, and 0.02 μM) and TRAIL (at 5, 10 and 25 ng/mL). Note that data points for CI values >150 are not shown. *These CI vs. Fa data points correspond to combinations where [AZD6244] was 0.05 μM. **(b)**, color codes used for visualization of CI and FA values in **Figure 2** and **Figure S4**. For each of the indicated CI values, or range of values, a corresponding meaning (antagonism, addittivity, synergy) is shown according to ref. 25.
