# Supplementary material for: Synergistic anti-tumor activity and inhibition of angiogenesis by cotargeting of oncogenic and death receptor pathways in human melanoma
Source: Cell Death Dis. 2014 Oct 2;5(10):e1434–. doi: 10.1038/cddis.2014.410 (PMC4649516; doi:10.1038/cddis.2014.410)
Supplement: Supplementary Figure S3 and S4 [file cddis2014410x2.pdf]

Figure S3

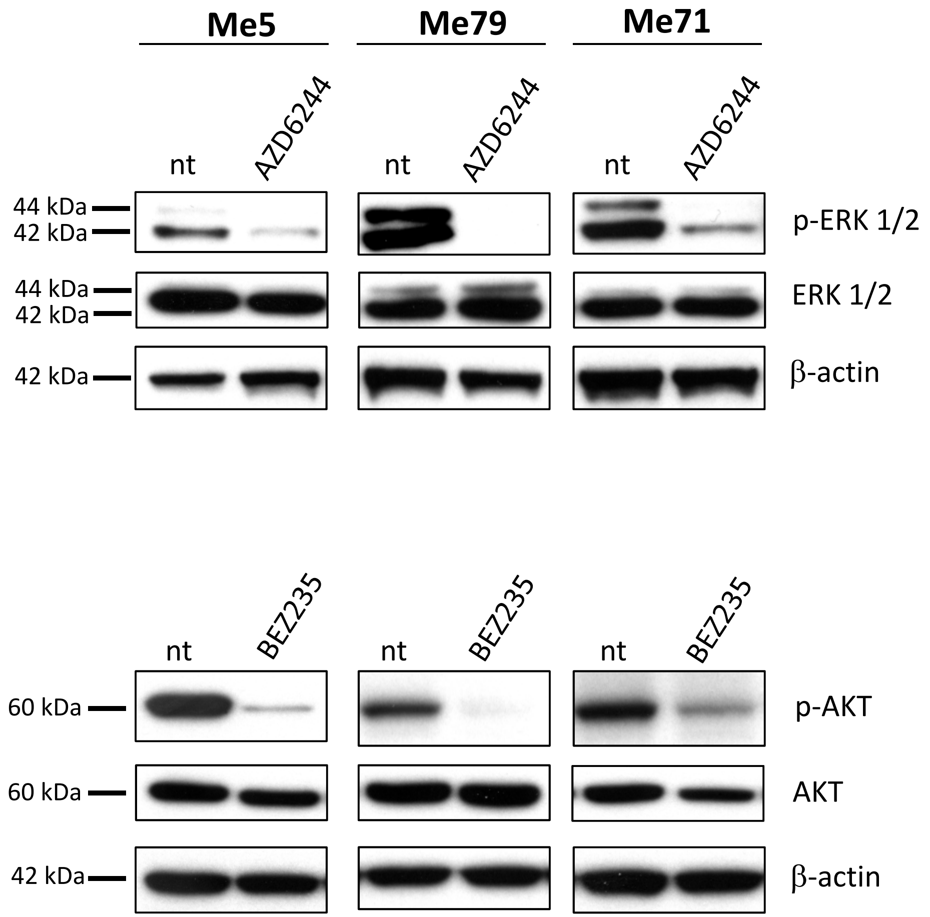

Figure S3. **Inhibition of ERK phosphorylation by AZD6244 and of AKT phosphorylation by BEZ235**. Melanoma cells from three different cell lines were cultured for 18 hr in the presence of AZD6244 (0.05 μM) or of BEZ235 (0.02 μM). Antibodies against p-ERK 1/2 and p-AKT (s473) as well as against total ERK1/2 and AKT were used.

Figure S4

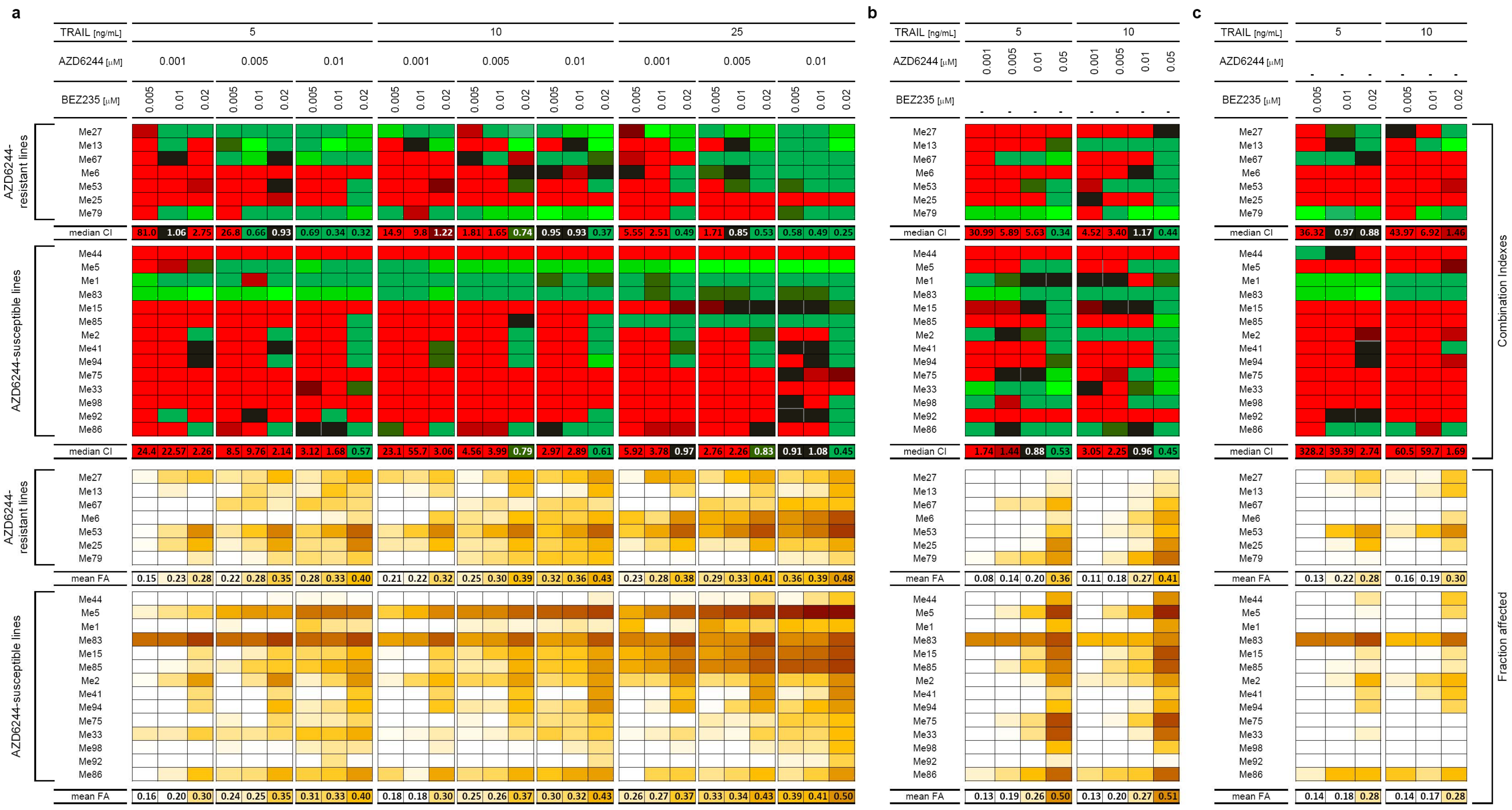

% dead cells at TRAIL=100 ng/mL	AZD6244 IC_{50} (μM)	BEZ235 IC_{50} (μM)	BRAF	NRAS	PTEN	p53
17.0	0.19	0.18	m	wt	wt/-	wt
6.2	0.31	0.11	m	wt	wt/+	wt
92.5	9.77	0.38	wt	m	wt/+	m^h
28.0	0.35	0.09	m	wt	wt/+	wt
44.0	0.29	0.01	m	wt	wt/+	wt
10.4	0.33	0.11	m	wt	wt/+	m
17.0	1.23	96.8	m	wt	wt/+	wt

4.7	0.05	0.22	m	wt	wt/+	wt
26.0	0.02	0.07	m	wt	m/-	wt
94.1	0.05	0.08	m	wt	wt/+	wt
65.7	0.12	0.10	wt	wt	m^h/+	wt
96.4	0.02	0.05	m	wt	wt/+	wt
49.0	0.01	0.53	m	wt	wt/+	wt
76.5	0.12	0.23	m	wt	wt/+	wt
35.0	0.02	0.05	m	wt	wt/+	wt
51.2	0.02	0.05	m	wt	wt/+	m
0.0	0.05	0.22	m	wt	wt/+	wt
0.0	0.02	0.28	m	wt	wt/+	wt
0.0	0.04	0.05	m	wt	m^d/+	wt
0.7	0.07	0.11	m	wt	wt/+	m
48.1	0.02	0.20	wt	m	wt/+	wt

Figure S4. Drug interaction analysis by Chou and Talalay method in two groups of melanoma cell lines with different responsiveness to AZD6244 (resistant lines: IC_{50} ≥0.2 μM, n=7; susceptible lines: IC_{50} ≤0.2 μM, n=14) treated with the association of AZD6244, BEZ235 and TRAIL (**a**), or AZD6244 and TRAIL (**b**), or BEZ235 and TRAIL (**c**). Combination indexes (CI, upper panel), and fraction affected (FA, lower panel) by a color code as in Figure 2. Susceptibility to TRAIL, AZD6244 and BEZ235 and main molecular features of all cell lines summarized in the right hand side panel (m: mutant; wt: wild type; +, -: expression/lack of expression of PTEN by western blot). Numbers at the bottom of the upper and lower panels: median CI values and mean FA values, respectively, for each combination of drugs and TRAIL doses.
