# Supplementary material for: Synergistic anti-tumor activity and inhibition of angiogenesis by cotargeting of oncogenic and death receptor pathways in human melanoma
Source: Cell Death Dis. 2014 Oct 2;5(10):e1434–. doi: 10.1038/cddis.2014.410 (PMC4649516; doi:10.1038/cddis.2014.410)
Supplement: Supplementary Figure S5 and S6 [file cddis2014410x3.pdf]

Figure S5

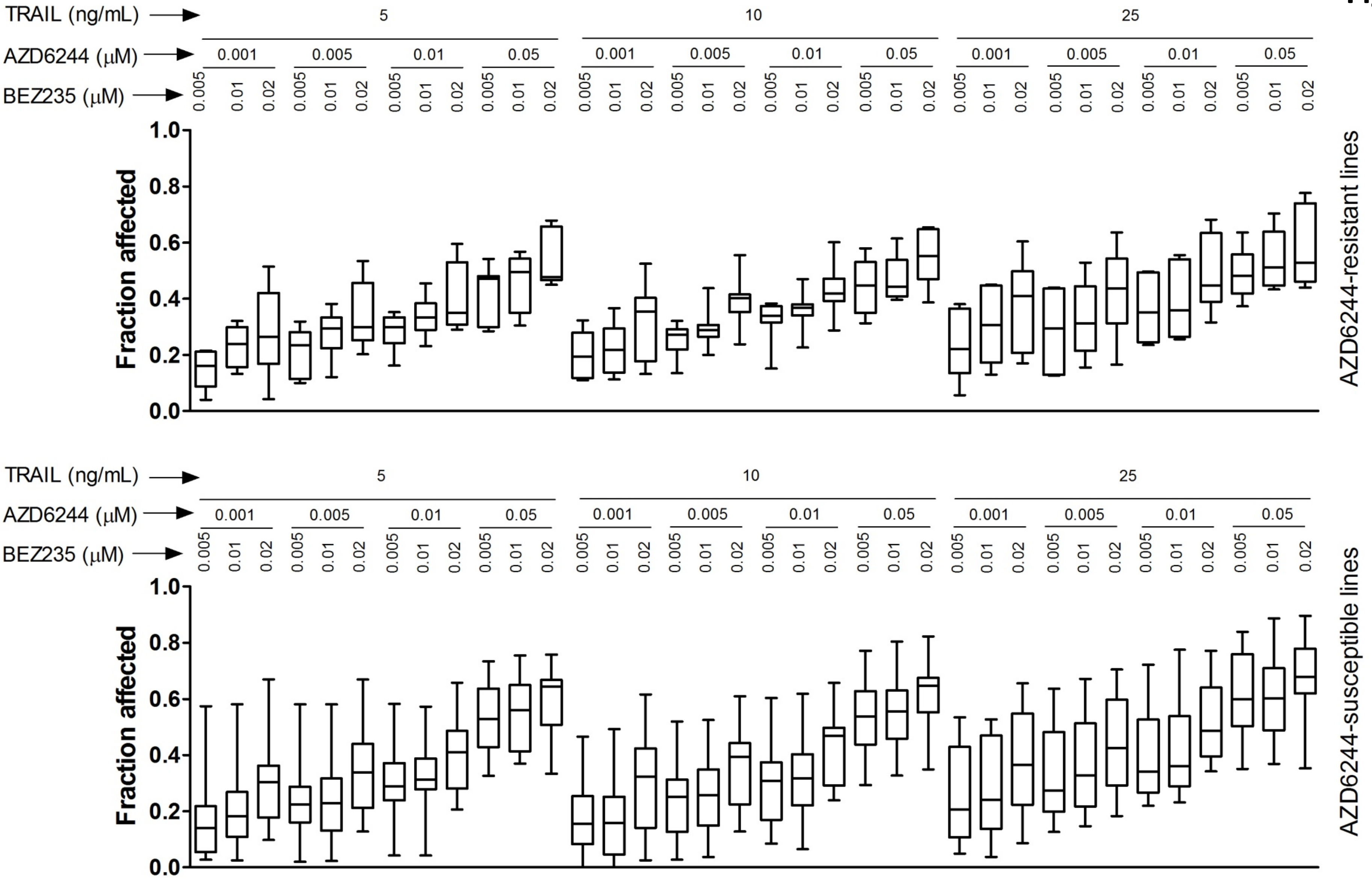

Figure S5. Box and whiskers plots of FA (Fraction affected) values by combinatorial treatment of AZD6244-resistant (n=7, upper graph) and AZD6244-susceptible (n=14, lower graph) melanoma cell lines with the association of AZD6244, BEZ235 and TRAIL.

Figure S6

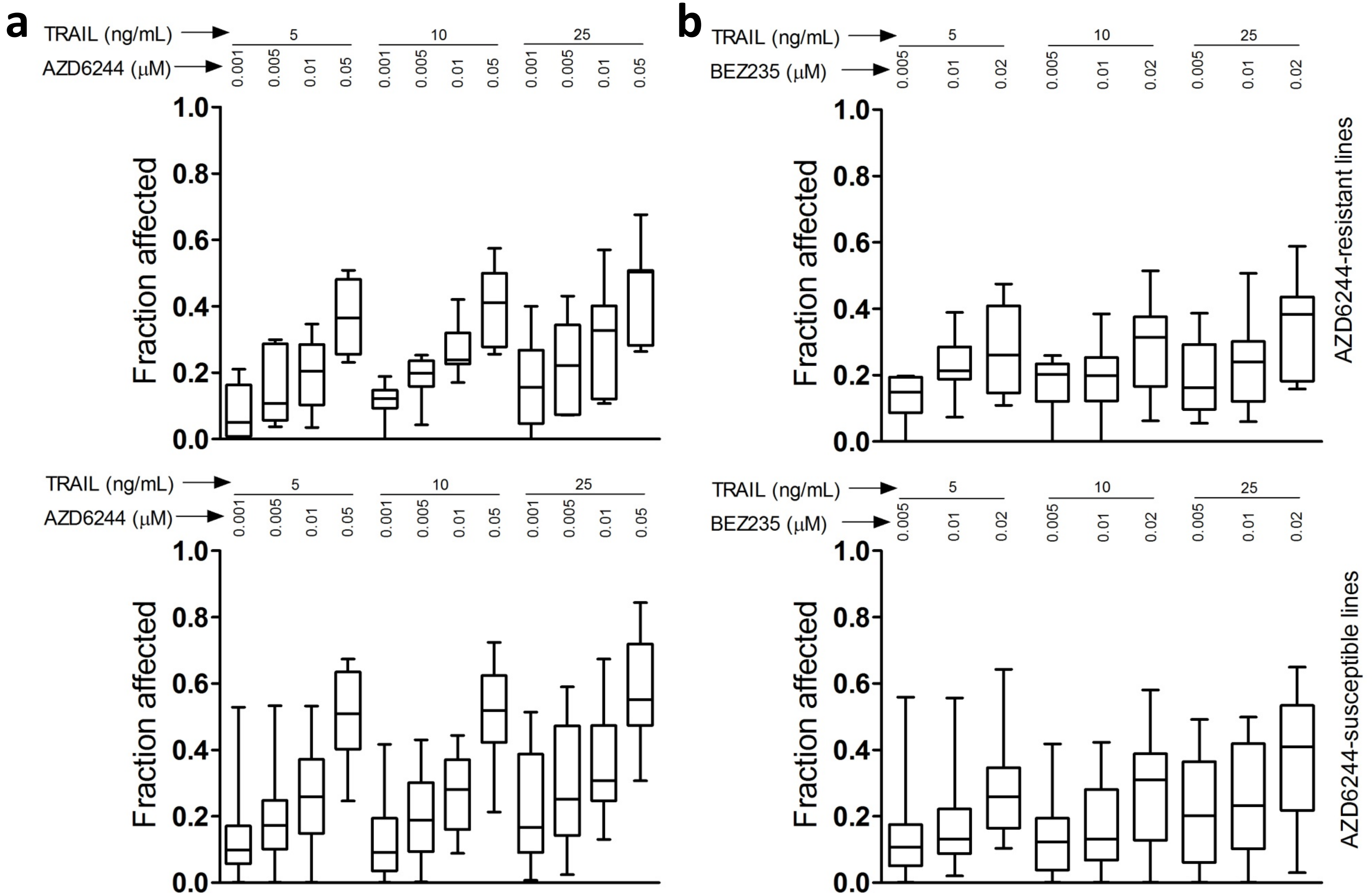

Figure S6. Box and whiskers plots of FA (Fraction affected) values by combinatorial treatment of AZD6244-resistant (n=7, upper graphs) and AZD6244-susceptible (n=14, lower graphs) melanoma cell lines with the association of AZD6244 and TRAIL (**a**) or of BEZ235 and TRAIL (**b**).
