# Supplementary material for: Synergistic anti-tumor activity and inhibition of angiogenesis by cotargeting of oncogenic and death receptor pathways in human melanoma
Source: Cell Death Dis. 2014 Oct 2;5(10):e1434–. doi: 10.1038/cddis.2014.410 (PMC4649516; doi:10.1038/cddis.2014.410)
Supplement: Supplementary Figure S7, S8, S9, S10, S11 and S12 [file cddis2014410x4.pdf]

Figure S7

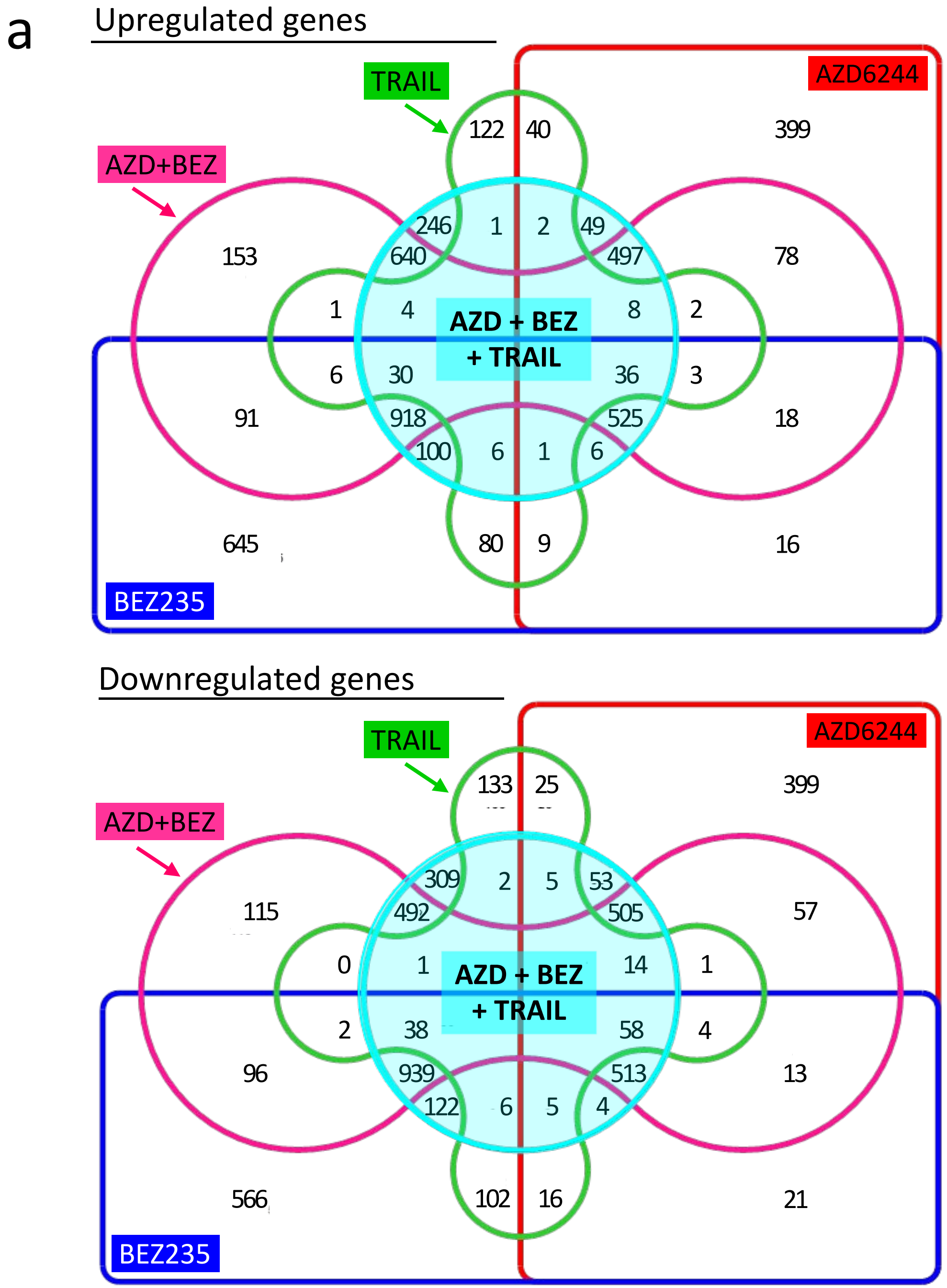
a
Upregulated genes
TRAIL
AZD6244
AZD+BEZ
122
40
399
153
246
1
2
49
640
497
78
1
4
AZD + BEZ
+ TRAIL
8
2
6
30
36
3
91
918
525
18
100
6
1
6
645
80
9
16
BEZ235
Downregulated genes
TRAIL
AZD6244
AZD+BEZ
133
25
399
115
309
2
5
53
492
505
57
0
1
AZD + BEZ
+ TRAIL
14
1
2
38
58
4
96
939
513
13
122
6
5
4
566
102
16
21
BEZ235

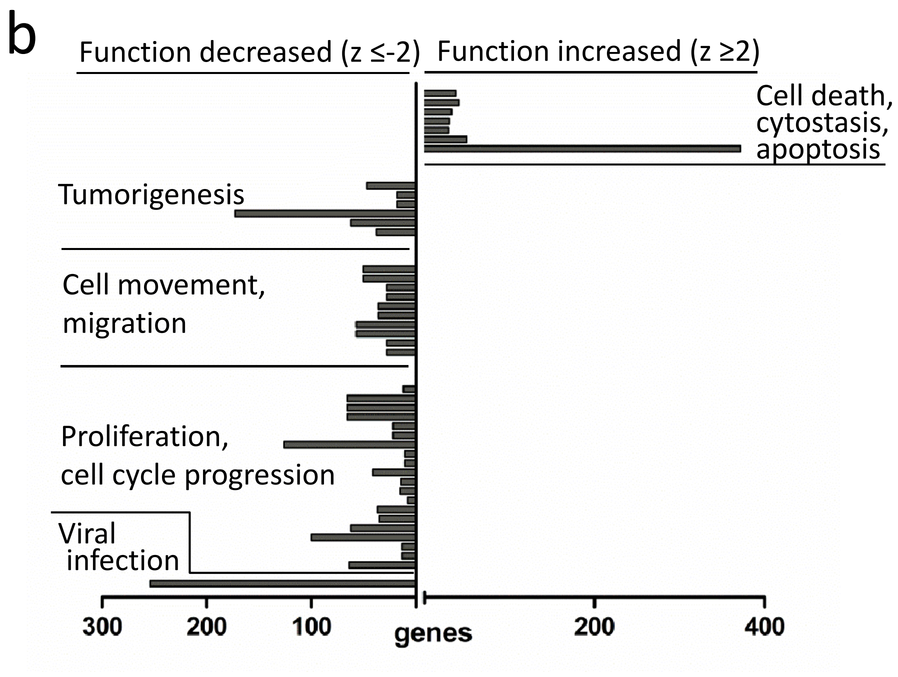
b
Function decreased (z ≤-2)
Function increased (z ≥2)
Cell death,
cytostasis,
apoptosis
Tumorigenesis
Cell movement,
migration
Proliferation,
cell cycle progression
Viral
infection
300
200
100
genes
200
400

Figure S7. Gene expression profiling of melanoma cells treated with AZD6244+BEZ235+TRAIL indicates significant increase of biological functions related to cell death. (a), Edwards-VENN diagram analysis of significantly modulated genes (upper panel, upregulated genes; lower panel, downregulated genes) in Me13 cells treated with AZD6244 (red rectangle), or BEZ235 (blue rectangle) or AZD6244+TRAIL (fuchsia shape), TRAIL (green cogwheel) or the AZD6244+BEZ235+TRAIL association (light blue circle). **(b)**, Downstream effect analysis (by IPA) based on the two subsets of genes identified by the light blue circles in panel **a**. See Table **S3** for the list of genes. Only biological functions with significant z score statistic (>2, indicating increase of biological function, or <-2, indicating decrease of biological function) and significant overlap p value are shown.

Figure S8

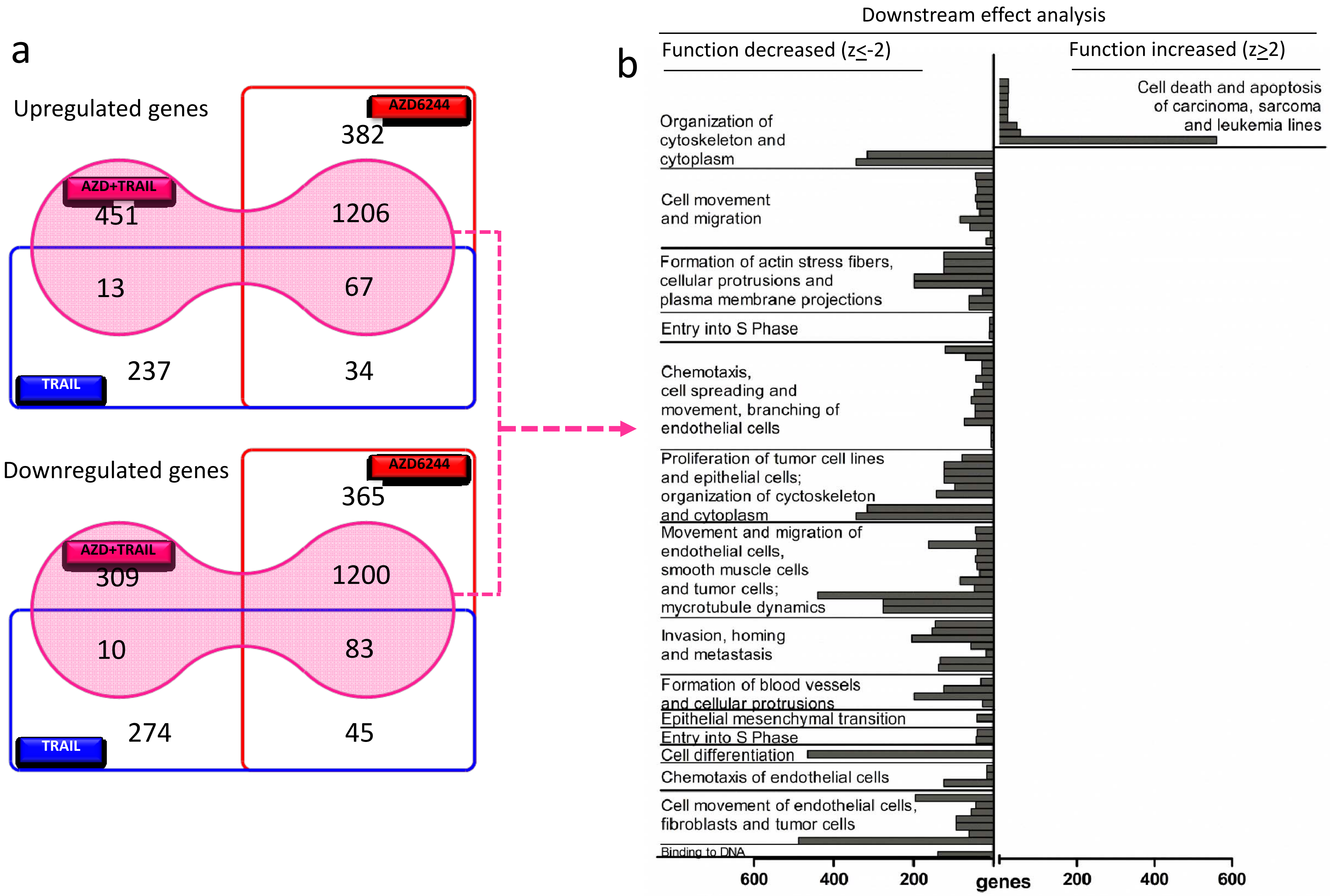

a
Upregulated genes
AZD6244
382
AZD+TRAIL
451
1206
13
67
TRAIL
237
34
Downregulated genes
AZD6244
365
AZD+TRAIL
309
1200
10
83
TRAIL
274
45
b
Downstream effect analysis
Function decreased (z≤-2)
Function increased (z≥2)
Cell death and apoptosis of carcinoma, sarcoma and leukemia lines
Organization of cytoskeleton and cytoplasm
Cell movement and migration
Formation of actin stress fibers, cellular protrusions and plasma membrane projections
Entry into S Phase
Chemotaxis, cell spreading and movement, branching of endothelial cells
Proliferation of tumor cell lines and epithelial cells; organization of cyctoskeleton and cytoplasm
Movement and migration of endothelial cells, smooth muscle cells and tumor cells; mycrotubule dynamics
Invasion, homing and metastasis
Formation of blood vessels and cellular protrusions
Epithelial mesenchymal transition
Entry into S Phase
Cell differentiation
Chemotaxis of endothelial cells
Cell movement of endothelial cells, fibroblasts and tumor cells
Binding to DNA
600
400
200
genes
200
400
600

Figure S8. Gene expression profiling of melanoma cells treated with AZD6244, TRAIL and their combination. (a), Edwards-VENN diagram analysis of significantly modulated genes (upper panel, upregulated genes; lower panel, downregulated genes) in ME13 cells treated with AZD6244 (red rectangle), or TRAIL (blue rectangle) or AZD6244+TRAIL (fuchsia shape). **(b)**, Downstream effect analysis (by IPA) based on the two subsets of genes identified by the fuchsia shape in panel **a**. Only biological functions with significant Z score statistic (>2, indicating increase of biological function, or <-2, indicating decrease of biological function) and significant overlap P value are shown. See **Table S4** for the list of genes mentioned in panel B.

Figure S9

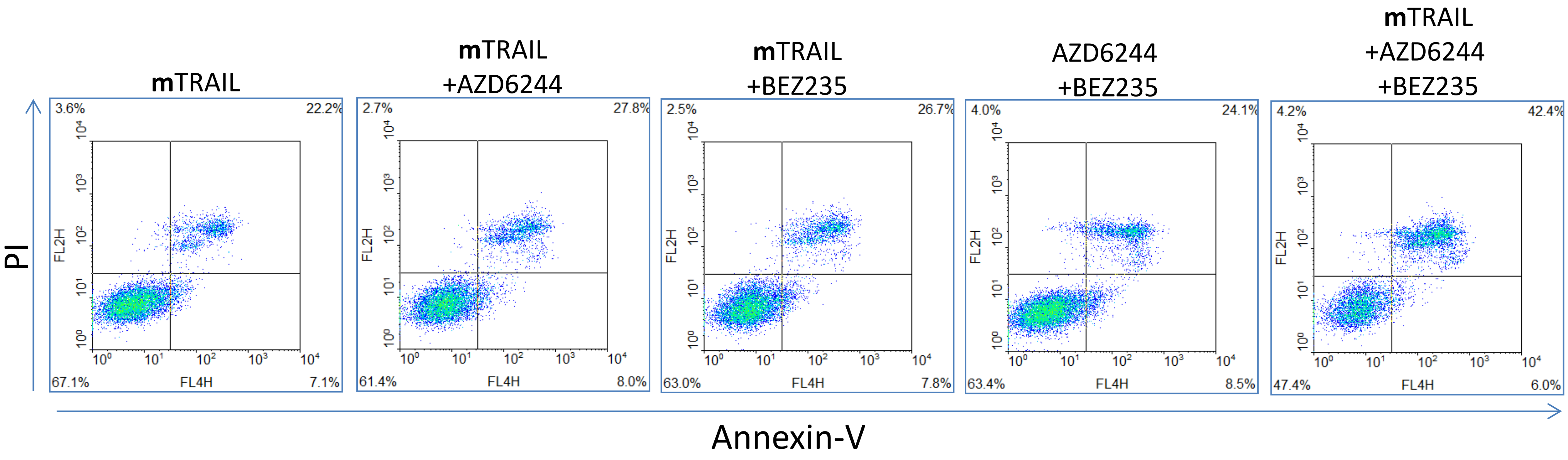

Figure S9. Enhanced melanoma apoptosis by association of mTRAIL with AZD6244 and BEZ235. Melanoma cells (Me1), stained with CFSE as described in ref. 25, were treated with AZD6244 (0.05 mM), BEZ235 (0.05 mM) or both inhibitors, and then co-cultured with $CD34^+$ cells transduced with an adenoviral vector encoding human TRAIL (**m**TRAIL, ref. 27). Extent of melanoma apoptosis was assessed at 72 h by staining for annexin-V and Propidium iodide (PI) after gating on $CFSE^+$ melanoma cells. Numbers in each dotplot indicate % of cells in each of the four quadrants.

Figure S10

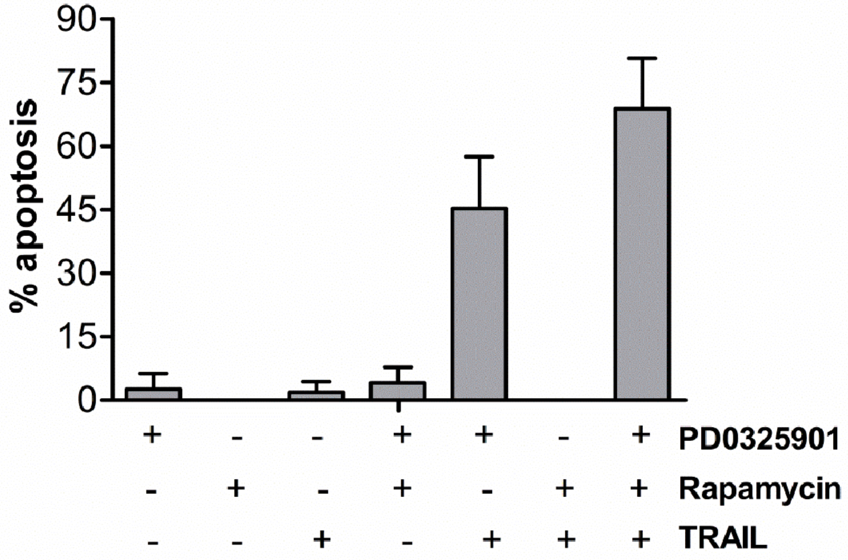

Figure S10. **Enhanced melanoma apoptosis by association of TRAIL with PD0325901 and rapamycin**. Me13 cells were treated with TRAIL (10 ng/mL), PD0325901 (5 nM), rapamycin (10 nM) or their combinations. Apoptosis was assessed at 72 h by flow cytometry. Apoptosis induced by the PD0325901/rapamycin/TRAIL and PD0325901/TRAIL combinations was significantly higher compared to the effects of single agents and of the PD0325901/rapamycin and rapamycin/TRAIL associations . Mean values (± S.D.) for three independent experiments ($p<0.001$ for all comparisons, by ANOVA and SNK test).

Figure S11

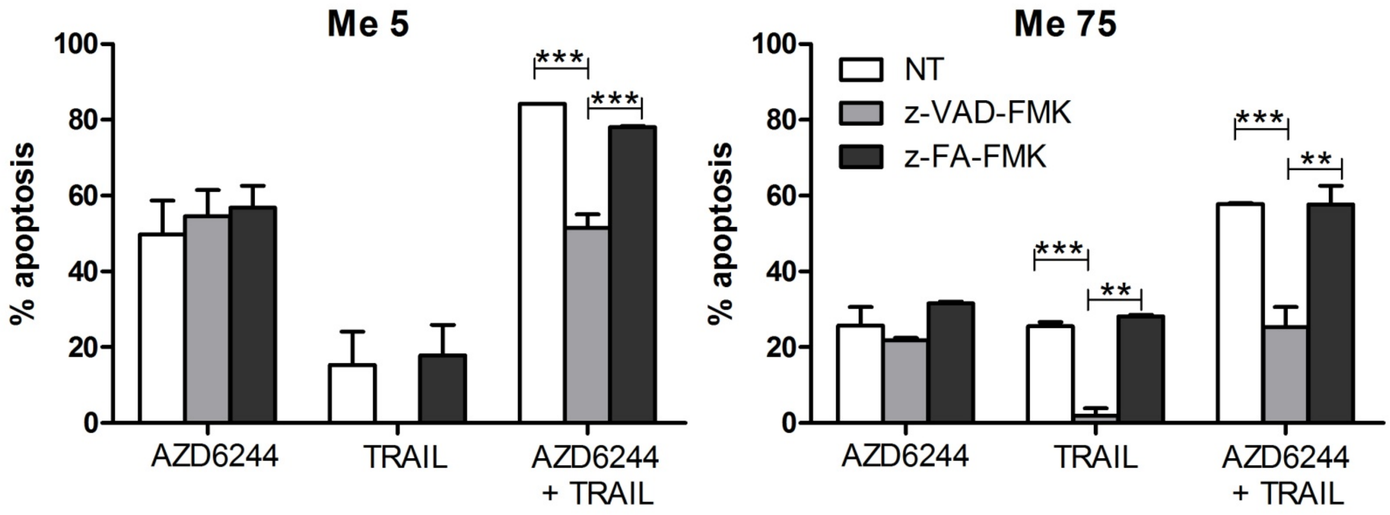

Figure S11. The association of AZD6244 and TRAIL promotes caspase-dependent melanoma apoptosis. Melanoma cell lines Me5 and Me75 were treated with AZD6244 (0.05 μM), or TRAIL (25 ng/mL) or their combinations for 72 h, in the presence of the pancaspase inhibitor z-VAD-fmk or of the negative control peptide z-FA-fmk, and apoptosis was assessed by Annexin-V/PI assay. Results shown as sum of early (annexin-V^+ PI^-) and late (annexin-V^+ PI^+) apoptosis values. Mean values (± S.D.) for three independent experiments. Statistical analysis by ANOVA followed by SNK test. ***: p<0.001, **: p<0.01.

Figure S12

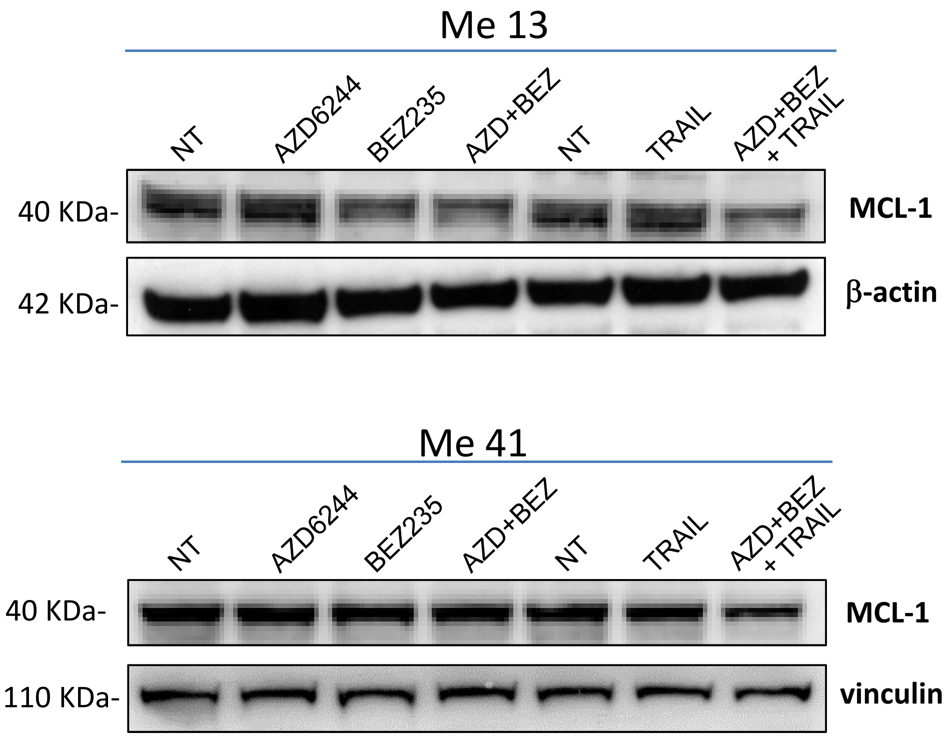

Figure S12. Modulation of Mcl-1 in melanoma cells treated with TRAIL, AZD6244, BEZ235 and their combinations. Modulation of MCL-1 expression by AZD6244, BEZ235, TRAIL and their combination assessed in two melanoma cell lines (Me 13 and Me 41) by Western Blot analysis at 48h of treatment.
