# Supplementary material for: Synergistic anti-tumor activity and inhibition of angiogenesis by cotargeting of oncogenic and death receptor pathways in human melanoma
Source: Cell Death Dis. 2014 Oct 2;5(10):e1434–. doi: 10.1038/cddis.2014.410 (PMC4649516; doi:10.1038/cddis.2014.410)
Supplement: Supplementary Figure S13, S14 and S15 [file cddis2014410x5.pdf]

Figure S13

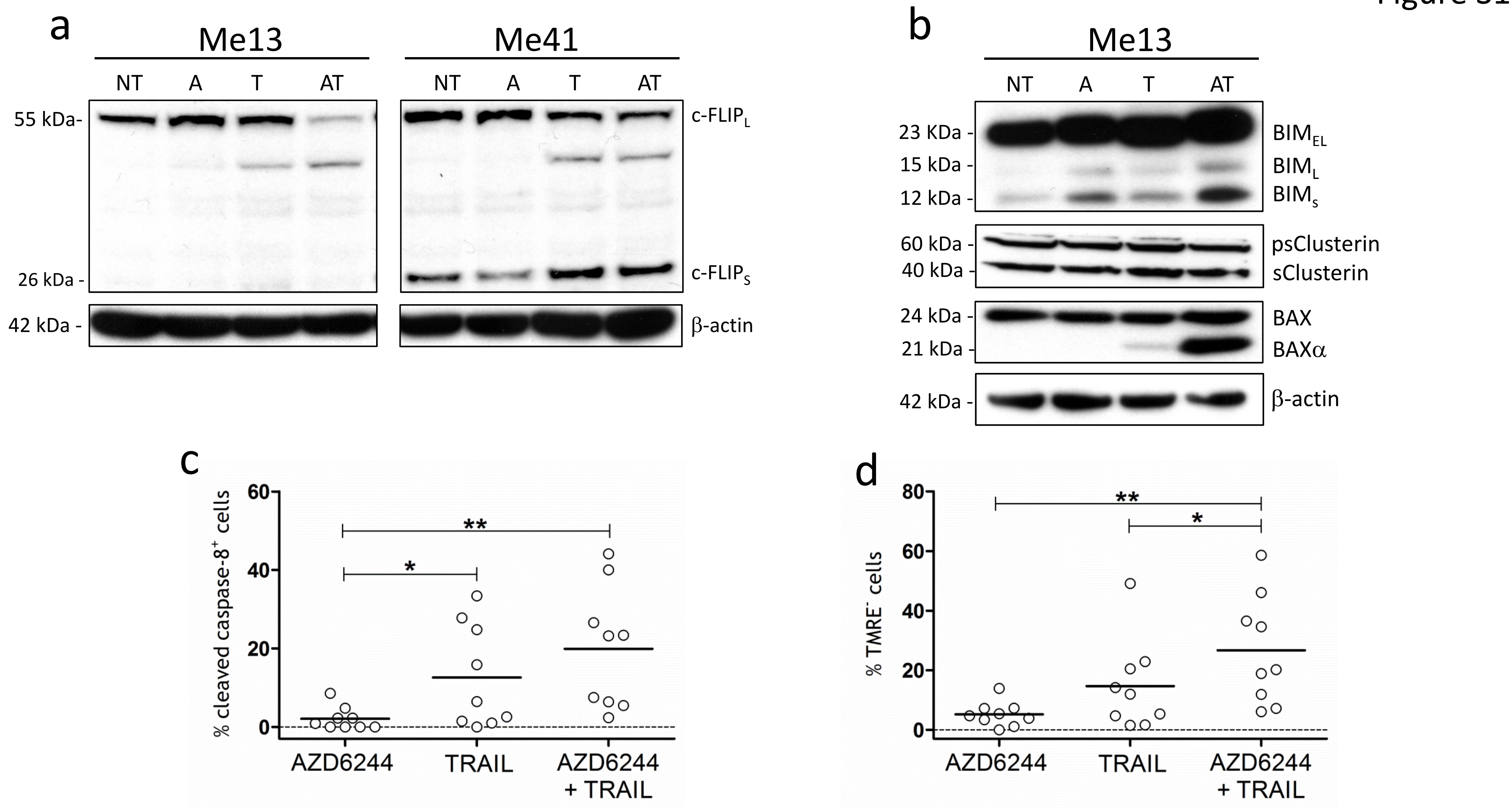

Figure S13. Modulation of pro- and anti-apoptotic molecules by melanoma treatment with the association of AZD6244 and TRAIL . (**a**), western blot analysis for c-FLIP expression in two melanoma cell lines (Me13 and Me41) treated with AZD6244 (A), TRAIL (T) or their combination (AT). (**b**), western blot analysis for expression of BIM, clusterin and BAX in Me13 cells treated as in (**a**). (**c**), cleaved caspase-8 analysis in a panel of 9 cell lines treated as in (**a**). (**d**), TMRE analysis for mitochondrial depolarization in a panel of 9 melanoma cell lines treated as in (**a**). Statistical analysis in **c, d** by ANOVA followed by SNK test; **, p<0.01; *, p<0.05.

Figure S14

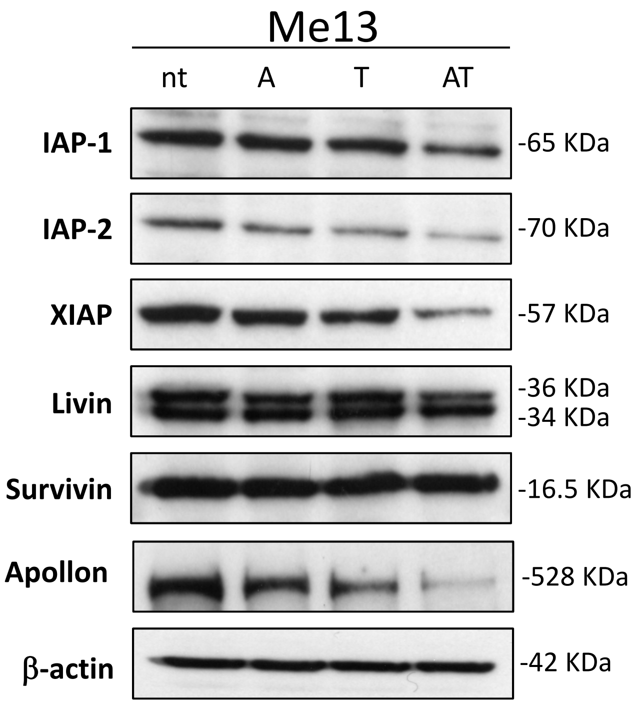

Figure S14. Modulation of the indicated IAP proteins by treatment of melanoma cells (Me13) with AZD6244 (A), or TRAIL (T) or AZD/TRAIL (AT) combination.

Figure S15

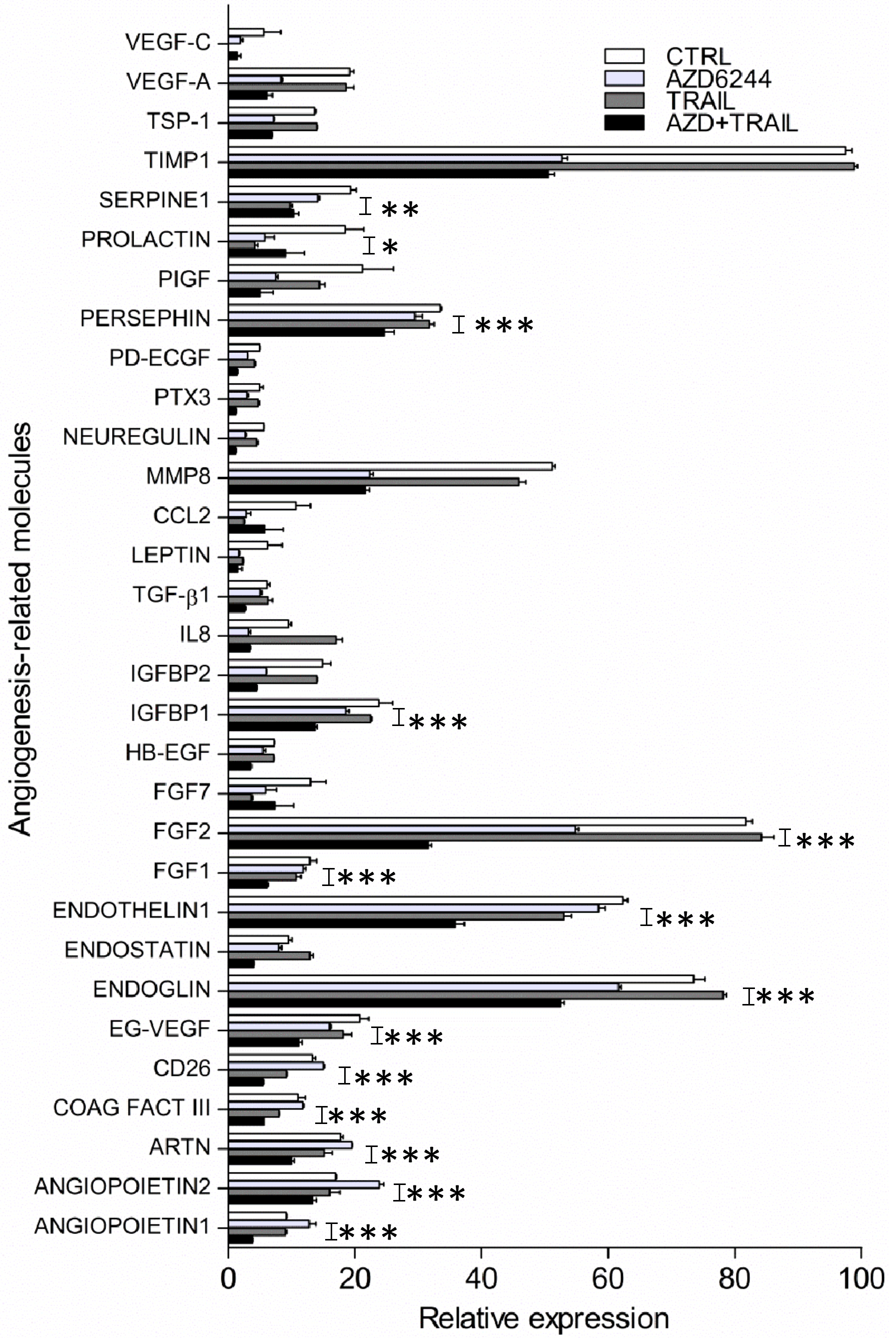

Figure S15. Modulation of angiogenesis-related molecules in Me13 cells treated with AZD6244, TRAIL and their combination. Modulation of the indicated molecule was assessed by protein array screening at 24 h after melanoma culture in the presence of the indicated drugs and drug combinations. AZD6244, and TRAIL were used at 0.1 μM, and 25 ng/mL respectively. All molecules shown in the figure were significantly modulated, compared to control untreated cells, by the AZD6244+TRAIL combination (P at least <0.05, by ANOVA and SNK test). In addition, the indicated statistical comparisons highlight instances where protein modulation by AZD6244+TRAIL is significantly different compared to results achieved by AZD6244 treatment.

***: p<0.001, **: p<0.01; *: P<0.05.
