# Supplementary material for: Synergistic anti-tumor activity and inhibition of angiogenesis by cotargeting of oncogenic and death receptor pathways in human melanoma
Source: Cell Death Dis. 2014 Oct 2;5(10):e1434–. doi: 10.1038/cddis.2014.410 (PMC4649516; doi:10.1038/cddis.2014.410)
Supplement: Supplementary Figure S16 and S17 [file cddis2014410x6.pdf]

Figure S16

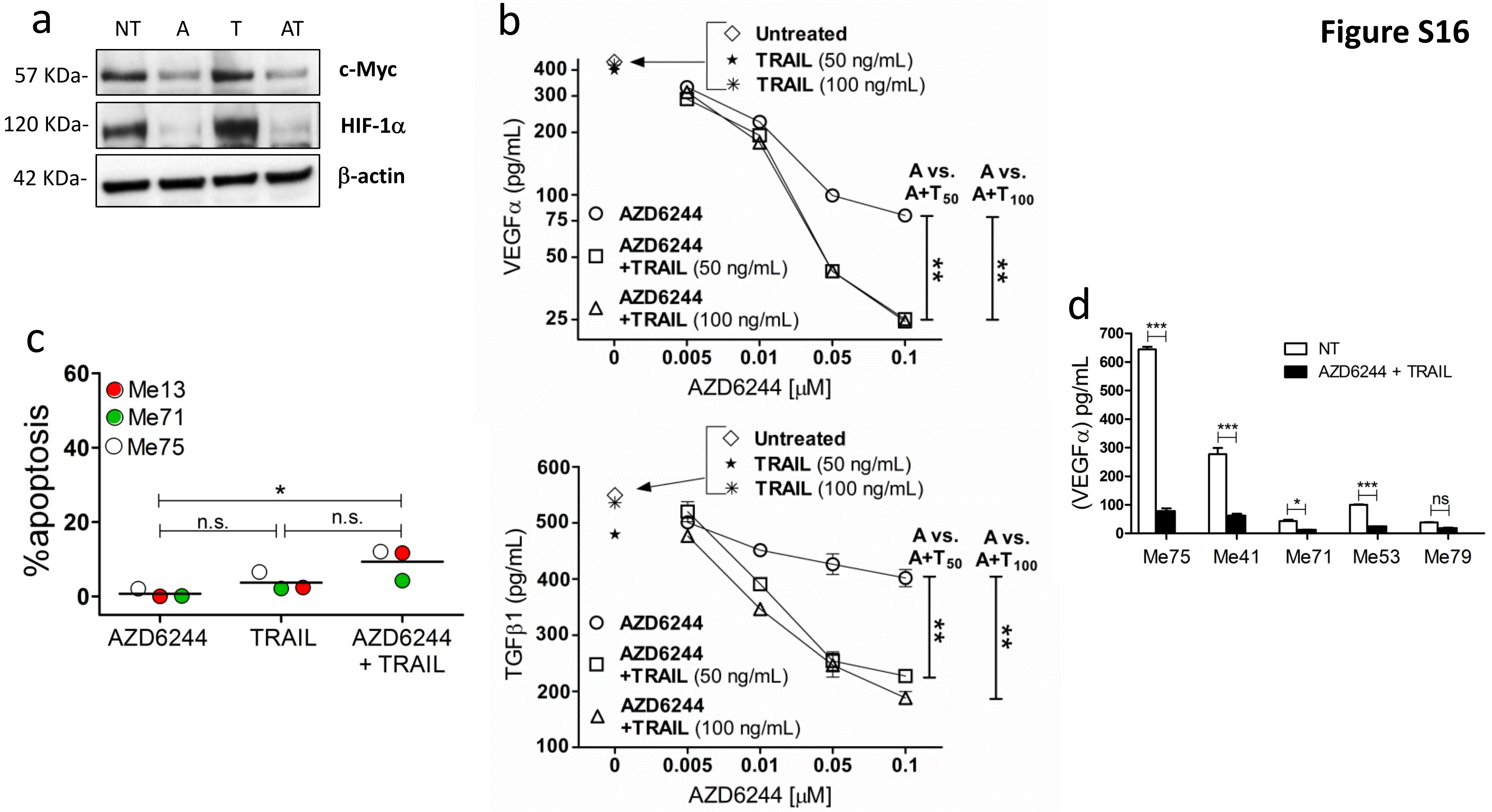

Figure S16. Inhibition of pro-angiogenic molecules in vitro by AZD6244 plus TRAIL treatment of melanoma cells. (a), Western blot analysis for Myc and HIF-1α in Me13 cells treated for 18 h in-vitro with AZD6244 (A), TRAIL (T) or their combination. (**b**), Secretion of VEGFα and TGFβ1 by Me13 treated in-vitro for 18 h with AZD6244, or TRAIL or their association. (**c**), apoptosis assay on three melanoma cell lines treated for 18 h with the AZD6244-TRAIL association (AZD6244= 0.05 μM; TRAIL=50 ng/mL). (**d**), Inhibition of VEGFα secretion in five melanoma cell lines by AZD6244+TRAIL treatment for 18 h as in (c). Statistical analysis in (b,c, d) by ANOVA and SNK test; ***: p<0.001; **: p<0.01; *: p<0.05.

Figure S17

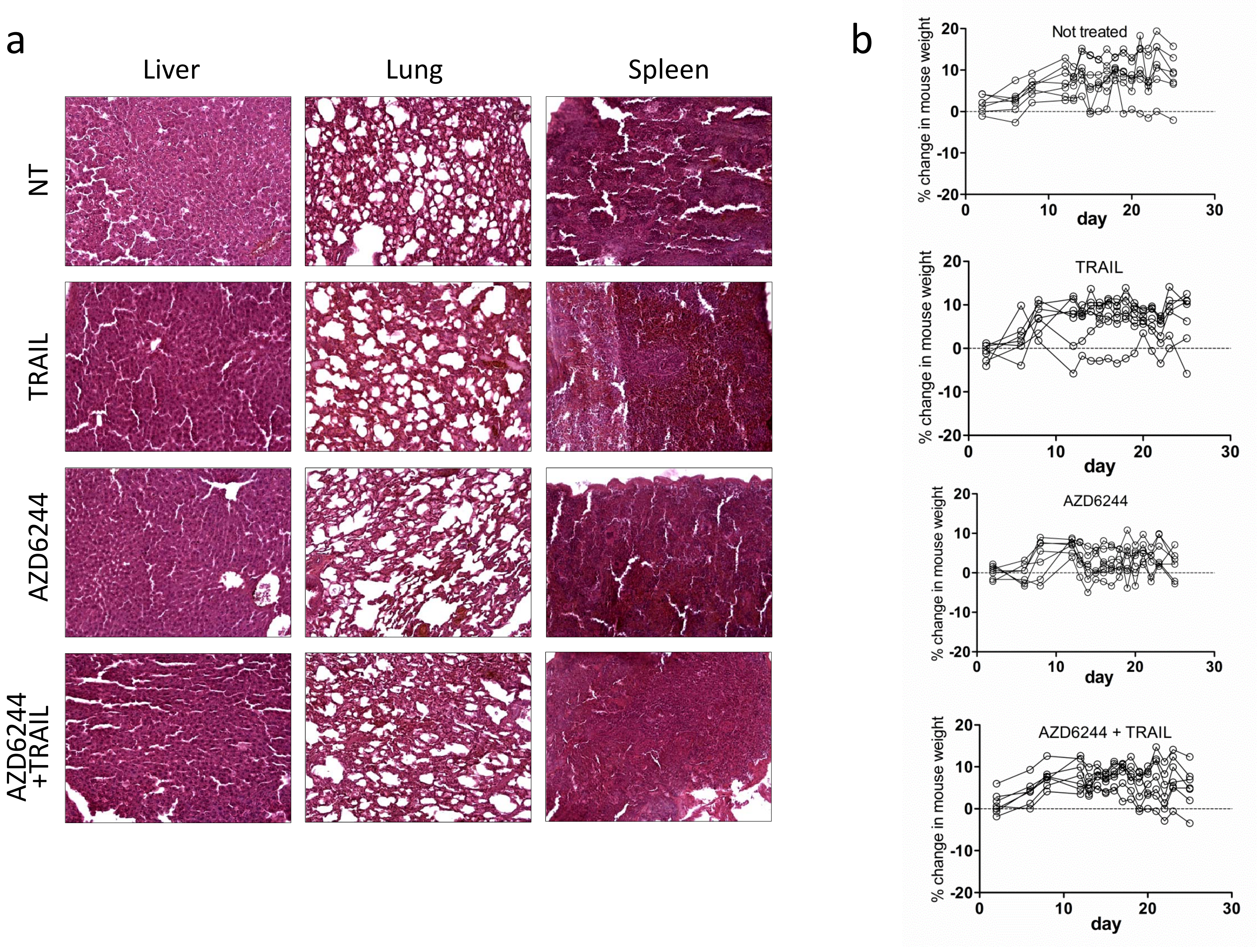

a
Liver
Lung
Spleen
NT
TRAIL
AZD6244
AZD6244 +TRAIL
b
Not treated
TRAIL
AZD6244
AZD6244 + TRAIL
% change in mouse weight
day
20
10
0
-10
-20
0
10
20
30

Figure S17. Tissue and body weight analysis of mice treated with AZD6244, TRAIL and their combination. (a), representative EE stainings of tissue sections from liver, lung and spleen of control and treated mice. (**b)**, body weight analysis of individual mice (n=7/treatment group) during treatment with AZD6244, TRAIL and their combination. Results shown as % change in body weight compared to pre-treatment values of each mouse.
