# Supplementary material for: Synergistic anti-tumor activity and inhibition of angiogenesis by cotargeting of oncogenic and death receptor pathways in human melanoma
Source: Cell Death Dis. 2014 Oct 2;5(10):e1434–. doi: 10.1038/cddis.2014.410 (PMC4649516; doi:10.1038/cddis.2014.410)
Supplement: Supplementary Tables S1, S2, S3, S4 and S5 [file cddis2014410x7.pdf]

Table S1 **Molecular and biological features of melanoma cell lines used in this study**

		Molecular features of the cell lines[b]			Susceptibility to TRAIL[c] (MTT assay, 100ng/mL, 48h)			Susceptibility to MEK and PI3K/mTOR inhibitors[d] (MTT assay, 48 h)			
Tumor code used in this study	Tissue of origin of cell line[a]	BRAF/NRAS status	PTEN status (gene/protein)	p53 status	Growth inhibition (% dead cells)	Mitochondrial depolarization (% TMRE$^-$ cells)	Caspase-8 activation (% cleaved caspase 8$^+$ cells)	AZD6244 (IC_{50}, μM)	BEZ235 (IC_{50}, μM)	Tumor code used in previous papers	References
Me1	ln met.	BRAF^{V600E}	wt/+	wt	94.1	49.0	28.0	0.050	0.080	Me14464	27, 42, 48
Me2	ln met.	BRAF^{V600E}	wt/+	wt	76.5	58.0	32.0	0.120	0.225	Me4023	27, 48
Me5	s.c. met.	BRAF^{V600E}	ex.5 del$_{403-409}$/-	wt	26.0	12.2	16.0	0.015	0.070	-	This manuscript
Me6	s.c. met.	BRAF^{V600E}	wt/+	wt	28.0	43.6	28.3	0.350	0.088	Me6824	42
Me13	ln met.	BRAF^{V600E}	wt/+	wt	6.2	4.7	2.6	0.308	0.109	Me15392	27, 42, 48
Me15	ln met.	BRAF^{V600E}	wt/+	wt	96.4	14.2	33.0	0.015	0.045	Me23682	27
Me17	ln met.	BRAF^{V600E}	wt/+	C135W^{Ho}	4.0	1.7	4.5	0.029	0.092	-	This manuscript
Me20	ln met.	NRAS^{Q61R}	wt/+	wt	9.3	26.7	19.0	0.010	2.520	Me18816	42
Me25	local recurrence	BRAF^{V600E}	wt/+	Y236H	10.4	5.5	0.0	0.328	0.110	Me1402r	48
Me27	ln met.	BRAF^{V600E}	wt/-	wt	17.0	19.6	16.3	0.186	0.178	Me13294	27
Me30	ln met.	BRAF^{V600E}	wt/+	wt	44.6	1.6	4.5	0.058	0.586	Me18656	27, 42, 48
Me32	VGP primary me.	NRAS^{G12S}	wt/+	wt	87.4	23.9	0.1	0.410	0.030	Me9923p	48
Me33	ln met.	BRAF^{V600E}	wt/+	wt	0.0	0.0	0.5	0.023	0.283	-	This manuscript
Me34	ln met.	NRAS^{Q61R}	wt/+	Y126H^{Ho}	71.5	15.3	14.4	0.560	0.030	-	This manuscript
Me36	ln met.	wt/wt	wt/+	wt	30.0	0.5	5.7	0.780	1.630	Me879	48
Me40	ln met.	BRAF^{V600E}	wt/-	wt	1.5	0.1	17.3	0.280	0.100	-	This manuscript
Me41	ln met.	BRAF^{V600E}	wt/+	wt	35.0	20.5	6.5	0.020	0.048	Me32562	42
Me43	ln met.	BRAF^{V600E}	wt/+	wt	42.2	2.2	13.1	0.029	0.168	Me18732	27, 42, 48
Me44	ln met.	BRAF^{V600E}	wt/+	wt	4.7	0.6	7.0	0.050	0.220	Me16938	42
Me46	s.c. met.	BRAF^{V600E}	wt/+	wt	7.9	1.5	1.0	0.020	0.030	-	This manuscript
Me49	ln met.	BRAF^{V600E}	P246S/+	S127F^{Ho}	19.8	0.0	1.1	0.049	0.089	Me2211	27, 42, 48
Me50	ln met.	BRAF^{V600E}	P246S^{He}/+	S127F^{Ho}	53.4	12.6	9.5	0.060	0.160	-	This manuscript
Me53	s.c. met.	BRAF^{V600E}	wt/+	wt	44.0	17.6	12.0	0.288	0.012	Me32669	27, 42
Me55	ln met.	wt/wt	wt/+	wt	63.0	9.7	20.0	0.720	1.140	Me3700	42
Me56	ln met.	BRAF^{V600E}	P38S/-	S127F	8.1	0.0	3.6	0.012	3.679	Me4686	48
Me57	VGP primary me.	wt/wt	wt/+	R213R	2.4	3.2	0.6	0.020	0.020	Me1007	48
Me58	ln met.	BRAF^{V600E}	wt/+	wt	11.0	22.9	12.0	0.387	5.429	Me2559	42
Me59	ln met.	NRAS^{Q61R}	wt/+	wt	29.4	21.7	0.0	0.030	0.590	Me4473	42, 48
Me63	ln met.	BRAF^{V600E}	Y223STOP^{He} / -	wt	25.8	3.1	0.8	0.085	4.796	-	This manuscript
Me64	ln met.	wt/wt	nd/+	wt	3.3	0.8	1.7	0.050	0.020	Me13923	48
Me67	soft tissue met.	NRAS^{Q61R}	wt/+	R213R^{He}	92.5	25.5	25.0	9.770	0.380	Me3044	42
Me69	ln met.	BRAF^{V600E}	wt/+	Y234C	46.7	15.5	10.5	0.214	12.882	Me17697	48
Me71	ln met.	BRAF^{V600E}	wt/+	wt	3.5	17.7	25.0	0.010	0.047	Me21158	27, 42
Me73	ln met.	BRAF^{V600E}	del$_{ex\ 3-5-6}$/-	P128S^{He}	18.0	5.3	0.0	0.019	0.062	Me1274	42
Me75	VGP primary me.	BRAF^{V600E}	wt/+	wt	0.0	1.5	5.8	0.047	0.222	Me10258	42
Me76	ln met.	BRAF^{V600E}	wt/+	E258K	21.4	0.0	0.8	0.016	0.067	Me14362	27, 42, 48
Me78	ln met.	BRAF^{V600E}	wt/+	wt	10.5	0.0	1.3	0.069	6.098	-	This manuscript
Me79	ln met.	BRAF^{V600E}	wt/+	wt	17.0	4.3	1.9	1.224	96.746	Me2934	27, 42, 48
Me83	ln met.	wt/wt	Q171Q / +	wt	65.7	13.8	11.0	0.120	0.100	Me2352	27, 42
Me85	VGP primary me.	BRAF^{V600E}	wt/+	wt	49.0	38.9	6.3	0.014	0.525	-	This manuscript
Me86	soft tissue met.	NRAS^{Q61R}	wt/+	wt	48.1	23.0	22.0	0.020	0.200	Me15094	27
Me88	ln met.	wt/wt	wt/+	wt	71.1	12.0	0.0	0.020	0.160	Me19410	42
Me92	VGP primary me.	BRAF^{L596S}	wt/+	G187S	0.7	3.6	0.0	0.069	0.107	Me20842	27, 42, 48
Me93	ln met.	BRAF^{L596S}	wt/+	wt	8.5	5.7	0.9	0.036	0.076	Me20842M1	48
Me94	s.c. met.	BRAF^{L596S}	wt/+	G187S	51.2	12.2	2.7	0.018	0.048	Me20842M2	48
Me96	ln met.	BRAF^{V600E}	wt/+	wt	17.2	0.0	0.6	0.030	0.280	Me9874	42
Me98	ln met.	BRAF^{V600E}	nd/+	wt	0.0	0.5	0.8	0.042	0.048	Me29318	42
Me99	VGP primary me.	BRAF^{V600E}	P89S, del$_{ex\ 6-8}$ /-	wt	13.5	2.0	0.7	8.697	0.212	Me26635	42
Me100	VGP primary me.	NRAS^{Q61R}	wt/+	null	52.1	0.0	2.2	0.120	0.560	Me4405	27, 42, 48

[a] Melanoma cell lines were isolated from: vertical growth phase primary melanomas (VGP primary me.), lymph node metastases (ln met.), subcutaneous metastases (s.c. met.), soft tissues metastases or local recurrences.

[b]Methods for identification of BRAF, NRAS, PTEN and p53 mutations are described in refs. 48, 49. PTEN data expressed as gene sequence data / protein expression by western blot. He:heterozygous; Ho: homozygous.

[c]Susceptibility to TRAIL was assessed by a 48 h MTT assay. Mitochondrial depolarization (TMRE assay) and caspase-8 cleavage in response to TRAIL were assessed at 24 h by flow cytometry.

[d] Susceptibility to AZD6244 and BEZ235, shown as IC_{50} values, was assessed by a 48 h MTT assay. IC_{50} values were obtained trough non linear regression analysis of dose response curves. See **Fig. 1A** for representative examples of dose-response plots.

Table S2. Statistical analysis of fraction affected (FA) data.

Comparison 1: High vs. low TRAIL doses in the AZD6244+BEZ235+TRAIL combination: significant FA increase at higher TRAIL doses

		**FA values in melanoma cells treated with the association**		
		AZD6244 →	**0.05** µM	**0.05** µM
		BEZ235 →	**0.005** µM	**0.005** µM
		TRAIL →	**10** ng/mL	**25** ng/mL
**FA values in melanoma cells treated with the association of:**	AZD6244 (**0.05** µM) BEZ235 (**0.005** µM) TRAIL (**5** ng/mL)		ns	**
	AZD6244 (**0.05** µM) BEZ235 (**0.005** µM) TRAIL (**10** ng/mL)			**

		**treated with the association of:**		
		AZD6244 →	**0.05** µM	**0.05** µM
		BEZ235 →	**0.01** µM	**0.01** µM
		TRAIL →	**10** ng/mL	**25** ng/mL
**FA values in melanoma cells treated with the association of:**	AZD6244 (**0.05** µM) BEZ235 (**0.01** µM) TRAIL (**5** ng/mL)		ns	**
	AZD6244 (**0.05** µM) BEZ235 (**0.01** µM) TRAIL (**10** ng/mL)			**

		**FA values in melanoma cells treated with the association**		
		AZD6244 →	**0.05** µM	**0.05** µM
		BEZ235 →	**0.02** µM	**0.02** µM
		TRAIL →	**10** ng/mL	**25** ng/mL
**FA values in melanoma cells treated with the association of:**	AZD6244 (**0.05** µM) BEZ235 (**0.02** µM) TRAIL (**5** ng/mL)		ns	*
	AZD6244 (**0.05** µM) BEZ235 (**0.02** µM) TRAIL (**10** ng/mL)			*

Comparison 2: AZD6244+TRAIL vs. AZD6244+BEZ235+TRAIL: significant FA increase by adding BEZ235

		**FA values in melanoma cells treated with the association of:**											
		AZD6244 →	**0.05** µM	**0.05** µM	**0.05** µM	AZD6244 →	**0.05** µM	**0.05** µM	**0.05** µM	AZD6244 →	**0.05** µM	**0.05** µM	**0.05** µM
		BEZ235 →	**0.005** µM	**0.01** µM	**0.02** µM	BEZ235 →	**0.005** µM	**0.01** µM	**0.02** µM	BEZ235 →	**0.005** µM	**0.01** µM	**0.02** µM
		TRAIL →	**5** ng/mL	**5** ng/mL	**5** ng/mL	TRAIL →	**10** ng/mL	**10** ng/mL	**10** ng/mL	TRAIL →	**25** ng/mL	**25** ng/mL	**25** ng/mL
**FA values in melanoma cells treated with the association of:**	AZD6244 (**0.05** µM) TRAIL (**5** ng/mL)		*	**	***								
	AZD6244 (**0.05** µM) TRAIL (**10** ng/mL)						ns	**	***				
	AZD6244 (**0.05** µM) TRAIL (**25** ng/mL)										ns	**	***

Comparison 3: AZD6244+BEZ235 vs AZD6244+BEZ235+TRAIL: Significant FA increase by adding high dose TRAIL

		**FA values in melanoma cells treated with the association of:**											
		AZD6244 →	**0.05** µM	**0.05** µM	**0.05** µM	AZD6244 →	**0.05** µM	**0.05** µM	**0.05** µM	AZD6244 →	**0.05** µM	**0.05** µM	**0.05** µM
		BEZ235 →	**0.005** µM	**0.005** µM	**0.005** µM	BEZ235 →	**0.01** µM	**0.01** µM	**0.01** µM	BEZ235 →	**0.02** µM	**0.02** µM	**0.02** µM
		TRAIL →	**5** ng/mL	**10** ng/mL	**25** ng/mL	TRAIL →	**5** ng/mL	**10** ng/mL	**25** ng/mL	TRAIL →	**5** ng/mL	**10** ng/mL	**25** ng/mL
**FA values in melanoma cells treated with the association of:**	AZD6244 (**0.05** µM) BEZ235 (**0.005** µM)		ns	ns	***								
	AZD6244 (**0.05** µM) BEZ235 (**0.01** µM)						ns	ns	***				
	AZD6244 (**0.05** µM) BEZ235 (**0.02** µM)										ns	ns	***

The analysis, carried out by ANOVA followed by SNK test, is based on FA data generated by Compusyn software on a dataset of the 21 melanoma cell lines (shown in **Fig. 2, Figure S5 and S6**) and treated with the indicated combinations of target-specific inhibitors and TRAIL.

***: p<0.05; **: p<0.01; ***: p<0.001**

Table S3. Downstream effect analysis by IPA software of significantly modulated genes by the association of TRAIL, AZD6244 and BEZ235.

Results shown here are based on the set of genes identified by the light blue circles in **Fig.S7A.**
See supplementary methods for the meaning of the p values and of the z score statistics.

Category	Functions Annotation	p-Value	Predicted Activation State	Activation z-score	Molecules	# Molecules	Shown in Fig. S4b
Cell Death and Survival	cell death of melanoma cell lines	6.46E-07	Increased	2.108	ATM, BAG3, BBC3, BIRC5, BMF, CDKN1B, DAB2, DDIT3, DIABLO, EGR1, EPOR, ETS1, FGF2, FOXD3, FOXO3, GADD45A, HSPA5, HSPB8, IL8, MAP2K1, MCL1, MITF, NME1, PAX3, PMAIP1, PPP1R15A, SMOX, SPHK1, STAT2, TFAP2A, TNFRSF1A, TOP1	32	yes
Cell Death and Survival	apoptosis of melanoma cell lines	2.24E-06	Increased	2.188	ATM, BAG3, BBC3, BIRC5, BMF, CDKN1B, DAB2, DDIT3, DIABLO, EGR1, EPOR, ETS1, FGF2, FOXO3, GADD45A, HSPA5, HSPB8, MAP2K1, MCL1, MITF, NME1, PAX3, PMAIP1, PPP1R15A, SMOX, SPHK1, STAT2, TNFRSF1A, TOP1	29	yes
Organismal Survival	organismal death	1.32E-05	Increased	2.819	ABCB6, ABL1, ABL2, ACACA, ACLY, ACTB, ACTG1, ACTN4, ADAM19, ADAMTS1, ADM, ADRB2, ADRBK1, AFG3L1P, AGRN, AHI1, AHR, AIM2, AKAP12, AKT1, ALDH1A3, ANGPTL4, ANK3, ANTXR2, APEX1, ARAP3, ARHGDIA, ARID4B, ARID5B, ARNTL, ARRB1, ASPA, ATF4, ATM, ATP2B1, ATXN3, B3GNT5, BAG3, BBC3, BCL6, BID, BIRC5, BMF, BMPR1A, C1GALT1, C3AR1, C9orf114, CA14, CABIN1, CBFB, CBLB, CBX2, CBY1, CCDC86, CCNB2, CCND1, CCND3, CCNE1, CCNE2, CD151, CD200, CD36, CD44, CDC25A, CDK19, CDK6, CDKN1B, CDKN2C, CEBPB, CEBPD, CEBPG, CHUK, CITED1, CITED2, CLDN1, COL5A2, CPOX, CREB3L2, CREB5, CSK, CSRNP1, CTLA4, CTNNB1, CTSF, CTSL2, CXCR4, CYR61, DAB2, DACT1, DDAH1, DDB2, DDR1, DHCR7, DHX58, DLX5, DMRT2, DNM1L, DUSP1, DYNC2LI1, E2F3, E2F4, E2F5, ECE2, EDNRB, EEF1E1, EGLN1, EGR3, EHD4, EHHADH, EIF2C2, EIF2S1, ELK3, ENDOG, ENG, ENPP2, EPAS1, EPOR, ERRFI1, ETS1, ETS2, EYA4, FABP3, FAIM, FAM20C, FASN, FBXO4, FBXO45, FBXW7, FCER1G, FEN1, FGF2, FGFR3, FGFRL1, FKBP4, FN1, FNDC3B, FOSL1, FOXD3, FOXF2, FOXO3, FOXQ1, FSTL1, FURIN, FXR1, GADD45A, GAS1, GCH1, GCLM, GHR, GJB1, GRN, GSTZ1, H1F0, HAS2, HBEGF, HEXA, HEY1, HEY2, HIF1A, HIPK2, HIST2H2AA3/HIST2H2AA4, HK2, HLX, HMBS, HMGCL, HMGN2, HPGD, HS3ST1, HSPA1A/HSPA1B, HSPA4L, HSPA5, HSPB8, HTR2B, HYAL2, ICMT, ID1, ID2, IDE, IFNAR1, IFNAR2, IFNGR1, IFT122, IGFBP3, IGFBP5, IL12A, IL1B, IL27RA, IL6, IL8, ING4, INSIG1, INSIG2, IP6K2, IRAK1, IRF9, IRS2, ITGA3, ITGA4, ITGA5, ITPKB, JUP, KIF5B, KIT, KLF2, KLF6, KPNA1, KRAS, KRR1, LAMP2, LATS2, LEF1, LFNG, LIF, LIMK1, LMNB2, LMO4, LPL, LRP5, LRP8, LTBR, LYN, MAFB, MAFF, MAP2K1, MAP2K3, MAP2K5, MAP3K12, MARCKS, MARCKSL1, MCL1, MCM10, MEF2C, MEF2D, MET, MGAT1, MITF, MKKS, MNT, MTA2, MTERFD1, MTHFR, MXI1, MYCBP2, NFAT5, NFIB, NFIC, NKX2-5, NKX3-2, NLRP3, NME1, NOA1, NOTCH1, NOTCH2, NOV, NQO1, NR5A2, NRP1, NUMA1, OAT, OPHN1, ORAI1, PAX3, PEX11B, PHB, PHC2, PIK3CB, PISD, PKNOX1, PLAT, PNN, PNPT1, POU3F1, POU3F2, PPAP2B, PPARD, PPARGC1A, PPARGC1B, PPIF, PPIG, PPME1, PPP1R14C, PPP1R15A, PPP3R1, PRNP, PRPF19, PRRX1, PSMD4, PTEN, PTGS2, PTPN1, PTRH2, PVRL3, RAB27A, RAD23B, RAPGEF1, RASSF1, RASSF2, RBBP8, RBM15, RDH10, RGS10, RIF1, RPL6, RTN4, RUNX1, RXRB, SAFB, SDC4, SEPP1, SERTAD1, SGCD, SHISA2, SIAH1, SLC20A1, SLC25A19, SLC25A37, SLC2A1, SLC2A3, SLC5A3, SLC9A3R1, SMAD3, SNAI2, SOX4, SP1, SPEN, SPRY1, SPRY2, SPRY4, ST8SIA1, STAM, STAMBP, STARD13, STAT2, STIP1, STK3, SV2A, SV2B, SYNJ1, TDG, TFAP2A, TFAP2C, TFB1M, TFDP1, TFRC, TGFA, TGFBR2, TGIF1, THBS1, THBS2, TIMP3, TK2, TNFRSF1A, TOP1, TP53INP1, TRAF3IP2, TRIM63, TRPV2, TUBG1, TXNIP, UBD, UHRF1, ULK1, VAMP2, VEGFA, VGF, VWF, WDR77, XBP1, ZBTB20, ZEB2, ZNF148	371	yes
Cell Death and Survival	cell death of prostate cancer cell lines	1.66E-05	Increased	2.080	ABCE1, ABL1, ACACA, AKAP12, AKT1, ATM, BIRC5, DDIT3, DIABLO, DKK3, EGR1, ETS2, FASN, FN1, FOXO3, GADD45A, GDF15, HBEGF, HIF1A, HOXC6, HSPA1A/HSPA1B, HSPB8, ICMT, ID1, IGFBP3, IGFBP4, IGFBP5, IL6, MAP2K1, MAP2K3, MCL1, MET, ORAI1, PIM1, PMAIP1, PTEN, PTGS2, SH3RF1, TGFA, TOP1, TPD52	41	yes
Cell Death and Survival	apoptosis of prostate cancer cell lines	2.21E-05	Increased	2.068	ABCE1, ABL1, ACACA, AKAP12, AKT1, ATM, BIRC5, DIABLO, DKK3, EGR1, ETS2, FASN, FN1, FOXO3, GADD45A, GDF15, HBEGF, HIF1A, HOXC6, HSPA1A/HSPA1B, HSPB8, ID1, IGFBP3, IGFBP4, IGFBP5, IL6, MAP2K3, MCL1, MET, ORAI1, PIM1, PMAIP1, PTEN, PTGS2, SH3RF1, TGFA, TPD52	37	yes
Cellular Growth and Proliferation	cytostasis of tumor cell lines	4.83E-04	Increased	2.184	ABL1, ADRBK1, BCL6, BHLHE40, BIRC5, BMPR1A, CCND1, CCND3, CDCA8, CDKN1B, CEACAM1, CTNNB1, CYR61, EGR1, ETS1, FOXO3, G3BP1, IL6, LIF, MXI1, NME1, NOTCH1, NQO1, PPP1R15A, PTEN, RACGAP1, RXRB, SMAD3, TGFBR2, TOP1	30	yes
Cellular Growth and Proliferation	cytostasis	7.51E-04	Increased	2.369	ABL1, ADRBK1, BCL6, BHLHE40, BIRC5, BMPR1A, CBX2, CCND1, CCND3, CDCA8, CDKN1B, CDKN2C, CEACAM1, CEBPB, CTNNB1, CYR61, EGR1, ETS1, FOXO3, G3BP1, GBP2, HAS2, HAS3, HBP1, HSPA1A/HSPA1B, ID1, ID2, IL6, ING4, KIT, KLF6, KRAS, LATS2, LIF, MXI1, NME1, NOTCH1, NQO1, PPP1R15A, PRKCZ, PTEN, PTPN1, RACGAP1, RXRB, SAFB, SMAD3, STAMBP, TFAP2A, TGFBR2, TOP1	50	yes

Category	Functions Annotation	p-Value	Predicted Activation State	Activation z-score	Molecules	# Molecules	Shown in Fig. S4b
Cell Cycle	interphase	1.21E-07	Decreased	-2.145	ABL1, AHR, AKAP12, AKT1, ANXA2, ARRB1, ATF3, ATF5, ATM, BHLHE40, BID, BIRC5, BTG1, BTK, C11orf82, CABLES1, CBX2, CCND1, CCND3, CCNE1, CCNE2, CCNG2, CD44, CDC25A, CDC25B, CDC25C, CDK5RAP3, CDK6, CDKN1B, CDKN2C, CDKN3, CDT1, CEBPB, CEBPD, COPS2, CTLA4, CTNNB1, CYR61, DDIT3, DDX3X, DEGS1, DUSP1, E2F3, E2F4, E2F5, EGR1, EPOR, ETS1, FASN, FBXL2, FEN1, FGF2, FN1, FOXO3, GADD45A, GAS1, GDF15, GHR, GRB2, HAS2, HBEGF, HINFP, ID1, ID2, ID4, IGFBP3, IGFBP5, IL1B, IL6, ING2, ING4, ITGA5, KDM5B, KIT, KLF6, KRAS, LATS2, LEF1, LIF, LIMK1, LSM10, MAP2K1, MAP2K3, MAP3K5, MCM10, MCMBP, MET, MGEA5, MT1A, MXD3, MXD4, MXI1, NOTCH1, NOTCH2, NPAT, PIM1, PNPT1, PPP2R2A, PRNP, PTEN, PTGS2, RASSF1, RBBP8, RGCC, RUNX1, SH2B3, SKP2, SP1, SPHK1, SPRY2, SRPK2, SRSF2, ST8SIA1, TAF2, TAOK2, TBRG4, TFDP1, TFDP2, TFRC, TGFA, TGFBR2, TXNIP, UHRF1, VEGFA, YWHAG, ZEB2	126	yes
Cell Cycle	S phase	1.98E-07	Decreased	-2.823	ABL1, ATM, BHLHE40, BID, BIRC5, CBX2, CCND1, CCND3, CCNE1, CCNG2, CDC25A, CDC25C, CDK6, CDKN1B, CDKN2C, CDT1, CEBPB, CTLA4, DDX3X, E2F3, E2F4, E2F5, EPOR, FEN1, FGF2, FN1, FOXO3, GADD45A, GAS1, GRB2, HBEGF, HINFP, ID1, ID2, IL1B, IL6, ING4, KRAS, LSM10, MAP2K1, MCM10, MCMBP, MET, MXD3, MXD4, MXI1, NOTCH1, NPAT, PNPT1, RASSF1, RBBP8, RGCC, RUNX1, SH2B3, SKP2, ST8SIA1, TFDP1, TFDP2, TGFA, TGFBR2, UHRF1, VEGFA	62	yes
Cell Cycle	entry into S phase	1.49E-06	Decreased	-2.733	ABL1, ATM, BHLHE40, CBX2, CCND1, CCND3, CCNE1, CDC25A, CDKN1B, CDKN2C, CEBPB, CTLA4, DDX3X, E2F3, E2F4, E2F5, FGF2, FN1, GAS1, GRB2, HBEGF, HINFP, ID2, IL1B, IL6, KRAS, MCM10, MET, NPAT, RGCC, SKP2, ST8SIA1, TFDP1, TFDP2, UHRF1	35	yes
Cell Cycle	entry into interphase	2.04E-06	Decreased	-2.663	ABL1, AKT1, ATM, BHLHE40, CBX2, CCND1, CCND3, CCNE1, CDC25A, CDKN1B, CDKN2C, CEBPB, CTLA4, DDX3X, E2F3, E2F4, E2F5, EGR1, FGF2, FN1, GAS1, GRB2, HBEGF, HINFP, ID2, IL1B, IL6, KRAS, MCM10, MET, NPAT, RGCC, SKP2, ST8SIA1, TFDP1, TFDP2, UHRF1	37	yes
Cellular Movement	cell movement of fibroblast cell lines	2.45E-06	Decreased	-2.308	ABL1, ACP1, ACTR2, AKT1, AMD1, CD44, CDKN1B, CSK, CTNNB1, DDR1, DDX3X, ELK3, FBXW7, FGF2, FN1, FOSL1, GNG12, GRB2, HAS2, HAS3, HBEGF, IL8, ITGA5, KLF2, LIMA1, MARCKS, MST1, MYLK, PTEN, PTGS2, PTPN1, PVR, RASSF1, SKP2, TP53INP1, VEGFA	36	yes
Connective Tissue Development and Function	cell movement of fibroblast cell lines	2.45E-06	Decreased	-2.308	ABL1, ACP1, ACTR2, AKT1, AMD1, CD44, CDKN1B, CSK, CTNNB1, DDR1, DDX3X, ELK3, FBXW7, FGF2, FN1, FOSL1, GNG12, GRB2, HAS2, HAS3, HBEGF, IL8, ITGA5, KLF2, LIMA1, MARCKS, MST1, MYLK, PTEN, PTGS2, PTPN1, PVR, RASSF1, SKP2, TP53INP1, VEGFA	36	yes
Cell Cycle	re-entry into interphase	3.43E-06	Decreased	-2.442	CCND1, CCND3, CCNE1, CDC25A, CDC25C, CDKN1B, CEBPB, FN1, ID2, MAP2K1, RUNX1, SKP2, TFDP1, TGFA, VEGFA	15	yes
Cellular Development	proliferation of fibroblasts	4.59E-06	Decreased	-2.018	ABL1, ACTB, AHR, AKT1, ATF3, ATF4, ATM, CBX2, CBX7, CCND1, CCND3, CCNE1, CD44, CDK6, CDKN1B, CEBPB, CIRBP, CITED2, CTNNB1, DNM1L, DUSP1, FEN1, FGF2, FN1, FNDC3B, FOSL1, GADD45A, GRB2, GRN, HBEGF, HIF1A, HMMR, ICMT, IFNAR1, IGFBP3, IGFBP4, IL1B, IL6, IP6K2, ITGA5, JUND, KRAS, LDHA, LIF, MAP2K3, MCM8, MET, PA2G4, PHC2, PLAT, PPARD, PPAT, PSME3, PTEN, SAFB, SERTAD1, SKP2, SMAD3, SPHK1, SPRY2, STARD10, TGFBR2, TGIF1, TNFRSF1A, TPR, TXNIP	66	yes
Cellular Growth and Proliferation	proliferation of fibroblasts	4.59E-06	Decreased	-2.018	ABL1, ACTB, AHR, AKT1, ATF3, ATF4, ATM, CBX2, CBX7, CCND1, CCND3, CCNE1, CD44, CDK6, CDKN1B, CEBPB, CIRBP, CITED2, CTNNB1, DNM1L, DUSP1, FEN1, FGF2, FN1, FNDC3B, FOSL1, GADD45A, GRB2, GRN, HBEGF, HIF1A, HMMR, ICMT, IFNAR1, IGFBP3, IGFBP4, IL1B, IL6, IP6K2, ITGA5, JUND, KRAS, LDHA, LIF, MAP2K3, MCM8, MET, PA2G4, PHC2, PLAT, PPARD, PPAT, PSME3, PTEN, SAFB, SERTAD1, SKP2, SMAD3, SPHK1, SPRY2, STARD10, TGFBR2, TGIF1, TNFRSF1A, TPR, TXNIP	66	yes
Connective Tissue Development and Function	proliferation of fibroblasts	4.59E-06	Decreased	-2.018	ABL1, ACTB, AHR, AKT1, ATF3, ATF4, ATM, CBX2, CBX7, CCND1, CCND3, CCNE1, CD44, CDK6, CDKN1B, CEBPB, CIRBP, CITED2, CTNNB1, DNM1L, DUSP1, FEN1, FGF2, FN1, FNDC3B, FOSL1, GADD45A, GRB2, GRN, HBEGF, HIF1A, HMMR, ICMT, IFNAR1, IGFBP3, IGFBP4, IL1B, IL6, IP6K2, ITGA5, JUND, KRAS, LDHA, LIF, MAP2K3, MCM8, MET, PA2G4, PHC2, PLAT, PPARD, PPAT, PSME3, PTEN, SAFB, SERTAD1, SKP2, SMAD3, SPHK1, SPRY2, STARD10, TGFBR2, TGIF1, TNFRSF1A, TPR, TXNIP	66	yes
Cell Cycle	re-entry into S phase	6.05E-06	Decreased	-2.442	CCND1, CCND3, CCNE1, CDC25A, CDKN1B, CEBPB, FN1, ID2, MAP2K1, RUNX1, SKP2, TFDP1, TGFA, VEGFA	14	yes
Cellular Movement	migration of fibroblast cell lines	1.22E-05	Decreased	-2.300	ABL1, ACP1, CDKN1B, CSK, CTNNB1, DDR1, DDX3X, ELK3, FBXW7, FGF2, FN1, GNG12, HAS2, HAS3, HBEGF, IL8, ITGA5, KLF2, LIMA1, MARCKS, MYLK, PTEN, PTGS2, PVR, RASSF1, SKP2, TP53INP1, VEGFA	28	yes
Connective Tissue Development and Function	migration of fibroblast cell lines	1.22E-05	Decreased	-2.300	ABL1, ACP1, CDKN1B, CSK, CTNNB1, DDR1, DDX3X, ELK3, FBXW7, FGF2, FN1, GNG12, HAS2, HAS3, HBEGF, IL8, ITGA5, KLF2, LIMA1, MARCKS, MYLK, PTEN, PTGS2, PVR, RASSF1, SKP2, TP53INP1, VEGFA	28	yes
Cancer	tumorigenesis of malignant tumor	1.25E-05	Decreased	-2.565	AHR, AKT1, ANK3, ARID4B, ASXL2, ATM, BIRC5, BMF, CABLES1, CBX7, CCND1, CCND3, CCNDBP1, CDKN1B, CDKN2C, CHD9, CTLA4, DCBLD2, DDB2, DTNA, EGR1, ERRFI1, FBXO4, FEN1, FGF2, FNDC3B, GADD45A, GDF15, GRB2, ID1, IFNAR1, IFNAR2, IGFBP3, IL1B, IL6, ING2, KRAS, LTBR, MCL1, MET, MUC1, MXI1, NOTCH1, NQO1, ODC1, PPARD, PSME3, PTEN, PTGS2, PTPN1, PTPRE, RASSF1, RUNX1, SMAD3, SPHK1, STAT2, TGFBR2, THBS1, TPR, VEGFA, ZEB2, ZNF148	62	yes
Cancer	tumorigenesis of epithelial tumor	4.86E-05	Decreased	-2.022	AHR, AKT1, ATF5, BBC3, BIRC5, CABLES1, CBX7, CCND1, CCND3, CCNDBP1, CDKN1B, CDKN2C, CTLA4, DDB2, EGR1, ENG, ERRFI1, FBXO4, FOXO3, GADD45A, GDF15, GRB2, IGFBP3, IL6, KRAS, LTBR, MAP2K1, MET, MUC1, MXI1, NOTCH1, NQO1, ODC1, PPARD, PSME3, PTEN, PTGS2, PTPRE, RASSF1, SMAD3, SPHK1, TGFBR2, THBS1, THBS2, TNFRSF1A, TPR, VEGFA	47	yes
Gene Expression	binding of DNA	6.22E-05	Decreased	-2.123	ABL1, ADRB2, AHR, AKT1, ALYREF, APEX1, ATM, BMPR1A, BTAF1, BTK, CALM1 (includes others), CBFB, CCND1, CEBPB, CEBPD, CHUK, CTLA4, DDB2, DDIT3, DMAP1, EGR1, EPAS1, EPOR, ETS1, ETS2, FGF13, FGF2, FN1, FOSL1, FOXO3, FYB, GHR, HBEGF, HEY1, HEY2, HIF1A, HIPK2, HMGB2, HSPA1A/HSPA1B, ID1, ID2, IER3, IFNAR1, IGFBP3, IL1B, IL6, IL8, ING4, IP6K2, IRF9, ITGA5, JUND, JUP, KLF13, KLHDC2, KRAS, LIF, LPL, LTBR, LYN, MAP2K1, NCL, NFIC, NFKBIE, NKX2-5, NME1, NOTCH1, NR5A2, PAX3, PHB, PNPT1, POLG, PPARD, PPARGC1A, PSME3, PTEN, PTPN1, RASSF1, RGCC, RUNX1, RXRB, SMAD3, SP1, SP100, TFAP2A, TFDP1, TFF3, TGFA, TNFRSF1A, TRIB3, TSC22D3, UBE2N, UCN, UCN2, VEGFA, YWHAB, YWHAG	97	no
Cellular Growth and Proliferation	proliferation of connective tissue cells	8.24E-05	Decreased	-2.923	ABL1, ACTB, AHR, AKAP12, AKT1, ATF3, ATF4, ATM, BMPR1A, CBX2, CBX7, CCND1, CCND3, CCNE1, CCNG2, CD44, CDK6, CDKN1B, CEBPB, CHUK, CIRBP, CITED2, CSK, CTNNB1, CTSL2, DNM1L, DUSP1, EDNRB, EFNA1, FEN1, FGF13, FGF2, FGFR3, FN1, FNDC3B, FOSL1, GADD45A, GHR, GRB2, GRN, HBEGF, HIF1A, HMMR, HSPA1A/HSPA1B, HTR2B, ICMT, ID2, IFNAR1, IGFBP3, IGFBP4, IGFBP5, IL1B, IL6, IL8, IP6K2, ITGA5, JUND, JUP, KLF10, KRAS, LDHA, LIF, LIMA1, LRP5, MAP2K1, MAP2K3, MAP3K5, MCM8, MET, NOTCH1, NOTCH2, PA2G4, PHC2, PLAT, PPARD, PPAT, PSME3, PTEN, PTGS2, PTPN1, RAPGEF1, RUNX1, SAFB, SERTAD1, SKP2, SMAD3, SMAD6, SNAI2, SPHK1, SPRY2, STARD10, STC1, TGFA, TGFBR2, TGIF1, TNFRSF12A, TNFRSF1A, TPR, TXNIP, UBE2C	100	yes
Cancer	tumorigenesis of mammary tumor	1.04E-04	Decreased	-2.318	AKT1, CCND1, CDC25B, CHUK, CTLA4, CTNNB1, FBXO4, GADD45A, KRAS, MET, MUC1, PTEN, PTGS2, PTPN1, PTPRE, TGFA, TGFBR2, THBS1	18	yes
Reproductive System Disease	tumorigenesis of mammary tumor	1.04E-04	Decreased	-2.318	AKT1, CCND1, CDC25B, CHUK, CTLA4, CTNNB1, FBXO4, GADD45A, KRAS, MET, MUC1, PTEN, PTGS2, PTPN1, PTPRE, TGFA, TGFBR2, THBS1	18	yes
Cancer	growth of mammary tumor	1.54E-04	Decreased	-2.178	ABL1, AKT1, ATM, CTLA4, GADD45A, HIF1A, IGFBP5, IL12A, KRAS, PTEN, PTGS2	11	yes
Gene Expression	binding of protein binding site	1.54E-04	Decreased	-2.635	AHR, AKT1, APEX1, ATM, BMPR1A, BTK, CBFB, CCND1, CEBPB, CEBPD, CHUK, DDB2, DDIT3, EGR1, EPAS1, ETS1, ETS2, FGF2, FN1, FOXO3, GHR, HBEGF, HEY1, HEY2, HSPA1A/HSPA1B, ID1, ID2, IER3, IGFBP3, IL1B, IL6, IL8, ING4, IP6K2, JUP, KLF13, KRAS, LIF, LTBR, MAP2K1, NFIC, NOTCH1, NR5A2, PNPT1, PTEN, RGCC, RUNX1, SMAD3, SP1, SP100, TFAP2A, TFDP1, TFF3, TGFA, TNFRSF1A, TRIB3, VEGFA	57	no
Cancer	quantity of tumor	1.91E-04	Decreased	-2.081	AKT1, CCND1, CDKN1B, CDKN2C, CTNNB1, EDNRB, GJB1, HIF1A, HMMR, HPGD, IFNGR1, KIT, KLF6, KRAS, MAP2K1, MET, MST1, NQO1, ODC1, PPARD, PTEN, RUNX1, SPHK1, STK3, TGFA, TGFBR2, TP53INP1	27	no
Cancer	tumorigenesis of carcinoma	2.50E-04	Decreased	-2.758	AHR, AKT1, BIRC5, CABLES1, CBX7, CCND3, CCNDBP1, CDKN1B, CDKN2C, CTLA4, DDB2, EGR1, ERRFI1, FBXO4, GADD45A, GDF15, GRB2, IGFBP3, KRAS, LTBR, MET, MUC1, MXI1, NOTCH1, NQO1, ODC1, PPARD, PSME3, PTEN, PTGS2, PTPRE, RASSF1, SMAD3, SPHK1, TGFBR2, THBS1, TPR, VEGFA	38	yes
Developmental Disorder	runting	4.15E-04	Increased	2.236	IL6, INSIG1, INSIG2, NFIC, NLRP3, PTRH2	6	no
Cellular Movement	movement of vascular endothelial cells	5.77E-04	Decreased	-2.821	ADAMTS1, ADM, AKT1, ANXA2, CD151, CDKN1B, CYR61, EFNA1, FGF13, FGF2, FN1, FOXO3, HIF1A, HLX, HSPA5, ID1, IL8, KLF2, NRP1, PTEN, PTGS2, RTN4, SP100, SPRY4, TGFBR2, THBS1, THBS2, VEGFA	28	yes
Cardiovascular System Development and Function	movement of vascular endothelial cells	5.77E-04	Decreased	-2.821	ADAMTS1, ADM, AKT1, ANXA2, CD151, CDKN1B, CYR61, EFNA1, FGF13, FGF2, FN1, FOXO3, HIF1A, HLX, HSPA5, ID1, IL8, KLF2, NRP1, PTEN, PTGS2, RTN4, SP100, SPRY4, TGFBR2, THBS1, THBS2, VEGFA	28	yes
Cellular Movement	cell movement of endothelial cells	6.63E-04	Decreased	-2.815	ACP1, ADAMTS1, ADM, AKT1, ANGPTL4, ANXA2, ARHGAP24, CD151, CD36, CDKN1B, CEACAM1, CSPG4, CXCR4, CYR61, EFNA1, ENG, ENPP2, FGF13, FGF2, FN1, FOXO3, GRN, HAS3, HEY1, HIF1A, HLX, HMMR, HSPA5, ID1, IGFBP3, IL1B, IL8, ITGA3, ITGA4, KLF2, MAP2K1, MAP2K3, MAP2K5, MARCKS, NRP1, PIM1, PRKCZ, PTEN, PTGS2, RGCC, RTN4, SDC4, SP100, SPRY4, STC1, TGFBR2, THBS1, THBS2, TIMP3, TNFRSF12A, VEGFA, WARS	57	yes

Cardiovascular System Development and Function	cell movement of endothelial cells	6.63E-04	Decreased	-2.815	ACP1, ADAMTS1, ADM, AKT1, ANGPTL4, ANXA2, ARHGAP24, CD151, CD36, CDKN1B, CEACAM1, CSPG4, CXCR4, CYR61, EFNA1, ENG, ENPP2, FGF13, FGF2, FN1, FOXO3, GRN, HAS3, HEY1, HIF1A, HLX, HMMR, HSPA5, ID1, IGFBP3, IL1B, IL8, ITGA3, ITGA4, KLF2, MAP2K1, MAP2K3, MAP2K5, MARCKS, NRP1, PIM1, PRKCZ, PTEN, PTGS2, RGCC, RTN4, SDC4, SP100, SPRY4, STC1, TGFBR2, THBS1, THBS2, TIMP3, TNFRSF12A, VEGFA, WARS	57	yes
Cell Cycle	cell cycle progression of tumor cell lines	7.70E-04	Decreased	-2.320	ABL1, AHR, AKT1, APBB3, BCL6, BIRC5, CCND1, CCND3, CCNE1, CCNG2, CD44, CDC25B, CDC25C, CDK6, CDKN1B, CDKN2C, CEBPB, CHUK, CSNK2A2, DIABLO, DUSP1, EGR1, FOXO3, FRMD6, HBEGF, HIF1A, HPGD, IER3, IGFBP3, IL6, KRAS, LIF, LYN, MAFB, NOTCH1, PHB, PTEN, RASSF1, SLFN11, SRSF2, XBP1	41	yes
Post-Translational Modification	activation of protein	8.42E-04	Decreased	-2.195	ADM, AKT1, AMD1, BBC3, BID, CCNE1, FABP5, FGF2, IGFBP3, IL1B, IL6, KIT, LIF, ODC1, PMAIP1, PTEN, SPHK1, VEGFA	18	no
Cancer	mammary tumor	8.79E-04	Decreased	-2.430	ABL1, ACTA2, ADAMTS1, AGPAT6, AKAP12, AKT1, ALDH1A3, ALYREF, ANAPC13, ANG, ARHGAP19, ARHGDIA, ARHGEF2, ARNTL, ARRB1, ATF3, ATM, BEX1, BEX2, BHLHE40, BIRC5, C14orf159, C1QBP, CALM1 (includes others), CCNB2, CCND1, CCNE1, CCNE2, CD44, CDC25B, CDKN1B, CDT1, CEBPB, CEBPD, CENPA, CHD7, CHUK, CLDN1, CLTCL1, COL4A3BP, COPS2, CTDSPL, CTLA4, CTNNB1, CYR61, DDIT4, DUSP1, EGLN1, EGR1, EIF1, EIF5, ELOVL6, ENG, EPOR, ERI2, FAM107B, FAM198B, FAM49B, FAM72D, FASN, FBXO4, FBXW4, FBXW7, FEN1, FN1, FOXO3, FSTL3, FURIN, GADD45A, GRN, H3F3A/H3F3B, HBEGF, HBP1, HEY2, HIF1A, HK2, HMMR, HNRNPA1, HSPA5, HSPB8, IDH3A, IER3, IGFBP3, IGFBP5, IL12A, IL6, ING4, JUND, KIT, KLF10, KLF11, KLF6, KRAS, LETMD1, LIMA1, MCL1, MCM10, MET, MKKS, MT1F, MT1G, MT2A, MUC1, MUM1, MYBBP1A, MYCBP2, MYLK, NEK2, NME1, NOL12, NOP16, NOTCH1, NOTCH2, NQO1, NUP93, ODC1, PAK1IP1, PDK4, PHB, PKD1L2, PMAIP1, PPFIA1, PRAME, PRIM1, PSMD12, PTEN, PTGS2, PTPN1, PTPRE, QKI, RAD23B, RANBP1, RASSF1, RBM4B, RGS2, RPL6, RPS24, RRP12, RUNX1, SAFB2, SCHIP1, SDC1, SIDT2, SKP2, SLC27A1, SLC2A1, SLC9A3R1, SMAD3, SNAI2, SQLE, SRP19, SRPK1, SSPN, SV2A, TAF1D, TGFA, TGFBR2, THBS1, TPD52, TUBB2A, TUBB4B, TUBG1, UBE2C, UBE2G2, UHRF1, VEGFA, WWTR1, XRCC3, YPEL5, ZBTB20, ZNF318, ZNF703, ZYX	173	yes
Cell Death and Survival	neuronal cell death	9.93E-04	Decreased	-2.015	ABL1, ADRBK1, AGRN, AKT1, ATF3, ATM, ATXN3, BAG5, BBC3, BID, BIRC5, BNIP3, CD200, CDC25A, CDK6, CDKN1B, CHUK, CTNNB1, CTSL2, DDIT3, DIABLO, DNAJB2, DUSP1, DUSP6, E2F3, E2F4, EGLN1, EGR1, EHD4, EPOR, ETS2, FAIM, FBXW7, FGF2, FN1, FOXO3, FZD2, GAB2, GCLM, GDF15, HBEGF, HIF1A, HIPK2, HK2, HMGB2, HRK, HSPA5, HSPH1, ID2, IDE, IGFBP3, IL1B, IL6, IL8, ITGA5, KLF6, KRAS, LDHA, LIF, MAP2K1, MAP2K3, MAP3K12, MAP3K5, MBP, MEF2C, MEF2D, MET, MXD3, NCS1, NQO1, NRTN, P2RX7, PEX11B, PHB, PLAT, PLP1, PPARGC1A, PRNP, PSME3, PTEN, PTGS2, RASD2, RGS10, RNF19A, RUNX1, SH3RF1, SIAH1, SLC2A1, SOX4, SP1, SRPK2, ST8SIA1, STAM, STAMBP, TFB1M, TFDP1, TGFA, TMEM158, TNFRSF1A, TNFRSF21, TRIM2, UBE2V2, UCN, VEGFA, WFS1, YWHAB	106	no
Infectious Disease	Viral Infection	1.45E-03	Decreased	-2.495	ABCE1, ABL1, ABTB1, ACTB, ACTN1, ACTR2, ADRBK1, AFG3L1P, AHCTF1, AHR, AKT1, ALKBH8, ANXA2, AP1B1, APOBEC3F, ARNTL, ARRB1, ASXL2, ATF4, ATM, ATP6V0A4, BBC3, BCL9, BICD2, C15orf52, C1R, C1S, C20orf24, C3AR1, C4orf33, C6orf48, CALCOCO1, CAPN3, CBLB, CD200, CD44, CDKN1B, CEBPD, CHD1, CHMP1B, CHORDC1, CHST6, CHUK, CLTCL1, CNST, COASY, CTSL2, CXCR4, CYP2U1, DCBLD1, DCLK1, DDIT3, DDX3X, DHRS2, DHX15, DHX33, DHX58, DMAP1, DNAJA1, DNAJB2, DTX3, DUSP1, EGR1, EIF2C2, EIF2S1, EIF4A3, ENC1, EPAS1, EPOR, EPS15, ERI2, ETS2, EXOSC10, EXOSC3, F12, FN1, FOXF2, FURIN, FXR1, G3BP1, G3BP2, GAB2, GABARAPL1, GHR, GPT2, GRB2, GRK5, H3F3A/H3F3B, HIST1H2AC, HIST1H2BD, HIST2H2AA3/HIST2H2AA4, HLX, HMGCS1, HMGN2, HNRPDL, HPGD, HSPA5, HSPA6, HYAL2, IER3, IFI35, IFIT2, IFNAR1, IFNAR2, IFNGR1, IL12A, IL1B, IL6, IL8, IMPDH1, IRF4, IRF9, IRS2, ISG20, ISG20L2, ITGA3, ITGA4, ITGA5, ITK, ITPKA, KLF2, KLHDC2, LAMP2, LEF1, LIMK1, LPL, MAP2K1, MAP2K3, MAP2K5, MAP3K12, MAT2A, MED31, MGAT1, MGLL, MND1, MPHOSPH6, MR1, MRPL44, MT2A, MYOF, NCL, NOP56, NRP1, NTHL1, NUP155, OPTN, OSBPL6, OTUD1, P2RX7, PACSIN3, PARP9, PDE3B, PDE7B, PDXK, PDZD8, PGRMC2, PIK3CB, PKD1L2, PLAT, PMM1, POLG, POU3F1, PPARGC1A, PPP1R15A, PPP2R2A, PPP2R5E, PPP3R1, PRDM7, PRMT3, PRNP, PRSS35, PSMD12, PSMD4, PTGS2, PVR, RAB32, RAB5C, RAB9A, RABEPK, RACGAP1, RALB, RANBP1, RAPGEF1, RASSF1, RBM25, RNF144A, RNF150, RTN2, RUNX1, SAFB, SDC1, SEC14L1, SEC61G, SH2B3, SIGMAR1, SLC2A1, SLC2A3, SLC4A7, SLC9A3R1, SMAD3, SNRPA, SNRPA1, SNRPD3, SNRPF, SNX10, SNX6, SON, SOX4, SP100, SPAST, SPCS3, SPEN, SPHK1, SPRY2, SRPK1, SRPK2, SRSF2, SSR1, ST3GAL5, ST6GAL1, STAG3L1, STAT2, STIP1, SYNJ1, TCFL5, TFDP2, TFRC, TGFBR2, THOC2, TIAM2, TMC6, TMEM181, TNFRSF1A, TRAPPC8, TRMT6, TRPV2, TUBB2A, TUBB4B, TUBG1, TXNIP, UBE2C, ULK1, VEGFA, VWF, WIPF1, WWP2, XBP1, ZCCHC17, ZEB2, ZNF148, ZNF446, ZNF536, ZNF688, ZYX	254	yes
DNA Replication, Recombination, and Repair	quantity of centrosome	1.67E-03	Decreased	-2.491	CDC25B, CHMP1B, GADD45A, ID1, KIF23, LATS2, LMO4, MKS1, RASSF1, SKP2, TFDP1, TMEM67	12	yes
Cellular Assembly and Organization	quantity of centrosome	1.67E-03	Decreased	-2.491	CDC25B, CHMP1B, GADD45A, ID1, KIF23, LATS2, LMO4, MKS1, RASSF1, SKP2, TFDP1, TMEM67	12	no
Cellular Development	proliferation of ovarian cancer cell lines	1.79E-03	Decreased	-2.143	AKT1, ARHGEF2, ATF3, CCND1, CD44, CXCR4, DAB2, E2F3, FOSL1, FOXO3, GDF15, GRN, HAS2, HAS3, HBEGF, IL6, KLF2, NUMA1, SERTAD1, SKP2, TGFA, TGFBR2	22	yes
Cellular Growth and Proliferation	proliferation of ovarian cancer cell lines	1.79E-03	Decreased	-2.143	AKT1, ARHGEF2, ATF3, CCND1, CD44, CXCR4, DAB2, E2F3, FOSL1, FOXO3, GDF15, GRN, HAS2, HAS3, HBEGF, IL6, KLF2, NUMA1, SERTAD1, SKP2, TGFA, TGFBR2	22	yes
Developmental Disorder	hypoplasia of organ	1.80E-03	Increased	2.376	ABL1, ADRBK1, AHR, AKT1, ALDH1A3, ARHGDIA, ARID5B, ARRB1, ATF4, BAG3, BCL6, CBY1, CCND3, CCNE1, CCNE2, CDK6, CDKN1B, CTLA4, DHCR7, E2F3, E2F4, ECE2, EPOR, ETS1, FGF2, FGFRL1, GAS1, HBEGF, HLX, IFNGR1, ITGA3, KRAS, LIF, LYN, MAFF, MAP2K1, MET, NFAT5, NKX2-5, NKX3-1, NKX3-2, NOTCH2, PKNOX1, PRRX1, PSMC3IP, RASSF2, RDH10, RUNX1, SLC20A1, SMAD3, SOX4, SPEN, TDG, VEGFA, WIPF1, XBP1	56	no
Cancer	bone marrow cancer	2.53E-03	Increased	2.200	ABL1, ACTG1, AHI1, AKT1, ARID4B, ARID5B, ATM, BCL6, BCOR, BIRC5, C12orf5, CBFB, CCND1, CDC25A, CHUK, CTNNB1, DLEU1, DLEU2, DNM1L, E2F3, E2F4, ENC1, EPOR, FAIM3, FBXL2, FBXW7, FOXO3, GHR, HLX, HSPA1A/HSPA1B, ID4, IFNAR1, IFNAR2, IL1B, IL6, IL8, IMPDH1, IRF4, ITGA3, ITGA4, ITGA5, KIT, KRAS, LDHA, LYN, MSMO1, NOTCH1, NQO1, PDE3B, PDE7B, PDE9A, PPAT, PRIM1, PTEN, RBM15, RPL6, RPS24, RUNX1, RXRB, SNRPD3, SOX4, SRSF2, TUBB2A, TUBB4B, TUBG1, U2AF1, XRCC3	67	no
Hematological Disease	bone marrow cancer	2.53E-03	Increased	2.200	ABL1, ACTG1, AHI1, AKT1, ARID4B, ARID5B, ATM, BCL6, BCOR, BIRC5, C12orf5, CBFB, CCND1, CDC25A, CHUK, CTNNB1, DLEU1, DLEU2, DNM1L, E2F3, E2F4, ENC1, EPOR, FAIM3, FBXL2, FBXW7, FOXO3, GHR, HLX, HSPA1A/HSPA1B, ID4, IFNAR1, IFNAR2, IL1B, IL6, IL8, IMPDH1, IRF4, ITGA3, ITGA4, ITGA5, KIT, KRAS, LDHA, LYN, MSMO1, NOTCH1, NQO1, PDE3B, PDE7B, PDE9A, PPAT, PRIM1, PTEN, RBM15, RPL6, RPS24, RUNX1, RXRB, SNRPD3, SOX4, SRSF2, TUBB2A, TUBB4B, TUBG1, U2AF1, XRCC3	67	no
Cell Cycle	S phase of fibroblast cell lines	2.56E-03	Decreased	-3.130	ABL1, BID, CCND1, CCNE1, CDC25A, CDKN1B, DDX3X, E2F3, E2F4, GADD45A, GAS1, SKP2, TFDP1	13	yes
Connective Tissue Development and Function	S phase of fibroblast cell lines	2.56E-03	Decreased	-3.130	ABL1, BID, CCND1, CCNE1, CDC25A, CDKN1B, DDX3X, E2F3, E2F4, GADD45A, GAS1, SKP2, TFDP1	13	yes
Gene Expression	expression of gene	2.69E-03	Increased	2.032	ANK3, ARID4B, CD44, CITED1, CITED2, CTNNB1, FGF2, HINFP, ID2, IL1B, IL6, ITGA3, KIT, LEF1, MEF2C, MET, NKX2-5, PLP1, PLSCR1, POU3F1, QKI, RBM4B, SPRY2, SRPK2, TFAP2A, TLE1, VEGFA	27	no
Cellular Movement	migration of endothelial cells	2.72E-03	Decreased	-2.282	ACP1, ADAMTS1, ADM, AKT1, ANGPTL4, ANXA2, ARHGAP24, CD151, CDKN1B, CYR61, EFNA1, ENG, FGF13, FGF2, FN1, FOXO3, GRN, HAS3, HEY1, HIF1A, HLX, HMMR, HSPA5, ID1, IGFBP3, IL1B, IL8, ITGA3, ITGA4, KLF2, MAP2K1, MAP2K3, MARCKS, NRP1, PIM1, PRKCZ, PTEN, PTGS2, RGCC, RTN4, SDC4, SP100, SPRY4, STC1, TGFBR2, THBS1, TIMP3, TNFRSF12A, VEGFA, WARS	50	yes
Cardiovascular System Development and Function	migration of endothelial cells	2.72E-03	Decreased	-2.282	ACP1, ADAMTS1, ADM, AKT1, ANGPTL4, ANXA2, ARHGAP24, CD151, CDKN1B, CYR61, EFNA1, ENG, FGF13, FGF2, FN1, FOXO3, GRN, HAS3, HEY1, HIF1A, HLX, HMMR, HSPA5, ID1, IGFBP3, IL1B, IL8, ITGA3, ITGA4, KLF2, MAP2K1, MAP2K3, MARCKS, NRP1, PIM1, PRKCZ, PTEN, PTGS2, RGCC, RTN4, SDC4, SP100, SPRY4, STC1, TGFBR2, THBS1, TIMP3, TNFRSF12A, VEGFA, WARS	50	yes
Cellular Growth and Proliferation	proliferation of epithelial cells	2.80E-03	Decreased	-3.131	AKT1, ASIP, BMPR1A, CCND1, CCND3, CCNG2, CDC25A, CDC25B, CDK6, CDKN1B, CDKN2C, CEBPB, CHUK, CTNNB1, CTSL2, DLX5, EDNRB, EFNA1, FGF2, FGFR3, FN1, FOXF2, GAB2, GAS1, GDF15, GRN, HBEGF, ID1, ID2, IGFBP4, IL1B, IL6, IL8, ITGA3, JUP, KIT, KLF10, KRAS, LTBR, MAP2K1, MAP2K5, MAP3K5, MST1, NFIB, NKX3-1, NME1, NOTCH1, PAX3, POU3F2, PPARD, PTEN, PTGS2, PTK6, RRAS, RUNX1, SKP2, SMAD3, SNAI2, SPHK1, STMN1, TGFA, TGFBR2, TNFRSF1A, VEGFA	64	yes
Cancer	hyperplasia of breast	2.97E-03	Decreased	-2.170	CCND1, CDC25B, CDKN2C, CEBPB, CSNK2A2, CTNNB1, ING4, PTGS2, PTPRE, SDC1, TGFA	11	yes
Cell Cycle	entry into interphase of fibroblast cell lines	3.33E-03	Decreased	-2.449	ABL1, AKT1, CCND1, CDC25A, DDX3X, E2F3, GAS1, SKP2	8	yes
Cancer	hyperplasia of exocrine gland	3.33E-03	Decreased	-2.135	CCND1, CDKN2C, CSNK2A2, CTNNB1, CTSL2, PTGS2, PTPRE, SDC1	8	no

Table S4. **Downstream effect analysis by IPA software of genes significantly modulated only by the association of TRAIL with AZD6244**

Results shown here are based on the set of genes identified by the areas highlighted in fuchsia in **Fig. S8A.**
See supplementary methods for the meaning of the p values and of the z score statistics.

Category	Functions Annotation	p-Value	Predicted Activation	Activation z-score	Molecules	# Molecules	Data shown in Fig. S5b
Cell Death and Survival	apoptosis of carcinoma cell lines	1.58E-04	Increased	2.907	ABCC1, AKT3, BAP1, BAX, BCL2, BCL2L11, BIRC5, CASP1, CCND1, CDKN1B, CDKN2C, DDIT3, DUSP1, ETS2, FADD, FAS, FBXL2, FGF2, FOXO3, HIF1A, HMGA1, ID3, IGFBP3, ING1, KIAA1967, KLF2, KRAS, KRT18, MAP3K7, MCL1, NFKBIA, PMAIP1, PMEPA1, PRKAR1A, PTGS2, RABGGTB, RARB, RBM4B, REPS2, RRM1, SPP1, SPRY2, SRC, TLR4, TNFSF10	45	yes
Cell Death and Survival	cell death of carcinoma cell lines	6.35E-05	Increased	2.396	ABCC1, AKT3, BAP1, BAX, BCL2, BCL2L11, BIRC5, CASP1, CCND1, CDKN1B, CDKN2C, CFH, DDIT3, DUSP1, ETS2, FADD, FAS, FBXL2, FGF2, FOXO3, GAB1, HIF1A, HK1, HMGA1, ID3, IGFBP3, ING1, JUN, KIAA1967, KLF2, KRAS, KRT18, MAP3K5, MAP3K7, MCL1, MYC, NEK2, NFKBIA, NQO1, PMAIP1, PMEPA1, PRKAR1A, PTGS2, RABGGTB, RARB, RBM4B, REPS2, RRM1, SPP1, SPRY2, SRC, TLR4, TNFSF10, TUBB3	54	yes
Cell Death and Survival	apoptosis of sarcoma cells	4.03E-04	Increased	2.308	ABL1, BAX, BCL2, BCL2L11, BTK, CASP1, CDK6, CDKN1B, CTLA4, FAS, FBXL2, HIF1A, KDR, LPAR1, NFKBIA, PIK3CD, PNP, PTEN, RARA, RUNX1, TNFSF10, TNFSF13B, TRIB1	23	yes
Tumor Morphology	apoptosis of sarcoma cells	4.03E-04	Increased	2.308	ABL1, BAX, BCL2, BCL2L11, BTK, CASP1, CDK6, CDKN1B, CTLA4, FAS, FBXL2, HIF1A, KDR, LPAR1, NFKBIA, PIK3CD, PNP, PTEN, RARA, RUNX1, TNFSF10, TNFSF13B, TRIB1	23	yes
Cell Death and Survival	apoptosis of leukemia cells	3.28E-04	Increased	2.153	ABL1, BAX, BCL2, BCL2L11, BTK, CASP1, CDK6, CDKN1B, CTLA4, FAS, FBXL2, HIF1A, LPAR1, NFKBIA, PIK3CD, PNP, PTEN, RARA, RUNX1, TNFSF10, TNFSF13B, TRIB1	22	yes
Tumor Morphology	apoptosis of leukemia cells	3.28E-04	Increased	2.153	ABL1, BAX, BCL2, BCL2L11, BTK, CASP1, CDK6, CDKN1B, CTLA4, FAS, FBXL2, HIF1A, LPAR1, NFKBIA, PIK3CD, PNP, PTEN, RARA, RUNX1, TNFSF10, TNFSF13B, TRIB1	22	yes
Cell Death and Survival	cell death of sarcoma cells	9.14E-04	Increased	2.082	ABL1, BAX, BCL2, BCL2L11, BTK, CASP1, CDK6, CDKN1B, CTLA4, FAS, FBXL2, HIF1A, HMOX1, KDR, LPAR1, NFKBIA, PIK3CD, PNP, PTEN, RARA, RUNX1, TNFSF10, TNFSF13B, TRIB1	24	yes
Tumor Morphology	cell death of sarcoma cells	9.14E-04	Increased	2.082	ABL1, BAX, BCL2, BCL2L11, BTK, CASP1, CDK6, CDKN1B, CTLA4, FAS, FBXL2, HIF1A, HMOX1, KDR, LPAR1, NFKBIA, PIK3CD, PNP, PTEN, RARA, RUNX1, TNFSF10, TNFSF13B, TRIB1	24	yes
Organismal Survival	organismal death	4.14E-07	Increased	2.020	ABCA1, ABCC1, ABCC4, ABI1, ABL1, ABL2, ABR, ACACA, ACSS1, ACVR1, ADAM19, ADAMTS1, ADCY3, ADD1, ADK, ADM, ADORA2B, ADRBK1, AFF1, AHR, AIM2, AKAP12, AKT3, ALDH1A3, ALX1, AMACR, AMPH, ANGPT1, ANGPT2, ANGPTL4, ANK3, ANO1, ANP32B, ANTXR2, ANXA1, AP2M1, APH1A, APOE, AQP11, ARAP3, ARID5B, ARNT, ASPA, ATF6, ATM, ATP1B1, ATP2A2, ATP2B1, ATP5J2, ATXN3, AURKB, BAG3, BAP1, BAX, BBS4, BCL2, BCL2L11, BCL3, BDNF, BECN1, BID, BIRC5, BMF, BMI1, BMP1, BMPR1A, BRCA1, C1GALT1, C3AR1, CA14, CADPS2, CALR, CASP1, CAV1, CBL, CBY1, CCDC86, CCNB2, CCND1, CCND2, CCR1, CD200, CD36, CD59, CD74, CDC25A, CDK5, CDK5R1, CDK6, CDKN1A, CDKN1B, CDKN2C, CEBPB, CEBPD, CFB, CFH, CHKA, CITED1, CKS1B, CLCN7, CLDN1, CLIC4, CLN3, CNTNAP1, COL5A2, COMMD1, COPS8, CPB2, CPOX, CR2, CREB3L2, CREB5, CRKL, CSDA, CSRNP1, CTC1, CTGF, CTLA4, CTNNBIP1, CTSF, CXCR4, DAB2, DBI, DCC, DDAH1, DDB2, DEFB4A/DEFB4B, DGCR8, DHCR7, DHX58, DICER1, DISP1, DKC1, DKK1, DLL3, DLX1, DMRT2, DNAJB4, DNAJC5, DNM3, DSP, DUSP1, DVL2, E2F2, E2F3, E2F5, ECE2, EDNRB, EEF1E1, EFNB2, EGLN1, EGR2, EGR3, EHD4, EIF2C2, EIF4A1, ELK3, ENPP2, EOMES, EPAS1, EPOR, ERCC5, ERRFI1, ESPL1, ETS1, ETS2, ETV1, EXTL3, F2R, F2RL1, FABP3, FADD, FAM20C, FANCL, FAS, FASN, FBN2, FBXO45, FCER1G, FEN1, FGF2, FGFR3, FGFRL1, FKBP4, FLI1, FNDC3B, FOS, FOSL1, FOXD3, FOXM1, FOXO1, FOXO3, FST, FSTL1, FTH1, FURIN, FYN, GAB1, GABPB1, GAS1, GATAD2A, GCLM, GHR, GJB1, GLCE, GLI2, GNA12, GNA13, GNAL, GNL3, GRIP1, GSTK1, GSTM1, H2AFX, HAS2, HAX1, HBEGF, HDAC7, HES1, HEXA, HEY1, HEY2, HHAT, HHEX, HIF1A, HIP1, HIPK1, HIPK2, HIST2H2AA3/HIST2H2AA4, HK2, HMBS, HMGA2, HMGCL, HMGCR, HMOX1, HNRNPD, HOXA5, HOXB2, HOXC13, HOXC4, HOXD11, HOXD13, HPGD, HS3ST1, HSP90AB1, HSPB8, HTR7, HTRA2, ICAM1, ID1, ID2, ID3, IDH1, IDS, IFNAR1, IFNAR2, IFT122, IGF2R, IGFBP3, IGFBP5, IL11RA, IL12A, IL1B, IL27RA, IL8, INCENP, ING1, ING4, INSIG2, IP6K2, IRAK1, IRF5, IRF9, IRS2, ITGA4, ITGA6, ITGB3, ITPKB, ITPR2, ITPR3, JAM3, JPH1, JUN, KAT2A, KCNAB1, KCNQ2, KDR, KIAA0586, KIDINS220, KIT, KLF2, KLF4, KLF9, KRAS, KRR1, LAMA2, LAMA4, LAMC1, LAMTOR2, LDB1, LEF1, LHX6, LIF, LIMK1, LIMS1, LMO4, LOX, LPAR1, LPL, LRP5, LRP8, LTBR, LYN, MACF1, MAF, MAFF, MAFG, MAL, MAN2A2, MAP2K1, MAP2K3, MAP2K5, MAP3K1, MAP3K12, MAP3K3, MAP3K7, MAPKAPK5, MARCKS, MARCKSL1, MCL1, MCM10, MCM2, MCOLN1, MED23, MEF2A, MEF2C, MERTK, MESP1, MGAT1, MITF, MMP16, MMP9, MNS1, MNT, MSN, MT1E, MTHFD2, MTHFR, MTSS1, MUT, MXI1, MYB, MYC, NAT1, NDRG1, NFIA, NFIB, NFKBIA, NKX2-5, NLGN2, NLRP3, NOV, NPC1, NQO1, NR2F1, NR3C1, NR4A2, NR5A2, NRCAM, NRP1, NRXN2, NUDT1, NUSAP1, OCRL, OPHN1, OTX1, OVOL2, PAK4, PAPPA, PARP2, PAX3, PAXIP1, PCID2, PCSK1, PDE4B, PELI1, PER2, PES1, PEX11B, PEX2, PEX5, PHB, PHC2, PKDCC, PKNOX1, PLAT, PLCB4, PLCL1, PMS2, PNN, PNPLA8, PNPT1, POU3F1, POU3F2, PPAP2B, PPARGC1A, PPIF, PPIG, PPP1R14C, PPP1R15A, PPP1R3C, PPP1R9A, PRDX1, PRKAR1A, PRKD1, PRMT1, PRNP, PRRX1, PSMC2, PSMD4, PTEN, PTGES, PTGS2, PTK2, PTPN12, PVRL3, RAB27A, RAC2, RAD54B, RAD9A, RAPGEF1, RARA, RARB, RASA3, RASSF2, RBBP8, RBMS1, REV3L, RGMA, RHBDF2, RHEBL1, RIF1, RIMS2, RNASEL, RPL29, RPL6, RTN4, RUNX1, RUNX2, RXRA, RYK, SATB1, SCNN1A, SDC4, SEMA3A, SFRP1, SGCD, SHB, SHISA2, SIAH1, SIK3, SIM2, SLC11A2, SLC17A7, SLC20A1, SLC25A19, SLC25A37, SLC2A1, SLC2A3, SLC2A8, SLC3A2, SLC7A1, SMARCAL1, SMPD1, SMTN, SNAI2, SNAP23, SNX27, SOD2, SOX4, SOX6, SPEN, SPP1, SPRED1, SPRED2, SPRY1, SPRY2, SPRY4, SRC, SSBP2, ST8SIA1, STAM, STARD13, STAT2, STIM1, STIM2, STK4, STXBP1, STXBP5, SV2B, SYK, SYNJ1, TACC3, TAF4, TARBP2, TBX2, TCF4, TCF7L2, TEAD2, TERF1, TFAP2A, TFAP2C, TFB1M, TFDP1, TGFA, TGFBR2, TGFBR3, TGIF1, THBS1, THBS2, TIMP2, TIMP3, TIRAP, TJP2, TK1, TLR4, TMOD1, TNFRSF1A, TNFRSF1B, TNFSF10, TNFSF13B, TP53BP1, TP53INP1, TPH2, TPP2, TRAF3IP2, TRAFD1, TRIM63, TRO, TRPV2, TRRAP, TWIST1, TXNIP, UBD, UBR3, UHRF1, ULK1, UPF2, VAMP2, VASP, VDR, VEGFA, VGF, WASL, XBP1, XPC, ZBTB20, ZEB2, ZFP36, ZFP57, ZNF259	561	yes
Category	Functions Annotation	p-Value	Predicted Activation	Activation z-score	Molecules	# Molecules	Data shown in Fig. S5b
Cellular Movement	migration of connective tissue cells	1.60E-05	Decreased	-4.133	ABL1, ABL2, ANGPT1, APOE, ARID5B, CAV1, CBL, CCL2, CCNA2, CCND1, CDKN1A, CDKN1B, CTGF, CYTH2, F2R, FGF2, FOSL1, GNA12, GNA13, HBEGF, ITGB3, LIMA1, MAP2K1, MAP3K1, MRAS, MYC, PLAUR, PRKAR1A, PRKCE, PTK2, PTPRA, RAC2, RAP2A, RAPGEF1, SCHIP1, SDC4, SEMA3A, SMO, SNX17, SPP1, SRC, STMN1, TGFBR2, THBS1, TIMP2, VASP, ZFAND5, ZYX	48	yes
Cell Cycle	entry into interphase	2.63E-04	Decreased	-3.900	ABL1, ATM, BHLHE40, CCND1, CDC25A, CDC6, CDKN1A, CDKN1B, CDKN2C, CEBPB, CTLA4, E2F2, E2F3, E2F5, EGR1, FGF2, FOS, FOXM1, GAS1, GTF2I, HBEGF, HINFP, HMGA1, HMOX1, ID2, ID3, IL1B, ILKAP, JUN, KLF4, KRAS, MCM10, MEIS2, MYC, PAM16, PTPRA, RUNX2, SOD2, SRC, ST8SIA1, TFDP1, UHRF1, VPS18	43	yes
Cell Cycle	entry into S phase	2.60E-04	Decreased	-3.869	ABL1, ATM, BHLHE40, CCND1, CDC25A, CDC6, CDKN1A, CDKN1B, CDKN2C, CEBPB, CTLA4, E2F2, E2F3, E2F5, FGF2, FOS, FOXM1, GAS1, GTF2I, HBEGF, HINFP, HMGA1, HMOX1, ID2, ID3, IL1B, ILKAP, JUN, KLF4, KRAS, MCM10, MEIS2, MYC, PAM16, SOD2, SRC, ST8SIA1, TFDP1, UHRF1, VPS18	40	yes
Cellular Movement	migration of fibroblasts	3.43E-06	Decreased	-3.750	ABL1, ABL2, ANGPT1, APOE, ARID5B, CAV1, CCL2, CCNA2, CCND1, CDKN1A, CDKN1B, F2R, FGF2, GNA12, GNA13, HBEGF, ITGB3, MAP2K1, MAP3K1, MRAS, MYC, PLAUR, PRKAR1A, PRKCE, PTK2, PTPRA, RAC2, RAP2A, RAPGEF1, SCHIP1, SDC4, SMO, SNX17, SPP1, STMN1, TGFBR2, THBS1, TIMP2, VASP, ZFAND5, ZYX	41	yes
Connective Tissue Development and Function	migration of fibroblasts	3.43E-06	Decreased	-3.750	ABL1, ABL2, ANGPT1, APOE, ARID5B, CAV1, CCL2, CCNA2, CCND1, CDKN1A, CDKN1B, F2R, FGF2, GNA12, GNA13, HBEGF, ITGB3, MAP2K1, MAP3K1, MRAS, MYC, PLAUR, PRKAR1A, PRKCE, PTK2, PTPRA, RAC2, RAP2A, RAPGEF1, SCHIP1, SDC4, SMO, SNX17, SPP1, STMN1, TGFBR2, THBS1, TIMP2, VASP, ZFAND5, ZYX	41	yes
Cellular Movement	cell movement of endothelial cells	2.80E-07	Decreased	-3.603	ADAMTS1, ADM, ANGPT1, ANGPT2, ANGPTL4, ANXA2, APOE, ARHGAP24, ARHGEF6, ARNT, BDNF, CAV1, CCL2, CD36, CDKN1B, CEACAM1, CSPG4, CTGF, CXCR4, DICER1, DKK1, EFNA1, EFNB2, ENPP2, EYA3, F2RL1, FAP, FGF13, FGF2, FOXO1, FOXO3, GAB1, HAS3, HEY1, HHEX, HIF1A, HMMR, HSP90AB1, HSPB1, ICAM1, ID1, ID3, IGF2R, IGFBP3, IL1B, IL8, ITGA4, ITGB3, KDR, KLF2, KLHL20, MAP2K1, MAP2K3, MAP2K5, MAP2K6, MAP3K7, MAPKAPK5, MARCKS, MDK, MMP9, MTSS1, MYC, NOX4, NPTX1, NRP1, PAXIP1, PGF, PLAUR, PLXNA1, PRKCE, PRKD1, PTEN, PTGS2, PTK2, PTPRZ1, RTN4, SCG2, SDC4, SEMA3A, SEMA4D, SP100, SPP1, SPRY4, SRC, STC1, TGFBR2, THBS1, THBS2, TIMP2, TIMP3, TNFRSF12A, TNFSF10, VASP, VEGFA	94	yes
Cardiovascular System Development and Function	cell movement of endothelial cells	2.80E-07	Decreased	-3.603	ADAMTS1, ADM, ANGPT1, ANGPT2, ANGPTL4, ANXA2, APOE, ARHGAP24, ARHGEF6, ARNT, BDNF, CAV1, CCL2, CD36, CDKN1B, CEACAM1, CSPG4, CTGF, CXCR4, DICER1, DKK1, EFNA1, EFNB2, ENPP2, EYA3, F2RL1, FAP, FGF13, FGF2, FOXO1, FOXO3, GAB1, HAS3, HEY1, HHEX, HIF1A, HMMR, HSP90AB1, HSPB1, ICAM1, ID1, ID3, IGF2R, IGFBP3, IL1B, IL8, ITGA4, ITGB3, KDR, KLF2, KLHL20, MAP2K1, MAP2K3, MAP2K5, MAP2K6, MAP3K7, MAPKAPK5, MARCKS, MDK, MMP9, MTSS1, MYC, NOX4, NPTX1, NRP1, PAXIP1, PGF, PLAUR, PLXNA1, PRKCE, PRKD1, PTEN, PTGS2, PTK2, PTPRZ1, RTN4, SCG2, SDC4, SEMA3A, SEMA4D, SP100, SPP1, SPRY4, SRC, STC1, TGFBR2, THBS1, THBS2, TIMP2, TIMP3, TNFRSF12A, TNFSF10, VASP, VEGFA	94	yes
Cellular Movement	chemotaxis of cells	6.97E-04	Decreased	-3.587	ABCC1, ABL1, ADRBK1, ANGPT1, ANGPT2, ANGPTL2, ANXA1, ANXA2, APOE, ARAP3, BDNF, C3AR1, CALR, CASP1, CAV1, CBL, CCL2, CCL20, CCL28, CCND1, CCR1, CD36, CD74, CDKN1A, CEACAM1, CKLF, COL4A3BP, CTGF, CTSE, CXCL13, CXCL16, CXCR4, DEFB4A/DEFB4B, DUSP6, EDNRB, EFNA1, ENPP2, EPHA2, ETV4, F2R, F2RL1, FADD, FAS, FCER1G, FCGR2A, FGF2, FYN, GAB1, GNA12, GNA13, HBEGF, HDAC6, HMGB2, HSPD1, ICAM1, IGF2R, IL16, IL1B, IL8, ITGA4, ITGA6, ITGB3, JAM3, JUN, KDR, KIT, LEF1, LIF, LIMK1, LOX, LYN, MAP3K1, MAP3K5, MDK, MMP9, MPP1, MST1, MYLK, NEDD9, NOV, NPTX1, NRCAM, NRP1, PDE4B, PGF, PIK3CD, PLAUR, PLXNB1, PREX1, PRNP, PTEN, PTGES, PTGS2, PTK2, RAC2, RAP1GAP, RGS1, ROCK1, RTN4, SCG2, SEMA3A, SEMA4D, SPHK1, SPP1, SRC, SYK, TFF3, TGFA, THBS1, THBS2, TIRAP, TLR4, TNFRSF1A, TRAF3IP2, TRIB1, TRPV2, VEGFA, VEGFB, WASL, YARS	120	yes
Cellular Assembly and Organization	formation of cellular protrusions	5.98E-09	Decreased	-3.583	ABI1, ABL1, ABL2, ACACA, ADCY3, AHR, AKAP12, ANGPTL4, ANK3, APOE, ARAP1, ARHGAP24, ARHGAP33, ATF6, ATL1, ATP7A, ATXN1, BAIAP2, BBS4, BCL2, BDNF, BECN1, BSN, BTG3, BTK, CACNA1H, CAV1, CBY1, CCL2, CDC42EP1, CDC42EP4, CDK5, CDK5R1, CDKN1A, CDKN1B, CNTNAP1, CRKL, CTGF, CTLA4, CXCR4, CYFIP1, CYTH2, DCC, DCLK1, DCX, DDAH1, DGKG, DIAPH2, DIAPH3, DICER1, DKK3, DLG4, DNM3, DZIP1L, EFHD1, EFNA1, EFNB2, ENC1, EPHA2, EPHA4, EPS15, ETV4, EVI5L, F2RL1, FEZ1, FGF13, FGF2, FNBP1L, FOS, FYN, GAB1, GDPD5, GNA12, GNA13, HMGB2, HSPB1, HTR7, IFT122, IL11RA, IL1B, IL1RAP, ITGA4, ITGA6, ITGB3, JUN, KBTBD10, KDR, KIAA1598, KIDINS220, KIT, KLF9, LAMB1, LAMC1, LIMK1, LOX, LPAR1, LRP8, LRRC16A, LYN, LZTS1, MAP2K1, MAP3K1, MAP3K7, MARCKS, MATN2, MBP, MKS1, MRAS, MSN, MT2A, MTSS1, MYO10, MYO5A, NCKIPSD, NEO1, NFIA, NFIB, NFKBIA, NPHP3, NRCAM, NRP1, NRTN, ONECUT2, OPHN1, OPTN, P2RX7, PACSIN2, PAK4, PARVB, PCM1, PHLDB2, PI4KB, PLAT, PLXNB1, PMP22, POU3F1, POU3F2, PPP1R9A, PREX1, PRICKLE2, PRKCE, PRMT1, PRNP, PTEN, PTK2, PTPRE, PTPRM, PTPRZ1, PVR, PVRL3, RAB17, RAB3IP, RAC2, RALA, RALB, RANBP1, RAP1GAP, RAP2A, RIN1, RIT1, ROCK1, ROCK2, RTN4, RUFY3, RUNX3, RYK, SDC4, SEMA3A, SEMA4D, SLC11A2, SMPD1, SMPD2, SPHK1, SRC, SRGAP2, ST8SIA1, STMN1, STX3, STXBP5, SYK, TBC1D7, TGIF1, THBS1, TIAM2, TM4SF1, TMEM67, TNC, TNFRSF12A, TNFRSF1A, TRPV4, TTC8, TWIST1, ULK1, VANGL2, VASP, VEGFA, WASL, WIPF1, WISP2, WWTR1	200	yes
Cellular Function and Maintenance	formation of cellular protrusions	5.98E-09	Decreased	-3.583	ABI1, ABL1, ABL2, ACACA, ADCY3, AHR, AKAP12, ANGPTL4, ANK3, APOE, ARAP1, ARHGAP24, ARHGAP33, ATF6, ATL1, ATP7A, ATXN1, BAIAP2, BBS4, BCL2, BDNF, BECN1, BSN, BTG3, BTK, CACNA1H, CAV1, CBY1, CCL2, CDC42EP1, CDC42EP4, CDK5, CDK5R1, CDKN1A, CDKN1B, CNTNAP1, CRKL, CTGF, CTLA4, CXCR4, CYFIP1, CYTH2, DCC, DCLK1, DCX, DDAH1, DGKG, DIAPH2, DIAPH3, DICER1, DKK3, DLG4, DNM3, DZIP1L, EFHD1, EFNA1, EFNB2, ENC1, EPHA2, EPHA4, EPS15, ETV4, EVI5L, F2RL1, FEZ1, FGF13, FGF2, FNBP1L, FOS, FYN, GAB1, GDPD5, GNA12, GNA13, HMGB2, HSPB1, HTR7, IFT122, IL11RA, IL1B, IL1RAP, ITGA4, ITGA6, ITGB3, JUN, KBTBD10, KDR, KIAA1598, KIDINS220, KIT, KLF9, LAMB1, LAMC1, LIMK1, LOX, LPAR1, LRP8, LRRC16A, LYN, LZTS1, MAP2K1, MAP3K1, MAP3K7, MARCKS, MATN2, MBP, MKS1, MRAS, MSN, MT2A, MTSS1, MYO10, MYO5A, NCKIPSD, NEO1, NFIA, NFIB, NFKBIA, NPHP3, NRCAM, NRP1, NRTN, ONECUT2, OPHN1, OPTN, P2RX7, PACSIN2, PAK4, PARVB, PCM1, PHLDB2, PI4KB, PLAT, PLXNB1, PMP22, POU3F1, POU3F2, PPP1R9A, PREX1, PRICKLE2, PRKCE, PRMT1, PRNP, PTEN, PTK2, PTPRE, PTPRM, PTPRZ1, PVR, PVRL3, RAB17, RAB3IP, RAC2, RALA, RALB, RANBP1, RAP1GAP, RAP2A, RIN1, RIT1, ROCK1, ROCK2, RTN4, RUFY3, RUNX3, RYK, SDC4, SEMA3A, SEMA4D, SLC11A2, SMPD1, SMPD2, SPHK1, SRC, SRGAP2, ST8SIA1, STMN1, STX3, STXBP5, SYK, TBC1D7, TGIF1, THBS1, TIAM2, TM4SF1, TMEM67, TNC, TNFRSF12A, TNFRSF1A, TRPV4, TTC8, TWIST1, ULK1, VANGL2, VASP, VEGFA, WASL, WIPF1, WISP2, WWTR1	200	yes
Cell Morphology	formation of cellular protrusions	5.98E-09	Decreased	-3.583	ABI1, ABL1, ABL2, ACACA, ADCY3, AHR, AKAP12, ANGPTL4, ANK3, APOE, ARAP1, ARHGAP24, ARHGAP33, ATF6, ATL1, ATP7A, ATXN1, BAIAP2, BBS4, BCL2, BDNF, BECN1, BSN, BTG3, BTK, CACNA1H, CAV1, CBY1, CCL2, CDC42EP1, CDC42EP4, CDK5, CDK5R1, CDKN1A, CDKN1B, CNTNAP1, CRKL, CTGF, CTLA4, CXCR4, CYFIP1, CYTH2, DCC, DCLK1, DCX, DDAH1, DGKG, DIAPH2, DIAPH3, DICER1, DKK3, DLG4, DNM3, DZIP1L, EFHD1, EFNA1, EFNB2, ENC1, EPHA2, EPHA4, EPS15, ETV4, EVI5L, F2RL1, FEZ1, FGF13, FGF2, FNBP1L, FOS, FYN, GAB1, GDPD5, GNA12, GNA13, HMGB2, HSPB1, HTR7, IFT122, IL11RA, IL1B, IL1RAP, ITGA4, ITGA6, ITGB3, JUN, KBTBD10, KDR, KIAA1598, KIDINS220, KIT, KLF9, LAMB1, LAMC1, LIMK1, LOX, LPAR1, LRP8, LRRC16A, LYN, LZTS1, MAP2K1, MAP3K1, MAP3K7, MARCKS, MATN2, MBP, MKS1, MRAS, MSN, MT2A, MTSS1, MYO10, MYO5A, NCKIPSD, NEO1, NFIA, NFIB, NFKBIA, NPHP3, NRCAM, NRP1, NRTN, ONECUT2, OPHN1, OPTN, P2RX7, PACSIN2, PAK4, PARVB, PCM1, PHLDB2, PI4KB, PLAT, PLXNB1, PMP22, POU3F1, POU3F2, PPP1R9A, PREX1, PRICKLE2, PRKCE, PRMT1, PRNP, PTEN, PTK2, PTPRE, PTPRM, PTPRZ1, PVR, PVRL3, RAB17, RAB3IP, RAC2, RALA, RALB, RANBP1, RAP1GAP, RAP2A, RIN1, RIT1, ROCK1, ROCK2, RTN4, RUFY3, RUNX3, RYK, SDC4, SEMA3A, SEMA4D, SLC11A2, SMPD1, SMPD2, SPHK1, SRC, SRGAP2, ST8SIA1, STMN1, STX3, STXBP5, SYK, TBC1D7, TGIF1, THBS1, TIAM2, TM4SF1, TMEM67, TNC, TNFRSF12A, TNFRSF1A, TRPV4, TTC8, TWIST1, ULK1, VANGL2, VASP, VEGFA, WASL, WIPF1, WISP2, WWTR1	200	yes

Cellular Movement	chemotaxis	6.77E-04	Decreased	-3.578	ABCC1, ABL1, ADRBK1, ANGPT1, ANGPT2, ANGPTL2, ANXA1, ANXA2, APOE, ARAP3, BDNF, C3AR1, CALR, CASP1, CAV1, CBL, CCL2, CCL20, CCL28, CCND1, CCR1, CD36, CD74, CDKN1A, CEACAM1, CKLF, COL4A3BP, CTGF, CTSE, CXCL13, CXCL16, CXCR4, DEFB4A/DEFB4B, DUSP6, EDNRB, EFNA1, EFNB2, ENPP2, EPHA2, ETV4, F2R, F2RL1, FADD, FAS, FCER1G, FCGR2A, FGF2, FOSL1, FYN, GAB1, GNA12, GNA13, HBEGF, HDAC6, HMGB2, HSPD1, ICAM1, IGF2R, IL16, IL1B, IL8, ITGA4, ITGA6, ITGB3, JAM3, JUN, KDR, KIT, LEF1, LIF, LIMK1, LOX, LYN, MAP2K1, MAP3K1, MAP3K5, MDK, MMP9, MPP1, MST1, MYLK, NEDD9, NOV, NPTX1, NRCAM, NRP1, PDE4B, PGF, PIK3CD, PLAUR, PLXNB1, PREX1, PRNP, PTEN, PTGES, PTGS2, PTK2, RAC2, RALA, RAP1GAP, RGS1, ROCK1, RTN4, SCG2, SEMA3A, SEMA4D, SPHK1, SPP1, SRC, SYK, TFF3, TGFA, THBS1, THBS2, TIRAP, TLR4, TNFRSF1A, TRAF3IP2, TRIB1, TRPV2, VEGFA, VEGFB, WASL, YARS	124	yes
Cellular Assembly and Organization	organization of cytoskeleton	3.30E-10	Decreased	-3.532	ABCC1, ABI1, ABL1, ABL2, ABR, ACACA, ADCY3, ADD1, ADM, ADRBK1, AFAP1, AHR, AKAP11, AKAP12, ANGPT2, ANGPTL4, ANK3, APOE, ARAP1, ARAP3, ARHGAP24, ARHGAP33, ARHGEF1, ARHGEF17, ARHGEF2, ATF6, ATL1, ATP2B1, ATP7A, ATXN1, BAG3, BAIAP2, BAX, BBS4, BCL2, BDNF, BECN1, BIRC5, BSN, BTG3, BTK, CACNA1H, CALR, CAV1, CBL, CBY1, CCL2, CD2, CDC25B, CDC42EP1, CDC42EP4, CDK2AP2, CDK5, CDK5R1, CDK5RAP3, CDKN1A, CDKN1B, CEP350, CHAMP1, CHMP1B, CHN2, CKAP4, CLIP3, CNTNAP1, CRIPT, CRKL, CTGF, CTLA4, CXCR4, CYFIP1, CYTH2, DAPK1, DCC, DCLK1, DCX, DDAH1, DGKG, DIAPH2, DIAPH3, DICER1, DKK3, DLG4, DLGAP5, DNM3, DSP, DVL2, DYNC1LI2, DYNLL1, DZIP1L, EFHD1, EFNA1, EFNB2, ENC1, EPHA2, EPHA4, EPS15, ESPL1, ETS1, ETV4, EVI5L, F2R, F2RL1, FAS, FCGR2A, FEZ1, FGF13, FGF2, FKBP4, FNBP1L, FOS, FOXM1, FYN, GAB1, GALK2, GDPD5, GHR, GJB1, GLB1, GNA12, GNA13, GOLGA2, GPSM2, GRK5, GSTM1, HAS2, HBP1, HDAC6, HMGB2, HSPB1, HTR7, ICAM1, IFT122, IL11RA, IL1B, IL1RAP, IL8, ITGA4, ITGA6, ITGB3, ITK, JUN, KBTBD10, KDR, KIAA1598, KIDINS220, KIF11, KIF13B, KIF2C, KIF4A, KIT, KLF2, KLF9, KLHL20, KRAS, KRT18, LAMA2, LAMB1, LAMC1, LIMK1, LMCD1, LOX, LPAR1, LPIN1, LRP8, LRRC16A, LYN, LZTS1, MACF1, MAP2K1, MAP2K5, MAP3K1, MAP3K12, MAP3K7, MAP6D1, MAPKAPK5, MAPRE3, MARCKS, MATN2, MBP, MERTK, MIA, MID1, MKS1, MRAS, MSN, MT2A, MTSS1, MYB, MYO10, MYO5A, NCKIPSD, NDC80, NDRG1, NEK2, NEO1, NFIA, NFIB, NFKBIA, NPC1, NPHP3, NQO1, NR2F1, NRCAM, NRP1, NRTN, NUSAP1, ONECUT2, OPHN1, OPTN, P2RX7, PACSIN2, PAK4, PARVB, PCM1, PDLIM4, PHLDB2, PI4KB, PLAT, PLAUR, PLK1S1, PLXNB1, PMP22, POU3F1, POU3F2, PPP1R9A, PREX1, PRICKLE2, PRKCE, PRMT1, PRNP, PSRC1, PTEN, PTK2, PTPRE, PTPRM, PTPRZ1, PVR, PVRL3, RAB17, RAB2A, RAB3IP, RAC2, RACGAP1, RALA, RALB, RAN, RANBP1, RAP1GAP, RAP2A, RARRES3, RGS20, RHOJ, RIN1, RIT1, RND3, RNF19A, ROCK1, ROCK2, RRBP1, RTN4, RUFY3, RUNX3, RYK, SDC4, SEMA3A, SEMA4D, SFRP1, SIAH1, SLC11A2, SLC3A2, SMPD1, SMPD2, SORBS1, SPHK1, SPP1, SRC, SRGAP2, SSH1, ST8SIA1, STK24, STMN1, STRADB, STX3, STXBP5, SYK, SYNM, TACC2, TACC3, TBC1D7, TERF1, TGIF1, THBS1, TIAM2, TIMP3, TLR4, TM4SF1, TMEM67, TNC, TNFRSF12A, TNFRSF1A, TNFSF10, TPX2, TRPV4, TTC8, TTK, TUBB3, TWIST1, ULK1, VANGL2, VASP, VEGFA, WASL, WIPF1, WISP2, WWTR1, XPC	316	yes
Cellular Function and Maintenance	organization of cytoskeleton	3.30E-10	Decreased	-3.532	ABCC1, ABI1, ABL1, ABL2, ABR, ACACA, ADCY3, ADD1, ADM, ADRBK1, AFAP1, AHR, AKAP11, AKAP12, ANGPT2, ANGPTL4, ANK3, APOE, ARAP1, ARAP3, ARHGAP24, ARHGAP33, ARHGEF1, ARHGEF17, ARHGEF2, ATF6, ATL1, ATP2B1, ATP7A, ATXN1, BAG3, BAIAP2, BAX, BBS4, BCL2, BDNF, BECN1, BIRC5, BSN, BTG3, BTK, CACNA1H, CALR, CAV1, CBL, CBY1, CCL2, CD2, CDC25B, CDC42EP1, CDC42EP4, CDK2AP2, CDK5, CDK5R1, CDK5RAP3, CDKN1A, CDKN1B, CEP350, CHAMP1, CHMP1B, CHN2, CKAP4, CLIP3, CNTNAP1, CRIPT, CRKL, CTGF, CTLA4, CXCR4, CYFIP1, CYTH2, DAPK1, DCC, DCLK1, DCX, DDAH1, DGKG, DIAPH2, DIAPH3, DICER1, DKK3, DLG4, DLGAP5, DNM3, DSP, DVL2, DYNC1LI2, DYNLL1, DZIP1L, EFHD1, EFNA1, EFNB2, ENC1, EPHA2, EPHA4, EPS15, ESPL1, ETS1, ETV4, EVI5L, F2R, F2RL1, FAS, FCGR2A, FEZ1, FGF13, FGF2, FKBP4, FNBP1L, FOS, FOXM1, FYN, GAB1, GALK2, GDPD5, GHR, GJB1, GLB1, GNA12, GNA13, GOLGA2, GPSM2, GRK5, GSTM1, HAS2, HBP1, HDAC6, HMGB2, HSPB1, HTR7, ICAM1, IFT122, IL11RA, IL1B, IL1RAP, IL8, ITGA4, ITGA6, ITGB3, ITK, JUN, KBTBD10, KDR, KIAA1598, KIDINS220, KIF11, KIF13B, KIF2C, KIF4A, KIT, KLF2, KLF9, KLHL20, KRAS, KRT18, LAMA2, LAMB1, LAMC1, LIMK1, LMCD1, LOX, LPAR1, LPIN1, LRP8, LRRC16A, LYN, LZTS1, MACF1, MAP2K1, MAP2K5, MAP3K1, MAP3K12, MAP3K7, MAP6D1, MAPKAPK5, MAPRE3, MARCKS, MATN2, MBP, MERTK, MIA, MID1, MKS1, MRAS, MSN, MT2A, MTSS1, MYB, MYO10, MYO5A, NCKIPSD, NDC80, NDRG1, NEK2, NEO1, NFIA, NFIB, NFKBIA, NPC1, NPHP3, NQO1, NR2F1, NRCAM, NRP1, NRTN, NUSAP1, ONECUT2, OPHN1, OPTN, P2RX7, PACSIN2, PAK4, PARVB, PCM1, PDLIM4, PHLDB2, PI4KB, PLAT, PLAUR, PLK1S1, PLXNB1, PMP22, POU3F1, POU3F2, PPP1R9A, PREX1, PRICKLE2, PRKCE, PRMT1, PRNP, PSRC1, PTEN, PTK2, PTPRE, PTPRM, PTPRZ1, PVR, PVRL3, RAB17, RAB2A, RAB3IP, RAC2, RACGAP1, RALA, RALB, RAN, RANBP1, RAP1GAP, RAP2A, RARRES3, RGS20, RHOJ, RIN1, RIT1, RND3, RNF19A, ROCK1, ROCK2, RRBP1, RTN4, RUFY3, RUNX3, RYK, SDC4, SEMA3A, SEMA4D, SFRP1, SIAH1, SLC11A2, SLC3A2, SMPD1, SMPD2, SORBS1, SPHK1, SPP1, SRC, SRGAP2, SSH1, ST8SIA1, STK24, STMN1, STRADB, STX3, STXBP5, SYK, SYNM, TACC2, TACC3, TBC1D7, TERF1, TGIF1, THBS1, TIAM2, TIMP3, TLR4, TM4SF1, TMEM67, TNC, TNFRSF12A, TNFRSF1A, TNFSF10, TPX2, TRPV4, TTC8, TTK, TUBB3, TWIST1, ULK1, VANGL2, VASP, VEGFA, WASL, WIPF1, WISP2, WWTR1, XPC	316	yes
Gene Expression	binding of protein binding site	1.06E-06	Decreased	-3.529	AHR, ATM, BCL2, BMPR1A, BRCA1, BTK, CCND1, CD2, CEBPB, CEBPD, CHRM1, CSDA, DDB2, DDIT3, EGR1, EGR2, EPAS1, ETS1, ETS2, FAS, FGF2, FLI1, FOS, FOXO1, FOXO3, FZD6, GABPB1, GHR, GLRX, HBEGF, HEY1, HEY2, HIVEP2, HMGA1, HOXB13, ID1, ID2, ID3, IER3, IFI16, IGFBP3, IL1B, IL8, ING4, IP6K2, ITGB3, JUN, KLF13, KRAS, LIF, LMCD1, LTBR, MAP2K1, MAP2K6, MAP3K1, MYB, MYC, NFKBIA, NR2F1, NR3C1, NR5A2, PNPT1, PPP1CC, PRDX1, PRMT2, PTEN, PTK2, RARA, RARB, RUNX1, RUNX2, RXRA, SOAT1, SOD2, SP100, SRC, SSBP2, TFAP2A, TFDP1, TFF3, TGFA, TNFRSF1A, TNFRSF1B, TRADD, TRIB3, TXLNG, VEGFA, ZBTB17	88	no
Cellular Assembly and Organization	organization of cytoplasm	4.45E-11	Decreased	-3.469	ABCA1, ABCC1, ABCD1, ABI1, ABL1, ABL2, ABR, ACACA, ADCY3, ADD1, ADM, ADRBK1, AFAP1, AHR, AKAP11, AKAP12, ANGPT2, ANGPTL4, ANK3, APOE, ARAP1, ARAP3, ARHGAP24, ARHGAP33, ARHGEF1, ARHGEF17, ARHGEF2, ATF6, ATL1, ATP2B1, ATP7A, ATXN1, BAG3, BAIAP2, BAX, BBS4, BCL2, BDNF, BECN1, BIRC5, BSN, BTG3, BTK, CACNA1H, CALR, CAV1, CBL, CBY1, CCL2, CD2, CDC25B, CDC42EP1, CDC42EP4, CDK2AP2, CDK5, CDK5R1, CDK5RAP3, CDKN1A, CDKN1B, CEP350, CHAMP1, CHMP1B, CHN2, CKAP4, CLIP3, CLN3, CLN5, CNTNAP1, COL4A3BP, CRIPT, CRKL, CROCC, CTGF, CTLA4, CXCR4, CYFIP1, CYTH2, DAPK1, DCC, DCLK1, DCX, DDAH1, DGKG, DIAPH2, DIAPH3, DICER1, DKK3, DLG4, DLGAP5, DNM3, DSP, DVL2, DYNC1LI2, DYNLL1, DZIP1L, EFHD1, EFNA1, EFNB2, ENC1, EPAS1, EPHA2, EPHA4, EPS15, ESPL1, ETS1, ETV4, EVI5L, F2R, F2RL1, FAS, FCGR2A, FEZ1, FGF13, FGF2, FKBP4, FNBP1L, FOS, FOXM1, FYN, GAB1, GALK2, GDPD5, GHR, GJB1, GLB1, GNA12, GNA13, GOLGA2, GORASP1, GPSM2, GRK5, GSTM1, HAS2, HAUS4, HBP1, HDAC6, HEXA, HMGB2, HMGCL, HPS4, HSPB1, HTR7, HTRA2, ICAM1, IFT122, IL11RA, IL1B, IL1RAP, IL8, ITGA4, ITGA6, ITGB3, ITK, JUN, KBTBD10, KDR, KIAA1598, KIDINS220, KIF11, KIF13B, KIF2C, KIF4A, KIT, KLF2, KLF9, KLHL20, KRAS, KRT18, LAMA2, LAMB1, LAMC1, LIMK1, LMCD1, LOX, LPAR1, LPIN1, LRP8, LRRC16A, LYN, LZTS1, MACF1, MAP2K1, MAP2K5, MAP3K1, MAP3K12, MAP3K7, MAP6D1, MAPKAPK5, MAPRE3, MARCKS, MATN2, MBP, MERTK, MFF, MIA, MID1, MKS1, MRAS, MSN, MT2A, MTSS1, MYB, MYO10, MYO5A, NCKIPSD, NDC80, NDRG1, NEK2, NEO1, NFIA, NFIB, NFKBIA, NPC1, NPHP3, NPLOC4, NQO1, NR2F1, NRCAM, NRP1, NRTN, NSFL1C, NUSAP1, ONECUT2, OPHN1, OPTN, P2RX7, PACSIN2, PAK4, PARVB, PCM1, PDLIM4, PEX11B, PEX16, PEX2, PEX5, PHLDB2, PI4KB, PLAT, PLAUR, PLEKHM2, PLK1S1, PLXNB1, PMP22, POU3F1, POU3F2, PPARGC1A, PPP1R9A, PREX1, PRICKLE2, PRKCE, PRKD1, PRMT1, PRNP, PSRC1, PTEN, PTK2, PTPRE, PTPRM, PTPRZ1, PVR, PVRL3, RAB17, RAB2A, RAB3IP, RAB5B, RAB6A, RAC2, RACGAP1, RALA, RALB, RAN, RANBP1, RAP1GAP, RAP2A, RARRES3, RGS20, RHOJ, RIN1, RIT1, RND3, RNF19A, ROCK1, ROCK2, RRBP1, RTN4, RUFY3, RUNX3, RYK, SDC4, SEMA3A, SEMA4D, SFRP1, SIAH1, SLC11A2, SLC3A2, SMPD1, SMPD2, SNX10, SOD2, SORBS1, SPHK1, SPP1, SRC, SRGAP2, SSH1, ST8SIA1, STK24, STMN1, STRADB, STX3, STXBP5, SYK, SYNJ2BP, SYNM, TACC2, TACC3, TBC1D7, TERF1, TGIF1, THBS1, TIAM2, TIMP3, TLR4, TM4SF1, TMEM67, TNC, TNFRSF12A, TNFRSF1A, TNFSF10, TPX2, TRPV4, TTC8, TTK, TUBB3, TWIST1, ULK1, VANGL2, VASP, VEGFA, VPS18, WASL, WIPF1, WISP2, WWTR1, XPC	345	yes
Cellular Function and Maintenance	organization of cytoplasm	4.45E-11	Decreased	-3.469	ABCA1, ABCC1, ABCD1, ABI1, ABL1, ABL2, ABR, ACACA, ADCY3, ADD1, ADM, ADRBK1, AFAP1, AHR, AKAP11, AKAP12, ANGPT2, ANGPTL4, ANK3, APOE, ARAP1, ARAP3, ARHGAP24, ARHGAP33, ARHGEF1, ARHGEF17, ARHGEF2, ATF6, ATL1, ATP2B1, ATP7A, ATXN1, BAG3, BAIAP2, BAX, BBS4, BCL2, BDNF, BECN1, BIRC5, BSN, BTG3, BTK, CACNA1H, CALR, CAV1, CBL, CBY1, CCL2, CD2, CDC25B, CDC42EP1, CDC42EP4, CDK2AP2, CDK5, CDK5R1, CDK5RAP3, CDKN1A, CDKN1B, CEP350, CHAMP1, CHMP1B, CHN2, CKAP4, CLIP3, CLN3, CLN5, CNTNAP1, COL4A3BP, CRIPT, CRKL, CROCC, CTGF, CTLA4, CXCR4, CYFIP1, CYTH2, DAPK1, DCC, DCLK1, DCX, DDAH1, DGKG, DIAPH2, DIAPH3, DICER1, DKK3, DLG4, DLGAP5, DNM3, DSP, DVL2, DYNC1LI2, DYNLL1, DZIP1L, EFHD1, EFNA1, EFNB2, ENC1, EPAS1, EPHA2, EPHA4, EPS15, ESPL1, ETS1, ETV4, EVI5L, F2R, F2RL1, FAS, FCGR2A, FEZ1, FGF13, FGF2, FKBP4, FNBP1L, FOS, FOXM1, FYN, GAB1, GALK2, GDPD5, GHR, GJB1, GLB1, GNA12, GNA13, GOLGA2, GORASP1, GPSM2, GRK5, GSTM1, HAS2, HAUS4, HBP1, HDAC6, HEXA, HMGB2, HMGCL, HPS4, HSPB1, HTR7, HTRA2, ICAM1, IFT122, IL11RA, IL1B, IL1RAP, IL8, ITGA4, ITGA6, ITGB3, ITK, JUN, KBTBD10, KDR, KIAA1598, KIDINS220, KIF11, KIF13B, KIF2C, KIF4A, KIT, KLF2, KLF9, KLHL20, KRAS, KRT18, LAMA2, LAMB1, LAMC1, LIMK1, LMCD1, LOX, LPAR1, LPIN1, LRP8, LRRC16A, LYN, LZTS1, MACF1, MAP2K1, MAP2K5, MAP3K1, MAP3K12, MAP3K7, MAP6D1, MAPKAPK5, MAPRE3, MARCKS, MATN2, MBP, MERTK, MFF, MIA, MID1, MKS1, MRAS, MSN, MT2A, MTSS1, MYB, MYO10, MYO5A, NCKIPSD, NDC80, NDRG1, NEK2, NEO1, NFIA, NFIB, NFKBIA, NPC1, NPHP3, NPLOC4, NQO1, NR2F1, NRCAM, NRP1, NRTN, NSFL1C, NUSAP1, ONECUT2, OPHN1, OPTN, P2RX7, PACSIN2, PAK4, PARVB, PCM1, PDLIM4, PEX11B, PEX16, PEX2, PEX5, PHLDB2, PI4KB, PLAT, PLAUR, PLEKHM2, PLK1S1, PLXNB1, PMP22, POU3F1, POU3F2, PPARGC1A, PPP1R9A, PREX1, PRICKLE2, PRKCE, PRKD1, PRMT1, PRNP, PSRC1, PTEN, PTK2, PTPRE, PTPRM, PTPRZ1, PVR, PVRL3, RAB17, RAB2A, RAB3IP, RAB5B, RAB6A, RAC2, RACGAP1, RALA, RALB, RAN, RANBP1, RAP1GAP, RAP2A, RARRES3, RGS20, RHOJ, RIN1, RIT1, RND3, RNF19A, ROCK1, ROCK2, RRBP1, RTN4, RUFY3, RUNX3, RYK, SDC4, SEMA3A, SEMA4D, SFRP1, SIAH1, SLC11A2, SLC3A2, SMPD1, SMPD2, SNX10, SOD2, SORBS1, SPHK1, SPP1, SRC, SRGAP2, SSH1, ST8SIA1, STK24, STMN1, STRADB, STX3, STXBP5, SYK, SYNJ2BP, SYNM, TACC2, TACC3, TBC1D7, TERF1, TGIF1, THBS1, TIAM2, TIMP3, TLR4, TM4SF1, TMEM67, TNC, TNFRSF12A, TNFRSF1A, TNFSF10, TPX2, TRPV4, TTC8, TTK, TUBB3, TWIST1, ULK1, VANGL2, VASP, VEGFA, VPS18, WASL, WIPF1, WISP2, WWTR1, XPC	345	yes
Cellular Development	formation of plasma membrane projections	5.89E-04	Decreased	-3.384	ABL1, ABL2, ADCY3, AHR, ANK3, APOE, ARHGAP24, ARHGAP33, ATF6, ATL1, ATP7A, ATXN1, BAIAP2, BBS4, BCL2, BDNF, BSN, BTG3, CACNA1H, CAV1, CDK5, CDK5R1, CDKN1A, CNTNAP1, CYTH2, DCC, DCLK1, DCX, DDAH1, DICER1, DLG4, EFHD1, EFNA1, EFNB2, ENC1, EPHA4, ETV4, FEZ1, FGF13, FGF2, FNBP1L, FOS, FYN, GDPD5, HMGB2, IL11RA, IL1B, IL1RAP, ITGB3, JUN, KDR, KIAA1598, KIDINS220, KLF9, LAMB1, LAMC1, LIMK1, LOX, LRP8, LYN, LZTS1, MAP2K1, MATN2, MBP, MSN, MT2A, MYO5A, NFIA, NFIB, NFKBIA, NRCAM, NRP1, NRTN, OPHN1, OPTN, P2RX7, PAK4, PARVB, PLAT, PLXNB1, PMP22, POU3F1, POU3F2, PPP1R9A, PREX1, PRICKLE2, PRKCE, PRMT1, PRNP, PTEN, PTK2, PTPRE, PTPRM, PTPRZ1, RAB17, RAC2, RALA, RALB, RAP2A, RIT1, ROCK1, ROCK2, RTN4, RUFY3, RUNX3, RYK, SEMA3A, SEMA4D, SLC11A2, SMPD1, SMPD2, SRC, SRGAP2, ST8SIA1, STMN1, STX3, STXBP5, TIAM2, TNC, TRPV4, ULK1, VASP, WASL, WISP2	124	yes
Cellular Assembly and Organization	formation of plasma membrane projections	5.89E-04	Decreased	-3.384	ABL1, ABL2, ADCY3, AHR, ANK3, APOE, ARHGAP24, ARHGAP33, ATF6, ATL1, ATP7A, ATXN1, BAIAP2, BBS4, BCL2, BDNF, BSN, BTG3, CACNA1H, CAV1, CDK5, CDK5R1, CDKN1A, CNTNAP1, CYTH2, DCC, DCLK1, DCX, DDAH1, DICER1, DLG4, EFHD1, EFNA1, EFNB2, ENC1, EPHA4, ETV4, FEZ1, FGF13, FGF2, FNBP1L, FOS, FYN, GDPD5, HMGB2, IL11RA, IL1B, IL1RAP, ITGB3, JUN, KDR, KIAA1598, KIDINS220, KLF9, LAMB1, LAMC1, LIMK1, LOX, LRP8, LYN, LZTS1, MAP2K1, MATN2, MBP, MSN, MT2A, MYO5A, NFIA, NFIB, NFKBIA, NRCAM, NRP1, NRTN, OPHN1, OPTN, P2RX7, PAK4, PARVB, PLAT, PLXNB1, PMP22, POU3F1, POU3F2, PPP1R9A, PREX1, PRICKLE2, PRKCE, PRMT1, PRNP, PTEN, PTK2, PTPRE, PTPRM, PTPRZ1, RAB17, RAC2, RALA, RALB, RAP2A, RIT1, ROCK1, ROCK2, RTN4, RUFY3, RUNX3, RYK, SEMA3A, SEMA4D, SLC11A2, SMPD1, SMPD2, SRC, SRGAP2, ST8SIA1, STMN1, STX3, STXBP5, TIAM2, TNC, TRPV4, ULK1, VASP, WASL, WISP2	124	yes
Cellular Function and Maintenance	formation of plasma membrane projections	5.89E-04	Decreased	-3.384	ABL1, ABL2, ADCY3, AHR, ANK3, APOE, ARHGAP24, ARHGAP33, ATF6, ATL1, ATP7A, ATXN1, BAIAP2, BBS4, BCL2, BDNF, BSN, BTG3, CACNA1H, CAV1, CDK5, CDK5R1, CDKN1A, CNTNAP1, CYTH2, DCC, DCLK1, DCX, DDAH1, DICER1, DLG4, EFHD1, EFNA1, EFNB2, ENC1, EPHA4, ETV4, FEZ1, FGF13, FGF2, FNBP1L, FOS, FYN, GDPD5, HMGB2, IL11RA, IL1B, IL1RAP, ITGB3, JUN, KDR, KIAA1598, KIDINS220, KLF9, LAMB1, LAMC1, LIMK1, LOX, LRP8, LYN, LZTS1, MAP2K1, MATN2, MBP, MSN, MT2A, MYO5A, NFIA, NFIB, NFKBIA, NRCAM, NRP1, NRTN, OPHN1, OPTN, P2RX7, PAK4, PARVB, PLAT, PLXNB1, PMP22, POU3F1, POU3F2, PPP1R9A, PREX1, PRICKLE2, PRKCE, PRMT1, PRNP, PTEN, PTK2, PTPRE, PTPRM, PTPRZ1, RAB17, RAC2, RALA, RALB, RAP2A, RIT1, ROCK1, ROCK2, RTN4, RUFY3, RUNX3, RYK, SEMA3A, SEMA4D, SLC11A2, SMPD1, SMPD2, SRC, SRGAP2, ST8SIA1, STMN1, STX3, STXBP5, TIAM2, TNC, TRPV4, ULK1, VASP, WASL, WISP2	124	yes
Cell Morphology	formation of plasma membrane projections	5.89E-04	Decreased	-3.384	ABL1, ABL2, ADCY3, AHR, ANK3, APOE, ARHGAP24, ARHGAP33, ATF6, ATL1, ATP7A, ATXN1, BAIAP2, BBS4, BCL2, BDNF, BSN, BTG3, CACNA1H, CAV1, CDK5, CDK5R1, CDKN1A, CNTNAP1, CYTH2, DCC, DCLK1, DCX, DDAH1, DICER1, DLG4, EFHD1, EFNA1, EFNB2, ENC1, EPHA4, ETV4, FEZ1, FGF13, FGF2, FNBP1L, FOS, FYN, GDPD5, HMGB2, IL11RA, IL1B, IL1RAP, ITGB3, JUN, KDR, KIAA1598, KIDINS220, KLF9, LAMB1, LAMC1, LIMK1, LOX, LRP8, LYN, LZTS1, MAP2K1, MATN2, MBP, MSN, MT2A, MYO5A, NFIA, NFIB, NFKBIA, NRCAM, NRP1, NRTN, OPHN1, OPTN, P2RX7, PAK4, PARVB, PLAT, PLXNB1, PMP22, POU3F1, POU3F2, PPP1R9A, PREX1, PRICKLE2, PRKCE, PRMT1, PRNP, PTEN, PTK2, PTPRE, PTPRM, PTPRZ1, RAB17, RAC2, RALA, RALB, RAP2A, RIT1, ROCK1, ROCK2, RTN4, RUFY3, RUNX3, RYK, SEMA3A, SEMA4D, SLC11A2, SMPD1, SMPD2, SRC, SRGAP2, ST8SIA1, STMN1, STX3, STXBP5, TIAM2, TNC, TRPV4, ULK1, VASP, WASL, WISP2	124	yes
Cellular Assembly and Organization	microtubule dynamics	9.05E-11	Decreased	-3.297	ABCC1, ABI1, ABL1, ABL2, ACACA, ADCY3, ADM, AHR, AKAP11, AKAP12, ANGPT2, ANGPTL4, ANK3, APOE, ARAP1, ARHGAP24, ARHGAP33, ARHGEF2, ATF6, ATL1, ATP2B1, ATP7A, ATXN1, BAIAP2, BAX, BBS4, BCL2, BDNF, BECN1, BIRC5, BSN, BTG3, BTK, CACNA1H, CAV1, CBL, CBY1, CCL2, CD2, CDC25B, CDC42EP1, CDC42EP4, CDK2AP2, CDK5, CDK5R1, CDK5RAP3, CDKN1A, CDKN1B, CEP350, CHAMP1, CHMP1B, CHN2, CKAP4, CLIP3, CNTNAP1, CRIPT, CRKL, CTGF, CTLA4, CXCR4, CYFIP1, CYTH2, DAPK1, DCC, DCLK1, DCX, DDAH1, DGKG, DIAPH2, DIAPH3, DICER1, DKK3, DLG4, DLGAP5, DNM3, DSP, DVL2, DYNC1LI2, DYNLL1, DZIP1L, EFHD1, EFNA1, EFNB2, ENC1, EPHA2, EPHA4, EPS15, ETV4, EVI5L, F2R, F2RL1, FAS, FEZ1, FGF13, FGF2, FKBP4, FNBP1L, FOS, FOXM1, FYN, GAB1, GALK2, GDPD5, GHR, GJB1, GLB1, GNA12, GNA13, GOLGA2, GRK5, GSTM1, HDAC6, HMGB2, HSPB1, HTR7, ICAM1, IFT122, IL11RA, IL1B, IL1RAP, IL8, ITGA4, ITGA6, ITGB3, JUN, KBTBD10, KDR, KIAA1598, KIDINS220, KIF13B, KIF2C, KIF4A, KIT, KLF2, KLF9, KRT18, LAMA2, LAMB1, LAMC1, LIMK1, LMCD1, LOX, LPAR1, LRP8, LRRC16A, LYN, LZTS1, MACF1, MAP2K1, MAP3K1, MAP3K12, MAP3K7, MAP6D1, MAPRE3, MARCKS, MATN2, MBP, MIA, MID1, MKS1, MRAS, MSN, MT2A, MTSS1, MYB, MYO10, MYO5A, NCKIPSD, NDRG1, NEK2, NEO1, NFIA, NFIB, NFKBIA, NPC1, NPHP3, NQO1, NR2F1, NRCAM, NRP1, NRTN, NUSAP1, ONECUT2, OPHN1, OPTN, P2RX7, PACSIN2, PAK4, PARVB, PCM1, PHLDB2, PI4KB, PLAT, PLXNB1, PMP22, POU3F1, POU3F2, PPP1R9A, PREX1, PRICKLE2, PRKCE, PRMT1, PRNP, PSRC1, PTEN, PTK2, PTPRE, PTPRM, PTPRZ1, PVR, PVRL3, RAB17, RAB2A, RAB3IP, RAC2, RACGAP1, RALA, RALB, RAN, RANBP1, RAP1GAP, RAP2A, RARRES3, RGS20, RIN1, RIT1, RNF19A, ROCK1, ROCK2, RRBP1, RTN4, RUFY3, RUNX3, RYK, SDC4, SEMA3A, SEMA4D, SLC11A2, SMPD1, SMPD2, SPHK1, SRC, SRGAP2, ST8SIA1, STK24, STMN1, STX3, STXBP5, SYK, TACC2, TACC3, TBC1D7, TERF1, TGIF1, THBS1, TIAM2, TIMP3, TLR4, TM4SF1, TMEM67, TNC, TNFRSF12A, TNFRSF1A, TPX2, TRPV4, TTC8, TUBB3, TWIST1, ULK1, VANGL2, VASP, VEGFA, WASL, WIPF1, WISP2, WWTR1, XPC	277	yes
Cellular Function and Maintenance	microtubule dynamics	9.05E-11	Decreased	-3.297	ABCC1, ABI1, ABL1, ABL2, ACACA, ADCY3, ADM, AHR, AKAP11, AKAP12, ANGPT2, ANGPTL4, ANK3, APOE, ARAP1, ARHGAP24, ARHGAP33, ARHGEF2, ATF6, ATL1, ATP2B1, ATP7A, ATXN1, BAIAP2, BAX, BBS4, BCL2, BDNF, BECN1, BIRC5, BSN, BTG3, BTK, CACNA1H, CAV1, CBL, CBY1, CCL2, CD2, CDC25B, CDC42EP1, CDC42EP4, CDK2AP2, CDK5, CDK5R1, CDK5RAP3, CDKN1A, CDKN1B, CEP350, CHAMP1, CHMP1B, CHN2, CKAP4, CLIP3, CNTNAP1, CRIPT, CRKL, CTGF, CTLA4, CXCR4, CYFIP1, CYTH2, DAPK1, DCC, DCLK1, DCX, DDAH1, DGKG, DIAPH2, DIAPH3, DICER1, DKK3, DLG4, DLGAP5, DNM3, DSP, DVL2, DYNC1LI2, DYNLL1, DZIP1L, EFHD1, EFNA1, EFNB2, ENC1, EPHA2, EPHA4, EPS15, ETV4, EVI5L, F2R, F2RL1, FAS, FEZ1, FGF13, FGF2, FKBP4, FNBP1L, FOS, FOXM1, FYN, GAB1, GALK2, GDPD5, GHR, GJB1, GLB1, GNA12, GNA13, GOLGA2, GRK5, GSTM1, HDAC6, HMGB2, HSPB1, HTR7, ICAM1, IFT122, IL11RA, IL1B, IL1RAP, IL8, ITGA4, ITGA6, ITGB3, JUN, KBTBD10, KDR, KIAA1598, KIDINS220, KIF13B, KIF2C, KIF4A, KIT, KLF2, KLF9, KRT18, LAMA2, LAMB1, LAMC1, LIMK1, LMCD1, LOX, LPAR1, LRP8, LRRC16A, LYN, LZTS1, MACF1, MAP2K1, MAP3K1, MAP3K12, MAP3K7, MAP6D1, MAPRE3, MARCKS, MATN2, MBP, MIA, MID1, MKS1, MRAS, MSN, MT2A, MTSS1, MYB, MYO10, MYO5A, NCKIPSD, NDRG1, NEK2, NEO1, NFIA, NFIB, NFKBIA, NPC1, NPHP3, NQO1, NR2F1, NRCAM, NRP1, NRTN, NUSAP1, ONECUT2, OPHN1, OPTN, P2RX7, PACSIN2, PAK4, PARVB, PCM1, PHLDB2, PI4KB, PLAT, PLXNB1, PMP22, POU3F1, POU3F2, PPP1R9A, PREX1, PRICKLE2, PRKCE, PRMT1, PRNP, PSRC1, PTEN, PTK2, PTPRE, PTPRM, PTPRZ1, PVR, PVRL3, RAB17, RAB2A, RAB3IP, RAC2, RACGAP1, RALA, RALB, RAN, RANBP1, RAP1GAP, RAP2A, RARRES3, RGS20, RIN1, RIT1, RNF19A, ROCK1, ROCK2, RRBP1, RTN4, RUFY3, RUNX3, RYK, SDC4, SEMA3A, SEMA4D, SLC11A2, SMPD1, SMPD2, SPHK1, SRC, SRGAP2, ST8SIA1, STK24, STMN1, STX3, STXBP5, SYK, TACC2, TACC3, TBC1D7, TERF1, TGIF1, THBS1, TIAM2, TIMP3, TLR4, TM4SF1, TMEM67, TNC, TNFRSF12A, TNFRSF1A, TPX2, TRPV4, TTC8, TUBB3, TWIST1, ULK1, VANGL2, VASP, VEGFA, WASL, WIPF1, WISP2, WWTR1, XPC	277	yes

Cellular Movement	cell movement of connective tissue cells	3.52E-06	Decreased	-3.246	ABL1, ABL2, ANGPT1, ANGPT2, APOE, ARID5B, CAV1, CBL, CCL2, CCNA2, CCND1, CDKN1A, CDKN1B, CTGF, CYTH2, ENPP2, F2R, FGF2, FOS, FOSL1, GNA12, GNA13, HBEGF, HMMR, IGF2R, IL16, IL1B, ITGB3, JUN, LIMA1, LIMK1, LPAR1, MAP2K1, MAP3K1, MRAS, MYC, NOV, PLAUR, PRKAR1A, PRKCE, PTEN, PTK2, PTPRA, RAC2, RAP2A, RAPGEF1, SCHIP1, SDC4, SEMA3A, SMO, SNX17, SPP1, SRC, STMN1, TGFA, TGFBR2, THBS1, TIMP2, VASP, ZFAND5, ZYX	61	yes
Cellular Growth and Proliferation	proliferation of epithelial cells	1.91E-04	Decreased	-3.241	ASIP, BAX, BMPR1A, BRCA1, CCND1, CCND2, CCNG2, CDC25A, CDC25B, CDK6, CDKN1A, CDKN1B, CDKN2C, CEBPB, DCC, DKK1, EDNRB, EFNA1, EPHA2, FGF2, FGFR3, FOS, FOXM1, FST, FYN, FZD7, GAS1, GDF15, GLI2, HBEGF, HOXA5, HOXB13, HSF4, ID1, ID2, IL1B, IL8, ITGA6, ITGB3, JUN, KDR, KIT, KLF4, KRAS, LAMB1, LAMC1, LRIG1, LTBP4, LTBR, MAGED1, MAP2K1, MAP2K5, MAP3K5, MAP3K7, MC1R, MCC, MMP9, MST1, MYB, MYC, NFIB, NFKBIA, NKX3-1, NR3C1, OVOL2, PAX3, PEX2, POU3F2, PPP1R13L, PTEN, PTGS2, PTK2, PTPN12, PTPRZ1, RARA, RRAS, RUNX1, RUNX3, RXRA, SEMA4D, SFRP1, SMO, SNAI2, SOX2, SPHK1, SPP1, STMN1, TCF4, TCF7L2, TGFA, TGFBR2, TGFBR3, TIMELESS, TNFRSF1A, TNFSF10, TWIST1, VDR, VEGFA	98	yes
Cellular Movement	homing of cells	6.72E-05	Decreased	-3.233	ABCC1, ABL1, ADRBK1, ANGPT1, ANGPT2, ANGPTL2, ANXA1, ANXA2, APOE, ARAP3, BCL2, BDNF, C3AR1, CALR, CASP1, CAV1, CBL, CCL2, CCL20, CCL28, CCND1, CCR1, CD36, CD74, CDKN1A, CEACAM1, CHST2, CKLF, COL4A3BP, CRKL, CTGF, CTSE, CXCL13, CXCL16, CXCR4, DEFB4A/DEFB4B, DUSP6, EDNRB, EFNA1, ENPP2, EPHA2, ETV4, F2R, F2RL1, FADD, FAS, FCER1G, FCGR2A, FGF2, FOXO1, FYB, FYN, GAB1, GNA12, GNA13, HBEGF, HDAC6, HMGB2, HSPD1, ICAM1, IGF2R, IGFBP3, IL16, IL1B, IL8, ITGA4, ITGA6, ITGB3, JAM3, JUN, KDR, KIT, LEF1, LIF, LIMK1, LOX, LTBR, LYN, MAP3K1, MAP3K5, MDK, MMP9, MPP1, MST1, MYB, MYLK, NEDD9, NOV, NPTX1, NQO1, NRCAM, NRP1, PDE4B, PGF, PIK3CD, PLAUR, PLXNB1, PREX1, PRNP, PTEN, PTGES, PTGS2, PTK2, PTPRA, PTPRZ1, RAC2, RAP1GAP, RGS1, ROCK1, RTN4, RUNX1, SCG2, SEMA3A, SEMA4D, SNAI2, SPHK1, SPP1, SRC, SYK, TFF3, TGFA, THBS1, THBS2, TIRAP, TLR4, TNFRSF1A, TRAF3IP2, TRIB1, TRPV2, VEGFA, VEGFB, WASL, YARS	133	yes
Cellular Movement	homing	5.72E-05	Decreased	-3.229	ABCC1, ABL1, ADRBK1, ANGPT1, ANGPT2, ANGPTL2, ANXA1, ANXA2, APOE, ARAP3, BCL2, BDNF, C3AR1, CALR, CASP1, CAV1, CBL, CCL2, CCL20, CCL28, CCND1, CCR1, CD36, CD74, CDKN1A, CEACAM1, CHST2, CKLF, COL4A3BP, CRKL, CTGF, CTSE, CXCL13, CXCL16, CXCR4, DEFB4A/DEFB4B, DUSP6, EDNRB, EFNA1, EFNB2, ENPP2, EPHA2, ETV4, F2R, F2RL1, FADD, FAS, FCER1G, FCGR2A, FGF2, FOSL1, FOXO1, FYB, FYN, GAB1, GNA12, GNA13, HBEGF, HDAC6, HMGB2, HSPD1, ICAM1, IGF2R, IGFBP3, IL16, IL1B, IL8, ITGA4, ITGA6, ITGB3, JAM3, JUN, KDR, KIT, LEF1, LIF, LIMK1, LOX, LTBR, LYN, MAP2K1, MAP3K1, MAP3K5, MDK, MMP9, MPP1, MST1, MYB, MYLK, NEDD9, NOV, NPTX1, NQO1, NRCAM, NRP1, PDE4B, PGF, PIK3CD, PLAUR, PLXNB1, PREX1, PRNP, PTEN, PTGES, PTGS2, PTK2, PTPRA, PTPRZ1, RAC2, RALA, RAP1GAP, RGS1, ROCK1, RTN4, RUNX1, SCG2, SEMA3A, SEMA4D, SNAI2, SPHK1, SPP1, SRC, SYK, TFF3, TGFA, THBS1, THBS2, TIRAP, TLR4, TNFRSF1A, TRAF3IP2, TRIB1, TRPV2, VEGFA, VEGFB, WASL, YARS	137	yes
Organismal Development	formation of vessel	3.92E-04	Decreased	-3.137	ADAMTS1, AHR, ANGPT1, ANGPT2, APOE, CCL2, CTGF, CXCR4, DGCR8, EFNB2, ENPP2, EPAS1, F12, FGF2, FOXM1, FOXO3, GAB1, IL1B, ING4, ITGB3, JUN, KDR, LTBR, MEF2C, MMP9, NRP1, PLAUR, SEMA4D, SRC, TGFBR3, TIMP2, VEGFA	32	yes
Cellular Movement	movement of vascular endothelial cells	1.79E-06	Decreased	-3.060	ADAMTS1, ADM, ANGPT1, ANGPT2, ANXA2, ARHGEF6, CDKN1B, DICER1, DKK1, EFNA1, EFNB2, F2RL1, FGF13, FGF2, FOXO1, FOXO3, GAB1, HHEX, HIF1A, HSP90AB1, ID1, ID3, IL8, ITGB3, KDR, KLF2, KLHL20, MAP3K7, MAPKAPK5, MDK, MMP9, NRP1, PTEN, PTGS2, PTPRZ1, RTN4, SP100, SPRY4, SRC, TGFBR2, THBS1, THBS2, TIMP2, TNFSF10, VEGFA	45	yes
Cardiovascular System Development and Function	movement of vascular endothelial cells	1.79E-06	Decreased	-3.060	ADAMTS1, ADM, ANGPT1, ANGPT2, ANXA2, ARHGEF6, CDKN1B, DICER1, DKK1, EFNA1, EFNB2, F2RL1, FGF13, FGF2, FOXO1, FOXO3, GAB1, HHEX, HIF1A, HSP90AB1, ID1, ID3, IL8, ITGB3, KDR, KLF2, KLHL20, MAP3K7, MAPKAPK5, MDK, MMP9, NRP1, PTEN, PTGS2, PTPRZ1, RTN4, SP100, SPRY4, SRC, TGFBR2, THBS1, THBS2, TIMP2, TNFSF10, VEGFA	45	yes
Cellular Movement	cell movement of muscle cells	2.09E-05	Decreased	-3.010	ADM, ANGPT2, APOE, CBL, CCL2, CCND1, CDKN1B, CLIC4, DUSP1, ENPP2, F2R, FGF2, FOXO1, FOXO4, FURIN, FZD4, GAB1, HBEGF, HMOX1, IGFBP5, IL1B, IL8, ITGB5, KDR, LOX, LPAR1, MMP9, MYC, NEO1, NOV, NQO1, PAPPA, PAX3, PLAT, PLAUR, PTGS2, PTK2, RCAN1, ROCK1, RTN4, SDC4, SIX4, SPP1, THBS1, TLR4, TNFSF10, TRIB1, VCAN, WISP2	49	yes
Cellular Movement	cell movement of smooth muscle cells	3.88E-05	Decreased	-2.991	ADM, ANGPT2, APOE, CBL, CCL2, CCND1, CDKN1B, CLIC4, DUSP1, ENPP2, F2R, FGF2, FOXO4, FZD4, HBEGF, HMOX1, IGFBP5, IL1B, IL8, ITGB5, KDR, LOX, LPAR1, MMP9, MYC, NEO1, NOV, NQO1, PAPPA, PLAT, PLAUR, PTGS2, PTK2, RCAN1, ROCK1, RTN4, SDC4, SPP1, THBS1, TLR4, TNFSF10, TRIB1, VCAN, WISP2	44	yes
Skeletal and Muscular System Development and Function	cell movement of smooth muscle cells	3.88E-05	Decreased	-2.991	ADM, ANGPT2, APOE, CBL, CCL2, CCND1, CDKN1B, CLIC4, DUSP1, ENPP2, F2R, FGF2, FOXO4, FZD4, HBEGF, HMOX1, IGFBP5, IL1B, IL8, ITGB5, KDR, LOX, LPAR1, MMP9, MYC, NEO1, NOV, NQO1, PAPPA, PLAT, PLAUR, PTGS2, PTK2, RCAN1, ROCK1, RTN4, SDC4, SPP1, THBS1, TLR4, TNFSF10, TRIB1, VCAN, WISP2	44	yes
Cellular Development	epithelial-mesenchymal transition	1.91E-05	Decreased	-2.972	ABL1, CAV1, CLIC4, DICER1, EFNA1, ESRP1, ETV5, FGF2, FOXO1, FTH1, HAS2, HEY1, HEY2, HHEX, HIF1A, HMGA2, HNRNPAB, ID1, KRAS, LEF1, LOXL3, MAP3K3, MCAM, MESP1, MRAS, NDRG1, NFKBIA, NRP1, PAX3, PLAUR, PRNP, PTEN, PTPN14, RUNX3, SNAI2, STMN1, TGFBR2, TGFBR3, TWIST1, VEGFA, ZEB2	41	yes
Cellular Movement	cell movement of fibroblasts	2.77E-07	Decreased	-2.940	ABL1, ABL2, ANGPT1, ANGPT2, APOE, ARID5B, CAV1, CCL2, CCNA2, CCND1, CDKN1A, CDKN1B, ENPP2, F2R, FGF2, FOS, GNA12, GNA13, HBEGF, HMMR, IGF2R, IL16, IL1B, ITGB3, JUN, LIMK1, LPAR1, MAP2K1, MAP3K1, MRAS, MYC, NOV, PLAUR, PRKAR1A, PRKCE, PTEN, PTK2, PTPRA, RAC2, RAP2A, RAPGEF1, SCHIP1, SDC4, SMO, SNX17, SPP1, SRC, STMN1, TGFA, TGFBR2, THBS1, TIMP2, VASP, ZFAND5, ZYX	55	yes
Connective Tissue Development and Function	cell movement of fibroblasts	2.77E-07	Decreased	-2.940	ABL1, ABL2, ANGPT1, ANGPT2, APOE, ARID5B, CAV1, CCL2, CCNA2, CCND1, CDKN1A, CDKN1B, ENPP2, F2R, FGF2, FOS, GNA12, GNA13, HBEGF, HMMR, IGF2R, IL16, IL1B, ITGB3, JUN, LIMK1, LPAR1, MAP2K1, MAP3K1, MRAS, MYC, NOV, PLAUR, PRKAR1A, PRKCE, PTEN, PTK2, PTPRA, RAC2, RAP2A, RAPGEF1, SCHIP1, SDC4, SMO, SNX17, SPP1, SRC, STMN1, TGFA, TGFBR2, THBS1, TIMP2, VASP, ZFAND5, ZYX	55	yes
Cardiovascular System Development and Function	formation of blood vessel	3.01E-04	Decreased	-2.867	ADAMTS1, ANGPT1, APOE, CCL2, CTGF, CXCR4, DGCR8, EFNB2, ENPP2, F12, FGF2, FOXM1, FOXO3, GAB1, IL1B, ING4, ITGB3, JUN, KDR, MEF2C, MMP9, NRP1, PLAUR, SEMA4D, SRC, TGFBR3, TIMP2, VEGFA	28	yes
Organismal Development	formation of blood vessel	3.01E-04	Decreased	-2.867	ADAMTS1, ANGPT1, APOE, CCL2, CTGF, CXCR4, DGCR8, EFNB2, ENPP2, F12, FGF2, FOXM1, FOXO3, GAB1, IL1B, ING4, ITGB3, JUN, KDR, MEF2C, MMP9, NRP1, PLAUR, SEMA4D, SRC, TGFBR3, TIMP2, VEGFA	28	yes
Cellular Movement	migration of fibroblast cell lines	1.57E-04	Decreased	-2.865	ABL1, BDNF, CCNA2, CDKN1A, CDKN1B, CLIC4, CRKL, ELK3, FGF2, GNG12, HAS2, HAS3, HBEGF, IL8, KLF2, LIMA1, MARCKS, MMP9, MYLK, NOX4, PLAUR, PRKD1, PTEN, PTGDS, PTGS2, PTK2, PTPN12, PTPRA, PVR, RAC2, SPP1, SRC, TP53INP1, VEGFA	34	yes
Connective Tissue Development and Function	migration of fibroblast cell lines	1.57E-04	Decreased	-2.865	ABL1, BDNF, CCNA2, CDKN1A, CDKN1B, CLIC4, CRKL, ELK3, FGF2, GNG12, HAS2, HAS3, HBEGF, IL8, KLF2, LIMA1, MARCKS, MMP9, MYLK, NOX4, PLAUR, PRKD1, PTEN, PTGDS, PTGS2, PTK2, PTPN12, PTPRA, PVR, RAC2, SPP1, SRC, TP53INP1, VEGFA	34	yes
Cellular Movement	migration of smooth muscle cells	5.45E-05	Decreased	-2.846	ADM, ANGPT2, APOE, CBL, CCL2, CDKN1B, CLIC4, DUSP1, F2R, FGF2, FOXO4, FZD4, HBEGF, HMOX1, IGFBP5, IL1B, IL8, ITGB5, KDR, LPAR1, MMP9, MYC, NEO1, NOV, NQO1, PAPPA, PLAT, PLAUR, PTGS2, PTK2, RCAN1, ROCK1, RTN4, SDC4, SPP1, THBS1, TLR4, TNFSF10, VCAN, WISP2	40	yes
Skeletal and Muscular System Development and Function	migration of smooth muscle cells	5.45E-05	Decreased	-2.846	ADM, ANGPT2, APOE, CBL, CCL2, CDKN1B, CLIC4, DUSP1, F2R, FGF2, FOXO4, FZD4, HBEGF, HMOX1, IGFBP5, IL1B, IL8, ITGB5, KDR, LPAR1, MMP9, MYC, NEO1, NOV, NQO1, PAPPA, PLAT, PLAUR, PTGS2, PTK2, RCAN1, ROCK1, RTN4, SDC4, SPP1, THBS1, TLR4, TNFSF10, VCAN, WISP2	40	yes
Cellular Movement	homing of endothelial cells	1.88E-04	Decreased	-2.835	ANGPT1, ANGPT2, CCL2, CD36, CEACAM1, CXCR4, EFNA1, ENPP2, FGF2, IGF2R, IGFBP3, IL8, KDR, NPTX1, PTGS2, SEMA4D, THBS1, THBS2, VEGFA	19	yes
Cardiovascular System Development and Function	homing of endothelial cells	1.88E-04	Decreased	-2.835	ANGPT1, ANGPT2, CCL2, CD36, CEACAM1, CXCR4, EFNA1, ENPP2, FGF2, IGF2R, IGFBP3, IL8, KDR, NPTX1, PTGS2, SEMA4D, THBS1, THBS2, VEGFA	19	yes
Gene Expression	binding of DNA	3.97E-05	Decreased	-2.801	ABL1, AHR, ARNT, ATF6B, ATM, BCL2, BCL3, BMPR1A, BRCA1, BTK, CALM1 (includes others), CALR, CAV1, CCND1, CD2, CDKN1A, CEBPB, CEBPD, CHRM1, CSDA, CTLA4, DDB2, DDIT3, EGR1, EGR2, EPAS1, EPOR, ERCC5, ETS1, ETS2, FADD, FAS, FGF13, FGF2, FLI1, FOS, FOSL1, FOXM1, FOXO1, FOXO3, FYB, FZD6, GABPB1, GFPT1, GHR, GLRX, GTF2B, HBEGF, HEY1, HEY2, HIF1A, HIPK2, HIVEP2, HMGA1, HMGB2, HOXB13, ID1, ID2, ID3, IER3, IFI16, IFNAR1, IGFBP3, IL1B, IL8, ING1, ING4, IP6K2, IRF9, ITGB3, JUN, JUND, KDR, KLF13, KLHDC2, KRAS, LIF, LMCD1, LOX, LPL, LTBR, LYN, MAP2K1, MAP2K6, MAP3K1, MYB, MYC, NCL, NFKBIA, NFKBIE, NKX2-5, NR2F1, NR3C1, NR5A2, OAS1, PAX3, PBXIP1, PELI1, PHB, PNPT1, PPARGC1A, PPP1CC, PRDX1, PRKCE, PRMT2, PTEN, PTK2, RARA, RARB, RUNX1, RUNX2, RXRA, SOAT1, SOCS2, SOD2, SP100, SPP1, SRC, SSBP2, TCF4, TCF7L2, TFAP2A, TFDP1, TFF3, TGFA, TNFRSF1A, TNFRSF1B, TNFSF13B, TRADD, TRIB3, TSC22D3, TXLNG, UCN2, VDR, VEGFA, XPC, YWHAG, ZBTB17, ZBTB5	139	yes
Cellular Movement	migration of muscle cells	1.42E-05	Decreased	-2.750	ADM, ANGPT2, APOE, CBL, CCL2, CDKN1B, CLIC4, DUSP1, F2R, FGF2, FOXO1, FOXO4, FURIN, FZD4, GAB1, HBEGF, HMOX1, IGFBP5, IL1B, IL8, ITGB5, KDR, LPAR1, MMP9, MYC, NEO1, NOV, NQO1, PAPPA, PAX3, PLAT, PLAUR, PTGS2, PTK2, RCAN1, ROCK1, RTN4, SDC4, SIX4, SPP1, THBS1, TLR4, TNFSF10, VCAN, WISP2	45	yes
Skeletal and Muscular System Development and Function	migration of muscle cells	1.42E-05	Decreased	-2.750	ADM, ANGPT2, APOE, CBL, CCL2, CDKN1B, CLIC4, DUSP1, F2R, FGF2, FOXO1, FOXO4, FURIN, FZD4, GAB1, HBEGF, HMOX1, IGFBP5, IL1B, IL8, ITGB5, KDR, LPAR1, MMP9, MYC, NEO1, NOV, NQO1, PAPPA, PAX3, PLAT, PLAUR, PTGS2, PTK2, RCAN1, ROCK1, RTN4, SDC4, SIX4, SPP1, THBS1, TLR4, TNFSF10, VCAN, WISP2	45	yes
Cancer	metastasis	1.18E-06	Decreased	-2.744	ABL1, ACTA2, ADAMTS1, AHR, AKAP12, AKT3, ANGPT2, ANGPTL2, ANGPTL4, ANK3, ANXA1, APOE, BIRC5, BMPR1A, C1R, CASP1, CAV1, CBR1, CCL2, CCND2, CD36, CD97, CDC25A, CDK5, CDK5RAP3, CDKN1A, CDKN2C, CEACAM1, CEBPD, CREB3L4, CRYZ, CTGF, CTLA4, CXCR4, DAPK1, DCC, DDB2, DGCR6L, DKK1, DLGAP5, DUSP6, EDNRB, ENPP2, EPAS1, EPHA2, F2R, FABP5, FBN2, FGF2, FOXM1, FST, FSTL3, FYN, FZD7, GART, GDF15, GLRX, GNA12, GREB1, GYG2, HIF1A, HMGB2, HMMR, HMOX1, HOXC6, ICAM1, ID2, IFNAR1, IFNAR2, IGFBP5, IL12A, IL1B, ING4, ITGA6, ITGB3, ITPR3, KDR, KIAA1199, KIDINS220, KIF20A, KIT, KRAS, KRT18, LETMD1, LMCD1, LRRC17, LRRC26, LTBR, MAP2K1, MAP3K5, MCAM, MITF, MLPH, MMP9, MYC, NCAPG, NDRG1, NEDD9, NFKBIA, NQO1, NR2F1, NT5E, NUAK1, PAPPA, PDLIM3, PDZRN3, PGF, PLAT, PLAUR, POLE, PPAP2B, PRNP, PROM1, PRRX1, PTEN, PTGS2, PTK2, PTPRA, RALB, REV3L, RRM1, RUNX2, SEMA4D, SLC16A3, SLC38A6, SMO, SPP1, SSTR2, STEAP3, TGFA, TGFBR2, TGFBR3, THBS1, TIMP3, TM4SF1, TP53BP1, TRIM9, TUBA1C, TUBB3, TUBB4A, TWIST1, TXNIP, VCAN, VEGFA, VEGFB, ZNF536	146	yes
Cancer	germ cell and embryonal neoplasm	2.35E-04	Decreased	-2.736	ABCC1, ABCC4, ARMCX3, ATM, BAX, BCL2, BMI1, BRCA1, CCDC41, CCND1, CDK6, CDKN1A, CDKN2C, CXCR4, DHRS4, FOXD3, FOXO3, FOXO4, FYN, GEMIN5, GLDC, GLI2, H2AFX, IDH1, ING4, KHDRBS3, KIF11, KIT, KRAS, LOXL3, MAP2K1, MDK, MMP9, MYC, NPTX1, OLIG1, PTEN, SFRP1, SMO, SOX2, ST7, STAT2, TUBA1C, TUBB3, TUBB4A, VEGFA, ZBTB17	47	no
Reproductive System Disease	germ cell and embryonal neoplasm	2.35E-04	Decreased	-2.736	ABCC1, ABCC4, ARMCX3, ATM, BAX, BCL2, BMI1, BRCA1, CCDC41, CCND1, CDK6, CDKN1A, CDKN2C, CXCR4, DHRS4, FOXD3, FOXO3, FOXO4, FYN, GEMIN5, GLDC, GLI2, H2AFX, IDH1, ING4, KHDRBS3, KIF11, KIT, KRAS, LOXL3, MAP2K1, MDK, MMP9, MYC, NPTX1, OLIG1, PTEN, SFRP1, SMO, SOX2, ST7, STAT2, TUBA1C, TUBB3, TUBB4A, VEGFA, ZBTB17	47	no
Cellular Movement	migration of breast cancer cell lines	2.11E-06	Decreased	-2.707	ACSL4, ANGPT2, ANKS1A, ANXA1, BCAR3, BHLHE41, CAV1, CCL2, CDKN1B, CHN2, CTSL1, CXCR4, DBF4, EPHA2, EPOR, ETS1, FGF2, FHOD1, FOSL1, FOXO3, GAB1, HIF1A, HMMR, ID1, IGFBP3, IL8, ITGA6, ITGB3, JUN, MAP2K1, MAP4K4, MCAM, MCF2L, MMP9, NDRG1, NRP1, PLAUR, PLXNA1, PPM1F, PRKD1, PTK2, RAB21, RAP1GAP, RNF144A, RUVBL1, SEMA3A, SEMA4D, SLC16A4, SNAI2, SPP1, SRC, SSH1, TFAP2A, TFAP2C, TGFA, TIMP2, VASP, VEGFA, WWTR1	59	yes
Cellular Movement	migration of endothelial cells	2.12E-06	Decreased	-2.697	ADAMTS1, ADM, ANGPT1, ANGPT2, ANGPTL4, ANXA2, APOE, ARHGAP24, ARHGEF6, ARNT, BDNF, CAV1, CCL2, CDKN1B, CTGF, DICER1, DKK1, EFNA1, EFNB2, EYA3, F2RL1, FAP, FGF13, FGF2, FOXO1, FOXO3, GAB1, HAS3, HEY1, HHEX, HIF1A, HMMR, HSP90AB1, HSPB1, ICAM1, ID1, ID3, IGF2R, IGFBP3, IL1B, IL8, ITGA4, ITGB3, KDR, KLF2, KLHL20, MAP2K1, MAP2K3, MAP2K6, MAP3K7, MAPKAPK5, MARCKS, MDK, MMP9, MYC, NOX4, NRP1, PAXIP1, PGF, PLAUR, PLXNA1, PRKCE, PRKD1, PTEN, PTGS2, PTK2, PTPRZ1, RTN4, SCG2, SDC4, SEMA3A, SP100, SPP1, SPRY4, SRC, STC1, TGFBR2, THBS1, TIMP2, TIMP3, TNFRSF12A, TNFSF10, VASP, VEGFA	84	yes
Cardiovascular System Development and Function	migration of endothelial cells	2.12E-06	Decreased	-2.697	ADAMTS1, ADM, ANGPT1, ANGPT2, ANGPTL4, ANXA2, APOE, ARHGAP24, ARHGEF6, ARNT, BDNF, CAV1, CCL2, CDKN1B, CTGF, DICER1, DKK1, EFNA1, EFNB2, EYA3, F2RL1, FAP, FGF13, FGF2, FOXO1, FOXO3, GAB1, HAS3, HEY1, HHEX, HIF1A, HMMR, HSP90AB1, HSPB1, ICAM1, ID1, ID3, IGF2R, IGFBP3, IL1B, IL8, ITGA4, ITGB3, KDR, KLF2, KLHL20, MAP2K1, MAP2K3, MAP2K6, MAP3K7, MAPKAPK5, MARCKS, MDK, MMP9, MYC, NOX4, NRP1, PAXIP1, PGF, PLAUR, PLXNA1, PRKCE, PRKD1, PTEN, PTGS2, PTK2, PTPRZ1, RTN4, SCG2, SDC4, SEMA3A, SP100, SPP1, SPRY4, SRC, STC1, TGFBR2, THBS1, TIMP2, TIMP3, TNFRSF12A, TNFSF10, VASP, VEGFA	84	yes
Cellular Movement	chemotaxis of endothelial cells	3.34E-04	Decreased	-2.689	ANGPT1, ANGPT2, CCL2, CD36, CEACAM1, CXCR4, EFNA1, ENPP2, FGF2, IGF2R, IL8, KDR, NPTX1, PTGS2, SEMA4D, THBS1, THBS2, VEGFA	18	yes
Cardiovascular System Development and Function	chemotaxis of endothelial cells	3.34E-04	Decreased	-2.689	ANGPT1, ANGPT2, CCL2, CD36, CEACAM1, CXCR4, EFNA1, ENPP2, FGF2, IGF2R, IL8, KDR, NPTX1, PTGS2, SEMA4D, THBS1, THBS2, VEGFA	18	yes
Cellular Movement	invasion of cells	5.52E-12	Decreased	-2.651	ABL1, ABL2, ACAT1, ACSL4, ADAMTS1, ADM, AKT3, AMD1, ANG, ANGPT2, ANXA1, ARAP3, ATP6V0A4, B4GALT5, BATF3, BAX, BCAN, BCAR3, BCL2, BDNF, BHLHE40, BHLHE41, CALR, CAV1, CBL, CCL2, CCNA2, CCND1, CCR1, CD97, CDC25B, CDK5, CDK5R1, CDK5RAP3, CDKN1B, CEACAM1, CHD1, CITED1, CLCA2, CLDN1, CMTM8, CSPG4, CTGF, CTSK, CTSL1, CXCR4, DAB2, DIAPH2, DIAPH3, DKK1, DKK3, DNAJB4, E2F5, EDNRB, EFNA1, ENPP2, EPHA2, ETS1, ETS2, ETV1, ETV4, ETV5, F2R, FABP5, FAS, FGF13, FGF2, FHOD1, FOSL1, FOXD3, FOXM1, FOXO3, FST, FSTL1, FURIN, FYN, GAB1, GBP1, GDF15, GJB1, GNA12, GNAL, HAS2, HAS3, HAX1, HBEGF, HBP1, HHEX, HIF1A, HIPK2, HMGA1, HMMR, HMOX1, ID1, ID2, IER3, IL1B, IL8, IRS2, ITGA4, ITGA6, ITGB3, JAM3, JUN, JUND, KLF4, KRAS, LAMC1, LDLRAP1, LEF1, LGMN, LIMK1, LOX, LPAR1, LRIG1, LRP5, LYN, MAP2K1, MAP2K5, MAP2K6, MAP3K11, MAP4K4, MARCKS, MCAM, MIA, MITF, MMP16, MMP9, MRAS, MST1, MYC, MYLK, NAMPT, NCOA4, NDRG1, NEDD9, NFKBIA, NOV, NQO1, NRCAM, NRP1, NUAK1, PA2G4, PAK4, PAPPA, PARVB, PLAT, PLAUR, PLGRKT, PLXNB1, POU3F2, PPM1F, PRKCE, PTEN, PTGS2, PTK2, PTP4A1, PTP4A2, RALA, RAP1GAP, RND3, RNF144A, ROCK1, RPRD1A, RUNX2, RUNX3, RUVBL1, SDC4, SEMA4D, SFRP1, SIM2, SLC2A1, SNAI2, SOD2, SP100, SPHK1, SPP1, SPRY2, SRC, SSTR2, ST8SIA1, STMN1, SYK, TBX2, TFAP2A, TFAP2C, TFF3, TGFA, TGFBR2, TGFBR3, THBS1, THBS2, TIMP2, TIMP3, TNFSF10, TWIST1, VASP, VCAN, VDR, VEGFA, VEGFB, WISP2, WWTR1, ZEB2	204	yes

Cell Cycle	S phase	6.17E-06	Decreased	-2.618	ABL1, ANGPT1, ANP32B, ATM, BCL2, BHLHE40, BID, BIRC5, BRCA1, CAV1, CCNA2, CCND1, CCND2, CCNG2, CDC25A, CDC25C, CDC6, CDK6, CDKN1A, CDKN1B, CDKN2C, CEBPB, CTLA4, DBF4, E2F2, E2F3, E2F5, EPOR, FEN1, FGF2, FOS, FOXM1, FOXO3, GAB1, GAS1, GTF2I, HBEGF, HINFP, HMGA1, HMOX1, ID1, ID2, ID3, IL1B, ILKAP, ING4, JUN, KLF4, KRAS, LZTS1, MAP2K1, MCM10, MCMBP, MEIS2, MIA, MXD3, MXD4, MXI1, MYC, NRIP1, PAM16, PMEPA1, PNPT1, RBBP8, REV3L, RUNX1, SH2B3, SLBP, SOD2, SRC, ST8SIA1, TFDP1, TGFA, TGFBR2, TIMELESS, TP53BP1, UHRF1, VEGFA, VPS18	79	yes
Cellular Movement	migration of vascular endothelial cells	1.19E-05	Decreased	-2.574	ADAMTS1, ADM, ANGPT1, ANGPT2, ANXA2, ARHGEF6, CDKN1B, DICER1, DKK1, EFNA1, EFNB2, F2RL1, FGF13, FGF2, FOXO1, FOXO3, GAB1, HHEX, HIF1A, HSP90AB1, ID1, ID3, ITGB3, KDR, KLF2, KLHL20, MAP3K7, MAPKAPK5, MDK, MMP9, NRP1, PTEN, PTGS2, PTPRZ1, RTN4, SP100, SPRY4, SRC, TGFBR2, TIMP2, TNFSF10, VEGFA	42	yes
Cardiovascular System Development and Function	migration of vascular endothelial cells	1.19E-05	Decreased	-2.574	ADAMTS1, ADM, ANGPT1, ANGPT2, ANXA2, ARHGEF6, CDKN1B, DICER1, DKK1, EFNA1, EFNB2, F2RL1, FGF13, FGF2, FOXO1, FOXO3, GAB1, HHEX, HIF1A, HSP90AB1, ID1, ID3, ITGB3, KDR, KLF2, KLHL20, MAP3K7, MAPKAPK5, MDK, MMP9, NRP1, PTEN, PTGS2, PTPRZ1, RTN4, SP100, SPRY4, SRC, TGFBR2, TIMP2, TNFSF10, VEGFA	42	yes
Cellular Movement	invasion of tumor cell lines	9.91E-12	Decreased	-2.567	ABL1, ABL2, ACAT1, ACSL4, ADAMTS1, ADM, AKT3, ANXA1, ARAP3, ATP6V0A4, BATF3, BCAN, BCAR3, BCL2, BDNF, BHLHE41, CALR, CAV1, CBL, CCL2, CCNA2, CCR1, CD97, CDC25B, CDK5, CDK5R1, CDK5RAP3, CDKN1B, CHD1, CMTM8, CSPG4, CTGF, CTSK, CXCR4, DAB2, DIAPH2, DIAPH3, DKK1, DKK3, DNAJB4, E2F5, EFNA1, ENPP2, EPHA2, ETS1, ETS2, ETV1, ETV4, ETV5, FABP5, FAS, FGF2, FHOD1, FOSL1, FOXD3, FOXM1, FURIN, GDF15, GJB1, GNA12, GNAL, HAS2, HAS3, HAX1, HBEGF, HBP1, HIF1A, HIPK2, HMGA1, HMMR, HMOX1, ID1, ID2, IL8, IRS2, ITGA4, ITGB3, JAM3, JUN, JUND, KLF4, KRAS, LDLRAP1, LEF1, LIMK1, LOX, LPAR1, LRIG1, LRP5, LYN, MAP4K4, MARCKS, MCAM, MIA, MITF, MMP16, MMP9, MST1, NAMPT, NDRG1, NFKBIA, NOV, NRP1, NUAK1, PA2G4, PAK4, PAPPA, PARVB, PLAT, PLAUR, PLGRKT, PLXNB1, POU3F2, PPM1F, PRKCE, PTEN, PTGS2, PTK2, PTP4A2, RALA, RAP1GAP, RND3, RNF144A, ROCK1, RUVBL1, SDC4, SFRP1, SIM2, SLC2A1, SNAI2, SOD2, SP100, SPHK1, SPP1, SPRY2, SRC, SSTR2, ST8SIA1, STMN1, TFAP2A, TFAP2C, TFF3, TGFA, TGFBR2, TIMP2, TIMP3, TNFSF10, TWIST1, VCAN, VEGFA, VEGFB, WWTR1, ZEB2	153	yes
Cancer	neoplasia of cells	1.06E-07	Decreased	-2.536	ABL1, ADAMTS1, ADAMTS9, ANG, ANGPT2, ANGPTL4, ARMC10, BAP1, BAX, BCDIN3D, BCL2, BCL3, BECN1, BMPR1A, CAV1, CBR1, CCL2, CCND1, CDC25A, CDKN1A, CKS1B, CREB3L4, CSNK1E, CTSL1, CXCR4, DAPK1, DCC, DGCR8, DICER1, DKK1, DNAJB4, DYNLL1, EGR1, ENPP2, EPAS1, ETS1, ETV1, F2R, FABP5, FADD, FGF2, FOS, FYN, HAS2, HIF1A, HMOX1, IFNAR1, IL27RA, IL8, ING1, KIT, KRAS, LRRC26, LZTS1, MAP2K1, MCAM, MMP9, MYC, NFKBIA, NOX4, NUPR1, PLAUR, PPAP2A, PPAP2B, PRKAR1A, PRNP, PROM1, PTEN, PTGES, PTK2, PTPRA, RALA, RALB, RIT1, RRM1, RUNX1, RUNX2, RUNX3, RYK, SEMA4D, SMO, SNAI2, SOD2, SOX2, SPHK1, SPRY2, TGFA, TGFBR2, THBS1, TIMP3, TP53BP1, TPD52, TWIST1, TXNIP, VEGFA	95	no
Cellular Movement	migration of tumor cell lines	3.89E-11	Decreased	-2.483	ACSL4, AFAP1, AKAP11, AKT3, ANGPT2, ANKS1A, ANXA1, ANXA2, AP2M1, BCAR3, BHLHE41, CAV1, CCL2, CCL20, CD36, CD97, CDC25B, CDK5, CDKN1B, CHN2, CKLF, CMTM8, CRKL, CTGF, CTSL1, CXCR4, DAB2, DBF4, DCBLD2, DEFB4A/DEFB4B, DGCR6L, E2F5, EGR1, ENPP2, EPHA2, EPOR, ETS1, ETV4, F2RL1, FBXL2, FGF2, FHOD1, FOSL1, FOXD3, FOXM1, FOXO3, FYN, GAB1, GNA13, GOLGA2, HAS2, HAS3, HAX1, HBEGF, HDAC6, HIF1A, HMMR, HMOX1, ID1, IGFBP3, IL8, ING4, ITGA4, ITGA6, ITGB3, JUN, KANK1, KDR, KIDINS220, KLF2, KLF4, KRAS, LAMB3, LIMK1, LMCD1, LOX, LPAR1, LYN, MAP2K1, MAP3K1, MAP3K7, MAP4K4, MC1R, MCAM, MCF2L, MDK, MERTK, MITF, MMP9, MSN, MST1, MYO10, NDRG1, NEDD9, NFKBIA, NOV, NREP, NRP1, PHB, PIK3C2B, PLAUR, PLCL1, PLGRKT, PLXNA1, PLXNB1, PPFIA1, PPM1F, PREX1, PRKD1, PRNP, PTEN, PTGS2, PTK2, PTP4A2, PTPN12, PTPRM, PTPRZ1, PVR, RAB21, RALA, RALB, RAP1GAP, RARRES1, RGS1, RNF144A, RUNX2, RUVBL1, SCNN1A, SEMA3A, SEMA4D, SH3PXD2B, SHC4, SLC16A4, SLC2A1, SNAI2, SOD2, SPHK1, SPP1, SPRY2, SRC, SSH1, ST6GAL1, STMN1, SYK, SYNM, TFAP2A, TFAP2C, TGFA, THBS1, THBS2, TIMP2, TLR4, TNC, TNFSF10, TP53INP1, TRIP6, TWIST1, VASP, VEGFA, VEGFB, VPS28, WWTR1, ZYX	163	yes
Cellular Development	branching of endothelial cell lines	3.94E-04	Decreased	-2.425	ADM, ETS1, FADD, FGF2, LAMA4, VEGFA	6	yes
Cell Morphology	branching of endothelial cell lines	3.94E-04	Decreased	-2.425	ADM, ETS1, FADD, FGF2, LAMA4, VEGFA	6	yes
Cardiovascular System Development and Function	branching of endothelial cell lines	3.94E-04	Decreased	-2.425	ADM, ETS1, FADD, FGF2, LAMA4, VEGFA	6	yes
Cancer	neoplasia of stomach	4.05E-04	Decreased	-2.425	ABL1, AHR, AIM2, ANXA1, APOE, ARAP3, BAX, BIRC5, BRCA1, CASP1, CAV1, CCND1, CD58, CDKN1A, CDKN1B, CDKN2C, COBLL1, DAPK1, DKK1, EPOR, ETV4, FOXM1, GSTM1, HMGA2, HMGCR, IGF2R, IGFBP3, IL1B, IL8, KDR, KRAS, LZTS1, MAL, MDK, MIA, MMP9, MTHFR, MYC, NAMPT, NQO1, NR3C1, NRIP1, NUDT1, PA2G4, PDK4, PHB, PTEN, PTGS2, RNF103, SEC31A, SIAH1, SIRT2, SLC16A4, SOX2, SPP1, TGFBR2, TLR4, TTK, TUBA1C, TUBB3, TUBB4A, UBE2C, UGT2B7	63	no
Gastrointestinal Disease	neoplasia of stomach	4.05E-04	Decreased	-2.425	ABL1, AHR, AIM2, ANXA1, APOE, ARAP3, BAX, BIRC5, BRCA1, CASP1, CAV1, CCND1, CD58, CDKN1A, CDKN1B, CDKN2C, COBLL1, DAPK1, DKK1, EPOR, ETV4, FOXM1, GSTM1, HMGA2, HMGCR, IGF2R, IGFBP3, IL1B, IL8, KDR, KRAS, LZTS1, MAL, MDK, MIA, MMP9, MTHFR, MYC, NAMPT, NQO1, NR3C1, NRIP1, NUDT1, PA2G4, PDK4, PHB, PTEN, PTGS2, RNF103, SEC31A, SIAH1, SIRT2, SLC16A4, SOX2, SPP1, TGFBR2, TLR4, TTK, TUBA1C, TUBB3, TUBB4A, UBE2C, UGT2B7	63	no
Cancer	growth of tumor	2.03E-07	Decreased	-2.414	ABCC1, ABL1, ADAMTS1, ADM, AHR, AKT3, ANG, ANGPT2, ANGPTL4, ANXA2, ATM, BCL2, BECN1, BIRC5, BMI1, BRCA1, CALR, CASP1, CCL2, CCR1, CD36, CD59, CDK5, CDKN1A, CDKN1B, CEACAM1, CENPA, CTLA4, DAB2IP, DAPK1, DKK1, EFNA1, EFNB2, EPAS1, EPHA2, F2R, FADD, FAS, FCER1G, FGF2, FGFR3, FOS, FOXD3, FOXM1, FOXO1, FRMD6, FTH1, GJB1, HBEGF, HIF1A, HMGA1, HMGCR, HMMR, HMOX1, IFNAR1, IGFBP5, IL12A, IL1B, IL27RA, ITGB3, ITGB5, JAM3, KDR, KIDINS220, KRAS, LAMA4, LAMB1, LIF, LTBR, MAP2K1, MAPKAPK5, MCL1, MMP9, MYB, MYC, NCAPG, NDRG1, NEDD9, NFKBIA, NOLC1, NRP1, NT5E, NUAK1, PGF, PTEN, PTGES, PTGS2, RAB27A, RALB, RUNX1, RUNX2, SAT1, SLC16A3, SMO, SOD2, SPP1, SRC, SSTR2, TACC2, TCF4, TGFBR2, THBS1, TIMP2, TLR4, TNC, TNFSF10, TPR, TPX2, TSC22D1, TUBB3, VEGFA	111	no
Cancer	Pancreatic Cancer and Tumors	8.10E-05	Decreased	-2.414	ADAMTS1, AHR, ANXA1, BAX, BIRC5, BNIP3, BRCA1, CASP1, CASP4, CCND1, CD59, CD74, CDK5, CDK5R1, CDKN1A, CDKN1B, CDKN2C, CXCR4, DCLK1, DKK1, EPHA2, FBN2, FXYD3, GDF15, HBEGF, HIF1A, HLA-DRA, HOXB2, ID1, IGFBP3, IL1B, KDR, KIT, KRAS, LAMA4, MMP9, MYB, MYC, NPTX1, NR5A2, PDK4, PLAT, PLAUR, POLE, PRKD1, PROM1, PTEN, PTGES, PTGS2, PTK2, RARA, RRM1, SPP1, SRC, SSTR2, TLR4, TP53INP1, TPM2, TTLL1, TWIST1, VEGFA	61	no
Gastrointestinal Disease	Pancreatic Cancer and Tumors	8.10E-05	Decreased	-2.414	ADAMTS1, AHR, ANXA1, BAX, BIRC5, BNIP3, BRCA1, CASP1, CASP4, CCND1, CD59, CD74, CDK5, CDK5R1, CDKN1A, CDKN1B, CDKN2C, CXCR4, DCLK1, DKK1, EPHA2, FBN2, FXYD3, GDF15, HBEGF, HIF1A, HLA-DRA, HOXB2, ID1, IGFBP3, IL1B, KDR, KIT, KRAS, LAMA4, MMP9, MYB, MYC, NPTX1, NR5A2, PDK4, PLAT, PLAUR, POLE, PRKD1, PROM1, PTEN, PTGES, PTGS2, PTK2, RARA, RRM1, SPP1, SRC, SSTR2, TLR4, TP53INP1, TPM2, TTLL1, TWIST1, VEGFA	61	no
Endocrine System Disorders	Pancreatic Cancer and Tumors	8.10E-05	Decreased	-2.414	ADAMTS1, AHR, ANXA1, BAX, BIRC5, BNIP3, BRCA1, CASP1, CASP4, CCND1, CD59, CD74, CDK5, CDK5R1, CDKN1A, CDKN1B, CDKN2C, CXCR4, DCLK1, DKK1, EPHA2, FBN2, FXYD3, GDF15, HBEGF, HIF1A, HLA-DRA, HOXB2, ID1, IGFBP3, IL1B, KDR, KIT, KRAS, LAMA4, MMP9, MYB, MYC, NPTX1, NR5A2, PDK4, PLAT, PLAUR, POLE, PRKD1, PROM1, PTEN, PTGES, PTGS2, PTK2, RARA, RRM1, SPP1, SRC, SSTR2, TLR4, TP53INP1, TPM2, TTLL1, TWIST1, VEGFA	61	no
Cellular Movement	migration of cells	2.68E-11	Decreased	-2.406	ABCA1, ABCC1, ABL1, ABL2, ABR, ACSL4, ACVR1, ADAM19, ADAMTS1, ADD1, ADM, ADORA2B, ADRBK1, AFAP1, AHR, AKAP11, AKT3, ANGPT1, ANGPT2, ANGPTL2, ANGPTL4, ANKS1A, ANXA1, ANXA2, AP2M1, APOE, ARAP3, ARHGAP24, ARHGEF1, ARHGEF6, ARID5B, ARNT, ATM, BAG3, BAX, BCAR3, BCL2, BDNF, BHLHE40, BHLHE41, BID, BMPR1A, BRCA1, BTK, C1GALT1, C2, C3AR1, CADPS2, CALR, CASP1, CAV1, CBL, CCL2, CCL20, CCL28, CCNA2, CCNB2, CCND1, CCR1, CD2, CD200, CD276, CD36, CD58, CD74, CD97, CD99L2, CDC25B, CDK5, CDK5R1, CDKN1A, CDKN1B, CDKN2C, CEACAM1, CFB, CFH, CHN2, CHST2, CKLF, CLCA2, CLIC4, CMTM8, COL4A3BP, CPB2, CR2, CRKL, CSPG4, CTGF, CTLA4, CTSE, CTSL1, CXCL13, CXCL16, CXCR4, CYTH2, DAB2, DBF4, DCBLD2, DCC, DCLK1, DCX, DEFB4A/DEFB4B, DGCR6L, DIAPH2, DICER1, DKK1, DLX1, DUSP1, E2F3, E2F5, EDNRB, EFNA1, EFNB2, EGLN1, EGR1, EGR2, ELK3, ENPP2, EPAS1, EPHA2, EPOR, ERRFI1, ETS1, ETV4, ETV5, EYA3, F2R, F2RL1, FAP, FAS, FBXL2, FBXO45, FCER1G, FCGR2A, FGF13, FGF2, FHOD1, FKBP4, FOSL1, FOXD3, FOXM1, FOXO1, FOXO3, FOXO4, FTH1, FURIN, FYB, FYN, FZD4, GAB1, GAL3ST1, GJB1, GLI2, GLRX, GNA12, GNA13, GNG12, GOLGA2, GPR183, GTPBP4, HAS2, HAS3, HAX1, HBEGF, HDAC6, HEY1, HHEX, HIF1A, HMMR, HMOX1, HOXB2, HSP90AB1, HSPB1, HSPD1, ICAM1, ID1, ID2, ID3, IFNAR1, IGF2R, IGFBP3, IGFBP5, IL11RA, IL12A, IL16, IL1B, IL27RA, IL8, ING4, IRF5, IRS2, ITGA4, ITGA6, ITGB3, ITGB5, ITK, JAM3, JUN, KANK1, KDR, KIDINS220, KIT, KLF2, KLF4, KLHL20, KRAS, LAMA2, LAMB1, LAMB3, LAMC1, LEF1, LGMN, LHX6, LIMA1, LIMK1, LMCD1, LMO4, LOX, LPAR1, LRP5, LRP8, LTBR, LYN, MAP2K1, MAP2K3, MAP2K5, MAP2K6, MAP3K1, MAP3K12, MAP3K5, MAP3K7, MAP4K4, MAPKAPK5, MARCKS, MARCKSL1, MATN2, MC1R, MCAM, MCF2L, MCM2, MDK, MERTK, MESP1, MGLL, MIA, MITF, MLLT11, MMP9, MPP1, MRAS, MSN, MST1, MYC, MYLK, MYO10, MYO9A, NAMPT, NCKIPSD, NCL, NDRG1, NEDD9, NEO1, NFIA, NFKBIA, NLRP3, NOV, NOX4, NPC1, NQO1, NR2F1, NR4A2, NREP, NRP1, NRTN, NT5E, ONECUT2, OVOL2, P2RX7, PA2G4, PACSIN2, PAK4, PAPPA, PAX3, PAXIP1, PDE4B, PEX11B, PEX2, PEX5, PGF, PHB, PIK3C2B, PIK3CD, PIK3IP1, PLAT, PLAUR, PLCL1, PLGRKT, PLP1, PLXNA1, PLXNB1, PMP22, POU3F2, PPAP2A, PPAP2B, PPFIA1, PPM1F, PPP1R15A, PRDX1, PREX1, PRKAR1A, PRKCE, PRKD1, PRMT2, PRNP, PTEN, PTGDS, PTGES, PTGS2, PTK2, PTP4A1, PTP4A2, PTPN12, PTPN14, PTPRA, PTPRM, PTPRZ1, PVR, RAB21, RAB27A, RAC2, RALA, RALB, RAP1GAP, RAP2A, RAPGEF1, RARA, RARRES1, RCAN1, REPS2, RGS1, RNF144A, ROCK1, ROCK2, RPRD1A, RRAS, RTN4, RUNX1, RUNX2, RUNX3, RUVBL1, RXRA, SARS, SCG2, SCHIP1, SCNN1A, SCPEP1, SDC3, SDC4, SEMA3A, SEMA3C, SEMA4D, SEMA6A, SFRP1, SH2B3, SH3BP1, SH3PXD2B, SHC4, SIX4, SLC16A4, SLC2A1, SLC3A2, SMO, SMPD1, SMPD2, SNAI2, SNX17, SOD2, SORBS3, SORT1, SOX8, SP100, SPHK1, SPP1, SPRY2, SPRY4, SRC, SRGAP1, SSH1, ST3GAL4, ST3GAL6, ST6GAL1, STC1, STIM1, STK24, STK4, STMN1, SYK, SYNM, TFAP2A, TFAP2C, TGFA, TGFBR2, THBS1, THBS2, TIAM2, TIMP2, TIMP3, TIRAP, TLR4, TM4SF1, TNC, TNFRSF12A, TNFRSF1A, TNFRSF1B, TNFSF10, TNFSF13B, TNS3, TP53INP1, TRAF3IP2, TRIM32, TRIP6, TRPV2, TUBB2B, TWIST1, TXK, VASP, VCAN, VDR, VEGFA, VEGFB, VPS18, VPS28, WASL, WISP2, WWTR1, YARS, ZBTB24, ZEB2, ZFAND5, ZP3, ZYX	441	yes
Cell Cycle	entry into S phase of connective tissue cells	2.64E-04	Decreased	-2.356	ATM, CCND1, CDKN1A, CDKN1B, E2F2, E2F3, E2F5, FGF2, ID3, MYC, TFDP1	11	yes
Cancer	quantity of tumor	4.98E-04	Decreased	-2.342	CCND1, CDKN1A, CDKN1B, CDKN2C, DCC, EDNRB, FOXM1, GJB1, HIF1A, HIP1, HIPK1, HMMR, HPGD, ITGB3, JUN, KIT, KRAS, MAP2K1, MMP9, MST1, MYC, NQO1, NT5E, PARP4, PRKCE, PTEN, RARA, RUNX1, SAT1, SPHK1, TAF4, TGFA, TGFBR2, TP53INP1, XPC	35	no
Connective Tissue Development and Function	quantity of fibroblast cell lines	7.12E-04	Decreased	-2.333	BECN1, CAV1, CCNA2, CCND1, CDKN1A, DYNLL1, ERRFI1, FAS, GNA12, NOX4, PTGS2, RBBP8, SPHK1, TNFSF10	14	no
Tissue Morphology	quantity of fibroblast cell	7.12E-04	Decreased	-2.333	BECN1, CAV1, CCNA2, CCND1, CDKN1A, DYNLL1, ERRFI1, FAS, GNA12, NOX4, PTGS2, RBBP8, SPHK1, TNFSF10	14	no
Cell Morphology	cell spreading	1.70E-04	Decreased	-2.312	ANGPT1, ARAP1, ARHGAP24, ATRN, BTK, CBL, CRKL, CSPG4, DAB2, DIAPH2, EDNRB, EFNA1, EPHA2, ETS1, FCGR2A, FGF2, IGF2R, IGFBP5, IL8, ING4, IRAK1, ITGA4, ITGB3, LAMA2, LPAR1, LPXN, LYN, MARCKS, MMP9, MYO10, NEDD9, PACSIN2, PAK4, PARVB, PMP22, PPFIA1, PRKCE, PRKD1, PRNP, PTBP1, PTK2, PTPN12, PTPN14, PTPRA, PVR, RAP1GAP, RAP2A, RAPGEF1, RRBP1, RTN4, SDC4, SEMA3A, SMPD1, SMPD2, SNAI2, SORBS1, SORBS3, SPHK1, SPP1, SPRY2, SRC, SYK, SYNM, THBS1, THBS2, TNC, VASP, VEGFA, WASL, ZYX	70	yes
Cellular Movement	cell movement of prostate cancer cell lines	3.22E-04	Decreased	-2.307	AFAP1, ANXA2, CAV1, CXCR4, DBF4, EFNA1, EPHA2, GDF15, HMOX1, ID1, IL8, ITGB3, KDR, LAMB3, MAP4K4, NDRG1, NRP1, PA2G4, PHB, PRKCE, PRKD1, RALB, RARRES1, TBXAS1, TNFSF10, TWIST1, VEGFA	27	yes
Cellular Movement	cell movement of fibroblast cell lines	1.08E-05	Decreased	-2.303	ABL1, AMD1, BDNF, CCNA2, CDKN1A, CDKN1B, CLIC4, CRKL, ELK3, FGF2, FOS, FOSL1, GAB1, GNA13, GNG12, HAS2, HAS3, HAX1, HBEGF, IL8, KLF2, LIMA1, LOX, MARCKS, MMP9, MST1, MTSS1, MYLK, NOX4, NRCAM, PLAUR, PRKD1, PTEN, PTGDS, PTGS2, PTK2, PTPN12, PTPRA, PVR, RAC2, SPP1, SRC, TP53INP1, TRIM32, VEGFA, WASL	46	yes
Connective Tissue Development and Function	cell movement of fibroblast cell lines	1.08E-05	Decreased	-2.303	ABL1, AMD1, BDNF, CCNA2, CDKN1A, CDKN1B, CLIC4, CRKL, ELK3, FGF2, FOS, FOSL1, GAB1, GNA13, GNG12, HAS2, HAS3, HAX1, HBEGF, IL8, KLF2, LIMA1, LOX, MARCKS, MMP9, MST1, MTSS1, MYLK, NOX4, NRCAM, PLAUR, PRKD1, PTEN, PTGDS, PTGS2, PTK2, PTPN12, PTPRA, PVR, RAC2, SPP1, SRC, TP53INP1, TRIM32, VEGFA, WASL	46	yes

Cellular Development	differentiation of cells	5.84E-09	Decreased	-2.293	ABCC4, ABI1, ABL1, ABL2, ACACA, ACVR1, ADAMTS9, ADD1, ADK, ADM, ADORA2B, ADRBK1, AFF1, AHCTF1, AHR, AMD1, ANG, ANGPT1, ANGPT2, ANGPTL2, ANKRD54, ANXA1, ANXA2, ARID2, ARID5B, ARL4A, ARNT, ASPA, ATM, ATP7A, BAIAP2, BATF, BATF3, BAX, BCL2, BCL2L11, BCL3, BDNF, BECN1, BHLHE40, BHLHE41, BMI1, BMP1, BMPR1A, BNIP3, BRCA1, BTG1, BTG3, BTK, C14orf169, C3AR1, CADPS2, CALR, CAV1, CBL, CBY1, CCL2, CCND1, CCR1, CD2, CD200, CD276, CD36, CD74, CDC25A, CDK5, CDK5R1, CDK5RAP3, CDK6, CDKN1A, CDKN1B, CDKN2C, CEACAM1, CEBPB, CEBPD, CHD1, CHD7, CHKA, CITED1, CLIC1, CLIC4, CMTM8, COL11A2, CR2, CREB3L2, CREB5, CSDA, CTDSPL, CTGF, CTHRC1, CTLA4, CTNNBIP1, CTSE, CTSK, CXCR4, CYTL1, DAB2, DCX, DDIT3, DEDD, DEF6, DHCR7, DICER1, DISP1, DKK1, DLK2, DLL3, DLX1, DSP, DUSP1, DUSP5, DVL2, DYRK3, E2F2, EDNRB, EFNA1, EFNB2, EGR1, EGR2, EGR3, ELF1, ELK4, ENC1, EOMES, EPAS1, EPHA2, EPHA4, EPOR, ERO1L, ERRFI1, ETS1, ETS2, ETV4, ETV5, EXOC6, EXTL1, FABP5, FADD, FAM20C, FAS, FCER1G, FEZ1, FGF2, FGFR3, FLI1, FNDC3B, FOS, FOSL1, FOXD3, FOXM1, FOXO1, FOXO3, FOXO4, FST, FSTL3, FYN, FZD1, FZD2, FZD7, GAB1, GAS1, GDPD5, GHR, GJA3, GLI2, GNA12, GNA13, GNA15, GNAL, GNL3, GPC4, GPSM2, GRAP2, HAS2, HBEGF, HBP1, HDAC7, HES1, HEY1, HEY2, HHEX, HIF1A, HINFP, HIST1H4A (includes others), HIVEP2, HMG20B, HMGA2, HMGB2, HMGB3, HMOX1, HOXA5, HOXB13, HOXB2, HOXD11, HPS4, HSF4, HSP90AB1, HSPA2, HSPD1, HTR7, ICAM1, ID1, ID2, ID3, IFI16, IFNAR1, IFNGR2, IGF2R, IGFBP3, IGFBP5, IKZF4, IL11RA, IL12A, IL17D, IL1B, IL1RAP, IL1RAPL1, IL27RA, IL7R, IL8, IRF4, IRF5, IRS2, IRX3, ITGA6, ITGB3, ITGB5, ITK, ITPKB, JAM3, JUN, JUND, KBTBD10, KDR, KIT, KLF13, KLF15, KLF4, KLF9, KRAS, L3MBTL1, LAMA2, LAMA4, LAMB3, LAMC1, LDB1, LEF1, LIF, LIMK1, LIMS1, LMO4, LPIN1, LRIG1, LRP5, LRP8, LRRC17, LTBP4, LTBR, LYN, MAF, MAFF, MAFG, MAGED1, MAL, MALT1, MAP2K1, MAP2K3, MAP2K6, MAP3K12, MAP3K5, MAP3K7, MBP, MC1R, MCC, MCL1, MDK, MEF2C, MERTK, MESP1, MITF, MKS1, MLLT11, MLPH, MMP9, MRAS, MST1, MVK, MXD3, MYB, MYC, MYO5A, N4BP2L2, NAMPT, NDRG1, NFIA, NFIB, NFKBIA, NKX2-5, NMRK2, NOC3L, NOV, NOX4, NR2F1, NR3C1, NR4A2, NREP, NRP1, NRTN, OCA2, OLIG1, ONECUT2, OSBPL9, OTX1, OVOL2, PA2G4, PAK4, PARP2, PAX3, PBXIP1, PCSK1, PDK4, PER3, PEX11B, PGF, PIK3CD, PIM3, PKDCC, PKNOX1, PLAT, PLAUR, PLCL1, PLP1, PMP22, PNP, POU3F1, POU3F2, PPARGC1A, PPP1CC, PPP1R13L, PRAME, PRKAR1A, PRKD1, PRMT5, PRNP, PTEN, PTGDS, PTGS2, PTK2, PTPRA, PTPRE, PTPRZ1, QKI, RAB27A, RAC2, RACGAP1, RARA, RARB, RARRES3, RASA3, RASSF2, RBM38, RIT1, RND3, ROCK1, ROCK2, ROGDI, RQCD1, RRAS, RTN4, RUNX1, RUNX2, RUNX3, RXRA, RYK, SACS, SARS, SATB1, SDC3, SELENBP1, SEMA3A, SEMA4D, SFRP1, SFXN1, SH2B3, SH3PXD2A, SH3PXD2B, SHB, SIK3, SLC3A2, SMAD6, SMARCA1, SMO, SNAI2, SOCS2, SOD2, SORT1, SOX2, SOX4, SOX6, SOX8, SPEN, SPHK1, SPP1, SPRED1, SPRED2, SPRY1, SPRY2, SPRY4, SRC, SSBP2, ST8SIA1, STAT2, STC1, STIM1, STK39, STK4, STMN1, SYK, TACC3, TBX2, TCF4, TCF7L2, TFAP2A, TFAP2C, TGFA, TGFBR2, TGIF1, THBS1, THBS2, TIMP2, TIMP3, TIRAP, TLR4, TMEM67, TNC, TNFRSF12A, TNFRSF1A, TNFRSF1B, TNFSF10, TNFSF13B, TRIB3, TRIM24, TRIM32, TRPM1, TSC22D3, TUBB3, TWIST1, TXK, TXNIP, TYRP1, UBD, ULK1, UPK1A, VASP, VCAN, VDR, VEGFA, VGF, WWTR1, XBP1, YWHAG, ZBTB17, ZFP36, ZIC2, ZNF536	465	yes
Cancer	head and neck cancer	4.90E-07	Decreased	-2.293	ABL1, ACTA2, ADAMTS1, ADAMTS9, ADSL, AKT3, ALAD, ANGPT2, ANO1, ANXA1, ANXA2, ARHGEF2, ATM, BAX, BCCIP, BCL2, BCL3, BDNF, BMI1, BRCA1, CALCOCO2, CASP4, CAV1, CCDC41, CCNB2, CCND1, CDK6, CDKN1A, CDKN1B, CDKN2C, CEBPD, CHKA, CHRM1, CHRNB1, CXCL16, CXCR4, DAPK1, DHRS4, DKC1, DVL2, E2F3, ECE2, EFNB2, EGR1, EIF2C2, ENC1, EPHA2, EPS15, ETS1, FABP3, FABP5, FADD, FASN, FOS, FOXM1, FOXO3, FOXO4, FTH1, FURIN, FZD7, GEMIN5, GLI2, GNA12, GNB4, GSTM1, HIPK2, HMGA1, HMGA2, HMGB2, HMOX1, HSP90AB1, ID1, IDH1, IGFBP3, IGFBP5, IL8, ING1, JUN, KDR, KHDRBS3, KIT, KRAS, KRT18, LAMA4, LAMB3, LMO4, LPAR1, LTBR, LYN, MAP4K4, MARCKS, MCL1, MCM2, MIA, MINPP1, MMP9, MSMO1, MTHFR, MYC, NCOA4, NFIB, NPTX1, NQO1, NR3C1, NRIP1, NUSAP1, NXT1, OLIG1, PAM16, PCM1, PGF, PINK1, PLAT, PPFIA1, PPIL1, PRKAR1A, PRKCE, PROM1, PTEN, PTGS2, PTPRM, RAC2, RAP1GAP, RUNX2, RXRG, SEMA4D, SFRP1, SLC2A1, SMO, SOX2, SP110, SPHK1, SPP1, SRC, STAT2, SYK, TERF1, TFAP2A, TFF3, TGFA, TGFBR2, THBS1, TIMP3, TLR4, TMEM45A, TNC, TNFRSF1A, TRIM24, TRIM32, TUBA1C, TUBB3, TUBB4A, TWIST1, UBE2C, ZEB2, ZNF185	156	no
Cancer	central nervous system tumor	4.59E-05	Decreased	-2.283	ABL1, ACTA2, ADAM19, AKT3, ALAD, ANXA1, ANXA2, ARHGEF2, B4GALT5, BAX, BCL2, BCL3, BDNF, BIRC5, BMI1, CAV1, CCDC41, CCNB2, CCND1, CDK6, CDKN1B, CDKN2C, CEBPD, CHKA, CHRNB1, CIC, CXCL16, DAPK1, DHRS4, DKC1, DVL2, E2F3, EGR1, ENC1, EPHA2, ETV4, FGF2, FOXM1, FOXO3, FOXO4, FURIN, GEMIN5, GLI2, GNB4, H3F3A/H3F3B, HBEGF, HIPK2, HMGA1, HMGA2, HMGB2, HMOX1, ID1, ID2, IDH1, ING4, KDR, KHDRBS3, KRAS, LAMA4, LAMB1, LAMB3, LPAR1, MAP4K4, MARCKS, MCL1, MIA, MMP9, MYC, NFIB, NPTX1, NR3C1, NRIP1, NUSAP1, OLIG1, PAM16, PINK1, PROM1, PTEN, PTPRM, RAC2, RHPN2, SEMA4D, SFRP1, SMO, SNAI2, SOX2, SPHK1, SRC, STAT2, TERF1, TFAP2A, TGFA, TNC, TNFRSF1A, TUBB4A, UBE2C, ZEB2	97	no
Neurological Disease	central nervous system tumor	4.59E-05	Decreased	-2.283	ABL1, ACTA2, ADAM19, AKT3, ALAD, ANXA1, ANXA2, ARHGEF2, B4GALT5, BAX, BCL2, BCL3, BDNF, BIRC5, BMI1, CAV1, CCDC41, CCNB2, CCND1, CDK6, CDKN1B, CDKN2C, CEBPD, CHKA, CHRNB1, CIC, CXCL16, DAPK1, DHRS4, DKC1, DVL2, E2F3, EGR1, ENC1, EPHA2, ETV4, FGF2, FOXM1, FOXO3, FOXO4, FURIN, GEMIN5, GLI2, GNB4, H3F3A/H3F3B, HBEGF, HIPK2, HMGA1, HMGA2, HMGB2, HMOX1, ID1, ID2, IDH1, ING4, KDR, KHDRBS3, KRAS, LAMA4, LAMB1, LAMB3, LPAR1, MAP4K4, MARCKS, MCL1, MIA, MMP9, MYC, NFIB, NPTX1, NR3C1, NRIP1, NUSAP1, OLIG1, PAM16, PINK1, PROM1, PTEN, PTPRM, RAC2, RHPN2, SEMA4D, SFRP1, SMO, SNAI2, SOX2, SPHK1, SRC, STAT2, TERF1, TFAP2A, TGFA, TNC, TNFRSF1A, TUBB4A, UBE2C, ZEB2	97	no
Cellular Movement	cell movement	7.71E-12	Decreased	-2.277	ABCA1, ABCC1, ABL1, ABL2, ABR, ACSL4, ACVR1, ADAM19, ADAMTS1, ADD1, ADM, ADORA2B, ADRBK1, AFAP1, AHR, AKAP11, AKT3, AMD1, ANGPT1, ANGPT2, ANGPTL2, ANGPTL4, ANKS1A, ANXA1, ANXA2, AP2M1, APOE, ARAP3, ARHGAP24, ARHGEF1, ARHGEF6, ARID5B, ARNT, ARPC1B, ATM, ATP2B4, BAG3, BAX, BCAN, BCAR3, BCL2, BDNF, BHLHE40, BHLHE41, BID, BMPR1A, BRCA1, BTK, C1GALT1, C2, C3AR1, CADPS2, CALR, CASP1, CAV1, CBL, CCL2, CCL20, CCL28, CCNA2, CCNB2, CCND1, CCR1, CD2, CD200, CD276, CD36, CD58, CD59, CD74, CD97, CD99L2, CDC25B, CDK5, CDK5R1, CDKN1A, CDKN1B, CDKN2C, CEACAM1, CEBPD, CFB, CFH, CHN2, CHST2, CKLF, CLCA2, CLIC4, CMTM8, COL4A3BP, CPB2, CR2, CRKL, CSPG4, CTGF, CTLA4, CTSE, CTSL1, CXCL13, CXCL16, CXCR4, CYTH2, DAB2, DBF4, DCBLD2, DCC, DCLK1, DCX, DEFB4A/DEFB4B, DGCR6L, DIAPH2, DICER1, DKK1, DKK3, DLX1, DNAJB4, DUSP1, DUSP6, E2F2, E2F3, E2F5, EDNRB, EFNA1, EFNB2, EGLN1, EGR1, EGR2, ELK3, ENPP2, EPAS1, EPHA2, EPHA4, EPOR, ERRFI1, ETS1, ETV4, ETV5, EYA3, F2R, F2RL1, FADD, FAP, FAS, FBXL2, FBXO45, FCER1G, FCGR2A, FGF13, FGF2, FHOD1, FKBP4, FNBP1L, FOS, FOSL1, FOXD3, FOXM1, FOXO1, FOXO3, FOXO4, FST, FTH1, FURIN, FYB, FYN, FZD4, GAB1, GAL3ST1, GDF15, GEMIN5, GJB1, GLI2, GLRX, GNA12, GNA13, GNG12, GOLGA2, GPR183, GTPBP4, HAS2, HAS3, HAX1, HBEGF, HDAC6, HEY1, HEY2, HHEX, HIF1A, HIPK2, HMGB2, HMMR, HMOX1, HOXB2, HSP90AB1, HSPB1, HSPD1, ICAM1, ID1, ID2, ID3, IFNAR1, IGF2R, IGFBP3, IGFBP5, IL11RA, IL12A, IL16, IL1B, IL27RA, IL8, ING4, IRF5, IRS2, ITGA4, ITGA6, ITGB3, ITGB5, ITK, JAM3, JUN, KANK1, KDR, KIDINS220, KIT, KLF2, KLF4, KLHL20, KRAS, LAMA2, LAMB1, LAMB3, LAMC1, LEF1, LGMN, LHX6, LIF, LIMA1, LIMK1, LIMS1, LMCD1, LMO4, LOX, LPAR1, LRIG1, LRP5, LRP8, LTBR, LYN, MAP2K1, MAP2K3, MAP2K5, MAP2K6, MAP3K1, MAP3K12, MAP3K5, MAP3K7, MAP4K4, MAPKAPK5, MARCKS, MARCKSL1, MATN2, MBP, MC1R, MCAM, MCF2L, MCM2, MDK, MERTK, MESP1, MGLL, MIA, MITF, MLLT11, MMP9, MPP1, MRAS, MSN, MST1, MTSS1, MYB, MYC, MYLK, MYO10, MYO9A, NAMPT, NCKIPSD, NCL, NCOA4, NDRG1, NEDD9, NEO1, NFIA, NFKBIA, NLRP3, NOV, NOX4, NPC1, NPTX1, NQO1, NR2F1, NR4A2, NRCAM, NREP, NRP1, NRTN, NT5E, ONECUT2, OVOL2, P2RX7, PA2G4, PACSIN2, PAK4, PAPPA, PAX3, PAXIP1, PDE4B, PEX11B, PEX2, PEX5, PGF, PHB, PIK3C2B, PIK3CD, PIK3IP1, PLAT, PLAUR, PLCL1, PLGRKT, PLP1, PLXNA1, PLXNB1, PMP22, POU3F2, PPAP2A, PPAP2B, PPFIA1, PPM1F, PPP1R15A, PPP1R9A, PPP3CC, PRDX1, PREX1, PRKAR1A, PRKCE, PRKD1, PRMT2, PRNP, PTEN, PTGDS, PTGES, PTGS2, PTK2, PTP4A1, PTP4A2, PTPN12, PTPN14, PTPRA, PTPRM, PTPRZ1, PVR, RAB21, RAB27A, RAC2, RALA, RALB, RAP1GAP, RAP2A, RAPGEF1, RARA, RARRES1, RCAN1, REPS2, RGS1, RIN1, RNF144A, ROCK1, ROCK2, ROPN1B, RPRD1A, RRAS, RTN4, RUNX1, RUNX2, RUNX3, RUVBL1, RXRA, SARS, SCG2, SCHIP1, SCNN1A, SCPEP1, SDC3, SDC4, SEMA3A, SEMA3C, SEMA4D, SEMA6A, SFRP1, SH2B3, SH3BP1, SH3PXD2A, SH3PXD2B, SHC4, SIX4, SLC16A4, SLC2A1, SLC2A8, SLC3A2, SMO, SMPD1, SMPD2, SNAI2, SNX17, SNX27, SOCS2, SOD2, SORBS3, SORD, SORT1, SOX8, SP100, SPHK1, SPP1, SPRY2, SPRY4, SRC, SRGAP1, SSH1, ST3GAL4, ST3GAL5, ST3GAL6, ST6GAL1, STARD13, STC1, STIM1, STK24, STK4, STMN1, SYK, SYNM, TBXAS1, TFAP2A, TFAP2C, TFF3, TGFA, TGFBR2, TGFBR3, THBS1, THBS2, TIAM2, TIMP2, TIMP3, TIRAP, TLR4, TM4SF1, TNC, TNFRSF12A, TNFRSF1A, TNFRSF1B, TNFSF10, TNFSF13B, TNS3, TP53INP1, TPR, TPST2, TRAF3IP2, TRIB1, TRIM32, TRIP6, TRPV2, TUBA1C, TUBB2B, TWIST1, TXK, VASP, VCAN, VDR, VEGFA, VEGFB, VPS18, VPS28, WASL, WISP2, WWTR1, YARS, ZBTB24, ZEB2, ZFAND5, ZP3, ZYX	488	yes
Cellular Development	T cell development	1.12E-03	Decreased	-2.273	ABL1, AFF1, AHR, ANXA1, ATM, ATP7A, BATF, BAX, BCL2, BCL2L11, BCL3, BHLHE40, BHLHE41, BIRC5, BRCA1, C3AR1, CASP1, CBL, CCND1, CD2, CD74, CDKN1A, CDKN1B, CEBPB, CHD7, CTLA4, CXCR4, DEF6, DICER1, DKK1, E2F2, EFNA1, EFNB2, EGR1, EGR2, EGR3, ELK4, EOMES, EPHA2, EPHA4, ETS1, ETS2, FADD, FAS, FCER1G, FCGR2A, FOXO1, FOXO3, FYN, FZD7, GRAP2, HIF1A, HIVEP2, HSPD1, ICAM1, ID1, ID2, ID3, IFNAR1, IFNAR2, IFNGR2, IGF2R, IL12A, IL1B, IL27RA, IL7R, IRF4, IRF5, ITGA4, ITK, ITPKB, KDR, KIT, KLF4, LEF1, LIF, MAF, MALT1, MAP2K1, MAP3K7, MBP, MCL1, MLLT11, MNT, MYB, NFKBIA, PCSK1, PHLPP1, PIK3CD, PKNOX1, PLP1, PNP, PRKD1, PRNP, PTGS2, RUNX1, RUNX2, RUNX3, SATB1, SLC3A2, SNAI2, SOX4, SPP1, STAT2, SYK, TCF4, TGFBR2, TNFRSF1A, TNFRSF1B, TNFSF10, TNFSF13B, TXK, VEGFA, WASL, ZBTB17	115	no
Cellular Function and Maintenance	T cell development	1.12E-03	Decreased	-2.273	ABL1, AFF1, AHR, ANXA1, ATM, ATP7A, BATF, BAX, BCL2, BCL2L11, BCL3, BHLHE40, BHLHE41, BIRC5, BRCA1, C3AR1, CASP1, CBL, CCND1, CD2, CD74, CDKN1A, CDKN1B, CEBPB, CHD7, CTLA4, CXCR4, DEF6, DICER1, DKK1, E2F2, EFNA1, EFNB2, EGR1, EGR2, EGR3, ELK4, EOMES, EPHA2, EPHA4, ETS1, ETS2, FADD, FAS, FCER1G, FCGR2A, FOXO1, FOXO3, FYN, FZD7, GRAP2, HIF1A, HIVEP2, HSPD1, ICAM1, ID1, ID2, ID3, IFNAR1, IFNAR2, IFNGR2, IGF2R, IL12A, IL1B, IL27RA, IL7R, IRF4, IRF5, ITGA4, ITK, ITPKB, KDR, KIT, KLF4, LEF1, LIF, MAF, MALT1, MAP2K1, MAP3K7, MBP, MCL1, MLLT11, MNT, MYB, NFKBIA, PCSK1, PHLPP1, PIK3CD, PKNOX1, PLP1, PNP, PRKD1, PRNP, PTGS2, RUNX1, RUNX2, RUNX3, SATB1, SLC3A2, SNAI2, SOX4, SPP1, STAT2, SYK, TCF4, TGFBR2, TNFRSF1A, TNFRSF1B, TNFSF10, TNFSF13B, TXK, VEGFA, WASL, ZBTB17	115	no
Hematological System Development and Function	T cell development	1.12E-03	Decreased	-2.273	ABL1, AFF1, AHR, ANXA1, ATM, ATP7A, BATF, BAX, BCL2, BCL2L11, BCL3, BHLHE40, BHLHE41, BIRC5, BRCA1, C3AR1, CASP1, CBL, CCND1, CD2, CD74, CDKN1A, CDKN1B, CEBPB, CHD7, CTLA4, CXCR4, DEF6, DICER1, DKK1, E2F2, EFNA1, EFNB2, EGR1, EGR2, EGR3, ELK4, EOMES, EPHA2, EPHA4, ETS1, ETS2, FADD, FAS, FCER1G, FCGR2A, FOXO1, FOXO3, FYN, FZD7, GRAP2, HIF1A, HIVEP2, HSPD1, ICAM1, ID1, ID2, ID3, IFNAR1, IFNAR2, IFNGR2, IGF2R, IL12A, IL1B, IL27RA, IL7R, IRF4, IRF5, ITGA4, ITK, ITPKB, KDR, KIT, KLF4, LEF1, LIF, MAF, MALT1, MAP2K1, MAP3K7, MBP, MCL1, MLLT11, MNT, MYB, NFKBIA, PCSK1, PHLPP1, PIK3CD, PKNOX1, PLP1, PNP, PRKD1, PRNP, PTGS2, RUNX1, RUNX2, RUNX3, SATB1, SLC3A2, SNAI2, SOX4, SPP1, STAT2, SYK, TCF4, TGFBR2, TNFRSF1A, TNFRSF1B, TNFSF10, TNFSF13B, TXK, VEGFA, WASL, ZBTB17	115	no
Hematopoiesis	T cell development	1.12E-03	Decreased	-2.273	ABL1, AFF1, AHR, ANXA1, ATM, ATP7A, BATF, BAX, BCL2, BCL2L11, BCL3, BHLHE40, BHLHE41, BIRC5, BRCA1, C3AR1, CASP1, CBL, CCND1, CD2, CD74, CDKN1A, CDKN1B, CEBPB, CHD7, CTLA4, CXCR4, DEF6, DICER1, DKK1, E2F2, EFNA1, EFNB2, EGR1, EGR2, EGR3, ELK4, EOMES, EPHA2, EPHA4, ETS1, ETS2, FADD, FAS, FCER1G, FCGR2A, FOXO1, FOXO3, FYN, FZD7, GRAP2, HIF1A, HIVEP2, HSPD1, ICAM1, ID1, ID2, ID3, IFNAR1, IFNAR2, IFNGR2, IGF2R, IL12A, IL1B, IL27RA, IL7R, IRF4, IRF5, ITGA4, ITK, ITPKB, KDR, KIT, KLF4, LEF1, LIF, MAF, MALT1, MAP2K1, MAP3K7, MBP, MCL1, MLLT11, MNT, MYB, NFKBIA, PCSK1, PHLPP1, PIK3CD, PKNOX1, PLP1, PNP, PRKD1, PRNP, PTGS2, RUNX1, RUNX2, RUNX3, SATB1, SLC3A2, SNAI2, SOX4, SPP1, STAT2, SYK, TCF4, TGFBR2, TNFRSF1A, TNFRSF1B, TNFSF10, TNFSF13B, TXK, VEGFA, WASL, ZBTB17	115	no
Lymphoid Tissue Structure and Development	T cell development	1.12E-03	Decreased	-2.273	ABL1, AFF1, AHR, ANXA1, ATM, ATP7A, BATF, BAX, BCL2, BCL2L11, BCL3, BHLHE40, BHLHE41, BIRC5, BRCA1, C3AR1, CASP1, CBL, CCND1, CD2, CD74, CDKN1A, CDKN1B, CEBPB, CHD7, CTLA4, CXCR4, DEF6, DICER1, DKK1, E2F2, EFNA1, EFNB2, EGR1, EGR2, EGR3, ELK4, EOMES, EPHA2, EPHA4, ETS1, ETS2, FADD, FAS, FCER1G, FCGR2A, FOXO1, FOXO3, FYN, FZD7, GRAP2, HIF1A, HIVEP2, HSPD1, ICAM1, ID1, ID2, ID3, IFNAR1, IFNAR2, IFNGR2, IGF2R, IL12A, IL1B, IL27RA, IL7R, IRF4, IRF5, ITGA4, ITK, ITPKB, KDR, KIT, KLF4, LEF1, LIF, MAF, MALT1, MAP2K1, MAP3K7, MBP, MCL1, MLLT11, MNT, MYB, NFKBIA, PCSK1, PHLPP1, PIK3CD, PKNOX1, PLP1, PNP, PRKD1, PRNP, PTGS2, RUNX1, RUNX2, RUNX3, SATB1, SLC3A2, SNAI2, SOX4, SPP1, STAT2, SYK, TCF4, TGFBR2, TNFRSF1A, TNFRSF1B, TNFSF10, TNFSF13B, TXK, VEGFA, WASL, ZBTB17	115	no
Cell-mediated Immune Response	T cell development	1.12E-03	Decreased	-2.273	ABL1, AFF1, AHR, ANXA1, ATM, ATP7A, BATF, BAX, BCL2, BCL2L11, BCL3, BHLHE40, BHLHE41, BIRC5, BRCA1, C3AR1, CASP1, CBL, CCND1, CD2, CD74, CDKN1A, CDKN1B, CEBPB, CHD7, CTLA4, CXCR4, DEF6, DICER1, DKK1, E2F2, EFNA1, EFNB2, EGR1, EGR2, EGR3, ELK4, EOMES, EPHA2, EPHA4, ETS1, ETS2, FADD, FAS, FCER1G, FCGR2A, FOXO1, FOXO3, FYN, FZD7, GRAP2, HIF1A, HIVEP2, HSPD1, ICAM1, ID1, ID2, ID3, IFNAR1, IFNAR2, IFNGR2, IGF2R, IL12A, IL1B, IL27RA, IL7R, IRF4, IRF5, ITGA4, ITK, ITPKB, KDR, KIT, KLF4, LEF1, LIF, MAF, MALT1, MAP2K1, MAP3K7, MBP, MCL1, MLLT11, MNT, MYB, NFKBIA, PCSK1, PHLPP1, PIK3CD, PKNOX1, PLP1, PNP, PRKD1, PRNP, PTGS2, RUNX1, RUNX2, RUNX3, SATB1, SLC3A2, SNAI2, SOX4, SPP1, STAT2, SYK, TCF4, TGFBR2, TNFRSF1A, TNFRSF1B, TNFSF10, TNFSF13B, TXK, VEGFA, WASL, ZBTB17	115	no
Cellular Development	development of leukocytes	2.73E-04	Decreased	-2.266	ABL1, ADM, AFF1, AHR, ANXA1, ATM, ATP7A, BATF, BAX, BCL2, BCL2L11, BCL3, BHLHE40, BHLHE41, BIRC5, BRCA1, BTK, C3AR1, CASP1, CBL, CCND1, CD2, CD74, CDKN1A, CDKN1B, CEBPB, CHD7, CR2, CTLA4, CXCR4, DEF6, DICER1, DKK1, E2F2, EFNA1, EFNB2, EGR1, EGR2, EGR3, ELK4, EOMES, EPHA2, EPHA4, EPOR, ETS1, ETS2, FADD, FAS, FCER1G, FCGR2A, FGF2, FOS, FOXO1, FOXO3, FYN, FZD7, GRAP2, HIF1A, HIVEP2, HSPD1, ICAM1, ID1, ID2, ID3, IFNAR1, IFNAR2, IFNGR2, IGF2R, IL11RA, IL12A, IL1B, IL27RA, IL7R, IRF4, IRF5, ITGA4, ITGB3, ITK, ITPKB, KDR, KIT, KLF2, KLF4, KRAS, LEF1, LIF, LTBR, LYN, MAF, MALT1, MAP2K1, MAP3K7, MBP, MCL1, MDK, MERTK, MLLT11, MNT, MYB, NFKBIA, NQO1, PCSK1, PGF, PHLPP1, PIK3CD, PKNOX1, PLEKHA2, PLP1, PNP, PRKD1, PRNP, PTGS2, RALA, RALB, RARA, RUNX1, RUNX2, RUNX3, SATB1, SLC3A2, SMO, SNAI2, SOX4, SPP1, STAT2, SYK, TCF4, TGFBR2, TNFRSF1A, TNFRSF1B, TNFSF10, TNFSF13B, TP53BP1, TXK, VEGFA, WASL, XBP1, ZBTB17	138	no
Hematological System Development and Function	development of leukocytes	2.73E-04	Decreased	-2.266	ABL1, ADM, AFF1, AHR, ANXA1, ATM, ATP7A, BATF, BAX, BCL2, BCL2L11, BCL3, BHLHE40, BHLHE41, BIRC5, BRCA1, BTK, C3AR1, CASP1, CBL, CCND1, CD2, CD74, CDKN1A, CDKN1B, CEBPB, CHD7, CR2, CTLA4, CXCR4, DEF6, DICER1, DKK1, E2F2, EFNA1, EFNB2, EGR1, EGR2, EGR3, ELK4, EOMES, EPHA2, EPHA4, EPOR, ETS1, ETS2, FADD, FAS, FCER1G, FCGR2A, FGF2, FOS, FOXO1, FOXO3, FYN, FZD7, GRAP2, HIF1A, HIVEP2, HSPD1, ICAM1, ID1, ID2, ID3, IFNAR1, IFNAR2, IFNGR2, IGF2R, IL11RA, IL12A, IL1B, IL27RA, IL7R, IRF4, IRF5, ITGA4, ITGB3, ITK, ITPKB, KDR, KIT, KLF2, KLF4, KRAS, LEF1, LIF, LTBR, LYN, MAF, MALT1, MAP2K1, MAP3K7, MBP, MCL1, MDK, MERTK, MLLT11, MNT, MYB, NFKBIA, NQO1, PCSK1, PGF, PHLPP1, PIK3CD, PKNOX1, PLEKHA2, PLP1, PNP, PRKD1, PRNP, PTGS2, RALA, RALB, RARA, RUNX1, RUNX2, RUNX3, SATB1, SLC3A2, SMO, SNAI2, SOX4, SPP1, STAT2, SYK, TCF4, TGFBR2, TNFRSF1A, TNFRSF1B, TNFSF10, TNFSF13B, TP53BP1, TXK, VEGFA, WASL, XBP1, ZBTB17	138	no
Hematopoiesis	development of leukocytes	2.73E-04	Decreased	-2.266	ABL1, ADM, AFF1, AHR, ANXA1, ATM, ATP7A, BATF, BAX, BCL2, BCL2L11, BCL3, BHLHE40, BHLHE41, BIRC5, BRCA1, BTK, C3AR1, CASP1, CBL, CCND1, CD2, CD74, CDKN1A, CDKN1B, CEBPB, CHD7, CR2, CTLA4, CXCR4, DEF6, DICER1, DKK1, E2F2, EFNA1, EFNB2, EGR1, EGR2, EGR3, ELK4, EOMES, EPHA2, EPHA4, EPOR, ETS1, ETS2, FADD, FAS, FCER1G, FCGR2A, FGF2, FOS, FOXO1, FOXO3, FYN, FZD7, GRAP2, HIF1A, HIVEP2, HSPD1, ICAM1, ID1, ID2, ID3, IFNAR1, IFNAR2, IFNGR2, IGF2R, IL11RA, IL12A, IL1B, IL27RA, IL7R, IRF4, IRF5, ITGA4, ITGB3, ITK, ITPKB, KDR, KIT, KLF2, KLF4, KRAS, LEF1, LIF, LTBR, LYN, MAF, MALT1, MAP2K1, MAP3K7, MBP, MCL1, MDK, MERTK, MLLT11, MNT, MYB, NFKBIA, NQO1, PCSK1, PGF, PHLPP1, PIK3CD, PKNOX1, PLEKHA2, PLP1, PNP, PRKD1, PRNP, PTGS2, RALA, RALB, RARA, RUNX1, RUNX2, RUNX3, SATB1, SLC3A2, SMO, SNAI2, SOX4, SPP1, STAT2, SYK, TCF4, TGFBR2, TNFRSF1A, TNFRSF1B, TNFSF10, TNFSF13B, TP53BP1, TXK, VEGFA, WASL, XBP1, ZBTB17	138	no

Lymphoid Tissue Structure and Development	development of leukocytes	2.73E-04	Decreased	-2.266	ABL1, ADM, AFF1, AHR, ANXA1, ATM, ATP7A, BATF, BAX, BCL2, BCL2L11, BCL3, BHLHE40, BHLHE41, BIRC5, BRCA1, BTK, C3AR1, CASP1, CBL, CCND1, CD2, CD74, CDKN1A, CDKN1B, CEBPB, CHD7, CR2, CTLA4, CXCR4, DEF6, DICER1, DKK1, E2F2, EFNA1, EFNB2, EGR1, EGR2, EGR3, ELK4, EOMES, EPHA2, EPHA4, EPOR, ETS1, ETS2, FADD, FAS, FCER1G, FCGR2A, FGF2, FOS, FOXO1, FOXO3, FYN, FZD7, GRAP2, HIF1A, HIVEP2, HSPD1, ICAM1, ID1, ID2, ID3, IFNAR1, IFNAR2, IFNGR2, IGF2R, IL11RA, IL12A, IL1B, IL27RA, IL7R, IRF4, IRF5, ITGA4, ITGB3, ITK, ITPKB, KDR, KIT, KLF2, KLF4, KRAS, LEF1, LIF, LTBR, LYN, MAF, MALT1, MAP2K1, MAP3K7, MBP, MCL1, MDK, MERTK, MLLT11, MNT, MYB, NFKBIA, NQO1, PCSK1, PGF, PHLPP1, PIK3CD, PKNOX1, PLEKHA2, PLP1, PNP, PRKD1, PRNP, PTGS2, RALA, RALB, RARA, RUNX1, RUNX2, RUNX3, SATB1, SLC3A2, SMO, SNAI2, SOX4, SPP1, STAT2, SYK, TCF4, TGFBR2, TNFRSF1A, TNFRSF1B, TNFSF10, TNFSF13B, TP53BP1, TXK, VEGFA, WASL, XBP1, ZBTB17	138	no
Cancer	brain cancer	1.84E-05	Decreased	-2.254	ABL1, ACTA2, AKT3, ALAD, ANXA1, ANXA2, ARHGEF2, BAX, BCL2, BCL3, BDNF, BMI1, CAV1, CCDC41, CCNB2, CCND1, CDK6, CDKN1B, CDKN2C, CEBPD, CHKA, CHRNB1, CXCL16, DAPK1, DHRS4, DKC1, DVL2, E2F3, EGR1, ENC1, EPHA2, FOXM1, FOXO3, FOXO4, FURIN, GEMIN5, GLI2, GNB4, HIPK2, HMGA1, HMGA2, HMGB2, HMOX1, ID1, IDH1, KDR, KHDRBS3, KRAS, LAMB3, LPAR1, MAP4K4, MARCKS, MCL1, MIA, MMP9, MYC, NFIB, NPTX1, NR3C1, NRIP1, NUSAP1, OLIG1, PAM16, PINK1, PROM1, PTEN, PTPRM, RAC2, SEMA4D, SFRP1, SMO, SOX2, SPHK1, SRC, STAT2, TERF1, TFAP2A, TGFA, TNC, TNFRSF1A, TUBB4A, UBE2C, ZEB2	83	no
Neurological Disease	brain cancer	1.84E-05	Decreased	-2.254	ABL1, ACTA2, AKT3, ALAD, ANXA1, ANXA2, ARHGEF2, BAX, BCL2, BCL3, BDNF, BMI1, CAV1, CCDC41, CCNB2, CCND1, CDK6, CDKN1B, CDKN2C, CEBPD, CHKA, CHRNB1, CXCL16, DAPK1, DHRS4, DKC1, DVL2, E2F3, EGR1, ENC1, EPHA2, FOXM1, FOXO3, FOXO4, FURIN, GEMIN5, GLI2, GNB4, HIPK2, HMGA1, HMGA2, HMGB2, HMOX1, ID1, IDH1, KDR, KHDRBS3, KRAS, LAMB3, LPAR1, MAP4K4, MARCKS, MCL1, MIA, MMP9, MYC, NFIB, NPTX1, NR3C1, NRIP1, NUSAP1, OLIG1, PAM16, PINK1, PROM1, PTEN, PTPRM, RAC2, SEMA4D, SFRP1, SMO, SOX2, SPHK1, SRC, STAT2, TERF1, TFAP2A, TGFA, TNC, TNFRSF1A, TUBB4A, UBE2C, ZEB2	83	no
Cancer	brain tumor	3.00E-05	Decreased	-2.254	ABL1, ACTA2, ADAM19, AKT3, ALAD, ANXA1, ANXA2, ARHGEF2, BAX, BCL2, BCL3, BDNF, BMI1, CAV1, CCDC41, CCNB2, CCND1, CDK6, CDKN1B, CDKN2C, CEBPD, CHKA, CHRNB1, CXCL16, DAPK1, DHRS4, DKC1, DVL2, E2F3, EGR1, ENC1, EPHA2, FGF2, FOXM1, FOXO3, FOXO4, FURIN, GEMIN5, GLI2, GNB4, HIPK2, HMGA1, HMGA2, HMGB2, HMOX1, ID1, IDH1, KDR, KHDRBS3, KRAS, LAMB3, LPAR1, MAP4K4, MARCKS, MCL1, MIA, MMP9, MYC, NFIB, NPTX1, NR3C1, NRIP1, NUSAP1, OLIG1, PAM16, PINK1, PROM1, PTEN, PTPRM, RAC2, SEMA4D, SFRP1, SMO, SOX2, SPHK1, SRC, STAT2, TERF1, TFAP2A, TGFA, TNC, TNFRSF1A, TUBB4A, UBE2C, ZEB2	85	no
Neurological Disease	brain tumor	3.00E-05	Decreased	-2.254	ABL1, ACTA2, ADAM19, AKT3, ALAD, ANXA1, ANXA2, ARHGEF2, BAX, BCL2, BCL3, BDNF, BMI1, CAV1, CCDC41, CCNB2, CCND1, CDK6, CDKN1B, CDKN2C, CEBPD, CHKA, CHRNB1, CXCL16, DAPK1, DHRS4, DKC1, DVL2, E2F3, EGR1, ENC1, EPHA2, FGF2, FOXM1, FOXO3, FOXO4, FURIN, GEMIN5, GLI2, GNB4, HIPK2, HMGA1, HMGA2, HMGB2, HMOX1, ID1, IDH1, KDR, KHDRBS3, KRAS, LAMB3, LPAR1, MAP4K4, MARCKS, MCL1, MIA, MMP9, MYC, NFIB, NPTX1, NR3C1, NRIP1, NUSAP1, OLIG1, PAM16, PINK1, PROM1, PTEN, PTPRM, RAC2, SEMA4D, SFRP1, SMO, SOX2, SPHK1, SRC, STAT2, TERF1, TFAP2A, TGFA, TNC, TNFRSF1A, TUBB4A, UBE2C, ZEB2	85	no
Cancer	neoplasia of tumor cells	2.39E-04	Decreased	-2.244	ABL1, CAV1, CBR1, CDC25A, CREB3L4, CTSL1, CXCR4, DCC, DGCR8, DICER1, KRAS, LZTS1, MCAM, MMP9, PTEN, PTK2, PTPRA, RRM1, RUNX2, SPHK1, TGFA, TGFBR2, TP53BP1, VEGFA	24	no
Cell Cycle	interphase of breast cell	1.10E-03	Decreased	-2.236	CDC25C, CDKN1A, CDKN1B, FADD, ID1, KDM5B, LEF1, MYC	8	yes
Cancer	genital tumor	2.55E-06	Decreased	-2.222	ABCC1, ABCC4, ABL1, ACAD8, ACO1, ACRC, ADAMTS9, AFAP1, ALPK1, AMACR, AMD1, ANAPC1/ANAPC1P1, ANGPT1, ANTXR2, ANXA2, APOE, ARAP3, ARG2, ARMCX3, ATM, ATP10B, ATXN1, AURKB, BAX, BCL2, BECN1, BHLHE40, BIRC5, BMI1, BMPR1A, BRCA1, BSDC1, BTG3, C11orf9, C1R, CALCOCO2, CAV1, CCDC41, CCL2, CCNB2, CCND1, CCND2, CD74, CDC25A, CDC25B, CDC25C, CDC42EP1, CDK6, CDKN1A, CDKN1B, CDKN2C, CFB, CFH, CHD1, CHRM1, CHST2, CIRBP, CMTM8, COL15A1, CROCC, CTDSPL, CTGF, CTLA4, CXCL16, CXCR4, DAB2, DAB2IP, DGCR8, DHRS4, DICER1, DTNA, DUSP1, DUSP4, DYNLRB1, E2F2, E2F3, EDNRB, EFNB2, EIF2C2, ELK4, EPHA4, EPOR, ESRP1, ETV1, ETV4, ETV5, F2R, FAM136A, FAM13A, FAM222B, FAS, FASN, FBXL13, FEN1, FGF2, FGFR3, FHOD3, FOS, FOSL1, FOXD3, FOXO3, FOXO4, FRMD6, FYCO1, FYN, GDF15, GEMIN5, GLDC, GLI2, GNA12, GPR101, GPR183, GPRC5A, GSTM1, GTF2I, GUCY1A2, H2AFX, HILPDA, HIP1, HIPK2, HIST2H2AA3/HIST2H2AA4, HMGA1, HMGA2, HMOX1, HOXA5, HOXB13, HOXB2, HOXC4, HOXC6, HOXD11, HSP90AB1, ID1, IDH1, IER3, IFNGR2, IGF2BP3, IGFBP3, IL1RAPL1, IL7R, IL8, ING4, IRS2, ITK, ITPR3, JUN, KCNS3, KDR, KHDRBS3, KIAA1147, KIAA1211, KIF11, KIT, KRAS, LARP1B, LCTL, LEF1, LOXL3, LPAR1, LRIG1, LRP5, LRP8, LTBR, MAMLD1, MAP2K1, MCAM, MCL1, MCM2, MDK, MMP9, MSMB, MST1, MTHFD2, MXI1, MYC, MYLK, NCL, NFKBIA, NKX2-5, NKX3-1, NPIPL2 (includes others), NPTX1, NR3C1, NT5E, OLIG1, PDCD2L, PDK4, PDLIM4, PGF, PGM5, PI15, PLAUR, PLGLB1/PLGLB2, PLXNB1, POLE, PPAP2A, PPAT, PPP3CC, PRAME, PRKCE, PROM1, PSAT1, PSMD4, PTEN, PTGDS, PTGES, PTGS2, PTK2, PTPRA, QKI, RACGAP1, RARB, RARRES1, RCAN2, RCBTB2, RNASEL, RNF144A, RNF144B, RNF145, RPL29, RPS6KC1, RRAS, RRM1, RUNX2, RUNX3, SAT1, SEC14L1, SECISBP2L, SEMA3C, SEMA4D, SFRP1, SH3PXD2A, SIM2, SLC16A3, SLC2A1, SLC7A1, SLC7A6, SMO, SNAI2, SOD2, SON, SORT1, SOX2, SPOP, SPP1, SPRED2, SPRY1, SPRY2, SRC, SRGAP1, SSTR2, ST7, STAT2, STC1, STIM1, STK4, SUN3, SYNPO2, TBC1D14, TFF3, TGFBR2, TIMP3, TK1, TNS3, TP53INP1, TPD52, TRADD, TRIM48, TRIOBP, TRO, TRRAP, TTK, TUBA1C, TUBB2B, TUBB3, TUBB4A, TUBB6, TUBB8, TXNIP, UBE2C, UGT2B17, VCAN, VEGFA, VEGFB, WDR19, WIPF1, XPC, YEATS2, ZBTB17, ZBTB20, ZBTB22, ZC3H15, ZDHHC11, ZFP36, ZFYVE26, ZNF827, ZWINT	296	no
Reproductive System Disease	genital tumor	2.55E-06	Decreased	-2.222	ABCC1, ABCC4, ABL1, ACAD8, ACO1, ACRC, ADAMTS9, AFAP1, ALPK1, AMACR, AMD1, ANAPC1/ANAPC1P1, ANGPT1, ANTXR2, ANXA2, APOE, ARAP3, ARG2, ARMCX3, ATM, ATP10B, ATXN1, AURKB, BAX, BCL2, BECN1, BHLHE40, BIRC5, BMI1, BMPR1A, BRCA1, BSDC1, BTG3, C11orf9, C1R, CALCOCO2, CAV1, CCDC41, CCL2, CCNB2, CCND1, CCND2, CD74, CDC25A, CDC25B, CDC25C, CDC42EP1, CDK6, CDKN1A, CDKN1B, CDKN2C, CFB, CFH, CHD1, CHRM1, CHST2, CIRBP, CMTM8, COL15A1, CROCC, CTDSPL, CTGF, CTLA4, CXCL16, CXCR4, DAB2, DAB2IP, DGCR8, DHRS4, DICER1, DTNA, DUSP1, DUSP4, DYNLRB1, E2F2, E2F3, EDNRB, EFNB2, EIF2C2, ELK4, EPHA4, EPOR, ESRP1, ETV1, ETV4, ETV5, F2R, FAM136A, FAM13A, FAM222B, FAS, FASN, FBXL13, FEN1, FGF2, FGFR3, FHOD3, FOS, FOSL1, FOXD3, FOXO3, FOXO4, FRMD6, FYCO1, FYN, GDF15, GEMIN5, GLDC, GLI2, GNA12, GPR101, GPR183, GPRC5A, GSTM1, GTF2I, GUCY1A2, H2AFX, HILPDA, HIP1, HIPK2, HIST2H2AA3/HIST2H2AA4, HMGA1, HMGA2, HMOX1, HOXA5, HOXB13, HOXB2, HOXC4, HOXC6, HOXD11, HSP90AB1, ID1, IDH1, IER3, IFNGR2, IGF2BP3, IGFBP3, IL1RAPL1, IL7R, IL8, ING4, IRS2, ITK, ITPR3, JUN, KCNS3, KDR, KHDRBS3, KIAA1147, KIAA1211, KIF11, KIT, KRAS, LARP1B, LCTL, LEF1, LOXL3, LPAR1, LRIG1, LRP5, LRP8, LTBR, MAMLD1, MAP2K1, MCAM, MCL1, MCM2, MDK, MMP9, MSMB, MST1, MTHFD2, MXI1, MYC, MYLK, NCL, NFKBIA, NKX2-5, NKX3-1, NPIPL2 (includes others), NPTX1, NR3C1, NT5E, OLIG1, PDCD2L, PDK4, PDLIM4, PGF, PGM5, PI15, PLAUR, PLGLB1/PLGLB2, PLXNB1, POLE, PPAP2A, PPAT, PPP3CC, PRAME, PRKCE, PROM1, PSAT1, PSMD4, PTEN, PTGDS, PTGES, PTGS2, PTK2, PTPRA, QKI, RACGAP1, RARB, RARRES1, RCAN2, RCBTB2, RNASEL, RNF144A, RNF144B, RNF145, RPL29, RPS6KC1, RRAS, RRM1, RUNX2, RUNX3, SAT1, SEC14L1, SECISBP2L, SEMA3C, SEMA4D, SFRP1, SH3PXD2A, SIM2, SLC16A3, SLC2A1, SLC7A1, SLC7A6, SMO, SNAI2, SOD2, SON, SORT1, SOX2, SPOP, SPP1, SPRED2, SPRY1, SPRY2, SRC, SRGAP1, SSTR2, ST7, STAT2, STC1, STIM1, STK4, SUN3, SYNPO2, TBC1D14, TFF3, TGFBR2, TIMP3, TK1, TNS3, TP53INP1, TPD52, TRADD, TRIM48, TRIOBP, TRO, TRRAP, TTK, TUBA1C, TUBB2B, TUBB3, TUBB4A, TUBB6, TUBB8, TXNIP, UBE2C, UGT2B17, VCAN, VEGFA, VEGFB, WDR19, WIPF1, XPC, YEATS2, ZBTB17, ZBTB20, ZBTB22, ZC3H15, ZDHHC11, ZFP36, ZFYVE26, ZNF827, ZWINT	296	no
Cellular Movement	cell movement of vascular smooth muscle cells	5.60E-04	Decreased	-2.216	ADM, ANGPT2, CCND1, CDKN1B, CLIC4, FGF2, FOXO4, FZD4, HMOX1, IGFBP5, IL1B, IL8, ITGB5, KDR, NEO1, NOV, NQO1, PAPPA, PLAUR, PTGS2, RCAN1, ROCK1, RTN4, SDC4, SPP1, THBS1, TLR4, TNFSF10, TRIB1, WISP2	30	yes
Skeletal and Muscular System Development and Function	cell movement of vascular smooth muscle cells	5.60E-04	Decreased	-2.216	ADM, ANGPT2, CCND1, CDKN1B, CLIC4, FGF2, FOXO4, FZD4, HMOX1, IGFBP5, IL1B, IL8, ITGB5, KDR, NEO1, NOV, NQO1, PAPPA, PLAUR, PTGS2, RCAN1, ROCK1, RTN4, SDC4, SPP1, THBS1, TLR4, TNFSF10, TRIB1, WISP2	30	yes
Cellular Movement	cell movement of breast cancer cell lines	2.37E-08	Decreased	-2.214	ACSL4, ANGPT2, ANKS1A, ANXA1, ARPC1B, BCAR3, BHLHE41, CAV1, CCL2, CDKN1B, CHN2, COL4A3BP, CTSL1, CXCR4, DBF4, EPHA2, EPOR, ETS1, FGF2, FHOD1, FOS, FOSL1, FOXO3, FURIN, GAB1, GEMIN5, HIF1A, HMMR, ID1, IGFBP3, IL8, ITGA6, ITGB3, JUN, LIMK1, LPAR1, LRIG1, MAP2K1, MAP4K4, MCAM, MCF2L, MMP9, NDRG1, NFKBIA, NRP1, PIK3CD, PLAUR, PLXNA1, PPM1F, PRKD1, PTK2, RAB21, RAB27A, RAP1GAP, RNF144A, RUVBL1, SEMA3A, SEMA4D, SLC16A4, SNAI2, SPHK1, SPP1, SRC, SSH1, SYK, TFAP2A, TFAP2C, TGFA, TIMP2, TUBA1C, VASP, VEGFA, WWTR1	73	yes
Cellular Development	proliferation of muscle cells	3.80E-05	Decreased	-2.209	ABCC2, ABCC4, ADM, ANG, ANGPT2, APOE, ATP2B1, BDNF, BIRC5, CAV1, CBL, CCL2, CDKN1A, CDKN1B, CEBPD, CTNNBIP1, CXCL16, DGCR8, DUSP1, E2F3, FGF2, FOS, FOXK1, FOXM1, FOXO1, FZD4, HBEGF, HEY1, HEY2, HIF1A, HMGA2, HMGCR, HMOX1, HSPD1, ID2, ID3, IGFBP3, IGFBP5, IL12A, IL1B, IL8, IRAK1, ITGA6, ITGB3, JUN, KIT, KLF4, KRAS, LIF, LPAR1, MAP2K1, MMP9, MYB, MYC, NAMPT, NOV, NOX4, NQO1, NR4A2, PAPPA, PLAT, PLAUR, PRKAR1A, PRNP, PRRX1, PTEN, PTGDS, PTGS2, PTK2, RARA, ROCK1, RXRA, SDC4, SPP1, ST8SIA1, TCF4, TGFBR2, TGFBR3, THBS1, TLR4, TNC, TNFRSF12A, TNFRSF1A, TNFRSF1B, TNFSF10, TRIB1, VCAN, VEGFA, WISP2	89	no
Cellular Growth and Proliferation	proliferation of muscle cells	3.80E-05	Decreased	-2.209	ABCC2, ABCC4, ADM, ANG, ANGPT2, APOE, ATP2B1, BDNF, BIRC5, CAV1, CBL, CCL2, CDKN1A, CDKN1B, CEBPD, CTNNBIP1, CXCL16, DGCR8, DUSP1, E2F3, FGF2, FOS, FOXK1, FOXM1, FOXO1, FZD4, HBEGF, HEY1, HEY2, HIF1A, HMGA2, HMGCR, HMOX1, HSPD1, ID2, ID3, IGFBP3, IGFBP5, IL12A, IL1B, IL8, IRAK1, ITGA6, ITGB3, JUN, KIT, KLF4, KRAS, LIF, LPAR1, MAP2K1, MMP9, MYB, MYC, NAMPT, NOV, NOX4, NQO1, NR4A2, PAPPA, PLAT, PLAUR, PRKAR1A, PRNP, PRRX1, PTEN, PTGDS, PTGS2, PTK2, RARA, ROCK1, RXRA, SDC4, SPP1, ST8SIA1, TCF4, TGFBR2, TGFBR3, THBS1, TLR4, TNC, TNFRSF12A, TNFRSF1A, TNFRSF1B, TNFSF10, TRIB1, VCAN, VEGFA, WISP2	89	no
Skeletal and Muscular System Development and Function	proliferation of muscle cells	3.80E-05	Decreased	-2.209	ABCC2, ABCC4, ADM, ANG, ANGPT2, APOE, ATP2B1, BDNF, BIRC5, CAV1, CBL, CCL2, CDKN1A, CDKN1B, CEBPD, CTNNBIP1, CXCL16, DGCR8, DUSP1, E2F3, FGF2, FOS, FOXK1, FOXM1, FOXO1, FZD4, HBEGF, HEY1, HEY2, HIF1A, HMGA2, HMGCR, HMOX1, HSPD1, ID2, ID3, IGFBP3, IGFBP5, IL12A, IL1B, IL8, IRAK1, ITGA6, ITGB3, JUN, KIT, KLF4, KRAS, LIF, LPAR1, MAP2K1, MMP9, MYB, MYC, NAMPT, NOV, NOX4, NQO1, NR4A2, PAPPA, PLAT, PLAUR, PRKAR1A, PRNP, PRRX1, PTEN, PTGDS, PTGS2, PTK2, RARA, ROCK1, RXRA, SDC4, SPP1, ST8SIA1, TCF4, TGFBR2, TGFBR3, THBS1, TLR4, TNC, TNFRSF12A, TNFRSF1A, TNFRSF1B, TNFSF10, TRIB1, VCAN, VEGFA, WISP2	89	no
Cellular Assembly and Organization	quantity of membrane ruffles	2.13E-05	Decreased	-2.189	ADD1, ARHGEF2, CDC42EP1, IL8, PTEN, ROCK2, SORBS2	7	no
Cellular Growth and Proliferation	proliferation of connective tissue cells	6.89E-05	Decreased	-2.171	ABL1, AHR, AKAP12, AKT3, ATM, BAX, BCL2, BMI1, BMPR1A, CAV1, CBL, CCL2, CCNA2, CCND1, CCND2, CCNG2, CD74, CDK6, CDKN1A, CDKN1B, CEBPB, CIRBP, CTC1, CTGF, CTHRC1, DICER1, DKK1, DUSP1, EDNRB, EFNA1, EPHA2, ESPL1, F2R, F2RL1, FADD, FAS, FEN1, FGF13, FGF2, FGFR3, FLI1, FNDC3B, FOS, FOSL1, FOXO1, FOXO4, FST, FTH1, FYN, GHR, GLI2, HBEGF, HIF1A, HMMR, HMOX1, HOXA5, HOXB13, ID2, IFNAR1, IGFBP3, IGFBP5, IL1B, IL8, IP6K2, ITGB3, JUN, JUND, KLF4, KRAS, LIF, LIMA1, LOX, LPAR1, LRIG1, LRP5, LZTS1, MAP2K1, MAP2K3, MAP2K6, MAP3K1, MAP3K5, MAP3K7, MCF2L, MMP9, MYC, NFKBIA, NOX4, NR3C1, NUPR1, OVOL2, PA2G4, PEX2, PGF, PHC2, PHLPP1, PLAT, PLAUR, PMS2, PPAT, PPP1R13L, PSMD10, PTEN, PTGES, PTGS2, PTK2, PTPRZ1, RAPGEF1, RARA, RIT1, RPL29, RRM1, RUNX1, RUNX2, RXRA, SFRP1, SIRT2, SLC3A2, SMAD6, SMARCAL1, SMO, SNAI2, SOD2, SOX8, SPHK1, SPP1, SPRY2, SRC, STARD10, STC1, TAF4, TGFA, TGFBR2, TGIF1, TNFRSF12A, TNFRSF1A, TNFSF10, TPR, TWIST1, TXNIP, UBE2C, VCAN, VDR, WASL	143	yes
Cellular Development	branching of cells	2.60E-04	Decreased	-2.163	ABL1, ADM, ANG, ANGPT1, ANGPT2, ANGPTL2, ANXA2, BCL2, BDNF, CDK5R1, DCX, DICER1, EFNA1, ETS1, FADD, FGF2, GAB1, HOXD11, JUN, KDR, KRAS, LAMA2, LAMA4, MKS1, MMP9, MST1, NFKBIA, PIM3, RND3, RRAS, RTN4, RUNX2, SARS, SEMA4D, SPP1, TGFA, TIMP2, TMEM67, TNC, VEGFA	40	no
Cell Cycle	entry into S phase of fibroblasts	8.24E-04	Decreased	-2.138	ATM, CCND1, CDKN1A, CDKN1B, E2F2, E2F3, E2F5, ID3, MYC, TFDP1	10	yes
Connective Tissue Development and Function	entry into S phase of fibroblasts	8.24E-04	Decreased	-2.138	ATM, CCND1, CDKN1A, CDKN1B, E2F2, E2F3, E2F5, ID3, MYC, TFDP1	10	yes
Cancer	neoplasia of cancer cells	5.77E-04	Decreased	-2.137	ABL1, CBR1, CREB3L4, CTSL1, CXCR4, DCC, DGCR8, DICER1, KRAS, LZTS1, MCAM, PTEN, RRM1, TGFA, TGFBR2, VEGFA	16	no
Cellular Development	proliferation of tumor cells	3.45E-06	Decreased	-2.086	ABL1, ADRBK1, AFAP1L2, ANXA2, ATM, ATP2A2, BAX, BCAN, BCL2, BECN1, BIRC5, BMI1, CASP1, CAV1, CCND1, CDC25A, CDK5, CDK6, CDKN1A, CDKN1B, CDKN2C, CXCR4, DCC, DDIT3, DGCR8, DICER1, DKK1, DUSP1, DVL2, EFNA1, EGR1, EPHA2, F2R, F2RL1, FAS, FASN, FGF2, FOS, FOSL1, FOXM1, FOXO3, FST, GDF15, GREB1, HBEGF, HDAC6, HIF1A, HMGA2, HMGCR, HMMR, HMOX1, ID1, ID2, ID3, IGFBP3, IGFBP5, IL12A, IL1B, IL8, KDR, KIT, KRAS, LZTS1, LZTS2, MAP2K1, MAP2K3, MAP2K5, MAP2K6, MCAM, MMP9, MST1, MYB, MYC, NFKBIA, NKX3-1, NOV, NOX4, NR4A2, NRP1, PLAT, PLAUR, POU3F2, PPP1R13L, PRKCE, PTEN, PTGS2, PTPRA, PTPRE, RARB, RCAN1, RUVBL1, RXRA, SEC14L2, SEMA4D, SFRP1, SLC3A2, SMO, SNAI2, SPHK1, SPP1, SRC, SSTR2, TAX1BP3, TCF7L2, TFF3, TGFA, TGFBR2, THBS1, TIAM2, TIMP2, TIMP3, TLE1, TNFSF10, TNFSF13B, TP53BP1, TPD52, TWIST1, TXNIP, USP10, VCAN, VEGFA, XBP1, ZMAT3	123	yes
Cellular Growth and Proliferation	proliferation of tumor cells	3.45E-06	Decreased	-2.086	ABL1, ADRBK1, AFAP1L2, ANXA2, ATM, ATP2A2, BAX, BCAN, BCL2, BECN1, BIRC5, BMI1, CASP1, CAV1, CCND1, CDC25A, CDK5, CDK6, CDKN1A, CDKN1B, CDKN2C, CXCR4, DCC, DDIT3, DGCR8, DICER1, DKK1, DUSP1, DVL2, EFNA1, EGR1, EPHA2, F2R, F2RL1, FAS, FASN, FGF2, FOS, FOSL1, FOXM1, FOXO3, FST, GDF15, GREB1, HBEGF, HDAC6, HIF1A, HMGA2, HMGCR, HMMR, HMOX1, ID1, ID2, ID3, IGFBP3, IGFBP5, IL12A, IL1B, IL8, KDR, KIT, KRAS, LZTS1, LZTS2, MAP2K1, MAP2K3, MAP2K5, MAP2K6, MCAM, MMP9, MST1, MYB, MYC, NFKBIA, NKX3-1, NOV, NOX4, NR4A2, NRP1, PLAT, PLAUR, POU3F2, PPP1R13L, PRKCE, PTEN, PTGS2, PTPRA, PTPRE, RARB, RCAN1, RUVBL1, RXRA, SEC14L2, SEMA4D, SFRP1, SLC3A2, SMO, SNAI2, SPHK1, SPP1, SRC, SSTR2, TAX1BP3, TCF7L2, TFF3, TGFA, TGFBR2, THBS1, TIAM2, TIMP2, TIMP3, TLE1, TNFSF10, TNFSF13B, TP53BP1, TPD52, TWIST1, TXNIP, USP10, VCAN, VEGFA, XBP1, ZMAT3	123	yes

Tumor Morphology	proliferation of tumor cells	3.45E-06	Decreased	-2.086	ABL1, ADRBK1, AFAP1L2, ANXA2, ATM, ATP2A2, BAX, BCAN, BCL2, BECN1, BIRC5, BMI1, CASP1, CAV1, CCND1, CDC25A, CDK5, CDK6, CDKN1A, CDKN1B, CDKN2C, CXCR4, DCC, DDIT3, DGCR8, DICER1, DKK1, DUSP1, DVL2, EFNA1, EGR1, EPHA2, F2R, F2RL1, FAS, FASN, FGF2, FOS, FOSL1, FOXM1, FOXO3, FST, GDF15, GREB1, HBEGF, HDAC6, HIF1A, HMGA2, HMGCR, HMMR, HMOX1, ID1, ID2, ID3, IGFBP3, IGFBP5, IL12A, IL1B, IL8, KDR, KIT, KRAS, LZTS1, LZTS2, MAP2K1, MAP2K3, MAP2K5, MAP2K6, MCAM, MMP9, MST1, MYB, MYC, NFKBIA, NKX3-1, NOV, NOX4, NR4A2, NRP1, PLAT, PLAUR, POU3F2, PPP1R13L, PRKCE, PTEN, PTGS2, PTPRA, PTPRE, RARB, RCAN1, RUVBL1, RXRA, SEC14L2, SEMA4D, SFRP1, SLC3A2, SMO, SNAI2, SPHK1, SPP1, SRC, SSTR2, TAX1BP3, TCF7L2, TFF3, TGFA, TGFBR2, THBS1, TIAM2, TIMP2, TIMP3, TLE1, TNFSF10, TNFSF13B, TP53BP1, TPD52, TWIST1, TXNIP, USP10, VCAN, VEGFA, XBP1, ZMAT3	123	yes
Cellular Movement	invasion of breast cancer cell lines	7.83E-06	Decreased	-2.049	ABL1, ABL2, ACSL4, ADAMTS1, ATP6V0A4, BCAR3, BHLHE41, CALR, CAV1, CBL, CCNA2, CDKN1B, CTSK, CXCR4, DIAPH2, DIAPH3, FAS, FGF2, FHOD1, FURIN, HAS2, HBP1, HIF1A, HMMR, ID1, ID2, IRS2, ITGB3, JUN, LIMK1, LOX, MCAM, MMP9, MST1, NAMPT, NFKBIA, PAK4, PARVB, PLAUR, PPM1F, PTGS2, PTK2, RNF144A, RUVBL1, SIM2, SP100, SPP1, SRC, STMN1, TFAP2A, TFAP2C, TGFBR2, TNFSF10, TWIST1, WWTR1, ZEB2	56	yes
Cancer	pulmonary adenoma	1.04E-03	Decreased	-2.041	BECN1, CDKN1B, CDKN2C, FOXM1, FOXO3, FOXO4, LZTS1, MYC, NUDT1, SSBP2, XPC	11	no
Respiratory Disease	pulmonary adenoma	1.04E-03	Decreased	-2.041	BECN1, CDKN1B, CDKN2C, FOXM1, FOXO3, FOXO4, LZTS1, MYC, NUDT1, SSBP2, XPC	11	no
Cancer	tumorigenesis of malignant tumor	5.73E-06	Decreased	-2.032	AFF1, AHR, ANK3, ASXL2, ATM, ATP2A2, AURKB, BCL2, BECN1, BIN3, BIRC5, BMF, BRCA1, CABLES1, CCND1, CDKN1A, CDKN1B, CDKN2C, CTLA4, DCBLD2, DCC, DDB2, DKC1, DTNA, E2F2, EGR1, ERRFI1, FEN1, FGF2, FNDC3B, FOS, FOXM1, GDF15, GLI2, GSTM1, H2AFX, HIP1, HMGA2, ID1, IFNAR1, IFNAR2, IGFBP3, IL1B, ING1, ING2, JARID2, JUN, KIF11, KRAS, LTBR, LZTS1, MCL1, MT1E, MXI1, MYB, MYC, NFIA, NFKBIA, NQO1, NUDT1, PIM2, PLAUR, PMS2, PRDX1, PRICKLE2, PRKAR1A, PRKCE, PTEN, PTGS2, PTPRE, ROCK2, RUNX1, RUNX3, SIRT2, SPHK1, SRC, SSBP2, STAT2, TGFBR2, THBS1, TNFSF10, TP53BP1, TPR, TRIM24, VEGFA, XPC, ZEB2	87	no
Tissue Development	myelination of nervous	3.22E-04	Decreased	-2.031	ADAM19, BDNF, DICER1, EGR2, FYN, GJB1, IL11RA, IL1B, LAMC1, MAL, MBP, MYO5A, P2RX7, PMP22, POU3F1, PTPRE, RTN4	17	no
Nervous System Development and Function	myelination of nervous tissue	3.22E-04	Decreased	-2.031	ADAM19, BDNF, DICER1, EGR2, FYN, GJB1, IL11RA, IL1B, LAMC1, MAL, MBP, MYO5A, P2RX7, PMP22, POU3F1, PTPRE, RTN4	17	no
Cellular Assembly and Organization	formation of actin stress fibers	5.41E-05	Decreased	-2.027	ABL2, AKAP13, ARAP1, ARHGAP18, ARHGEF1, ARHGEF11, ARHGEF17, ARHGEF2, ARHGEF3, BOP1, C3AR1, CAV1, CDKN1A, CHRM1, CNTNAP1, CTGF, DAB2, DIAPH3, F2R, FGF2, FHOD1, FYN, GNA12, GNA13, GNG12, HAX1, HDAC6, IL8, KANK2, KDR, KLF2, KRAS, LIMK1, LMCD1, LPAR1, MAP2K1, MCF2L, MYLK, NCKIPSD, NOX4, PAK4, PLAT, PPM1F, PRKCE, PTK2, PTPRA, RND3, ROCK1, ROCK2, SDC4, SEMA4D, SORBS1, SORBS3, SPRY2, SRC, STARD13, TNC, TNS3, VASP, VEGFA, ZYX	61	no
Cellular Function and Maintenance	formation of actin stress fibers	5.41E-05	Decreased	-2.027	ABL2, AKAP13, ARAP1, ARHGAP18, ARHGEF1, ARHGEF11, ARHGEF17, ARHGEF2, ARHGEF3, BOP1, C3AR1, CAV1, CDKN1A, CHRM1, CNTNAP1, CTGF, DAB2, DIAPH3, F2R, FGF2, FHOD1, FYN, GNA12, GNA13, GNG12, HAX1, HDAC6, IL8, KANK2, KDR, KLF2, KRAS, LIMK1, LMCD1, LPAR1, MAP2K1, MCF2L, MYLK, NCKIPSD, NOX4, PAK4, PLAT, PPM1F, PRKCE, PTK2, PTPRA, RND3, ROCK1, ROCK2, SDC4, SEMA4D, SORBS1, SORBS3, SPRY2, SRC, STARD13, TNC, TNS3, VASP, VEGFA, ZYX	61	yes
Tissue Development	formation of actin stress fibers	5.41E-05	Decreased	-2.027	ABL2, AKAP13, ARAP1, ARHGAP18, ARHGEF1, ARHGEF11, ARHGEF17, ARHGEF2, ARHGEF3, BOP1, C3AR1, CAV1, CDKN1A, CHRM1, CNTNAP1, CTGF, DAB2, DIAPH3, F2R, FGF2, FHOD1, FYN, GNA12, GNA13, GNG12, HAX1, HDAC6, IL8, KANK2, KDR, KLF2, KRAS, LIMK1, LMCD1, LPAR1, MAP2K1, MCF2L, MYLK, NCKIPSD, NOX4, PAK4, PLAT, PPM1F, PRKCE, PTK2, PTPRA, RND3, ROCK1, ROCK2, SDC4, SEMA4D, SORBS1, SORBS3, SPRY2, SRC, STARD13, TNC, TNS3, VASP, VEGFA, ZYX	61	yes
Cancer	pituitary cancer	6.23E-04	Decreased	-2.021	ACTA2, ANXA2, ARHGEF2, BCL3, BDNF, CCNB2, CCND1, CDKN1B, CDKN2C, CEBPD, CHKA, CHRNB1, DAPK1, DKC1, E2F3, EGR1, FOXO3, FOXO4, GNB4, HMGA1, HMGA2, HMGB2, HMOX1, ID1, LAMB3, MIA, MMP9, NFIB, NR3C1, NRIP1, NUSAP1, PAM16, PTPRM, RAC2, SEMA4D, SOX2, TFAP2A, TGFA, TUBB4A	39	no
Reproductive System Disease	pituitary cancer	6.23E-04	Decreased	-2.021	ACTA2, ANXA2, ARHGEF2, BCL3, BDNF, CCNB2, CCND1, CDKN1B, CDKN2C, CEBPD, CHKA, CHRNB1, DAPK1, DKC1, E2F3, EGR1, FOXO3, FOXO4, GNB4, HMGA1, HMGA2, HMGB2, HMOX1, ID1, LAMB3, MIA, MMP9, NFIB, NR3C1, NRIP1, NUSAP1, PAM16, PTPRM, RAC2, SEMA4D, SOX2, TFAP2A, TGFA, TUBB4A	39	no
Neurological Disease	pituitary cancer	6.23E-04	Decreased	-2.021	ACTA2, ANXA2, ARHGEF2, BCL3, BDNF, CCNB2, CCND1, CDKN1B, CDKN2C, CEBPD, CHKA, CHRNB1, DAPK1, DKC1, E2F3, EGR1, FOXO3, FOXO4, GNB4, HMGA1, HMGA2, HMGB2, HMOX1, ID1, LAMB3, MIA, MMP9, NFIB, NR3C1, NRIP1, NUSAP1, PAM16, PTPRM, RAC2, SEMA4D, SOX2, TFAP2A, TGFA, TUBB4A	39	no
Endocrine System Disorders	pituitary cancer	6.23E-04	Decreased	-2.021	ACTA2, ANXA2, ARHGEF2, BCL3, BDNF, CCNB2, CCND1, CDKN1B, CDKN2C, CEBPD, CHKA, CHRNB1, DAPK1, DKC1, E2F3, EGR1, FOXO3, FOXO4, GNB4, HMGA1, HMGA2, HMGB2, HMOX1, ID1, LAMB3, MIA, MMP9, NFIB, NR3C1, NRIP1, NUSAP1, PAM16, PTPRM, RAC2, SEMA4D, SOX2, TFAP2A, TGFA, TUBB4A	39	no
Cellular Movement	cell movement of tumor cell lines	2.41E-10	Decreased	-2.006	ABL1, ACSL4, AFAP1, AKAP11, AKT3, ANGPT2, ANKS1A, ANXA1, ANXA2, AP2M1, ARAP3, ARPC1B, BCAN, BCAR3, BHLHE41, CALR, CAV1, CCL2, CCL20, CD36, CD97, CDC25B, CDK5, CDKN1B, CHN2, CKLF, CMTM8, COL4A3BP, CRKL, CTGF, CTSL1, CXCL13, CXCR4, DAB2, DBF4, DCBLD2, DEFB4A/DEFB4B, DGCR6L, DKK3, DNAJB4, E2F5, EFNA1, EGR1, ENPP2, EPHA2, EPOR, ETS1, ETV4, ETV5, F2RL1, FADD, FBXL2, FGF2, FHOD1, FOS, FOSL1, FOXD3, FOXM1, FOXO3, FURIN, FYN, GAB1, GDF15, GEMIN5, GNA13, GOLGA2, HAS2, HAS3, HAX1, HBEGF, HDAC6, HIF1A, HIPK2, HMMR, HMOX1, ID1, IGFBP3, IL8, ING4, ITGA4, ITGA6, ITGB3, JUN, KANK1, KDR, KIDINS220, KIT, KLF2, KLF4, KRAS, LAMB3, LIF, LIMK1, LMCD1, LOX, LPAR1, LRIG1, LRP5, LYN, MAP2K1, MAP3K1, MAP3K7, MAP4K4, MC1R, MCAM, MCF2L, MDK, MERTK, MITF, MMP9, MSN, MST1, MYB, MYO10, NDRG1, NEDD9, NFKBIA, NOV, NRCAM, NREP, NRP1, PA2G4, PGF, PHB, PIK3C2B, PIK3CD, PLAUR, PLCL1, PLGRKT, PLXNA1, PLXNB1, PPFIA1, PPM1F, PREX1, PRKCE, PRKD1, PRNP, PTEN, PTGS2, PTK2, PTP4A2, PTPN12, PTPRM, PTPRZ1, PVR, RAB21, RAB27A, RALA, RALB, RAP1GAP, RARRES1, RGS1, RNF144A, RUNX2, RUVBL1, SCNN1A, SEMA3A, SEMA4D, SH3PXD2B, SHC4, SLC16A4, SLC2A1, SNAI2, SNX27, SOD2, SPHK1, SPP1, SPRY2, SRC, SSH1, ST3GAL5, ST6GAL1, STARD13, STMN1, SYK, SYNM, TBXAS1, TFAP2A, TFAP2C, TGFA, THBS1, THBS2, TIMP2, TLR4, TNC, TNFSF10, TP53INP1, TRIP6, TUBA1C, TWIST1, VASP, VEGFA, VEGFB, VPS28, WASL, WWTR1, ZYX	197	yes

Table S5. Upstream regulator analysis, by IPA software, of genes significantly modulated by the association of TRAIL with AZD6244

Results shown here are based on the set of genes identified by areas highlighted in fuchsia in Fig. S8A.
See supplementary methods for the meaning of the p values and of the z score statistics.

Upstream Regulator	Log Ratio	Molecule Type	Predicted Activation State	Activation z-score	p-value of overlap	Target molecules in dataset
MITF	0.442	transcription regulator	Activated	4.882	3.09E-14	ACO2, APOE, ATP6V1B2, ATP6V1C1, BCL2, BEST1, CA14, CAPN3, CCNG2, CDK5R1, CHKA, CLCN7, CTSK, DAPK1, DSTYK, EDNRB, ESRP1, FAM53B, FOS, GMPR, GREB1, GYG2, HIF1A, HPGD, HPS4, IL8, IRF4, ITGA4, ITPKB, KCNN2, KIAA1598, KIT, MBP, MC1R, MDH1, MICAL1, MITF, MLANA, NR3C1, PHACTR1, PIR, PLA1A, PPM1H, QDPR, RAB27A, SEMA6A, SORT1, SOX6, ST3GAL6, STX7, STXBP1, TBC1D16, TBX2, TFAP2A, TMC6, TMCC2, TRPM1, TXNIP, TYRP1, USP48, VAT1
HNF4A		transcription regulator	Activated	2.058	3.90E-06	ANKRA2, ANKZF1, APH1A, APOE, ARG2, ARHGEF19, AS3MT, ATAT1, ATF6B, ATP6V1H, AVPI1, BAZ1A, BCCIP, BMI1, BPGM, BRD8, BSDC1, BTG1, C11orf1, C11orf71, C12orf52, C1orf123, C1S, C2, C21orf59, C22orf28, C2orf44, C4orf19, CAV1, CCDC115, CCDC41, CCDC53, CCNA2, CCND1, CCNG2, CD3EAP, CD55, CDC25A, CDC6, CDK2AP2, CDK5, CDK5RAP3, CDKN1A, CDKN1B, CEBPB, CEBPD, CEP95, CES2, CETN3, CHERP, CHMP1B, CIAO1, CLDN1, CLTCL1, COPS7B, COPS8, CPB2, CRADD, CRIPT, CRKL, CROT, CRYZ, CYP27A1, CYTH2, DBP, DCAF13, DDX10, DDX18, DHDDS, DHRS4, DLG4, DNAJB4, DPH5, DSN1, DUSP3, DUSP6, E2F5, EGR1, EIF4G1, EIF5, ELP5, EMC2, EMG1, ENC1, EPHA2, EPM2AIP1, ERCC5, ERI2, ERLIN1, ERO1L, EXOSC2, F12, FAM107B, FAM216A, FAM46A, FAS, FEM1B, FEN1, FGF13, FKBPL, FOXO1, FOXRED1, FTSJ1, FURIN, FXYD6, FYCO1, FZD1, G3BP2, GAB1, GALM, GAS1, GDF15, GJB1, GLCE, GNL3, GOLGA2, GPR137, GPR37, GSPT1, GSTA4, GSTK1, GSTO1, GTF2B, GTF2I, GTPBP3, GUSB, HADHB, HBP1, HDAC6, HEXA, HEY1, HIF1A, HIST1H2BD, HIST1H4A , HIST2H2AA3/HIST2H2AA4, HIST2H2BE , HLA-F, HMGB2, HPS5, HTRA2, IER5, IFNAR1, IFT122, IL11RA, IL1RAP, INCENP, ING4, INTS7, IPO13, ISOC1, ISOC2, ITGA6, JUN, KCNN2, KIF20A, KLF15, KLHL20, KRR1, LAMTOR2, LARS2, LDB1, LHX6, LIMS1, LPCAT3, LPGAT1, LRP5, MAP2K5, MAP3K3, MAP3K7, MCEE, MDH1, MED23, MGEA5, MGST1, MICU1, MID1, MINA, MOCOS, MPP1, MRPL3, MRPL32, MRPL44, MRPS12, MRPS18C, MRTO4, MSMB, MST1, MT1X, MT2A, MTHFS, MUT, MYC, N4BP2L2, NAMPT, NARS2, NCBP2, NCOA4, NDC80, NDRG1, NDUFB3, NEURL2, NOC3L, NOLC1, NOP16, NR2F1, NR5A2, NUAK1, NUCB1, NUDT2, NUDT6, NUP54, OTUD6B, PAAF1, PAGR1, PAN2, PARP4, PCDH20, PDK4, PELO, PEPD, PEX11B, PEX16, PFKFB4, PHB, PI4KB, PIK3R3, PINK1, PITPNB, PNMA1, PNO1, PNP, POLR1B, POLR1C, POLR3E, POLR3G, PPARGC1A, PPFIBP1, PPIL1, PPP1R12B, PPP1R3C, PPP2R3C, PRCP, PRELID2, PRICKLE4, PRKCE, PRMT1, PROM1, PRPF38B, PSAT1, PSMB5, PSMD10, PTGDS, PTK2, PTPRE, QTRTD1, RAB2A, RABEPK, RAP1GAP, RARA, RARB, RBM23, RHPN2, RIOK1, RNASE4, ROCK1, RPS6KC1, RQCD1, RRM1, RTCA, RTFDC1, RXRA, SAMM50, SAT2, SEC31A, SEMA3C, SEPSECS, SERPINB8, SETDB2, SIRT2, SLC16A6, SLC22A18AS, SLC25A19, SLC25A20, SLC25A40, SLC30A7, SLC38A1, SNAI2, SNAP23, SNAPC1, SNX17, SNX5, SPATA5L1, SPCS2, SPP1, SRPRB, SRSF2, SSU72, STAM, STARD10, STIM1, STK19, STK24, STOML1, STX18, SUCLG1, SULT1A3/SULT1A4, SYNJ2BP, TAOK3, TBC1D15, TBC1D16, TCF7L2, TEX10, TFB1M, TFB2M, TIMM21, TIMP3, TMC6, TMEM140, TMEM187, TMEM216, TMEM59, TMEM63A, TMEM87B, TMUB2, TNC, TOM1, TOR2A, TPP2, TPX2, TRAIP, TRAPPC6A, TRIM24, TRIM4, TRMT1L, TRPC4AP, TSPAN14, TTC19, TUBB4A, TUFT1, TXNIP, UBQLN2, UCHL5, UGT2B11, UPF3B, UQCC, UTP23, VIPAS39, WASL, WDR12, ZC3H10, ZC3H15, ZCCHC9, ZDHHC6, ZFYVE19, ZKSCAN5, ZNF193
KLF2	-0.211	transcription regulator	Activated	2.748	2.10E-03	ADM, ANGPT2, BCL3, CCL2, CD55, CDKN1A, CTGF, CXCR4, EFNA1, EPAS1, HIF1A, ID1, ID3, IL1B, IL8, ITGB3, ITGB5, KCNN4, KDR, MAP3K5, MT2A, MYC, NDRG1, NFKBIA, NQO1, PGF, PPAP2B, PTGDS, PTGS2, RALA, RUNX2, SLC2A1, TCF4, THBS1
MEOX2		transcription regulator	Activated	2.138	2.73E-03	ANGPT1, CCL2, CCL20, CDKN1A, EFNA1, FGF2, HBEGF, ICAM1, ID1, ID3, IL8, ITGB3, ITGB5
TFEB		transcription regulator	Activated	2.395	2.65E-02	ATP6V1H, CLCN7, CTSF, HEXA, MCOLN1, SCPEP1, TMEM55B, TYRP1, VEGFA
SOX9		transcription regulator	Activated	2.198	3.13E-02	BMI1, CDKN1A, CTGF, CXCR4, FOS, KIT, MIA, MITF, PTGDS, RUNX2, SNAI2, SOX8, TCF7L2, VEGFA
CIITA		transcription regulator	Activated	2.172	7.71E-02	CCND1, CD74, GCNT2, HLA-DPA1, HLA-DRA, HLA-DRB1, MMP9
FOXA1		transcription regulator	Activated	2.021	2.08E-01	ACTA2, ACTG2, ANXA1, CD58, EFHD1, ELK3, FNDC3B, FSTL1, HK1, LHFP, LYN, MALT1, NR4A2, NRIP1, PRNP, TFF3, TPM2, TRIM2, UGT2B17, XBP1
MYOCD		transcription regulator	Activated	2.383	3.26E-01	ACTA2, ATP2A2, CDKN1A, FOS, MEF2C, MYLK, VCAN
IKZF1		transcription regulator	Activated	2.615	3.52E-01	ADAM19, CCND2, CDKN1B, EPOR, FGF13, FGFR3, HES1, HNRPLL, KIT, LHFP, LHFPL2, MYC, PPP1R9A, PRNP, RAI14, SASH1, SLC27A3
GATA6		transcription regulator	Activated	2.069	4.62E-01	ACTA2, CAV1, CDKN1A, DAB2, MEF2C, MYLK, SEMA3C, TNFSF10

Upstream Regulator	Log Ratio	Molecule Type	Predicted Activation State	Activation z-score	p-value of overlap	Target molecules in dataset
TGFB1		growth factor	Inhibited	-3.235	4.82E-20	ABCA1, ABCC2, ABCD1, ABCE1, ABL1, ACAA2, ACSL3, ACSS1, ACTA2, ACTG2, ACTN1, ACVR1, ADAM19, ADK, ADM, ADORA2B, AHNAK, AHR, AIF1L, AIM2, ALDH18A1, AMD1, ANGPT1, ANGPTL4, ANXA2, APOE, AQP11, ARHGEF19, ARID5B, ARL4A, ASNS, ATM, ATXN1, B3GALT2, BAX, BCL2, BCL2L11, BCL3, BDNF, BECN1, BHLHE40, BIRC5, BMF, BMI1, BMP1, BTG1, C1S, C2, C20orf24, CALCOCO2, CALM1 , CAMK2G, CASP1, CASP4, CAV1, CCL2, CCL20, CCNA2, CCNB2, CCND1, CCND2, CCNG2, CCR1, CD36, CD55, CD59, CDC25A, CDC25C, CDC42EP4, CDK5R1, CDKN1A, CDKN1B, CDKN2C, CEBPB, CELF2, CENPA, CKS1B, CLCA2, CLIC4, CMTM5, CNIH, COL16A1, CRYGS, CSPG4, CSRP2, CTGF, CTLA4, CTPS1, CTSH, CTSK, CXCR4, CYB561, DAB2, DAPK1, DDB2, DDIT4, DDX21, DKC1, DKK3, DLL3, DNAJB4, DSP, DUSP1, DUSP4, DYNLL1, DYRK2, EDNRB, EGLN1, EGR1, EGR2, EGR3, EIF4A3, ELK3, EOMES, EPHA2, ERCC5, ESPL1, ESRP1, ETS1, F2R, F2RL1, FABP5, FAM3C, FAM53B, FAS, FCER1G, FGF12, FGF2, FLI1, FNDC3B, FOS, FOXO1, FOXO3, FSTL3, FTH1, FTL, FURIN, FXYD6, FYN, FZD1, FZD2, GALM, GAS1, GBP1, GDF15, GDPD5, GLCE, GLI2, GLRX2, GMPR, GNA13, GNB4, GPR19, GPR83, GPRC5A, GUSB, H1FX, HAS2, HAS3, HBEGF, HES1, HEXA, HEY1, HIF1A, HLA-DRB1, HMGA1, HMGA2, HMOX1, HNMT, HOXD1, HPGD, HSD17B10, HSPB1, ICAM1, ID1, ID2, ID3, IER3, IFI16, IGFBP3, IGFBP5, IL12A, IL17D, IL1B, IL8, ING1, ITGA4, ITGA6, ITGB3, ITGB5, ITPR2, JUN, JUND, KCNG1, KDELR3, KDM5B, KDR, KIAA1199, KIT, KLF15, KLF2, KLF4, KLF9, KRAS, KRT18, LAMB3, LAMC1, LDB1, LIF, LIMS1, LOX, LPCAT3, LPL, MAF, MAP2K1, MAP2K3, MAP3K11, MBNL2, MCM2, MEF2C, MGEA5, MID1, MITF, MMP9, MPP6, MSMB, MSMO1, MSN, MTHFD2, MTRR, MXD3, MXD4, MXI1, MYB, MYC, MYLK, MYO10, NAMPT, NCAPG, NDC80, NEDD9, NEK2, NFIB, NFKBIA, NLRP3, NNMT, NOC3L, NOP58, NOV, NOX4, NPAS2, NR4A2, NRP1, NT5E, NUAK1, NUPR1, OARD1, OPN3, P4HA1, PA2G4, PAPPA, PDLIM4, PDLIM5, PDXK, PHLDA2, PIK3CD, PINK1, PLA1A, PLAT, PLAUR, PLCL1, PMEPA1, PMM1, PNO1, PNP, PPP1R13B, PPP1R3C, PPP2R5A, PRODH, PROM1, PTEN, PTGDS, PTGES, PTGS2, PTK2, RAB6A, RAB9A, RACGAP1, RALB, RARA, RBMS1, RIN1, RUNX1, RUNX2, RUNX3, RXRA, SCCPDH, SDC4, SELENBP1, SEMA3A, SFRP1, SLC16A3, SLC20A1, SLC2A1, SLC2A3, SLC39A14, SLC39A8, SLC7A1, SLC7A5, SMAD6, SMTN, SNAI2, SNTB2, SOD2, SOX4, SPHK1, SPOCK1, SPP1, SPRY1, SRC, SRI, SRM, SRSF2, SSTR2, ST3GAL5, STC2, TACC2, TAX1BP3, TCN2, TGFA, TGFBR2, TGFBR3, TGIF1, THBS1, TIMP2, TIMP3, TJP2, TLE4, TLR4, TNC, TNFRSF12A, TNFSF13B, TPM2, TPST2, TRIM2, TRIM9, TSC22D1, TSC22D3, TUBB3, TWIST1, TXNIP, UCK2, UST, VASP, VAT1, VCAN, VDR, VEGFA,
MYC	-1.011	transcription regulator	Inhibited	-2.033	1.85E-18	ABCE1, ACAT1, ACSL4, ACTN1, ADD1, ADK, ADM, AIMP2, AK2, AKAP12, ALDH18A1, AMD1, ANGPT1, ANGPT2, ASNS, AURKB, BAX, BCKDHB, BCL2, BDNF, BIRC5, BMI1, BOP1, BRCA1, CASP1, CAV1, CCNA2, CCNB2, CCND1, CCND2, CCNG2, CDC25A, CDC25B, CDC25C, CDK6, CDKN1A, CDKN1B, CEBPD, CHKA, CHRNB1, CLIC4, CLUH, COL15A1, COL5A2, CSDA, CSPG4, CSRP2, DBI, DDB2, DDIT3, DDX18, DDX21, DKC1, DKK1, DUSP1, DUSP6, E2F2, E2F3, EGLN1, EGR2, EIF4A1, EIF4G1, F2R, FABP5, FAM129A, FAP, FAS, FASN, FBN2, FOS, FOSL1, FOXM1, FSTL1, FTH1, GART, GAS1, GCLM, GFPT1, GOLGA2, GRPEL1, GSR, GTF2F2, HAS2, HIF1A, HIST1H4A , HIVEP2, HK2, HMGA1, HMOX1, HNRNPAB, HNRNPD, HSPB1, HSPD1, ICAM1, ID1, ID2, ID3, IER3, IL1RAP, IL8, IMPA2, IRF9, ITGA6, JARID2, KDR, KLF4, KRAS, LAMB3, LGMN, LIMA1, LIMS1, LOX, LXN, MAPKAPK5, MBP, ME2, METAP1, MGAT1, MINA, MITF, MMP9, MNT, MRPL12, MSN, MT1E, MTHFR, MYC, NCL, NDRG1, NOLC1, NOP56, NOP58, NUCB1, NUDC, OAS1, OLIG1, PFAS, PFKFB3, PHB, PHF20, PLA1A, PLAUR, PLP1, PMAIP1, PMP22, PNO1, POLR1B, PPAT, PPP1CC, PPP1R15A, PRMT1, PRODH, PSAT1, PTEN, RAB40B, RANBP1, RARA, RARRES1, ROCK1, ROCK2, RPL6, RPL7, RPS7, RRP1B, RRS1, RTN2, RUVBL1, SAT1, SCPEP1, SERBP1, SFRP1, SFXN1, SLC16A3, SLC1A5, SLC25A19, SLC2A1, SLC2A3, SLC38A1, SLC3A2, SLC7A5, SNHG12, SNRPC, SOD2, SOX2, SOX6, SPP1, SRM, SRSF2, ST3GAL1, ST3GAL4, STMN1, TFDP1, TGFBR2, THBS1, THBS2, TIMM23, TIMP2, TLE1, TLE4, TNC, TNS3, TP53I3, TPD52, TPP2, TXNIP, UBE2C,

ERBB2		kinase	Inhibited	-2.222	2.76E-16	ABL1, ACAA2, ACSL4, ACTA2, ADAM19, ADIPOR2, AHNAK, ALAD, ANG, ANGPTL2, ANGPTL4, ANXA2, ATP1B3, BAG2, BCKDHB, BCL2, BCL3, BHLHE40, BIRC5, BMP1, BNIP3, CCL20, CCNA2, CCNB2, CCND1, CCND2, CD36, CD59, CDC25A, CDC25C, CDK6, CDKN1A, CDKN1B, CDKN2C, CENPA, CHST2, CKS1B, COL5A2, CRADD, CTGF, CYB561, DDIT3, DECR1, DUSP6, EGR1, ELF2, ELK3, EPS15, ESPL1, ETV1, ETV4, ETV5, F2R, FAM134B, FASN, FEN1, FOS, FOXO1, FSTL1, FSTL3, FZD4, G3BP1, GHR, GNAI1, HADHB, HAS2, HES1, HIF1A, HIST1H4A , HMGA2, HMGB2, HSPB1, HSPB8, ID1, ID2, IDH1, IGF2R, IGFBP3, IGFBP5, IL1B, IL8, IRX3, ITGA6, ITGB3, ITPR2, JUN, KCNN4, KDM5B, KDR, KIT, KLF4, LAMB1, LXN, MACF1, MAP2K3, MARCKS, MITF, MMP9, MSMB, MXD3, MXI1, MYC, MYO10, NCAPG NDC80, NDRG1, NEDD9, NEK2, NFIB, NNMT, NOX4, NRP1, PDE8A, PDK4, PDLIM4, PFKFB3, PHLDA2, PHLDB1, PHYH, PIK3CD, PINK1, PLAT, PLAUR, PMEPA1, PPP2R2B, PPP2R5A, PTEN, PTGES, PTGS2, QKI, RALB, RASA3, RNF149, SEC61A1, SFRP1, SLC2A1, SMTN, SNAI2, SORBS1, SOX4, SPAG1, SPOCK1, SQLE, SRC, ST3GAL4, ST3GAL6, STK17B, TAX1BP3, TFAP2C, TGFA, TGIF1, THBS1, TIMP3, TNC, TPD52, TSC22D1, TUBB4A, UBE2C, UCK1, VCAN, VDR, VEGFA, WBP5, WFS1, WISP2, WWC1, XBP1, ZNF703, ZWINT
NUPR1	0.299	transcription regulator	Inhibited	-6.095	1.29E-15	ABL2, ACSS1, ADM, AEN, AKAP12, ANK3, AP5M1, AS3MT, AVPI1, BNIP3, BRCA1, BRI3BP, BTG1, C2orf44, C3orf58, C8orf58, CCDC77, CCNA2, CCNB2, CDC25C, CDCA3, CDCA8, CDKN1B, CEBPB, CENPL, CHMP1B, CHTF8, CNPY4, COQ10A, CREB5, CROT, CTPS2, CXCR4, DDIT3, DGCR8, DSN1, DUSP5, EGLN1, EME1, ESPL1, ETS1, ETV1, FAM111B, FAM115C, FOXO3, FUCA1, GCNT2, GDF15, GPSM2, GRAMD3, GSTA4, GTSE1, HBEGF, HILPDA, HIST1H4A , HK2, HSPA2, IFIT2, IGF2BP3, IL13RA1, IL8, ING2, IP6K2, IPP, IRS2, ITPR3, KANK2, KIF11, KIF20A, KIF2C, KLF4, LARS2, LHFPL2, LIF, LRP8, LRRC16A, LRRC33, MAFG, MCM10, MEGF9, MGLL, MT1X, MTFMT, MYC, NAPEPLD, NCKIPSD, NDRG1, NFIB, NT5DC3, NUP50, NUPR1, OSGEPL1, PER3, PFKFB3, PFKFB4, PHLDA1, PM20D2, PMP22, PPP1R15A, PRELID2, PRNP, PXDC1, RAB17, RAB32, RAB38, RBMS1, RCL1, RFTN2, RILPL2, RIMKLA, RNF122, RNF19B, SAMD4A, SAT1, SH2D5, SHPK, SIX4, SLC16A10, SLC16A6, SLC25A20, SLC2A1, SLC39A8, SRGAP2, STX3, SUOX, TCEANC2, TFAP2A, TMEM158, TMEM167B, TMEM19, TMEM194A, TNS3, TOLLIP, TP53BP1, TRAFD1, TRIB1, TRIB3, UAP1, UBIAD1, UPP1, VPS45, WDR91, XBP1,
HGF		growth factor	Inhibited	-3.636	3.22E-15	ACTA2, ADAMTS1, AHR, AIMP2, AKAP12, AKAP13, ANGPT2, ANGPTL2, ANGPTL4, ANK3, ASNS, ATM, AURKB, BAG2, BCL2, BIRC5, CALM1 , CASP1, CAV1, CCL2, CCND1, CCNG2, CD3EAP, CDC25A, CDC25B, CDC25C, CDC6, CDKN1A, CDKN1B, CDKN2C, CKS1B, CTGF, CTSK, CXCR4, DBF4, DDIT3, DDX21, DIAPH2, DKC1, DUSP4, DUSP6, DYNLL1, EFNB2, EGR1, EPHA4, ERRFI1, ETS1, FAM3C, FASTK, FEN1, FOS, FOSL1, FOXM1, GAB1, GNA13, GPSM2, GRK5, HBEGF, HES1, HK1, HK2, HMMR, HMOX1, HTR7, ICAM1, IGFBP3, IL1B, IL27RA, IL8, ISG20, ITGA6, ITPR3, KIF11, KIF2C, KRR1, KRT18, LGMN, LRP8, MAP2K3, MCL1, MCM2, MERTK, MID1, MMP9, MYC, NAMPT, NDC80, NEK2, NEK3, NOLC1, NOP2, NPC1, NR4A2, NR5A2, NRCAM, NRIP1, NRP1, PEX5, PGF, PHB, PHLDA1, PHLDA2, PIM2, PLAUR, PMP22, PPAN, PPAP2A, PPAP2B, PTGS2, PVR, RGS20, RIN1, RNF103, RUNX2, SEMA3A, SFRP1, SLC20A1, SLC7A1, SLK, SOCS2, SOX2, SPP1, SPRY2, STC1, STK10, TGFA, TGFBR2, THBS1, TIMP3, TMEM158, TNFRSF1A, TPX2, TRAIP, TRIB1, TRIP13, TTK, UBE2C, VDR, VEGFA
RAF1		kinase	Inhibited	-2.961	5.67E-13	AKAP12, AMD1, ANXA1, BCL2, BCL2L11, BECN1, BTG1, C22orf28, CCND1, CD74, CDC25A, CDC42EP1, CDC6, CDKN1A, CDKN1B, CLDN1, CTSH, CTSL1, DCBLD2, DHRS3, DUSP4, DUSP5, DUSP6, EGR1, EGR3, FAM13A, FAS, FOS, HBEGF, HIF1A, HMGA2, HMGB3, HNRNPAB, HPCAL1, HSPB1, ID3, IER3, IGFBP3, IL12A, IL1B, ITGB3, ITGB5, JUN, LIF, LPGAT1, MCF2L, MGLL, MMP9, MYC, NOP56, PHLDA1, PHLDA2, PLAT, PLAUR, PNP, PTGS2, RAN, RGS1, RND3, SCNN1A, SEC14L2, SELENBP1, SMAGP, SPRY2, SQLE, ST3GAL4, ST6GALNAC2, TOMM40, TPM2, TSC22D1, TXNIP, VEGFA, WISP2, YPEL5
EGF		growth factor	Inhibited	-3.078	1.94E-12	ABI1, ACSL4, ACTN1, ANGPT1, ARHGEF2, ATM, ATP2A2, AURKB, B4GALT5, BCL2, BIRC5, BRCA1, CASP1, CAV1, CCND1, CCND2, CDC25C, CDC42EP1, CDK5, CDKN1A, CDKN1B, CEBPB, CEBPD, CPB2, CTGF, CXCR4, DDIT3, DUSP1, DUSP4, DUSP5, DUSP6, E2F3, EGR1, EGR2, EGR3, EPAS1, EPHA2, EPS15, ERRFI1, ETS1, ETS2, FASN, FGF2, FOS, FOSL1, FST, FSTL1, GAL3ST1, GAS1, GFPT1, GSTA4, HAS2, HAS3, HBEGF, HES1, HIF1A, HMOX1, ICAM1, ID1, ID2, IDH1, IDS, IER3, IGFBP3, IGFBP5, IL1B, IL7R, IL8, ITGB3, JUN, JUND, KRAS, LPCAT3, MCL1, MMP9, MST1, MYC, NCL, NR4A2, NRP1, PDK4, PHLDA2, PLAT, PLAUR, PPAP2B, PTGES, PTGS2, PTPN12, RALA, RRM1, RUNX1, RXRA, SCG2, SH2B3, SNAI2, SOX4, SPP1, SPRY2, SPRY4, ST3GAL1, ST3GAL4, TFAP2A, TFDP1, TGFA, TGFBR2, TGIF1, THBS1, TIMP3, TNC, TNFRSF12A, TWIST1, UGT2B17, ULK1, VAMP2, VASP, VCAN, VDR, VEGFA, ZFP36
TNF		cytokine	Inhibited	-4.265	7.22E-12	ABCA1, ABCC1, ABCC2, ABR, ABTB2, ACACA, ACADS, ADM, ADORA2B, AK2, AKAP12, ALAD, ALDH1A3, ANGPT1, ANGPT2, ANGPTL4, APOE, ARSI, ATP2A2, ATP2B1, BAX, BCKDHB, BCL2, BCL2L11, BCL2L13, BCL3, BDNF, BHLHE40, BHLHE41, BID, BIRC5, BMPR1A, BTG1, BTG3, CALR, CASP1, CASP4, CAV1, CCL2, CCL20, CCL28, CCND1, CCND2, CCR1, CCRN4L, CD36, CD55, CDC25C, CDC42EP4, CDK5R1, CDKN1A, CDKN2C, CEBPB, CEBPD, CFB, CHST2, CHSY3, CLIC4, CLTCL1, COL15A1, COL16A1, CRADD, CTGF, CTLA4, CTSF, CXCL13, CXCL16, CXCR4, CYP27A1, CYTH3, DBI, DDIT3, DEFB4A/DEFB4B, DHRS3, DKK1, DLX1, DUSP1, DUSP14, DUSP5, DUSP6, EDNRB, EFNA1, EFNB2, EGR1, EGR2, ELK3, ENPP2, EPHA2, ETS1, F2RL1, FABP5, FADD, FADS1, FAS, FASN, FCER1G, FGF2, FGFRL1, FOS, FOSL1, FOXO4, FST, FTH1, GAB1, GBP1, GCLM, GDF15, GHR, GNAI1, GNB4, GSPT1, GSTM2, GUSB, HBEGF, HERC1, HES1, HEXA, HIF1A, HIPK2, HK2, HLA-DRA, HLA-F, HMGCR, HMOX1, HPGD, HSP90AB1, HSPD1, ICAM1, ID1, IER3, IFNAR2, IFNGR2, IGFBP3, IGFBP5, IL12A, IL16, IL1B, IL7R, IL8, IRF4, IRF5, ITGA4, ITGA6, ITGB3, ITPR2, JARID2, JUN, JUND, KDR, KIF20A, KIT, KLF2, KLF4, KYNU, LAMA4, LAMB3, LAMC1, LIF, LOX, LPL, LSS, LTBR, LXN, LYN, MAFF, MAP2K3, MAP2K6, MAP3K7, MARCKSL1, MBP, MC1R, MCAM, MCL1, MGST1, MIA, MMP16, MMP9, MNT, MST1, MT1A, MT1E, MYC, MYLK, NAMPT, NEDD9, NFKBIA, NFKBIE, NLRP3, NNMT, NOV, NQO1, NR3C1, NR4A2, NR5A2, NRIP1, NRP1, NUAK1, OAS1, OGN, OLFML2B, OPTN, P2RX5, PAPPA, PCDH7, PDE4B, PHLDA1, PLAT, PLAUR, PLIN2, PLP1, PMAIP1, PNPLA8, PPAP2A, PPARGC1A, PPIF, PPP1R15A, PPP1R3C, PTEN, PTGES, PTGS2, PTPN12, QKI, RAB32, RARA, RARRES3, RASSF7, RCAN1, RGS1, RGS20, RNASE4, RND3, RNF149, RRM1, RUNX2, RXRA, SAMD4A, SAT1, SCNN1A, SDC4, SEMA3C, SERPINB8, SFRP1, SLC20A1, SLC2A1, SLC7A1, SMPD1, SMPD2, SNN, SOAT1, SOD2, SORBS1, SOX4, SPHK1, SPP1, SPSB1, SQLE, ST3GAL5, ST3GAL6, ST6GAL1, STMN1, SUPT4H1, SVIL, TBXAS1, TFAP2A, TFAP2C, TGFA, TGFBR2, TGIF1, THBS1, THBS2, TIMP2, TIMP3, TK1, TLR4, TM4SF1, TNC, TNFRSF1A, TNFRSF1B, TNFSF10, TNFSF13B, TP53I3, TP53INP1, TPP2, TPST1, TRADD, TRAFD1, TRIM63, TSC22D3, TWIST1, TXN2, TXNIP, UBD, UBE2H, UBQLN2, UCK1, VASP, VDR, VEGFA, WISP2, YWHAG, ZFP36,
VEGFA	-0.581	growth factor	Inhibited	-2.983	4.54E-11	ADAMTS1, AK4, ANGPT2, ARG2, ATP5J2, BAX, BCL2, BIRC5, BTK, CAV1, CCL2, CCND1, CD55, CDC25C, CDKN1A, CDKN1B, CEACAM1, CLIC4, CTGF, CXCR4, DECR1, DUSP4, DUSP5, E2F2, E2F3, EFNB2, EGR1, ETS1, ETS2, ETV4, ETV5, FGF2, FOS, GART, GBP1, HBEGF, HK1, HMOX1, ICAM1, ID1, ID3, IGFBP5, IL8, KDR, LDB1, MCL1, ME2, MEF2C, MMP9, MRPL3, MRPS12, PLAT, PPA1, PPAP2B, PPARGC1A, PTGS2, RCAN1, RUNX1, RUNX2, SDHB, SMTN, SNAI2, SOD2, STARD8, STC1, SYNM, THBS1, TJP2, TOMM20, TXN2, VEGFA
HIF1A	-0.809	transcription regulator	Inhibited	-2.673	8.15E-11	ACO1, ACTA2, ADAMTS1, ADM, ADORA2B, AKAP12, ANGPTL4, ASNS, ATP2A2, BCL2, BHLHE40, BHLHE41, BID, BIRC5, BNIP3, BRCA1, CAV1, CDKN1A, CDKN1B, CHKA, CTPS1, CXCR4, EGLN1, EPAS1, EPOR, ERO1L, ETS1, FAM13A, FOS, FURIN, GHR, H2AFX, HIF1A, HILPDA, HIST1H2AC, HIST1H2AG , HIST1H4A , HIST2H2AC, HK2, HMGCL, HMOX1, ID2, IGFBP3, IGFBP5, IL1B, IL8, IRS2, ITGB3, JUN, KIAA1199, KRT18, LOX, MAFF, MCL1, MEF2C, MITF, MMP9, MYC, NDRG1, NOV, NOX4, NT5E, P4HA1, PFKFB3, PFKFB4, PGF, PLAUR, PMAIP1, PPFIA4, PTGS2, QKI, SCG2, SDC4, SIRT2, SLC16A4, SLC25A37, SLC29A1, SLC2A1, SLC2A3, SLC40A1, SPHK1, ST3GAL1, STC2, TCF4, TGFA, TMEM19, TMEM45A, VASP, VEGFA
IL2		cytokine	Inhibited	-3.174	3.66E-10	ABCC1, ADCY3, AHR, ATM, BAX, BCCIP, BCL2, BHLHE40, CAPN5, CASP1, CCL2, CCND1, CCND2, CCNG2, CD2, CD58, CD59, CDC25A, CDC6, CDK5R1, CDK6, CDKN1A, CDKN1B, CDKN2C, CEACAM1, CLSPN, CSRNP1, CTLA4, CTPS1, CXCR4, CYP27A1, DAPK1, DDX21, DKC1, DUSP4, DUSP5, DUSP6, EEF1E1, ELF1, ENPP2, EOMES, ETS1, FAIM3, FAS, FCER1G, FGF2, FOS, FOXO1, FOXO3, FYN, GART, GDF15, GNL3, HK2, HSPD1, ICAM1, IER3, IFNGR2, IL1B, IL27RA, IL7R, IL8, ING4, IP6K2, IRF4, ISG20, ITGA4, ITK, JUN, JUND, KIT, KLF13, LIF, MAF, MAP2K1, MAP2K6, MMP9, MYB, MYC, NOP2, PDCD2L PHB, PHLDA1, PHLDA2, PIK3R3, PIM2, PMAIP1, PNP, POLR1B, POLR1C, PPAT, PPP2R2B, PTGS2, PUS1, SATB1, SESN1, SESN3, SLC30A1, SNAP23, SOCS2, SPP1, SPRED1, SPRED2, STAM, STK17B, TNFRSF12A, TNFRSF1A, TNFRSF1B, TNFSF10, TP53INP1, TRAF5, TRIB3, UCK2, UPP1, VEGFA, XBP1, YARS, ZHX1
AGT		growth factor	Inhibited	-2.514	5.90E-10	ACTA2, ADD3, ADM, ADORA2B, ANGPT1, ANGPT2, ANK3, ARHGEF11, ARID2, ATP1B1, ATP2B1, BAX, BCL2, BECN1, BRCA1, CALR, CAV1, CCL2, CCND1, CCND2, CD36, CDC25A, CDKN1A, CDKN1B, COL11A2, CREB5, CTGF, CYTH3, DAB2, DUSP1, EDNRB, EFNB2, EGR1, ERRFI1, ETS1, ETV1, FASN, FGF13, FGF2, FOS, FOXO3, FRAT2, GAS2, GFPT1, GRK5, HAS2, HBEGF, HES1, HEY1, HIF1A, HMGB2, HMGCR, HMOX1, HSPB1, ICAM1, ID3, IDH1, IGFBP3, IGFBP5, IL8, ITGB3, ITGB5, ITPKB, JUN, LAMB1, LIF, LSS, MATN2, MGST1, MMP9, MSMO1, MXD4, MYB, MYC, NCALD, NCL, NDRG1, NOX4, NR4A2, NREP, PGF, PIR, PNKD, PPFIBP1, PPP1R3C, PTEN, PTGS2, RCAN1, RRAS, SCNN1A, SEMA6A, SGCD, SLC2A1, SOD2, SOX4, SOX6, SPP1, SPRY1, STC1, TBX2, TGFA, TGFBR2, TNFRSF12A, TUBB3, TXNIP, VEGFA, WASL, ZEB2, ZFP36
KRAS	0.529	enzyme	Inhibited	-2.271	1.82E-09	ABCC4, AHR, AMPH, ATP1B1, BCL2, BMI1, BNIP3, CASP1, CCND1, CD74, CDKN1A, CDKN1B, DCLK1, DDAH1, DDIT3, DUSP4, DUSP5, DUSP6, E2F5, EFNA1, EIF5, ERO1L, ETV1, ETV5, FOS, FOSL1, FSTL1, FTH1, GAS1, GNA13, GTF2I, HADHB, HEXA, HIF1A, HLA-DRB1, HMGA2, ID1, IER3, IGF2BP2, IL1B, IL8, IP6K2, IRF9, ITGB3, KRAS, LAMB3, LOX, LTBP4, MAP2K1, MCAM, MICB, MTRR, MYLK, NPC1, NQO1, NR3C1, NR5A2, NT5E, OAS1, PHC2, PLA1A, PLAT, PMAIP1, PPFIBP1, PTEN, PTGS2, PTK2, RNASE4, ROCK1, SDC4, SLC3A2, SOAT1, SORT1, SP100, SPRY2, STAT2, TEAD2, TGFBR2, THBS1, TIMP2, TNC, TPM2, UPP1, VCAN, VEGFA
IFNG		cytokine	Inhibited	-2.168	3.24E-09	ABCA1, ABI1, ACO1, ACSS1, ADM, ADORA2B, ADRBK1, AHR, AIF1L, AIM2, ALDH1A3, ANGPTL4, ANKS1A, AQP11, ARG2, ATP2A2, BAX, BCAN, BCL2, BCL2L11, BDNF, BECN1, BID, BMF, BTG1, C1R, CALCOCO2, CAPN3, CASP1, CASP4, CAV1, CCL2, CCL20, CCL28, CCNA2, CCND1, CCND2, CCR1, CD2, CD200, CD276, CD36, CD55, CD74, CDK5R1, CDKN1A, CDKN1B, CEACAM1, CEBPB, CEBPD, CFB, CHAC1, CIRBP, CLIC4, COL5A2, CTGF, CTSH, CTSK, CXCL16, CXCR4, CYB561, CYP27A1, DAPK1, DBP, DDB2, DDIT3, DECR1, DEFB4A/DEFB4B, DKK1, DUSP1, DUSP5, E2F2, E2F5, EDNRB, EGR1, EGR2, EGR3, EPOR, ETS2, F2R, F2RL1, FABP5, FAS, FASN, FCER1G, FCGR2A, FGF12, FGF2, FOS, FTH1, FTL, FZD1, FZD2, GAL3ST1, GART, GBP1, GDF15, GLIPR2, GMPR, GNA13, GNB4, GPR19, GPR83, GUSB, HAS2, HBEGF, HIF1A, HK2, HLA-DRA, HLA-DRB1, HLA-F, HMGCR, HMOX1, HSP90AB1, HSPB1, HSPD1, ICAM1, ID1, IER3, IFI16, IFIT2, IFNGR2, IL12A, IL17D, IL1B, IL7R, IL8, IRAK1, IRF4, IRF5, IRF9, IRS2, ISG20, ITGA6, ITGB3, ITPKB, JUN, JUND, KDR, KLF2, KLF4, KRAS, KYNU, LAMC1, LARGE, LGALS3BP, LIF, LIMK1, LOX, LPL, LYN, LZTS1, MAP2K1, MARCKSL1, MITF, MLANA, MMP9, MRAS, MT1E, MTMR3, MYC, NAMPT, NCALD, NEDD9, NFKBIA, NQO1, NUPR1, OAS1, OPN3, OPTN, PANX1, PAPPA, PCSK1, PELI1, PHACTR1, PHLDA1, PI4KB, PIM2, PIM3, PLAUR, PMAIP1, PRAME, PRNP, PTGES, PTGS2, RAB27A, RAB38, RAC2, RARRES1, RARRES3, RTP4, RUNX2, SCLY, SCNN1A, SDC4, SHB, SLC11A2, SLC29A1, SLC2A1, SLC40A1, SLC7A5, SLFN5, SMAGP, SMTN, SNAI2, SOAT1, SOCS2, SOD2, SP100, SP110, SPP1, SQLE, STAT2, SYNM, TBXAS1, TCF7L2, TFDP1, TGFBR2, TGFBR3, TGIF1, THBS1, TLR4, TMEM158, TMOD1, TNFRSF12A, TNFRSF1A, TNFRSF1B, TNFSF10, TNFSF13B, TRAFD1, TXK, TYRP1, UBD, VDR, VEGFA, ZFP36, ZYX

FGF2	-0.341	growth factor	Inhibited	-2.373	5.36E-08	ACTA2, AGAP3, ANG, ANGPT1, ANGPT2, BAX, BCL2, BDNF, BIRC5, CAV1, CCL2, CCND1, CCND2, CDC25A, CDKN1A, CDKN1B, CTSK, CTSL1, CXCR4, DDIT3, EDNRB, EFNB2, EGR1, ENPP2, EPAS1, EPOR, ERRFI1, ETS1, FAS, FGF2, FGFR3, FOS, FOSL1, FOXO1, FOXO3, FOXO4, FTH1, GBP1, HAS2, HBEGF, HIF1A, ICAM1, ID3, IGFBP3, IGFBP5, IL1B, JUN, KDR, KRAS, LOX, MBP, MITF, MMP9, NOV, NR3C1, NR4A2, PLAT, PLAUR, PRKCE, PTGS2, PTPRE, RUNX1, RUNX2, SCG2, SFRP1, SLC20A1, SLC2A1, SPP1, SPRY1, SPRY2, SPRY4, ST3GAL1, ST3GAL4, TGFBR3, THBS1, TIMP3, TNFRSF12A, TNFSF10, TWIST1, UGT2B17, VEGFA, VGF, ZFP57
BRAF		enzyme	Inhibited	-2.218	1.20E-07	BCL2L11, BMF, CCND1, CDKN1A, CDKN1B, CEBPB, DUSP4, EGR1, EPAS1, FOXD3, HIF1A, IL1B, IL8, MMP9, MYC, POU3F2, RND3, THBS1, TSC22D1
EPAS1	0.797	transcription regulator	Inhibited	-2.295	2.17E-07	ACACA, ADM, AKAP12, ANGPT2, ANGPTL4, BHLHE40, BNIP3, CAV1, CCND1, CHKA, CXCR4, DDIT3, FAM13A, FASN, FOS, GAL3ST1, HIF1A, HILPDA, HIST1H2AC, HOXA5, IGFBP3, IGFBP5, IRS2, ITGB3, KDR, KIAA1199, LOX, MAFF, MYOM2, NDRG1, PAN2, PFKFB3, PGF, PLIN2, PTPRZ1, SLC11A2, SLC16A4, SLC29A1, SLC2A1, SLC2A3, SOD2, SPHK1, STC2, TGFA, TMEM45A, TPP2, UGP2, VEGFA, WISP2
CSF1		cytokine	Inhibited	-3.495	2.28E-07	APOE, BAX, BCL2, BCL2L11, BIRC5, CBL, CCL2, CCND1, CCND2, CD97, CDKN1A, CTSK, DUSP1, DUSP5, EGR1, EGR3, ETS2, F2R, F2RL1, FAS, FCER1G, FCGR2A, FOS, GDF15, GRAP2, IL11RA, IL12A, IL1B, IRF4, IRF5, ITGA4, ITGB3, ITGB5, JUN, MAP3K3, MERTK, MMP16, MMP9, MYC, NKIRAS1, RUNX1, SFRP1, SLC29A1, STX3, TNFRSF1A, TNFRSF1B, TRAIP, VEGFA
NRG1		growth factor	Inhibited	-2.902	2.66E-07	ACTN1, ANGPT1, ARHGEF2, BCL2, BNIP3, BRCA1, CCND1, CCND2, CDC42EP1, CDKN1A, CDKN1B, CTGF, DDIT3, DNAJB2, DUSP1, DUSP4, DUSP6, EGR1, EGR2, EGR3, EPHA2, ERRFI1, FOS, FSTL1, G3BP1, HES1, HIF1A, HK2, HMGA1, HMGCR, HMOX1, ID1, IER3, IL8, ITGB3, ITGB5, JUND, LMO4, LPCAT3, MBP, MCL1, MMP9, MST1, MYC, PLAUR, POU3F1, PTGS2, RUNX1, SLC2A1, SLC2A3, SOX4, VEGFA, ZFP36
OSM		cytokine	Inhibited	-2.234	5.30E-07	ABCA1, ABCC1, ABCC4, ADAMTS1, AHCYL1, AHR, AMACR, ANGPT2, ANXA1, ANXA2, ARHGEF2, ARL4A, ASNS, ATP2B4, ATP9A, BAIAP2, BHLHE40, BMI1, BRD8, C1R, C1S, CASP4, CCL2, CCL20, CCND1, CCND2, CCNG2, CDC42EP4, CDKN1A, CDKN1B, CEBPD, CHD1, CTSH, CTSL1, DAPK1, DEFB4A/DEFB4B, DHRS3, DYRK3, ETS2, EXOSC10, FGF2, FOS, GAB1, GART, GBP1, GFPT1, GLE1, GMPR, GOLGA2, HBEGF, HIF1A, HK2, HLA-F, HMG20B, HMOX1, HSF4, HSPA2, ICAM1, ID1, ID2, IL13RA1, IL1B, IL8, IRAK1, IRF9, ISG20, ITPKB, JMJD1C, JUN, KCNG1, KRR1, LARGE, LIF, MARCKS, MGLL, MLLT11, MMP9, MOAP1, MT1X, MT2A, MYC, NAMPT, NUAK1, OAS1, PDLIM5, PEPD, PFKFB3, PLCB4, PLLP, PPP3CC, PTEN, PTGES, PTP4A1, PTPRZ1, QKI, RAP2A, RNASE4, RUNX1, RYK, SERPINB8, SLC16A3, SLC16A6, SMPD1, SON, SORD, SRPK1, ST8SIA1, STK4, TIMP3, TM4SF1, TNC, TOM1, TPX2, TRRAP, TULP3, UAP1, UBQLN2, UPK1A, VDR, VEGFA, ZNF266, ZNF330
TREM1		transmembrane receptor	Inhibited	-3.706	8.72E-07	ABL2, ACSL3, ADORA2B, ARRDC4, ASNS, ATP1B1, BRE, CCL20, CEBPB, CFB, DEFB4A/DEFB4B, DUSP14, DUSP4, EGR1, EGR2, EGR3, EPM2AIP1, ETS2, FABP3, FOSL1, GCLM, GPRC5A, HBEGF, HES1, IFIT2, IL1B, IL8, KANK1, LIF, LPL, MAFF, MCOLN2, MOAP1, MT1E, NFKBIA, NPC1, NR4A2, NT5E, PHLDA1, PHLDA2, PIM2, PPAP2B, PTGS2, RCAN1, RGS1, RHOBTB3, SNAPC1, SPP1, SPRY2, SYNJ2, TBC1D7, THBS1, TLR4, TMEM158, WBP5, YRDC
STAT3		transcription regulator	Inhibited	-2.591	1.51E-06	ADM, ANGPT2, ANGPTL4, ARG2, BATF, BCL2, BCL2L11, BCL3, BIRC5, CASP1, CCL2, CCL20, CCND1, CCND2, CCR1, CD74, CDC25A, CDKN1A, CDKN1B, CEACAM1, CEBPB, CEBPD, CFB, CTSL1, CXCL13, DDIT3, EGR1, EGR2, EGR3, EME1, EPAS1, FAS, FASN, FCER1G, FGF2, FOS, HAS2, HIF1A, HIST2H2AA3/HIST2H2AA4, HK2, HLA-DRB1, HMOX1, ICAM1, ID2, IFI16, IGFBP5, IL1B, IL8, IRF4, ISG20, KDR, KLF4, LIF, LTBR, MAF, MAP2K5, MCL1, MITF, MMP9, MRAS, MT1E, MYB, MYC, NAMPT, NDRG1, OAS1, PAX3, PCSK1, PHB, PHLDA1, PIM2, PLAUR, PMAIP1, PPARGC1A, PTGS2, RAB27A, SLFN5, SMAD6, SOD2, SP110, STC2, TCF4, TFF3, THBS1, TNFRSF1B, TNFSF10, TWIST1, VCAN, VEGFA, ZFP36
IKBKB		kinase	Inhibited	-2.431	2.88E-06	ABCA1, AURKB, BCL2, BRCA1, CCL2, CCL20, CCNA2, CCND1, CCR1, CCRN4L, CDC25B, CDC6, CDKN1A, CEBPB, CEBPD, CKS1B, CTSF, CTSK, CXCR4, DUSP6, EGR1, ENPP2, FAS, FASN, FOS, FOXM1, FOXO3, FYN, GRK5, H2AFX, HIF1A, HK2, HMOX1, ICAM1, IL1B, IL8, ITGB3, ITGB5, KIF20A, MBP, MMP9, MT1E, MYC, MYO1D, NFKBIA, OGN, PCDH7, PTEN, PTGS2, RCAN1, SEMA3C, SFRP1, SOCS2, SOD2, TIMP2, TIMP3, TNFRSF1B, TPM2, TRIM63, TWIST1, VCAN, VEGFA
IL6		cytokine	Inhibited	-2.908	3.21E-06	ABCA1, ABCC1, ABCC2, ADAMTS1, ADRBK1, AHNAK, AHR, ANG, ANXA1, APOE, ATP2A2, BATF, BAX, BCL2, BCL2L11, BCL3, BDNF, BIRC5, BMI1, CASP1, CCL2, CCL20, CCNA2, CCNB2, CCND1, CCR1, CD36, CD74, CD97, CDC25C, CDC6, CDKN1A, CDKN1B, CEACAM1, CEBPB, CEBPD, CENPA, CES2, CPB2, CTGF, CTSK, CXCR4, DEFB4A/DEFB4B, DUSP1, DUSP6, E2F2, EGR1, ENPP2, EOMES, ETS2, F12, FAS, FLI1, FOS, FOXM1, GLRX, GSTA4, HIF1A, HLA-DRB1, HMOX1, HOMER3, HPGD, ICAM1, ID1, ID2, IFI16, IFIT2, IGFBP3, IGFBP5, IL12A, IL7R, IL8, IRF4, IRF9, ITGB3, ITGB5, JUN, JUND, KDR, KIF11, KIF2C, KIT, KRT18, LARGE, LIF, LPL, MAF, MAP2K1, MCL1, MERTK, MMP9, MRAS, MT1E, MYB, MYC, NAMPT, NFKBIA, OSBPL1A, PHB, PLAT, PPP1R15A, PPRC1, PRNP, PROM1, PTGES, PTGS2, SCNN1A SLC39A14, SMOX, SNX10, SOCS2, SOD2, SP110, SPP1, SRC, SV2B, TBC1D9, TBXAS1, THBS1, TK1, TLR4, TNFRSF1A, TNFRSF1B, TRAIP, TTK, TWIST1, UBE2C, VASP, VEGFA, XBP1
MAPK9		kinase	Inhibited	-2.769	3.58E-06	BAX, BCL2, BCL2L11, BMP1, CAV1, CCL2, CDKN1A, CDKN1B, CEBPB, CHERP, EGR1, FOS, FOSL1, GSTM1, HIF1A, HMGA1, IFI16, IL12A, IL1B, IL8, IRF9, ISG20, JUN, JUND, LGALS3BP, LIF, MARCKSL1, MMP9, MYC, NCL, PLA1A, PLAT, PPP1R15A, PTEN, PTGES, RILPL1, SOD2, VDR, ZFP36, ZYX
MAP2K1	-0.339	kinase	Inhibited	-2.436	3.93E-06	ABCC1, ACTA2, AHR, APOE, BCL2, BCL2L11, BRCA1, CAPN3, CCL2, CCND1, CDKN1A, CDKN1B, CTGF, DAB2, DKK1, DUSP1, DUSP5, DUSP6, EGR2, ETV5, F2R, FASN, FGF2, FOS, FOSL1, FURIN, GUSB, HIF1A, IL8, ITGB3, JUN, JUND, MITF, MMP9, MYC, NFKBIA, PLAUR, PTGS2, RAB38, RAP1GAP, SNAI2, THBS1, TNC, TWIST1, VEGFA
ITGB1		transmembrane receptor	Inhibited	-2.184	4.33E-06	ABCC1, ACTA2, APOE, BCL2, CCL2, CDKN1A, CDKN1B, FGFR3, FOS, GAS1, ICAM1, IL1B, IL8, ITGB3, JUN, LAMB1, LAMC1, LIMS1, MMP9, MYC, PTK2, RAC2, TCF4, THBS1, TIMP2, VEGFA
IL5		cytokine	Inhibited	-3.038	8.75E-06	ACAA2, ANXA2, ASNS, ATP1B1, ATXN1, BCL2, BCL3, BNIP3, CASP4, CCL2, CCND2, CCR1, CD55, CEACAM1, CKAP4, CXCR4, DDX21, DUSP5, DUSP6, EGLN1, EGR1, EGR2, EGR3, ERO1L, FAM65B, FAS, GCLM, GLIPR2, HBEGF, HIF1A, HMGCR, HMMR, HSPA6, ICAM1, IER3, IL8, KIAA1147, KLHDC2, MMP9, MYC, NDRG1, NEK2, P4HA1, PDE4B, PMP22, PPIF, PSAT1, RAB21, RAP1GAP, SLC16A3, SLC1A5, SLC2A1, SLC39A8, SLC7A5, SNAP23, SNTB1, SOCS2, SPCS2, SRI, ST7, STK39, TUBB2B, UCK2, UPP1, XBP1, ZYX
ZBTB17	0.295	transcription regulator	Inhibited	-2.183	1.00E-05	BCL2, BHLHE40, BTG3, CCND1, CDKN1A, DDB2, DDIT3, EGR1, IER3, LRRN3, PMAIP1, ZFP36
IL3		cytokine	Inhibited	-4.289	1.42E-05	AKR1A1, ARHGEF1, BCL2, BCL2L11, CALR, CCL2, CCND1, CCND2, CD97, CDKN1A, CDKN1B, COPS4, CTLA4, CXCR4, DDIT3, EGR1, EGR2, EGR3, ELK4, F2R, FAS, FASN, FCGR2A, FOS, FOXD3, GART, GNA13, HBEGF, HK1, HK2, HSPA2, ICAM1, IL1B, IL8, ITGB3, JUN, KLF13, KLF9, LIF, LYN, MCL1, MID1, MKI67IP, MT1E, MYC, NCL, NEDD9, NOV, NRP1, PIM2, PPM1G, RALA, RAN, RANBP1, RPL29, RPL6 RPL7, RPS7, SH3BP1, SLC2A1, SLC2A3, SLC3A2, SOX4, SPEN, TK1, TLR4, TNFSF10, TPD52, VASP, VEGFA, XBP1, YWHAG, ZBTB17, ZFAND5, ZSCAN21
PTGS2	-0.734	enzyme	Inhibited	-2.168	1.65E-05	ANG, ANGPT1, ANGPT2, ANXA1, ANXA2, BAX, BCL2, BRCA1, CCL2, CCND1, CDK5, CDKN1A, CDKN1B, CDKN2C, CTSK, CXCR4, DUSP1, DYNLL1, EGR1, FOS, ICAM1, IGFBP3, IL12A, IL1B, IL8, ITGA6, KDR, MCL1, MMP9, MYC, NOP2, NR4A2, PPA1, PTGS2, RUNX2, TNFSF10, VEGFA
CCL5		cytokine	Inhibited	-3.077	1.95E-05	AHR, CCL2, CCR1, CD97, DUSP1, DUSP6, F2R, F2RL1, FOS, HMGA1, IL12A, IL1B, IL8, LIMS1, MMP9, NAMPT, PLAUR, PNP, PPIF, PTGDS, SQLE, TLR4, VASP, ZFP36
NRIP1	-0.295	transcription regulator	Inhibited	-2.36	2.45E-05	ACAA2, ACACA, ACO2, CCNB2, CCND1, CCNG2, CDC6, CDKN1A, FASN, HADHB, HAS2, IL1B, PDK4, PTGS2, RARB, SDHB, SLC16A10, SLC25A19, SLC25A20, SMAD6, SUOX, TK1
HRAS		enzyme	Inhibited	-2.002	3.66E-05	ADM, ALDH1A3, AMACR, ANXA2, ASNS, ATP2A2, ATXN1, AURKB, B4GALT3, BCL2, BCL2L11, BIRC5, BMP1, BNIP3, CAV1, CCNA2, CCND1, CCND2, CD97, CDC25A, CDKN1A, CDKN1B, CEBPB, CEBPD, CECR5, CFB, COL5A2, CSRP2, CTGF, DDIT3, DUSP1, DUSP6, EGR1, ELK3, ETS1, F2R, FABP3, FAM167A, FAS, FGF2, FOS, FOSL1, FRMD6, FSTL1, FSTL3, FURIN, FZD1, FZD2, GNA13, GUSB, HAS2, HIF1A, HMOX1, HSPB1, ICAM1, ID2, IGFBP5, IL13RA1, IL1B, IL8, ITGA4, ITGA6, ITGB5, JUN, KRAS, KRT18, LRRC17, LXN, MCAM, MMP9, MRPL12, MSMO1, MYC, NFKBIA, NOP58, NQO1, NRP1, NSFL1C, PAFAH1B3, PDLIM5, PGF, PLAUR, PMEPA1, PRNP, PTEN, PTGS2, RAD9A, RAP1GAP, RARB, RASA3, RNF19B, RTN4, SASH1, SCNN1A, SESN1, SESN3, SOAT1, SORBS1, SOX4, SPP1, SPRY2, SQLE, STK10, TACC3, THBS1, TIMP3, TK1, TOM1, TOX2, TPM2, TSC22D1, TWIST1, UBASH3B, VCAN, VEGFA
IL1B	-0.471	cytokine	Inhibited	-3.529	3.99E-05	ABCC2, ACTA2, ADAMTS1, ADM, ANGPT1, ANGPTL4, ANXA1, APOE, ATP2A2, BAX, BCL2, BCL3, BDNF, BMF, C1R, CASP4, CCL2, CCL20, CCL28, CCR1, CCRN4L, CD55, CD74, CDKN1A, CEBPB, CEBPD, CFB, CPB2, CSRNP1, CTSF, CXCR4, DAB2, DBP, DDIT3, DDIT4, DEFB4A/DEFB4B, DUSP1, DUSP5, E2F2, EFNA1, EGR1, ENPP2, EPAS1, ERRFI1, F2RL1, FABP5, FAM129A, FAS, FGF2, FGFR3, FOS, FOSL1, FOXO1, FST, GBP1, GDF15, GHR, GUSB, HAS2, HBEGF, HES1, HEXA, HIF1A, HK2, HLA-DRA, HMGA1, HMOX1, HSPB1, ICAM1, ID3, IER3, IGFBP3, IGFBP5, IL12A, IL16, IL1B, IL1RAP, IL8, IRAK1, IRS2, ISG20, ITGB3, ITPKB, JUN, KDR, LAMB3, LIF, LOX, MAP2K6, MARCKSL1, MCL1, MIA, MMP9, MT1E, MT2A, MYC, NAMPT, NFKBIA, NQO1, NR4A2, P2RX7, PAPPA, PCDH7, PCSK1, PDE4B, PHLDA1, PIM3, PLAT, PLXDC2, PPARGC1A, PTGDS, PTGES, PTGS2, PTP4A1, RAC2, RARA, RASA2, RCAN1, RRS1, RUNX2, RXRA, SCLY, SCNN1A, SDC4, SESN1, SLC11A2, SLC20A1, SLC2A1, SOCS2, SOD2, SPP1, TGFBR2, THBS1, TIMP2, TIMP3, TK1, TLR4, TNFRSF1B, TNFSF10, TRAFD1, TSC22D3, TWIST1, UAP1, UBD, VASP, VCAN, VDR, VEGFA, XYLT1, ZFP36, ZYX
ELK1		transcription regulator	Inhibited	-2.569	6.62E-05	CDKN1A, CDKN1B, EGR1, EGR2, FOS, FOSL1, JUN, MCL1, MMP9, MYLK, PTGS2, RUNX2, SLC2A1, SPP1, ZFP36

EGR1	-1.389	transcription regulator	Inhibited	-2.105	8.87E-05	ATP2A2, BAX, BCL2L11, CACNA1H, CAV1, CCL2, CCND1, CCND2, CCR1, CDK5R1, CDKN1A, DUSP4, EGR1, EGR2, FAS, FGF2, FOSL1, FTL, GDF15, HMGCR, HMOX1, ICAM1, IL1B, IL8, JUN, JUND, MMP9, MYB, MYC, NDRG1, PTEN, PTGES, PTGS2, SOD2, SQLE, TGFBR2, THBS1, TLR4, TNFSF10, VEGFA
F2R	-0.373	G-protein coupled	Inhibited	-2.825	1.00E-04	ANGPT1, ANGPT2, CCL2, CCND1, CD55, CTGF, DUSP1, EGR1, F2R, FOS, HMOX1, ICAM1, IL1B, IL8, KDR, PTGS2, THBS1, VEGFA
PDGFB		growth factor	Inhibited	-3.34	1.00E-04	ACTA2, EGR1, FOS, FOXO1, FOXO3, FOXO4, ICAM1, IL8, KLF2, MMP9, MYC, NOV, PHLDA1, PTEN, PTGS2, SPP1, THBS1, TNC
CD40LG		cytokine	Inhibited	-2.831	1.21E-04	AHR, AKAP13, ALG3, BATF3, BAX, BCL2, BNIP3, BTG1, BTG3, CASP1, CCL2, CCL20, CCNG2, CCR1, CDC25B, CDK6, CDKN1A, CDKN1B, CELF2, CLUH, CSDA, CTGF, CXCL13, CXCR4, DECR1, DUSP1, DUSP4, DUSP5, E2F2, EGR1, FAS, FOS, FURIN, GPR183, HIF1A, HK2, ICAM1, ID2, ID3, IFIT2, IL12A, IL13RA1, IL1B, IL1RAP, IL7R, IL8, IRF4, ITGA4, JUN, JUND, MAP2K3, MARCKS, MARCKSL1, MCL1, METAP1, MT1E, MT1G, MT2A, MYC, NAMPT, NFKBIA, NFKBIE, ORC5, PLAUR, PMAIP1, PTGS2, PTPN12, PVR, RAB9A, RASSF2, RGS1, RUNX3, SEMA4D, SLC29A1, SMG7, SOD2, STK4, TGIF1, TNFSF10, TRADD, TRAF5, UBD, VEGFA, ZZZ3
EDN1		cytokine	Inhibited	-4.167	1.56E-04	ACTA2, ADAM19, ANXA1, ARHGEF11, ATF6, ATP2A2, BAG2, BCL2, CCND1, CDC25A, CDKN1B, CTGF, EDNRB, EGR1, ERRFI1, FGF2, FOS, FOSL1, FST, HEY1, HIF1A, ICAM1, IL8, ITGB3, JUN, MAP2K1, MARCKSL1, MBP, MCAM, MITF, MMP9, MSN, MYC, NUPR1, PLAUR, PLCB4, PRKCE, PTGS2, SLC2A1, THBS1, TIMP3, TPM2, VCAN, VEGFA
PRKCE	0.191	kinase	Inhibited	-3.09	1.97E-04	ACTA2, BAX, BCL2, BIRC5, CAV1, CCND1, CD55, CDKN1A, CEBPB, EGR1, FOS, HSP90AB1, IL1B, IL8, JUN, MMP9, MYC, PRKCE, PRKD1, PTGS2, VEGFA
STAT5A		transcription regulator	Inhibited	-2.112	2.48E-04	ARNT, ATM, BAX, BCL2, CASP4, CCND1, CCND2, CDC25C, CDK6, CDKN1A, CDKN1B, CEBPB, EPAS1, EPOR, FAS, FASN, FCER1G, FOS, GJB1, ICAM1, ID1, ID2, LTBR, MAF, MCL1, MYC, NR4A2, RIMKLA, SLC2A1, SOCS2, TNFRSF1A, UGP2, YWHAG, ZFP36, ZP3
MAPK1		kinase	Inhibited	-2.465	2.58E-04	ACO1, APBB3, APOE, ATP1B1, B3GALT4, BCL2, BDNF, BIRC5, C1S, CCND1, CDK5R1, CDKN1A, CDKN1B, CFB, CHST2, DAB2, DUSP1, EGR1, EGR2, ENDOD1, EXOC2, FAIM3, FOS, GBP1, GFOD1, GPR83, HMOX1, IFI16, IFIT2, IGF2BP3, IL1B, IRF9, ISG20, ITGB3, ITPR2, JUN, JUND, L3MBTL1, LAMA4, LGALS3BP, LIF, LZTS2, MBP, MCL1, MMP9, MNS1, NRP1, NT5E, NUPR1, OAS1, OSBPL3, PARP12, PDLIM3, PTGS2, QKI, SCNN1A, SOCS2, SP100, SP110, SPOCK1, SPP1, SPRY2, SPRY4, SPSB1, STAT2, SUOX, TMEM158, TNFRSF1B, TNFSF10, UBE2C, VDR, WIPF1
MAPK3		kinase	Inhibited	-3.611	2.66E-04	B4GALT5, BCL2L11, CCND1, DUSP4, EGR1, FOS, FOSL1, FURIN, IL8, JUN, MCL1, MMP9, MYC, PLAUR, PPARGC1A, PTGES, PTGS2, SPP1, THBS1
IL15		cytokine	Inhibited	-3.129	3.10E-04	ACADS, ACTG2, AHR, ANXA1, BCL2, BCL2L11, BTG1, CALM1 , CCL2, CCND2, CCR1, CD2, CD55, CD59, CD74, CDK6, CEACAM1, CKS1B, CTGF, CXCR4, DDX18, DHRS4, DUSP14, DUSP5, EGR2, ELK3, ETS1, FAS, FYB, FYN, GABPB1, GLDC, GNL2, GPRC5A, HIST2H2AA3/HIST2H2AA4, HNRNPF, IL1RAP, IL7R, IL8, ITGB5, JUN, LEF1, LIF, LYN, MAF, MCL1, MICB, MST1, MT1G, MYC, NFKBIA, NUDT1, OGN, PA2G4, PAFAH1B3, PDE4B, PMAIP1, PNP, PTP4A1, PTP4A2, RAC2, RBBP8, SOCS2, SP100, SRSF7, TGFBR2, TJP2, TNFRSF1A, TNFSF10, UPP1, VDR, XBP1, YARS, ZFP36
PPRC1	-0.393	transcription regulator	Inhibited	-3.622	3.50E-04	CCL20, CLDN1, DDIT4, DUSP5, ERRFI1, FBXL13, GDF15, HK2, IL8, LAMB3, NAMPT, PHLDA1, PTGS2, RND3, SPRR2D, SUN3, TMEM154
IGF1		growth factor	Inhibited	-2.106	4.24E-04	ACACA, ACTA2, ADM, ANGPT2, BAX, BCL2, BDNF, BHLHE40, BIRC5, BRCA1, CCNA2, CCND1, CCND2, CDKN1A, CDKN1B, CSDA, CTGF, DDIT3, DDIT4, DEFB4A/DEFB4B, EFNB2, EGR1, EGR2, EPAS1, FASN, FOS, FOXO1, GHR, H2AFX, HIF1A, HMGCR, HMOX1, ICAM1, ID2, IER3, IFNGR2, IGFBP3, IGFBP5, IL16, IL1B, IL8, IRS2, ITGB5, JUN, LPL, MAFG, MAK16, MBP, MCL1, MYB, MYC, NFKBIA, NOX4, OSBPL7, PHLDA1, PLAUR, PLP1, PTEN, RARB, RUNX2, SLC20A1, SLC2A1, SNAI2, SOX2, SPP1, SQLE, THBS1, TK1, TLR4, TNFRSF12A, TUBB3, TWIST1, VEGFA
MIF		cytokine	Inhibited	-2.96	4.63E-04	ACTA2, C3AR1, CCL2, CCND1, CDKN1B, DUSP1, F2R, F2RL1, FASN, FOS, ICAM1, IL1B, IL8, ITIH5, JUN, MMP9, PTGS2, TLR4, TPH2
CREB1		transcription regulator	Inhibited	-2.941	6.18E-04	ABCA1, ADM, ANGPTL2, APOE, BCL2, BDNF, BHLHE40, CCNA2, CCND1, CEBPB, CEBPD, CR2, CSRNP1, CSRP2, DUSP1, DUSP14, EGR1, EGR2, ERRFI1, FASN, FOS, FRMD6, GPR19, HAS2, HLA-DRA, HMGCR, HMOX1, ID1, IL1B, IRS2, JUN, LSS, MCL1, MNS1, MSMO1, MVK, MYC, NDUFA10, NR4A2, PCSK1, PDXK, PER2, PIM3, PITPNB, PPARGC1A, PPP1R15A, PTGS2, SLC2A3, SMAD6, SOD2, SRPK2, TFAP2A, TOM1, UPP1, VEGFA, ZFP36
HBEGF	-0.161	growth factor	Inhibited	-2.892	6.88E-04	BCL2, CCND1, CCND2, CXCR4, FGF2, HK2, MMP9, NRP1, PDK4, PTGS2, TGFA, VEGFA
GRP		growth factor	Inhibited	-2.689	7.06E-04	CCND1, CDKN1B, FOS, IL8, JUN, MYC, PTGS2, PTK2, RRM1, VEGFA
BCL2	-0.394	transporter	Inhibited	-2.464	7.15E-04	ABCC1, ATP2A2, BAX, BCL2, BCL2L11, BNIP3, CCND1, CDKN1A, CDKN1B, CTSH, FAS, FOS, HIF1A, ICAM1, IL1B, IL8, MCAM, MCL1, MMP9, NDRG1, NFKBIA, PLAUR, PTEN, SPHK1, TIMP2, VEGFA
SYVN1		transporter	Inhibited	-4	8.12E-04	ABCC4, ACSL3, AHR, ATP1B3, BHLHE40, CALM1 , CCND1, CSPG4, CTPS1, CYB561, DAB2, DUSP1, EPHA2, FASN, GPRC5A, HSPB1, IL7R, ITGA6, KCNN4, LGALS3BP, MCAM, NDFIP2, PPAP2B, PTBP1, SLC1A5, SLC20A1, SLC27A3, SLC2A1, SLC2A3, SLC30A1, SLC39A10, SLC3A2, SLC43A3, SLC4A7, SLC7A5, SNAP23
HMGA1	-0.234	transcription regulator	Inhibited	-2.267	8.42E-04	ATM, BCL2, BRCA1, CAV1, CCND1, DAB2, DHCR7, HMGCR, ID3, KIT, MIA, MMP9, MVK, PTGS2, TWIST1
SRC	-0.181	kinase	Inhibited	-3.12	1.22E-03	CAV1, CBL, CCL2, CCND1, CCND2, CDKN1A, DUSP1, F2R, FOS, FOSL1, HAS2, HIF1A, HMOX1, ICAM1, ID1, IL8, JUN, KRT18, MCL1, MMP9, MYC, NQO1, PLAUR, PRKCE, PTGS2, SPP1, SRC, VEGFA
DDX17		enzyme	Inhibited	-2.176	1.67E-03	CCND1, CDKN1A, FOSL1, JUN, MYC
NGF		growth factor	Inhibited	-2.78	1.97E-03	ANXA2, BAX, BCL2, BCL2L11, BDNF, CAV1, CCND1, CDK5R1, CDKN1A, CDKN1B, DLG4, DUSP1, DUSP4, E2F2, EGR1, EGR2, EHD4, EPAS1, FAS, FOS, FOSL1, HMOX1, ID1, JUN, MAP3K11, MAP3K12, MMP9, MT1A, MYC, PLAUR, PPP2R2B, PTEN, SCG2, SLC40A1, TFAP2A, TNFRSF12A, TRPV4, VEGFA, VGF
TNFSF13B	-0.432	cytokine	Inhibited	-2.861	2.04E-03	BAX, BCL2, BCL2L11, CCND2, CR2, DUSP5, ICAM1, IL8, MCL1, MMP9, MYC, NAMPT, PIM2
SPHK1	-0.429	kinase	Inhibited	-2.478	2.66E-03	BCL2L11, CCND1, CDKN1A, CTGF, DEFB4A/DEFB4B, EGR1, IL1B, JUN, MAP2K6, PTGES, PTGS2
ARNT	0.203	transcription regulator	Inhibited	-3.13	3.25E-03	ADM, AHR, BHLHE40, BHLHE41, BNIP3, CAV1, CCND2, ERO1L, FURIN, GAS1, HIF1A, ID2, IRS2, ITGB3, KIF20A, MACF1, MPP1, NQO1, RANBP1, SLC2A1, UBE2C, VEGFA
PTK2	-0.306	kinase	Inhibited	-2.021	3.27E-03	ACTA2, CCND1, CDKN1A, CDKN1B, CSPG4, DSP, FOS, KRT18, MMP9, P4HA1, PRRX1, PTK2, THBS1, TIMP3, TNC
PRKCB		kinase	Inhibited	-2.761	3.27E-03	BAX, CCND1, CDKN1A, EGR1, FASN, FOS, ICAM1, IRS2, JUN, MMP9, MYC, PPARGC1A, PTGS2, SOD2, TGFBR2
CHRM1	0.171	G-protein coupled	Inhibited	-2.412	3.76E-03	CHRM1, EGR1, EGR2, EGR3, FOS, JUN
SMAD3		transcription regulator	Inhibited	-2.454	4.01E-03	ACTA2, BCL2, BCL2L11, CCL2, CCND1, CCND2, CCNG2, CDKN1A, CTGF, DAPK1, DUSP4, EGR1, FOS, FST, FSTL3, GLI2, HAS2, HBEGF, HEY1, HMOX1, ID1, IL1B, ITGB5, JUN, JUND, MMP9, MYC, NOV, RUNX2, SOX2, SPP1, SSTR2, TGFA, THBS1, TIMP3, TNC, TPM2, VDR, VEGFA, ZFP36, ZYX
IL7		cytokine	Inhibited	-2.531	4.25E-03	AHR, BAX, BCL2, BCL2L11, CCND2, CD2, CDK6, CDKN1A, CDKN1B, CEACAM1, CXCR4, DUSP5, FAS, GSR, IL7R, KLF2, LEF1, LIF, MAF, MCL1, MYC, PIM2, PMAIP1, RUNX2, SLC2A1, TNFSF10, UPP1, XBP1
TCF4	0.632	transcription regulator	Inhibited	-2.397	5.21E-03	BIRC5, CCND1, CDKN1A, DKK1, ID2, JUN, MIA, MITF, MYB, MYC, PSD3, SSTR2, TCF7L2, TYRP1, VCAN
E2F3	-0.184	transcription regulator	Inhibited	-2.18	5.33E-03	BIRC5, BMI1, CCNA2, CCND1, CDC25A, CDC6, CDCA4, CDKN1A, E2F2, E2F3, FGF2, MCM10, MCM2, MT1G, MYB, MYC, PPP1R13B, RRM1, TK1
MAP3K1	-0.479	kinase	Inhibited	-2.421	5.92E-03	CCND1, DUSP1, EGR1, FAS, FOS, HMOX1, HSPB1, IL8, JUN, LOXL3, NOV, PLAUR, PRDX1, PTGS2, THBS1, TNC
MAX		transcription regulator	Inhibited	-2.393	6.07E-03	BAX, CCND2, CCNG2, CDC25A, CDKN1A, CDKN1B, DDIT3, DKC1, E2F2, FTH1, HMGA1, ID1, ID2, JARID2, LRRN3, MYC, NCL, RBBP8, SCPEP1, TXNIP, UBE2C
NOTCH1		transcription regulator	Inhibited	-2.133	7.18E-03	ACTA2, ADAM19, ANGPT1, BCL2, CCND1, CCND2, CDKN1A, CDKN1B, CEBPD, CTGF, EFNB2, EPHA4, FGF2, FOS, FOSL1, GLI2, HES1, HEY1, HEY2, ICAM1, ID1, IGFBP3, IL8, ITGA6, LEF1, LOX, MCAM, MYC, PTEN, PTGDS, PTGS2, RLBP1, RUNX2, RUNX3, SPP1, TGFBR2, TGFBR3
GRN		growth factor	Inhibited	-2.201	7.55E-03	ANGPT1, BCL2, CCND1, FZD2, VEGFA

CXCL12		cytokine	Inhibited	-3.021	8.25E-03	ACTA2, BAX, BCL2, BCL3, BMP1, CCL2, CCND1, CD36, CTSK, CXCR4, EGR1, FOS, FYN, GAS2, GSR, HNRNPD, ICAM1, IFNAR2, IFNGR2, IL8, ITGB3, JMJD1C, JUN, MAPRE3, MARCKS, MMP9, MYC, PPEF1, PTGS2, ROCK1, RUNX3, SORBS3, TNFRSF1B, TNFSF10
FOXL2		transcription regulator	Inhibited	-2.001	8.79E-03	CCL20, FOS, FST, ICAM1, IER3, IL12A, LIF, MAFF, NR5A2, PPARGC1A, PPP1R15A, PTGS2, SMAD6, SOD2, SOX4, SPRY1
CSF2RB		transmembrane receptor	Inhibited	-2.219	1.05E-02	BCL2, CCND2, FOS, JUN, MYC, SNAP23
HDAC5		transcription regulator	Inhibited	-2.646	1.11E-02	CDKN1A, DAPK1, HES1, HK2, HLA-DRA, MAP3K3, MEF2C, MYC, PPARGC1A, PTEN, TNFRSF1A
MAP3K3	0.492	kinase	Inhibited	-2.2	1.24E-02	ADSSL1, BCL2, FOS, HAS2, IL8, JUN, SNAI2, TGFBR3
EPHB1		kinase	Inhibited	-2.224	1.31E-02	EGR1, EGR2, FOS, JUN, PTGS2
RHOA		enzyme	Inhibited	-2.491	1.34E-02	ACTA2, BAX, BCL2, CCND1, CDKN1A, CDKN1B, CEBPB, CTGF, FOS, GDF15, ICAM1, IL8, JUN, MMP9, RND3, RUNX2, SNAI2
IL17A		cytokine	Inhibited	-2.206	1.49E-02	BCL2, CCL2, CCL20, CEBPB, CEBPD, CTGF, CXCL13, CYTH3, DEFB4A/DEFB4B, DLX1, FAS, FOS, GUSB, HBEGF, HSPB8, ICAM1, IL16, IL1B, IL8, ITPR2, JUN, LOX, MMP9, NRP1, PTGS2, SPSB1, TIMP2, TLR4, VEGFA, YWHAG
CR2	0.122	transmembrane receptor	Inhibited	-2	1.54E-02	BCL2, CR2, FAS, IL1B
DNMT3B		enzyme	Inhibited	-2.688	1.63E-02	ABCD1, AHCTF1, BATF3, CDC25C, CDKN1A, EMILIN2, EPM2AIP1, HOXB13, IRF5, KDELR3, LONRF1, LXN, MAPRE3, MGST1, MID1, PRUNE2, RPP25, SLC30A1
MAP2K5	0.296	kinase	Inhibited	-2.411	1.68E-02	CCND1, FOS, JUN, KLF2, MEF2A, MMP9, PTGS2
FGFR1		kinase	Inhibited	-2.418	1.85E-02	BIRC5, CCND1, CCND2, CDKN1A, DNAJC1, EIF4G1, EIF5B, FGF2, FGFR3, FOS, HES1, JUN, MYC, PCSK1, PLAUR, PTGS2, PTPRA
TGFA	-1.34	growth factor	Inhibited	-2.907	1.94E-02	BIRC5, CASP1, CCL2, CCND1, CDKN1A, CEBPB, CTGF, ERRFI1, FOS, GJB1, ICAM1, IL8, MMP9, NR4A2, PLAT, PRNP, PTGS2, TGFA, VEGFA
IL1A		cytokine	Inhibited	-2.467	2.07E-02	ABCC2, ADAMTS1, ADORA2B, ALDH1A3, BCL2, BCL3, CCL2, CCL20, CDKN1A, DEFB4A/DEFB4B, F2RL1, FAS, FGF2, FOS, FTH1, GBP1, GNB4, HMOX1, ICAM1, IFNGR2, IGFBP5, IL1B, IL8, IRAK1, ITGB3, JUN, KIT, LIF, LOX, MCAM, MMP9, MT2A, MYC, NFKBIA, NR3C1, PTGES, PTGS2, SOD2, SPP1, TK1, UGT2B17
F3		transmembrane receptor	Inhibited	-2.611	2.22E-02	ANGPT1, CCL2, CTGF, EGR1, IL1B, IL8, MMP9, VEGFA
CXCR1		G-protein coupled	Inhibited	-2	2.69E-02	BAX, BCL2, CCND1, IL8
RELA		transcription regulator	Inhibited	-3.239	2.93E-02	APOE, BCL2, BCL3, BECN1, BIRC5, CAV1, CCL2, CCL20, CCND1, CDKN1A, CDKN1B, CEBPB, CFB, CR2, CXCR4, DDIT3, DEFB4A/DEFB4B, DGCR6, DUSP1, EGR1, FAS, FGF2, FOS, GDF15, GLI2, GRK5, HAS2, HES1, HIF1A, HMOX1, ICAM1, IER3, IFNGR2, IL12A, IL1B, IL7R, IL8, IRF4, JUN, KIT, LYN, MIA, MMP9, MYB, MYC, NAMPT, NFKBIA, NFKBIE, PTEN, PTGDS, PTGES, PTGS2, SDC4, SOD2, STIM1, TWIST1, UBE2H, VASP, VEGFA
IRS2	0.543	enzyme	Inhibited	-2.096	2.98E-02	ACACA, CDKN1B, EGR1, FASN, FOS, HMGCR, LPL, PPARGC1A, SLC2A1, VEGFA
LCK		kinase	Inhibited	-2.2	3.98E-02	ANXA1, CCNA2, CD2, FOS, JUN, KRT18, SOX2
PLAUR	-0.456	transmembrane receptor	Inhibited	-2.592	4.07E-02	ANG, CCL2, CCND1, ITGA6, ITGB3, KDR, MMP9, MYC, PLAUR
A2M		transporter	Inhibited	-3	4.07E-02	ATF6, BCL2, CCND1, DDIT3, EIF2A, FOXO1, MAP3K5, PPP1R15A, XBP1
THPO		cytokine	Inhibited	-2.241	4.37E-02	AURKB, BAX, BIRC5, CCNA2, CCND1, CCND2, FOS, ITGB3, KDR, MYC
CXCR4	-0.78	G-protein coupled	Inhibited	-2.736	4.37E-02	CCL2, CCND1, CXCR4, EGR1, ID1, ID2, IL8, MYC, RUNX2, TNFSF10
CSF2RA		transmembrane receptor	Inhibited	-2.216	4.42E-02	CCND2, FOS, JUN, MYC, SNAP23
TLR4	-0.247	transmembrane receptor	Inhibited	-2.804	4.99E-02	ADM, ADRBK1, ATM, BATF, BCL2, CCL2, CCND2, CD200, CDK6, CEBPD, CFB, CTSK, DAB2, DEFB4A/DEFB4B, E2F5, HBEGF, HHEX, HMOX1, ICAM1, IFIT2, IL12A, IL1B, IL8, ISG20, LMO4, MERTK, METTL1, MMP9, MYC, NFKBIA, PELI1, PLA1A, PLAT, PTGES, PTGS2, RGS1, RILPL1, RXRA, SLC6A12, SLCO3A1, SPP1, ST3GAL1, STAT2, TCF4, TIMELESS, TLR4, TNFSF10, TSC22D1, XBP1
CTNNB1		transcription regulator	Inhibited	-2.799	5.10E-02	ABCC1, ACTA2, ADSS, AHR, AKAP13, ALG3, ANXA1, ARL4A, ATM, BCL2, BIRC5, BMP1, CALM1 , CASC4, CCNA2, CCND1, CCND2, CDKN1A, CEACAM1, CTDSPL, CTGF, DIAPH3, DKK1, EOMES, ETV4, F2R, FAS, FCER1G, FEN1, FOS, FOSL1, FSTL3, FZD7, GHR, GJB1, GLI2, GNA12, HHEX, HMG20B, HOXA5, ID2, ID3, IGFBP5, IL1B, IL8, IRF4, ITGA6, JUN, LAMB1, LEF1, LPL, MAP3K11, MCL1, MITF, MMP16, MMP9, MYC, MYLK, NPTX1, NRCAM, PAX3, PDE4B, PHLDA2, PLAUR, PMP22, POU3F2, PTGS2, RAI14, RCN1, RUNX2, SEC61A1, SEMA3C, SFRP1, SIM2, SLC1A5, SMAD6, SNAI2, SORBS3, SOX2, SOX4, SPP1, STXBP1, SYK, SYNM, TCF4, TCF7L2, TGFA, TIMP3, TLE4, TMEM2, TNC, TSC22D1, TWIST1, VCAN, VEGFA, ZNF624
MYCN		transcription regulator	Inhibited	-2.051	5.22E-02	ABCC1, ARPC1B, BAX, BID, BIRC5, BMI1, CAV1, CCND1, CCND2, CDKN1A, CDKN1B, CKAP4, COL5A2, CTGF, DKK3, E2F2, E2F5, EIF4A1, FRMD6, HK2, HMGA1, HSP90AB1, HSPD1, ID2, JARID2, LRRN3, MXI1, NCL, NUCB1, OLIG1, PHB, PTK2, RPL29, RPL6, RPL7, RPL9, RPS7, SLC25A19, SLC2A1, SORD, TIMP2, TNFRSF1A, WAC, ZEB2, ZFAND5, ZYX
FHL2		transcription regulator	Inhibited	-2	8.77E-02	ACTA2, BCL2L11, CCND1, IL8, MITF, SPP1
LRP1		transmembrane receptor	Inhibited	-2.425	1.07E-01	C1R, C1S, DDIT3, MMP9, PLIN2, PTGS2
C5		cytokine	Inhibited	-2.128	1.13E-01	BCL2, CCL2, CCND1, EFNB2, EGR1, FCGR2A, GDF15, ICAM1, IFNGR2, IL12A, IL1B, IL8, NFKBIA, PLAT, PPP1R15A, SLC25A15, ST6GAL1, VEGFA, ZFP36
IKBKE		kinase	Inhibited	-2.485	1.81E-01	BCL2, CCND1, CDKN1B, IFIT2, IL8, MMP9, MYC, NFKBIA, PTGS2
GHRL		growth factor	Inhibited	-2.195	2.01E-01	ACACA, BCL2, BDNF, FASN, FOS, IL1B, LPL, PTGS2, SPP1
TLR7		transmembrane receptor	Inhibited	-2.06	2.14E-01	BCL2, CCL2, CCL20, CREB5, FAIM3, FAM19A4, FGF2, HIVEP2, ICAM1, IER3, IL1B, IL8, NFKBIA, PLAT, TMEM154
TBK1		kinase	Inhibited	-2.288	2.35E-01	ATM, ICAM1, IFIT2, IL12A, IL1B, IL8, ISG20, PLA1A, PTGS2, RILPL1, SLCO3A1, TSC22D1, VEGFA
HSPD1	-0.238	enzyme	Inhibited	-2.219	2.39E-01	BAX, HSPD1, ICAM1, IL12A, IL1B
CARM1		transcription regulator	Inhibited	-2	2.39E-01	EGR3, ICAM1, MYC, PTGES, STC2
GDNF		growth factor	Inhibited	-2.184	3.01E-01	BDNF, CCND1, CDKN1B, EGR1, EGR2, ITGA6, KIT, SPHK1, STC1, TUBB4A, VASP
IL6ST		transmembrane receptor	Inhibited	-2.579	3.07E-01	ATP2A2, CCND1, EGR1, JUN, MYC, PIM2, TNFRSF1A, TNFRSF1B
HDAC6	0.174	transcription regulator	Inhibited	-2	3.17E-01	BIRC5, HIF1A, JUN, MYC, SRSF2, TPH2
TLR2		transmembrane receptor	Inhibited	-2.012	3.36E-01	CCL2, CCR1, CEBPB, CEBPD, DEFB4A/DEFB4B, DUSP1, HLA-DRB1, HMOX1, ICAM1, IL1B, IL8, IRAK1, ITGA4, KLF2, MMP9, PTGS2, TLR4, VDR, XBP1
IL18		cytokine	Inhibited	-2.131	4.02E-01	BCL2, CCL2, CCL20, CXCL16, FAS, ICAM1, IL12A, IL1B, IL8, IRF9, JUN, MMP9, PTEN, PTGS2, TGFBR2, TLR4, TXK, VEGFA
IL22		cytokine	Inhibited	-3.095	5.01E-01	ACTA2, BCL2, CCND1, DEFB4A/DEFB4B, HMOX1, HSPB1, IL1B, IL8, MCL1, MYC
TLR5		transmembrane receptor	Inhibited	-2.2	5.07E-01	CCL20, DEFB4A/DEFB4B, ICAM1, IL1B, IL8
DNMT1		enzyme	Inhibited	-2.189	1.00E+00	ASIP, BIRC5, CDC25C, CDKN1A, CDKN1B, MT1E
