# Supplementary material for: Synergistic anti-tumor activity and inhibition of angiogenesis by cotargeting of oncogenic and death receptor pathways in human melanoma
Source: Cell Death Dis. 2014 Oct 2;5(10):e1434–. doi: 10.1038/cddis.2014.410 (PMC4649516; doi:10.1038/cddis.2014.410)
Supplement: Supplementary Methods [file cddis2014410x8.pdf]

Grazia et al. Supplementary methods

Genome-wide expression profiling of melanoma cells treated with target-specific inhibitors and TRAIL. The BRB-array Tools (Vers.4.3.0) software was used to identify genes significantly modulated by treatment of melanoma cells with inhibitors, or with TRAIL or with their association. To this end, class comparison was carried out as previously described (27) by a random-variance F-test with a nominal significance level of 0.001. Permutation *P* values for significant genes were computed based on 10,000 random permutations. Pairwise analysis of significance of gene modulation between any two of the treatments was carried out at P=0.01. VENNTURE software (26), which allows Edwards-Venn diagram generation for multiple pharmacological dataset analysis, was used to classify all genes with significant modulation by any of the treatments, including single drug, two drug and three drug combinations. Ingenuity Pathway Analysis (IPA 8.5, www.ingenuity.com) was used to carry out downstream effects analysis and upstream regulator analysis on significantly modulated genes by different treatments. Downstream effects analysis is a computational tool that allows to identify which biological functions are expected to be increased or decreased given the observed gene expression changes. Upstream regulator analysis allows to identify the upstream transcriptional regulators that can explain the observed gene expression changes in the dataset. Both computational tools return results based on p-values and Z score statistics. P values indicate the likelihood of the association between a set of genes and related function, or the likelihood of the overlap between the genes in the dataset and those that are regulated by a predicted transcription factor. The meaning of the Z score statistics is to infer the activation states ("increased" or "decreased") of the identified biological functions and of the predicted transcription factors. Only Z scores greater than 2 or smaller than -2 can be considered significant.
